# The use of Xuanbai Chengqi decoction on monkeypox disease through the estrone-target AR interaction

**DOI:** 10.3389/fmicb.2023.1234817

**Published:** 2023-09-20

**Authors:** Yanqi Jiao, Chengcheng Shi, Yao Sun

**Affiliations:** ^1^School of Science, Harbin Institute of Technology (Shenzhen), Shenzhen, China; ^2^School of Science/State Key Laboratory of Urban Water Resource and Environment, Harbin Institute of Technology (Shenzhen), Shenzhen, China

**Keywords:** Xuanbai Chengqi decoction, monkeypox, network pharmacology, molecular docking, molecular dynamics simulation

## Abstract

**Introduction:**

After COVID-19, there was an outbreak of a new infectious disease caused by monkeypox virus. So far, no specific drug has been found to treat it. Xuanbai Chengqi decoction (XBCQD) has shown effects against a variety of viruses in China.

**Methods:**

We searched for the active compounds and potential targets for XBCQD from multiple open databases and literature. Monkeypox related targets were searched out from the OMIM and GeneCards databases. After determining the assumed targets of XBCQD for monkeypox treatment, we built the PPI network and used R for GO enrichment and KEGG pathway analysis. The interactions between the active compounds and the hub targets were investigated by molecular docking and molecular dynamics (MD) simulations.

**Results:**

In total, 5 active compounds and 10 hub targets of XBCQD were screened out. GO enrichment and KEGG analysis demonstrated that XBCQD plays a therapeutic role in monkeypox mainly by regulating signaling pathways related to viral infection and inflammatory response. The main active compound estrone binding to target AR was confirmed to be the best therapy choice for monkeypox.

**Discussion:**

This study systematically explored the interactions between the bioactive compounds of XBCQD and the monkeypox-specific XBCQD targets using network pharmacological methods, bioinformatics analyses and molecular simulations, suggesting that XBCQD could have a beneficial therapeutic effect on monkeypox by reducing the inflammatory damage and viral replication via multiple pathways. The use of XBCQD on monkeypox disease was confirmed to be best worked through the estrone-target AR interaction. Our work could provide evidence and guidance for further research on the treatment of monkeypox disease.

## 1. Background

Monkeypox, a virus that used to be common around rainforests in Central and West Africa, has spread out globally (Zardi and Chello, 2022). In June 2022, the World Health Organization (WHO) reported that more than 550 confirmed cases of monkeypox were identified in 30 countries and territories worldwide (Hasan and Saeed, 2022). In July 2022, WHO sounded the alarm again, declaring the monkeypox outbreak to be a Public Health Emergency of International Concern (PHEIC) (Shafaati and Zandi, 2023). It was the seventh PHEIC the WHO had declared by far, and was the highest alert level the agency could give (Wilder-Smith and Osman, 2020; Shafaati et al., 2022).

Monkeypox disease is a rare zoonotic disease caused by the monkeypox virus (Dou et al., 2023). It is endemic in central and west Africa, with the greatest concentration in the Democratic Republic of the Congo. Although it was firstly found in captive monkeys, available data suggested that the African rodents were natural hosts. For instance, squirrels, rats, mice, monkeys, marmots and humans were all infected (Chen et al., 2022). There were two genetically distinct branches identified (Hutson et al., 2010), and the Congo Basin (central Africa) branch was reported more frequently than the west African branch. The current outbreak of Monkeypox in 2022 involved multiple countries on different continents, mainly in men who had sex with men (MSM), and its manifestations were related to genital lesions (Abu-Hammad et al., 2023). Up to 22 June 2022, 99% of the 508 confirmed cases of monkeypox in Madrid region of Spain belonged to the MSM population, and the lesions affected the genital, perineum, or perianal area (Iñigo Martínez et al., 2022). It was also identified swollen inguinal lymph nodes as the main features, indicating that sexual transmission was the primary mode of transmission (Letafati and Sakhavarz, 2023). On 6 July 2022, Germany reported 1,304 confirmed cases, which were also concentrated in the MSM population (Patel and Patel, 2023). Sequencing data from countries indicated that the 2022 outbreak was caused by the west African branch of the monkeypox virus. However, the up-to-date research suggested that there were two distinct lineages of the monkeypox virus with separate sources found in the US.

The monkeypox virus is a double-stranded DNA virus that is related to the variola virus (Kaler et al., 2022; Shafaati and Zandi, 2022). The clinical manifestations of human monkeypox are similar to those of smallpox, which often cause rash, fever, chills, and muscle soreness (Reynolds et al., 2019). The fatality rate of monkeypox is about 3% ~ 6%, but relatively higher in children, young adults, and immunodeficient individuals (Ligon, 2004). When monkeypox is complicated with septicemia, meningitis, osteomyelitis, and other diseases, the death rate could be as high as 10% (Patel et al., 2022; Rizk et al., 2022). Up to now, some antiviral drugs and vaccines initially developed in smallpox have been approved for the treatment and prevention of monkeypox (Hung et al., 2022). But the effects of the treatment and prevention are still being investigated (Shamim et al., 2023). In fact, no specific drugs against monkeypox virus have been developed by far (Zovi et al., 2022). In view of the immunomodulatory and antiviral effects of traditional Chinese medicine (TCM) and its long history of clinical applications, this study aims to explore the potential treatment of monkeypox with TCM.

Xuanbai Chengqi decoction (XBCQD) is a TCM consisting of four Chinese herbs, including mineral-based gypsum, herbal rhubarb, bitter almond, and trichosanthes (Hanzlicek et al., 2014). It was reported to improve the disease symptoms effectively and prognosis of acute lung injury (ALI) patients with fewer adverse reactions by protecting lung function, alleviating excessive inflammatory reactions and tissue damage (Zhu et al., 2021). In addition, XBCQD was found to be an alternative treatment for severe infectious lung diseases caused by influenza and severe acute respiratory syndrome coronavirus 2 (SARS-CoV-2) (Huang et al., 2021; Zhu et al., 2021).

In fact, XBCQD is a representative Chinese medicine prescription in the Differentiation of Febrile Disease written by Wu Jutong in Qing dynasty. It has been widely used in China for the treatment of lung injury, pulmonary fibrosis, chronic obstructive pulmonary and other common respiratory diseases. It can effectively reduce phlegm, heat, cough, wheezing, chest tightness, and has less adverse reactions (Wang et al., 2021; Huo et al., 2022). With the spread of monkeypox worldwide, the China National Health Commission and the National Administration of TCM issued the guidelines for the diagnosis and treatment of monkeypox in June 2022, in which XBCQD and other TCM were recommended to treat monkeypox patients with different symptoms (Desai et al., 2022; Warner et al., 2022).

Network pharmacology is a powerful tool to uncover the effective compounds in TCM from a systematical molecular way, integrating multiple open databases and bioinformatics techniques to construct a comprehensive drug-target-disease network (Zhang et al., 2013). Such a multi-component, multi-target network could reveal the mechanism behind the action of the drug (Noor et al., 2022). Molecular docking offers a possible way to reveal the *in vivo* binding patterns of ligand-receptor (Salmaso and Moro, 2018), which could be used to further explore the ligand-receptor relationship (Meng et al., 2011). Molecular dynamics (MD) simulation allows for the study of various ligand-receptor motions based on Newtonian mechanics to assess their long-time stabilities and flexibilities (Hollingsworth and Dror, 2018).

In this work, we first utilized network pharmacology to screen active drug compounds for the monkeypox virus and explored the potential biological mechanism behind the treatment of monkeypox by XBCQD from a systematic and molecular perspective. In total, 5 active compounds and 10 hub targets of XBCQD were screened out. Subsequently, the biological functional network of XBCQD was constructed to elucidate the regulatory way of XBCQD. The results demonstrated that XBCQD played a therapeutic role in monkeypox mainly by regulating signaling pathways related to viral infection and inflammatory response. Molecular docking was used to predict the binding energy scores and patterns between the hub targets and the potential therapeutic compounds. Finally, MD simulation was adopted to simulate the interaction dynamics and calculate the change of binding free energy of the target-compound complex, so as to provide theoretical foundation for the future clinical applications. The main active compound estrone binding to target AR was finally confirmed to be the best therapy choice for monkeypox.

## 2. Materials and methods

### 2.1. Active compounds in XBCQD and the corresponding targets

The TCMSP platform^1^ was adopted to screen out the active compounds in XBCQD under the following standard criteria: the oral availability (OB) ≥ 30% and drug-like likeness (DL) ≥ 0.18, followed by a target search for active compounds by their MOL.IDs. The standard criteria is the general standard for screening Chinese medicine according to the relevant literature (Ru et al., 2014). The active compounds of Trichosanthes pericarpium were collected from the SYMmap database.^2^ The PubChem database^3^ was used to derive the Simplified Molecular Input Line Entry System (SMILES) of the active compounds. The SMILES were then input into the SwissTargetPrediction structural similarity forecast target database^4^ to predict the valid targets. Unmatched names of targets were supplemented by literature review (Richards et al., 2015; Wang et al., 2021). Finally, the Uniprot database^5^ was used to annotate the relevant targets.

### 2.2. The compound-target network

The Cytoscape 3.9.2 software was adopted to prepare the compound-target network file and type file, as well as to conduct the network topology analysis. To construct the compound-target network map, the target map, color, transparency, and size were adjusted according to the connectivity (degree) of the targets.

### 2.3. The targets of monkeypox disease

The targets of monkeypox disease were searched by using OMIM^6^ and GeneCards^7^ databases. Through the search in GeneCards database, “monkeypox” and “monkeypox virus” were set as keywords to get monkeypox related targets. Monkeypox related targets were also obtained by searching “monkeypox” as a keyword in the OMIM database (see footnote 6). The targets symbols information corresponding to monkeypox disease was downloaded. Targets and functions were set to “human” and “VLOOKUP” to match target gene names.

### 2.4. The cross targets

Using the Venny online database,^8^ a reflection of intersections of drug target genes and disease genes, that is, the crossover between potential targets for XBCQD and monkeypox related targets could be identified. These identified crossover targets were considered as potential anti-monkeypox hub targets.

### 2.5. PPI network and cluster analysis

The String^9^ platform was used to construct the PPI network. Potential anti-monkeypox key targets (identified in the “The cross targets” section) were evaluated using the String platform with PPI highest confidence and species limited to 0.900 and homo sapiens. The string PPI analysis results were then uploaded to Cytoscape 3.9.2 software to identify potential hub anti-monkeypox targets. In addition, the Simple Text Data Format (.tsv) files of PPI results were imported into Cytoscape 3.9.2 software to visualize the PPI network. As the number of nodes in the PPI network decreases, its color changes from red to yellow. Nodes that met the requirement of degree centrality were retrieved and identified as the hub targets of monkeypox. In the PPI network, the degree of the node represents the number of edges between it and other nodes. And the number of connections with other nodes indicates the significance of the node (Yu et al., 2022).

### 2.6. Go and KEGG analyses

GO function and KEGG path enrichment were analyzed using Bioconductor^10^ platform in R language. GO analysis of drug therapy gene functions was annotated in terms of biological process (BP), cellular component (CC), and molecular function (MF). KEGG is mainly a pathway analysis, aiming to elucidate the major signaling pathways for drug therapy. After installation of the R package, we introduced the disease target genes with specific parameters. The “Selection identifier” and “List type” were set to “official gene symbol” and “gene list.” The species were defined as “Homo,” “background” and “*Homo sapiens*” in “List.” The target, the minimum overlap, the *p-*value and the minimum concentration were set to “human,” 3, 0.05 and 1.5, respectively. Then we ran the gene ontology function and KEGG pathway on the target genes. We screened and preserved the results of the most important BP, CC, MF, and KEGG pathways. Finally, the corresponding bubble diagram was derived.

### 2.7. Molecular docking

To reveal the binding patterns between the active compounds and the targets, AutoDock Vina software, Discovery Studio 4.5 Client and PyMOL software were used. The 3D structures of the central targets (receptors) were obtained from the RCSB PDB^11^ database and saved into PDB format. The obtained 3D structures (in PDB format) were further processed using PyMOL software (version 2.2.0) to remove water molecules (“solvent removal” command) and small ligands (“organics removal” command). The active compounds in 2D structures in SDF format were downloaded from PubChem website (see footnote 3). The files were then converted into PDB format using the Open Babel software (version 2.4.1). Hydrogen and Gasteiger charges were added to the above receptors and ligands using AutoDock Vina software, and then saved into PDBQT format. The AutoGrid tool of AutoDock Vina software was used to set the interfacing frame parameters, including the grid box that contained the entire system. The parameter was set to Lamarck Genetic Algorithm (LGA), which generated 10 docking results for each ligand and corresponding receptor. All the docking results were visualized by PyMOL software. Finally, the optimal docked structure could be obtained based on the docking scores of all the possible docked structures.

### 2.8. MD simulation

The long-range electrostatic interactions were calculated using the particle mesh Ewald (PME) method. The target was placed in the center of simulation box filled with water TIP3P molecules with distance of 1.2 nm from the box boundary. There were 33 Na+ ions introduced in the water box to neutralize the charge of the whole system. The system was firstly balanced with energy minimization process which ran up to 100,000 steps using the steepest descent algorithm. Then the system was equilibrated to 310 K with v-rescale (velocity rescaling) method and backbone restrained. Subsequently, the system was further equilibrated at constant pressure (1 bar) and constant temperature (310 K). Finally, 200 ns MD test was conducted after all the restraints released. The simulation results were analyzed by Gromacs 5.1.2 built-in tools and our in-house scripts.

## 3. Results

### 3.1. Active compounds in XBCQD and the corresponding targets

In total, 285 compounds were screened out by the TCMSP database, among which 16, 19 and 11 compounds were from herbal rhubarb, bitter almond, and trichosanthes, respectively. And 43 active compounds met the OB ≥ 30% and DL ≥ 0.18 standards, which were selected for further analysis after removal of the duplicates. The TCMSP database was used to obtain the corresponding targets. All targets were then entered into the Uniprot database and normalized by removing the repeated ones. Finally, 116 potential targets were obtained.

### 3.2. Targets of monkeypox virus

**Figure 1:**
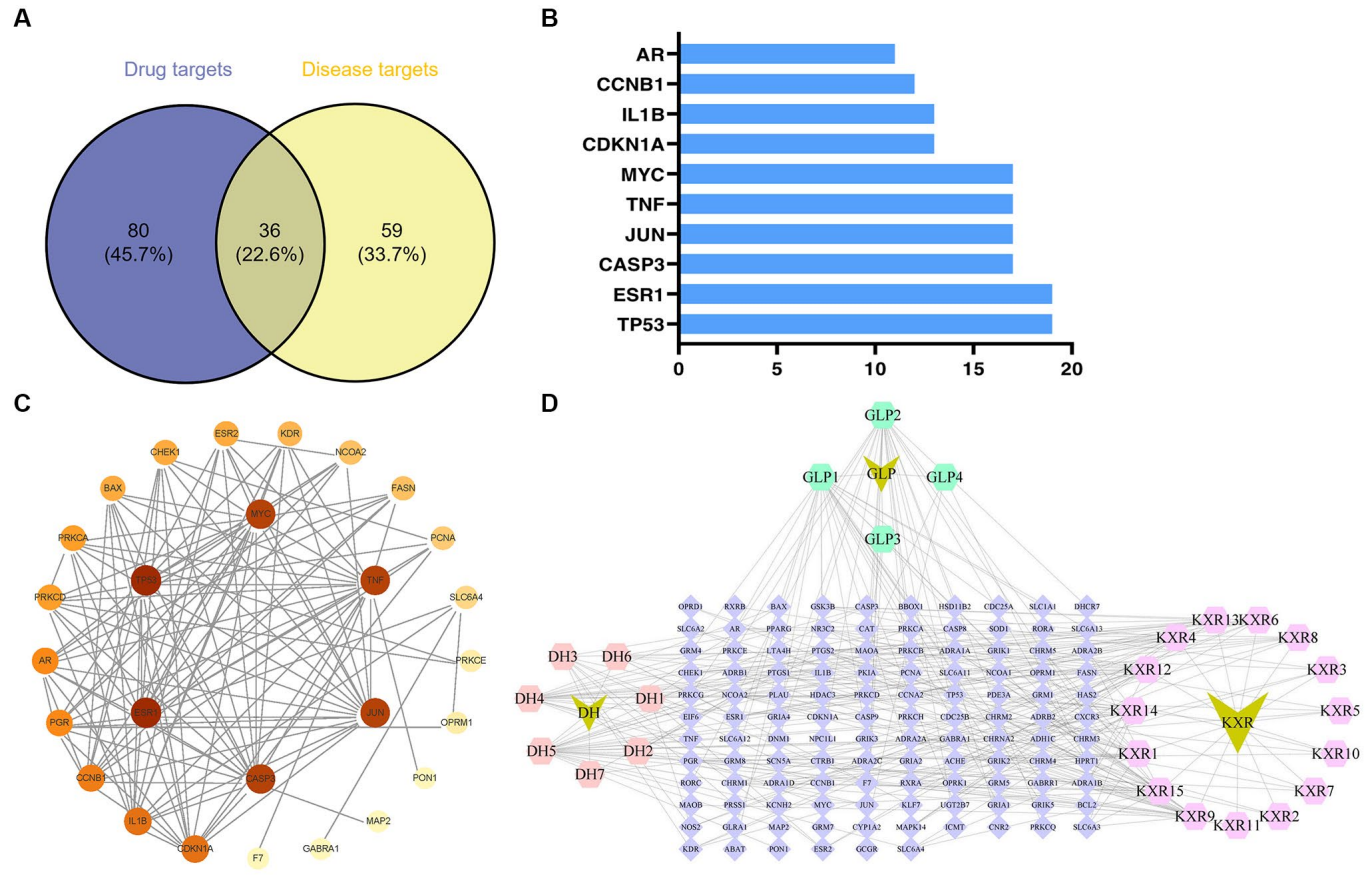
Potential targets of XBCQD and monkeypox, and PPI network. **(A)** Venn diagram of potential gene targets. **(B)** The top 10 monkeypox targets by degree. **(C)** The PPI network of XBCQD for the treatment of monkeypox. **(D)** The compounds-targets network showing potential mechanism of XBCQD for the treatment of monkeypox.

The GeneCards database (Supplementary Table S1) and the OMIM database (Supplementary Table S2) yielded a total of 36 and 95 targets, respectively. After removal of duplicates, the number of related targets was 95. The 116 XBCQD-related targets and the 95 monkeypox virus gene targets were mapped to each other using the online tool Venny 2.1.0 software.^12^ 36 XBCQD-monkeypox virus intersection targets were obtained (Figure 1A). All of the intersection targets were located between the differentially expressed genes in the monkeypox virus dataset.

### 3.3. PPI network analysis

According to the PPI network analysis (Figure 1C), the 36 predicted destinations were imported into String. If a node’s degree, betweenness, and proximity meet certain criteria, it can be designated as a hub node (Wan et al., 2019). The network centrality was used, individually or collectively, to define the network properties of the compounds (degree centrality, betweenness centrality, and closeness centrality) and to judge the importance of the nodes (Valente et al., 2008; Oldham et al., 2019). Nodes with higher levels (larger sizes) were considered to play a more critical role in the network (Chen et al., 2013).

The ranking of the nodes of the most important active compounds in XBCQD was summarized in Table 1, including beta-sitosterol, Stigmasterol, Gamma-Aminobutyric Acid, Phytol, estrone, Machiline, l-SPD, aloe-emodin, Glabridin, and Licochalcone B, etc. The top 10 targets were ESR1 (Estrogen Receptor 1, degree = 19), TP53 (tumor protein p53, degree = 19), CASP3 (cysteine-aspartic acid protease 3, degree = 17), JUN (transcription factor Jun, degree = 17), TNF (Tumor Necrosis Factor, degree = 17), MYC (Cellular myelocytomatosis oncogene, degree = 17), CDKN1A (Cyclin-dependent kinase inhibitor 1A, degree = 13), 1L1B (Interleukin 1 Beta, degree = 13), CCNB1 (G2/mitotic-specific cyclin-B1, degree = 12), and AR (androgen receptor, degree = 11) (Figure 1B). Based on the above compound and target information, a compound-target pathway network was constructed to explain the mechanism of XBCQD against monkeypox virus, as shown in Figure 1D. The yellow V-shaped, light purple hexagon, light pink hexagon, light green hexagon and blue diamond shape represent herb names, KXR targets, DH targets, GLP targets and intersection core targets, respectively.

**Table 1:** Node ranking of the main active compounds.

ID	Name	Degree
MOL000087	beta-sitosterol	28
MOL003035	Stigmasterol	26
MOL000388	Gamma-Aminobutyric Acid	24
MOL001442	Phytol	23
MOL008204	estrone	20
MOL007207	Machiline	20
MOL012922	l-SPD	20
MOL000471	aloe-emodin	19
MOL004908	Glabridin	19
MOL004841	Licochalcone B	13

### 3.4. Go and KEGG pathway enrichment analyses

**Figure 2:**
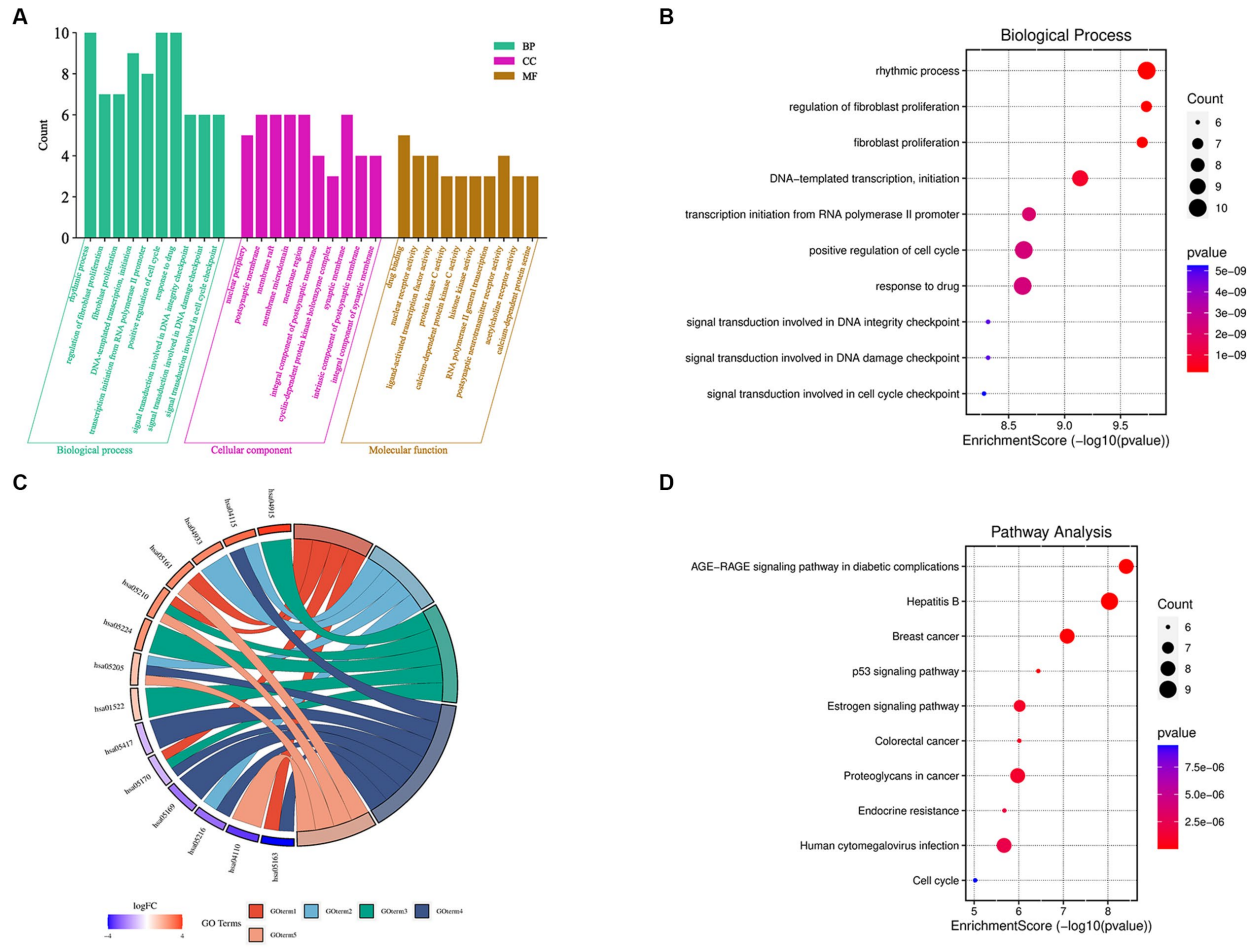
GO function and KEGG pathway enrichment analysis of XBCQD in the treatment of monkeypox. **(A)** GO functional analysis, including BP, CC, and MF. **(B)** Bubble diagram of BP enrichment. **(C)** Gene ontology of the top 10 pathways in XBCQD against monkeypox and **(D)** bubble diagram of KEGG pathway enrichment.

The 36 XBCQD-monkey pox virus intersection targets were imported into the Metascape platform. GO functional enrichment analysis was performed on the targets of the active ingredients in the treatment of monkey pox virus from the levels of BP, CC, and MF (Figure 2A). The top 10 items were selected for visual analysis (Figure 2B). In total, there were 2,455 BP items (Supplementary Table S3). The size of the circles indicates the number of targets, and the darker of the circle indicates the larger log *p* value of the BP item.

It can be found that BP was related to rhythmic process, positive regulation of cell cycle, response to drug, epithelial cell proliferation, DNA-templated transcription, initiation, response to steroid hormone, regulation of epithelial cell proliferation, negative regulation of protein phosphorylation, regulation of epithelial cell proliferation, and transcription initiation from RNA polymerase II promoter. There were 155 items in the CC analysis (Supplementary Table S4), and the top 10 items were selected for visual analysis (Supplementary Figure S1), including postsynaptic membrane, membrane raft, membrane microdomain, presynaptic membrane components, presynaptic membrane components, nuclear periphery, integral component of postsynaptic membrane, integral component of synaptic membrane and intrinsic component of synaptic membrane. In addition, there were a total of 260 items in MF molecular function analysis (Supplementary Table S5), with top 10 entries also visualized (Supplementary Figure S2), which were related to drug binding, nuclear receptor activity, ligand-activated transcription factor activity, protein kinase C activity, calcium-dependent protein kinase C activity, histone kinase activity, RNA polymerase II general transcription initiation factor binding, postsynaptic neurotransmitter receptor activity, acetylcholine receptor activity, and calcium-dependent protein serine/threonine kinase activity. According to the log *p* value in Figure 2D, 10 signaling pathways with high probability were screened according to the enrichment factor value and the number of genes involved in each pathway, which were closely related to the therapeutic mechanism of monkeypox virus. The size of the circle indicates the number of targets, and the darker of the circle indicates the larger log *p* value of the path.

To analyze the significance of hub targets in pathways involved in monkeypox virus treatment, the top 10 pathways determined according to gene counts and adjusted *p* values from the KEGG enrichment analysis and related targets were used to construct the KEGG key pathway network (Figure 2C). According to Figure 2C and Supplementary Table S6, XBCQD in the treatment of monkeypox virus could be mainly related to Hepatitis B (Supplementary Figure S3), AGE-RAGE signal pathway of diabetes complications, Breast cancer, Proteoglycans in cancer, Human cytomegalovirus infection, MAPK signaling pathway, Estrogen signaling pathway, Epstein–Barr virus infection, Human immunodeficiency virus 1 infection and Chemical carcinogenesis-receptor activation. Therefore, XBCQD could target multiple functional and biological factors in the treatment of monkeypox virus, and its effect was mainly reflected in affecting the process of cell proliferation and apoptosis. The components in XBCQD had direct or indirect regulatory effects on the inflammatory response with regard to pruritic inflammation. However, the effects and far-reaching impact are still needed to be further verified.

### 3.5. Molecular docking of compound-target

**Figure 3:**
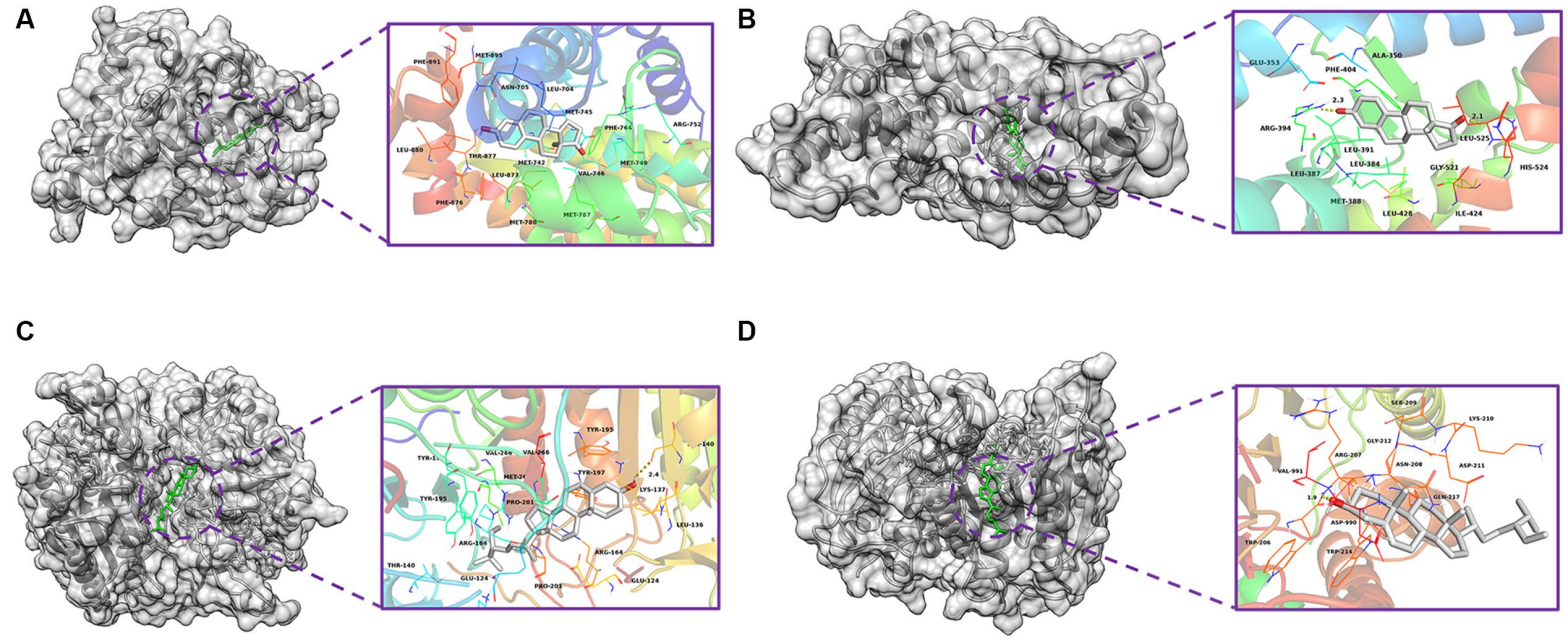
Molecular docking diagrams with 2D and 3D plots. The complexes of **(A)** estrone-AR (−12 kcal/mol), **(B)** estrone-ESR1 (−11.1 kcal/mol), **(C)** Stignasterol-CASP3 (−10.4 kcal/mol), and **(D)** beta-sitosterol-CASP3 (−9.9 kcal/mol).

Based on the monkeypox-related targets and selected compounds from the PPI network, molecular docking was performed. The interactions between the potential active compounds and the hub targets were analyzed using the AutoDock Vina, Discovery Studio 4.5 Client and PyMOL software applications. The selected top 5 active compounds included beta-sitosterol (MOL000087), Stigmasterol (MOL003035), Gamma-Aminobutyric Acid (MOL000388), Phytol (MOL001442) and estrone (MOL008204). The protein structures of hub targets were acquired online from RCSB PDB, including AR (PDB ID: 2QPY), CASP3 (PDB ID: 6BDV), CCNB1 (PDB ID: 2B9R), CDKN1A (PDB ID: 3TS8), ESR1 (PDB ID: 1A52), IL1B (PDB ID: 4DEP), JUN (PDB ID: 1FOS), MYC (PDB ID: 7C36), TP53 (PDB ID: 4MZI) and TNF (PDB ID: 1TNF). The values of affinity energy were obtained by docking analyses (Table 2). Notably, the lower of the affinity energy indicates the stronger of the binding capacity and more stable of the binding conformation. In general, the energy less than −5 kcal/mol indicates that the receptor has some binding ability to the ligand. Our results showed that estrone-AR (−12 kcal/mol), estrone-ESR1 (−11.1 kcal/mol), Stignasterol-CASP3 (−10.4 kcal/mol), and beta-sitosterol-CASP3 (−9.9 kcal/mol) exhibited stronger binding affinities than the other moieties. The docking patterns of all molecules were shown in Figure 3.

**Table 2:** The docking energy scores of the potential active compounds and hub targets.

ID	MOL000087	MOL008204	MOL000388	MOL001442	MOL003035
Name	Beta-sitosterol	Estrone	Gamma-Aminobutyric Acid	Phytol	Stignasterol
AR	−8	−12	−4.3	−6.7	−8.4
CASP3	−9.9	−8.3	−4	−6.1	−10.4
CCNB1	−7.1	−7.3	−4.2	−4.8	−7.3
CDKN1A	−7.3	−7.2	−3.8	−5	−7.5
ESR1	−7	−11.1	−4.3	−5.8	−7.3
IL1B	−6.8	−7	−4.3	−4.4	−7.3
JUN	−8.1	−8.2	−4.7	−5.7	−8.2
MYC	−6.8	−7.6	−4.4	−5.1	−7.1
TNF	−6.6	−6.8	−4.2	−4.3	−6.8
TP53	−7.8	−7.9	−3.8	−5.3	−8.1

### 3.6. Structural stability and interaction energy by MD simulation

**Figure 4:**
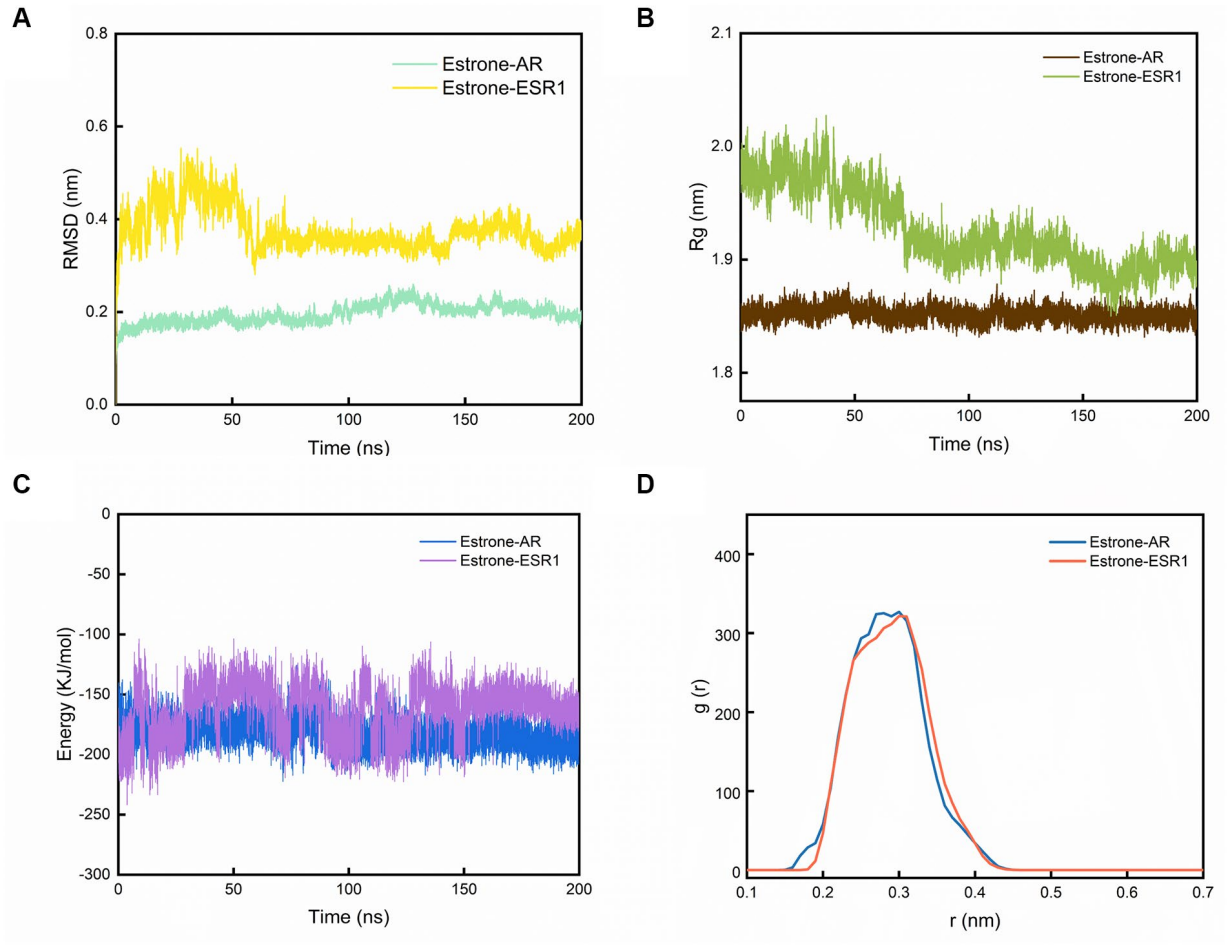
The results of MD simulations. **(A)** Evolutions of the RMSD, **(B)** Rg, **(C)** interaction energy values over 200 ns for the estrone-AR and estrone-ESR1, and **(D)** radial distribution function from estrone to AR and ESR1.

The two best dockings between the compounds and the targets (estrone-AR and estrone-ESR1) were selected to perform the MD simulations. After 200 ns of MD simulations, the dynamic evolutions of estrone-AR and estrone-ESR1 complexes were studied. The conformations of estrone-AR and estrone-ESR1 complexes as well as their contact residues were shown in Figures 4A,B. The interaction energy, radius of gyration (Rg), distance distribution, number of hydrogen bonds, root-mean-square fluctuation (RMSF) and root-mean-square deviation (RMSD) were analyzed accordingly.

As shown in Figure 4A, the RMSD of the last 50 ns for estrone-ESR1 was 0.37 ± 0.06 nm, which was higher than the value of 0.20 ± 0.03 nm for estrone-AR, demonstrating a higher structural flexibility of estrone-ESR1 complex. Subsequently, we analyzed the change of protein cyclotron radius during the 200 ns simulation, which could characterize the compactness of the protein structure. From Figure 4B, it can be observed that the radius of gyration (Rg) of AR basically remained at 1.85 ± 0.02 nm during the whole simulation process, whereas the Rg of ESR1 decreased from 1.97 ± 0.04 nm in the initial 50 ns to 1.89 ± 0.03 nm in the last 50 ns. The evolutions of Rg values were consistent with those of RMSD, indicating that the presence of estrone leaded to a tighter structure of ESR1. According to the changes of interaction energy during the 200 ns simulations shown in Figure 4C, the estrone-AR complex showed lower interaction energy of −186 ± 27 kJ/mol than that of estrone-ESR1 complex (−157 ± 25 kJ/mol) during the last 50 ns. The radial distribution functions from estrone to ESR1 and AR were plotted in Figure 4D. It can be observed that estrone had a slightly closer contact with AR than ESR1.

**Figure 5:**
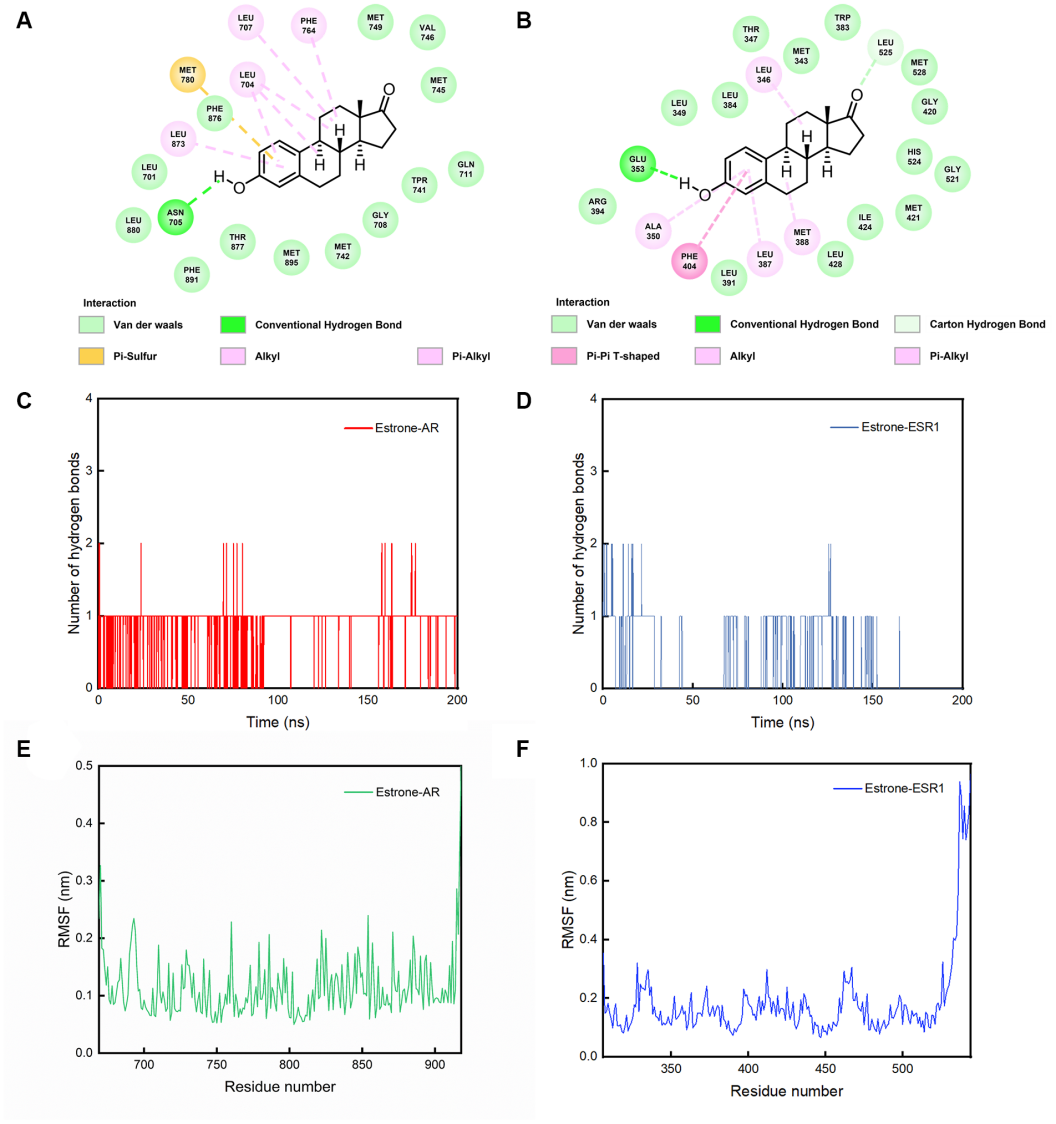
The conformations of contact residues for **(A)** estrone-AR and **(B)** estrone-ESR1 complexes, number of hydrogen bonds between the **(C)** estrone-AR and **(D)** estrone-ESR1 complexes, and **(E)** RMSF curves for AR and **(F)** ESR1 during the 200 ns simulations.

From Figures 5A,B, it can be observed that amino acid Glu 353 of AR and Asn 705 of ESR1 could form hydrogen bonds with phenol hydroxyl of estrone. Comparing Figures 5C,D, it can be clearly seen that AR-estrone were more likely to generate hydrogen bonds and more stable than the ESR1-estrone. It can be seen from Figures 5E,F that the RMSF values of amino acids in AR (residues 676–913: 0.11 ± 0.04 nm) were generally lower than those in ESR1 (residues 312–521: 0.15 ± 0.05 nm). Based on the results of MD simulations, the estrone-AR complex was proposed to possess better interaction stability and binding ability than the estrone-ESR1 complex.

## 4. Discussion

TCM has accumulated a long history of clinical experience and efficacious prescriptions in preventing and treating diseases (Xing and Liu, 2021). It has been demonstrated to inhibit viral replications (Magden et al., 2005), however, the molecular mechanisms behind its effects have not been fully elucidated (Estep et al., 2011). To explore the potential pharmacological and molecular mechanism of XBCQD against monkeypox, we first employed network pharmacology in this study. A total of 36 potential targets associated with monkeypox were identified. Many of the targets were found to be hit by more than one compound. The results indicated that the active compounds of XBCQD could regulate more than one target and could have a synergistic effect on these targets.

The PPI analysis of the 36 targets showed that the top 10 hub targets, including ESR1, TP53, CASP3, JUN, TNF, MYC, CDKN1A, 1L1B, CCNB1, and AR may be the key targets of the treatment of monkeypox virus. The top 5 beta-sitosterol, Stigmasterol, Gamma-Aminobutyric Acid, Phytol, estrone and the top 10 targets were selected in this study. These bioactive compounds were found to effectively bind to the 10 targets according to the results of molecular docking. We further performed GO enrichment analysis on the 36 selected targets to better understand the multiple effects of XBCQD against monkeypox virus from a systematic perspective. The top 10 GO functional categories indicated that XBCQD may exert its effects through the involvement in the BP, MF, and CC. Among these targets, TP53 and ESR1 ranked highest. It was reported that TP53 could be one of the key enzymes in prostaglandin biosynthesis, which had an association with inflammation and mitosis (Costa et al., 2002). In addition, the inhibition of HIV-1, HCV and HSV by the serine protease inhibitor antithrombin III (ATIII) might be the result of TP53-mediated downstream synthesis of arachidonic acids, including prostaglandins (Smee et al., 2014; He et al., 2015; Mussbacher et al., 2019). There was another study suggesting that the inhibition of ESR1 might be a potential therapeutic strategy for SARS-CoV-2 infection (Stilhano et al., 2020; Zhou et al., 2020; Li et al., 2022). Other identified hub targets were reported to be related to immune response and cytokine secretion (Lei et al., 2021). For example, JUN was found to be a stress response gene that altered cell structure during human development (Provençal et al., 2020). IL-6 was one of the classical pro-inflammatory cytokines that directly or indirectly activated a range of different cell types, further causing the secretion of cytokines (Turner et al., 2014; Hirano, 2021).

The concentration of GO terms in hub targets showed that XBCQD treatment of monkeypox was mainly involved the rhythmic process, positive regulation of cell cycle, response to drug, epithelial cell proliferation and DNA-templated transcription, etc. KEGG pathway enrichment analysis of these targets indicated that they were involved in Hepatitis B, AGE-RAGE signal pathway of diabetes complications, Breast cancer, Proteoglycans in cancer, Human cytomegalovirus infection, etc. The AGE-RAGE signaling pathway was reported to have a regulatory role in diabetes (Ramasamy et al., 2011; Kay et al., 2016). Some studies reported that IL-17 regulated viral infections (Ma et al., 2019; Ge et al., 2020; Mills, 2023). As a key cytokine in the pathogenesis of inflammation, TNF was involved in viral infection (Seo and Webster, 2002; Tuazon Kels et al., 2020; Darif et al., 2021). The Hepatitis B pathway was reported as major cellular signaling pathway activated by a variety of viruses (Branda and Wands, 2006; Nguyen et al., 2008; Herrscher et al., 2020). Moreover, estrogen signaling pathway was found to be enriched in both monkeypox infected monkeys and human models (Chadwick et al., 2005; Falcinelli et al., 2016; Xuan et al., 2022). GO and KEGG enrichment analysis suggested that XCBQD may have a positive effect on monkeypox by inhibiting inflammation and viral replication *via* these pathways.

The results of network pharmacology were validated by molecular docking of the top 10 targets and 5 active compounds. The two complexes with lowest binding energy scores were found to be estrone-AR and estrone-ESR1. Furthermore, MD simulations showed that the average value of interaction energy of estrone-AR was −186 kcal/mol (energy drift: 27 kcal/mol), while was lower than the value of −157 kcal/mol (energy drift: 25 kcal/mol) of the estrone-ESR1 complex. Therefore, the interaction of estrone-AR complex was more stable comparing to estrone-ESR1 complex.

In summary, this study systematically explored the interactions between the bioactive compounds of XBCQD and the monkeypox-specific XBCQD targets using network pharmacological methods, bioinformatics analyses and molecular simulations, suggesting that XBCQD could have a beneficial therapeutic effect on monkeypox by reducing the inflammatory damage and viral replication *via* multiple pathways. In addition, the use of XBCQD on monkeypox disease was confirmed to be best worked through the estrone-target AR interaction. By using a series of computational approaches, our study established the drug screening of XBCQD on monkeypox disease for the first time, hopefully could provide some guidance for future drug development.

## 5. Conclusion

By combining network pharmacology, molecular docking and MD simulation, the molecular mechanism of XBCQD in the treatment of monkeypox virus was systematically investigated. According to our results, the top 5 compounds (beta-sitosterol, Stigmasterol, Gamma-Aminobutyric Acid, Phytol, estrone) and the top 10 targets (ESR1, TP53, CASP3, JUN, TNF, MYC, CDKN1A, 1L1B, CCNB1, and AR) were identified. We also found that XBCQD in the treatment of monkeypox virus could be mainly related to Hepatitis B, AGE-RAGE signal pathway of diabetes complications, Breast cancer, Proteoglycans in cancer, Human cytomegalovirus infection, MAPK signaling pathway, Estrogen signaling pathway, Epstein–Barr virus infection, Human immunodeficiency virus 1 infection and Chemical carcinogenesis-receptor activation. Moelcular docking showed that estrone-AR and estrone-ESR1 exhibited stronger binding affinities than the other moieties. And the estrone-AR possessed higher structural and interaction stabilities than the estrone-ESR1 with the extension of molecular simulation time. Our study provides a comprehensive explanation of the multi-component, multi-target and multi-pathway intervention mechanism of XBCQD in the treatment of monkeypox, which is expected to provide a basis and new insights for further pharmacological research.

## Data availability statement

The original contributions presented in the study are included in the article/Supplementary materials, further inquiries can be directed to the corresponding author.

## Author contributions

YS designed the simulations. YJ and CS performed the simulations and analyzed the data. YS, YJ, and CS wrote the manuscript. All authors contributed to the article and approved the submitted version.

## Funding

This work is supported by the National Natural Science Foundation of China (Ref: 12102113) and the Major program of the National Natural Science Foundation of China (T2293720/T2293722).

## Conflict of interest

The authors declare that the research was conducted in the absence of any commercial or financial relationships that could be construed as a potential conflict of interest.

## Publisher’s note

All claims expressed in this article are solely those of the authors and do not necessarily represent those of their affiliated organizations, or those of the publisher, the editors and the reviewers. Any product that may be evaluated in this article, or claim that may be made by its manufacturer, is not guaranteed or endorsed by the publisher.

## Footnotes

^1^ https://tcmsp-e.com/

^2^ http://www.symmap.org/

^3^ https://pubchem.ncbi.nlm.nih.gov

^4^ http://www.swisstargetprediction.ch

^5^ https://www.uniprot.org

^6^ https://www.omim.org/

^7^ https://www.genecards.org/

^8^ https://bioinfogp.cnb.csic.es/tools/venny/

^9^ https://string-db.org/

^10^ http://www.bioconductor.org/

^11^ https://www.rcsb.org/

^12^ http://bioinfo.genotoul.fr/jvenn
