# Supplementary material for: The use of Xuanbai Chengqi decoction on monkeypox disease through the estrone-target AR interaction
Source: Front Microbiol. 2023 Sep 20;14:1234817. doi: 10.3389/fmicb.2023.1234817 (PMC10553791; doi:10.3389/fmicb.2023.1234817)
Supplement: Supplementary file 1 [file Data_Sheet_1.docx]

Supplementary Materials

**The use of Xuanbai Chengqi decoction on monkeypox disease through the estrone-target AR interaction**

*Yanqi Jiao^1,#^, Chengcheng Shi^2,#^, and Yao Sun^1,*^*

*^1^School of Science, Harbin Institute of Technology (Shenzhen), Shenzhen 518055, Guangdong, China.*

*^2^State Key Lab of Urban Water Resource and Environment, School of Science, Harbin Institute of Technology (Shenzhen), Shenzhen 518055, Guangdong, China.*

*^*^To whom correspondence should be addressed. Email: sunyao0819@hit.edu.cn (Dr. Yao Sun).*

*(^#^These authors contribute equally to this work.)*

*^*^*^)^ Corresponding author:

Dr. Yao Sun ([sunyao0819@hit.edu.cn](mailto:sunyao0819@hit.edu.cn))


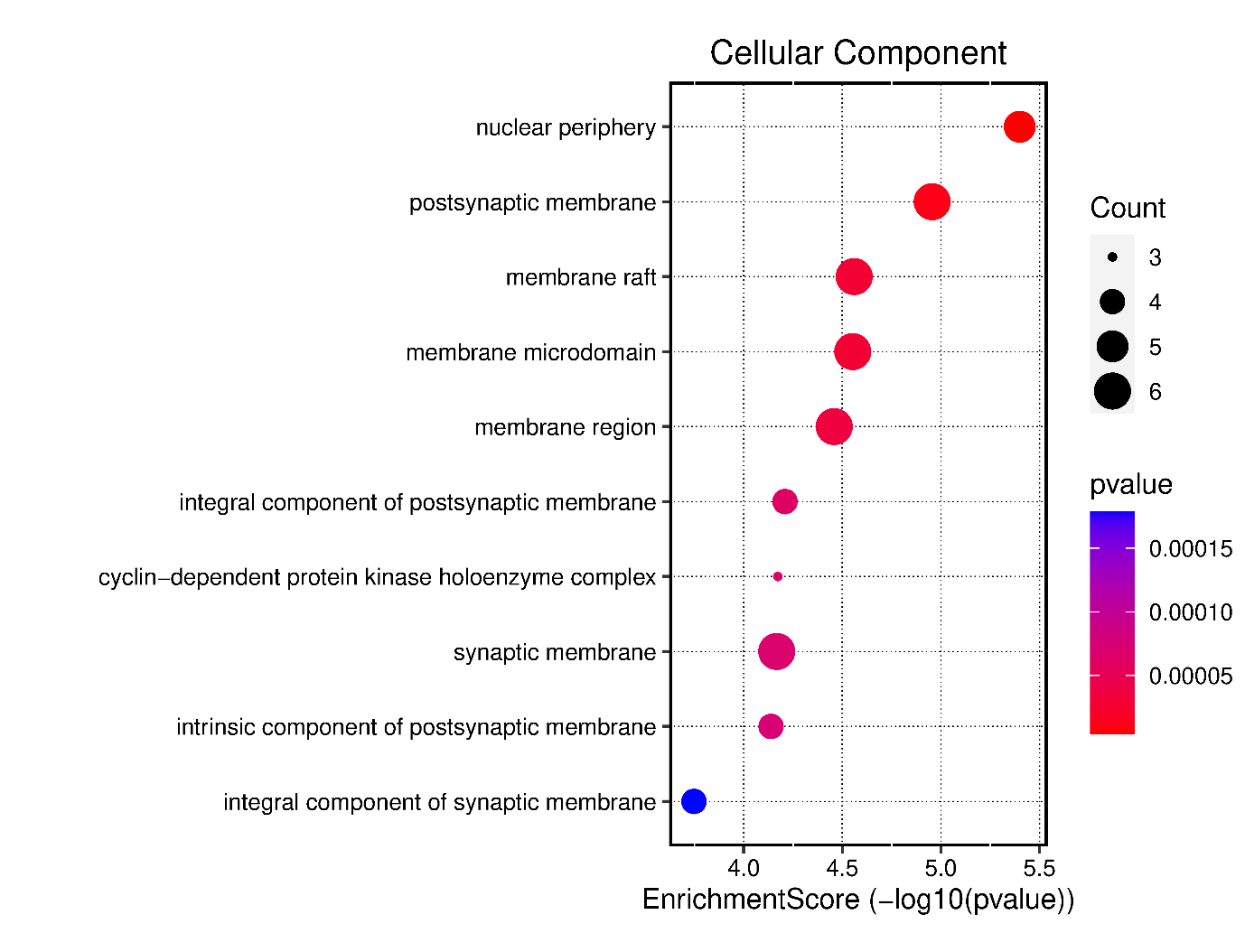


**Figure S1.** Bubble diagram of Cellular Component (CC) enrichment.


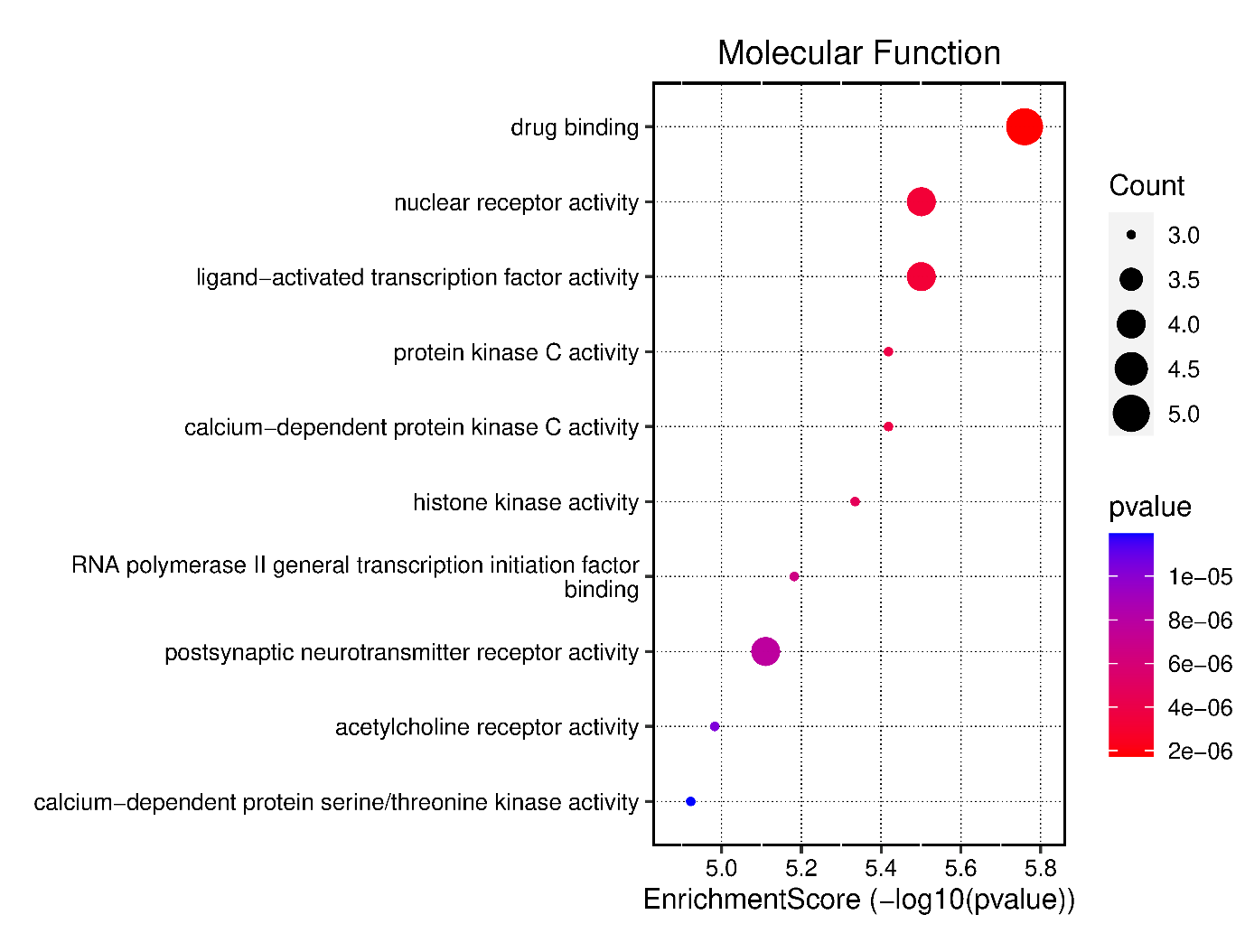


**Figure S2.** Bubble diagram of Molecular Funtion (MF) enrichment.


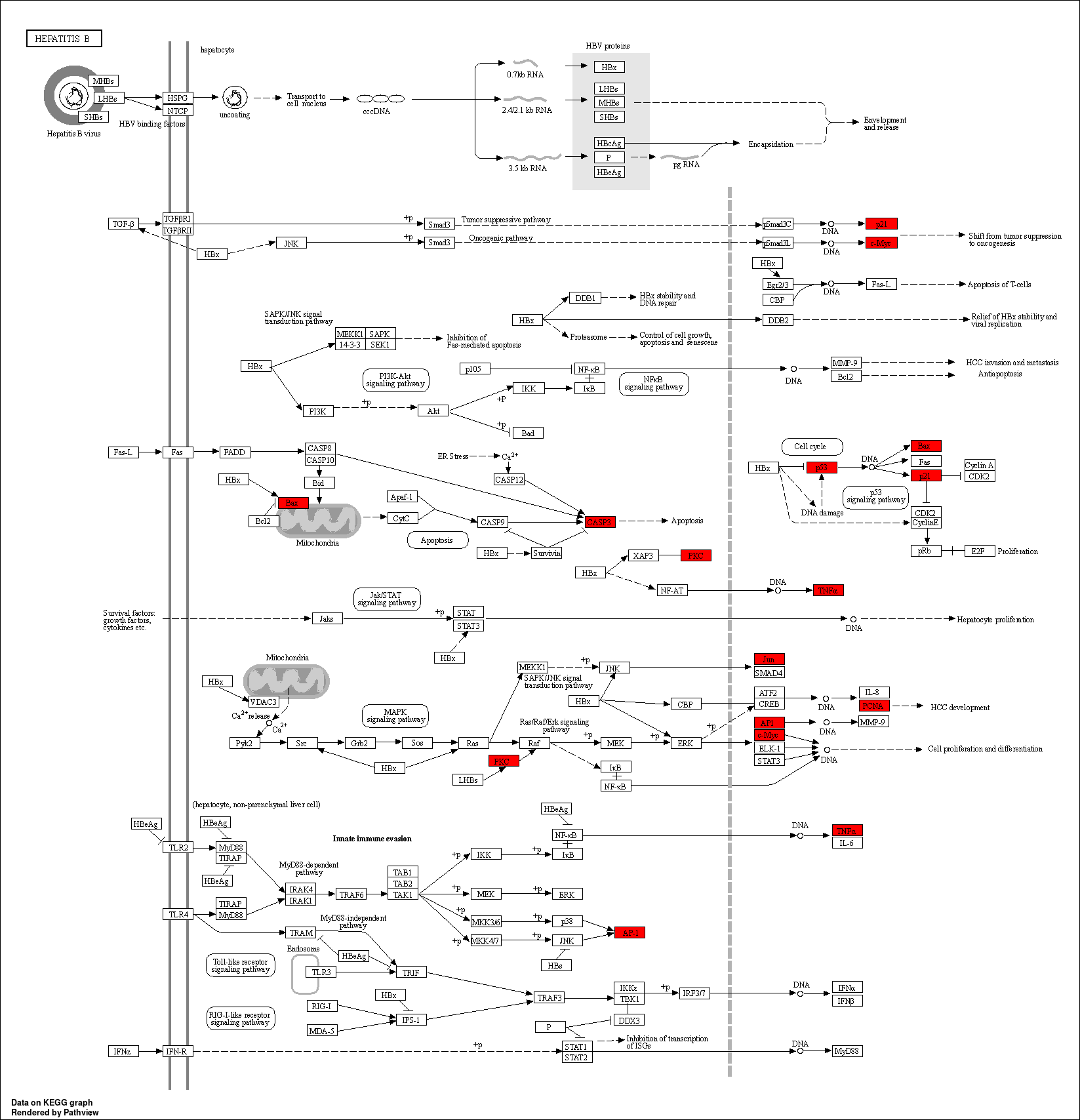


**Figure S3.** The Hepatitis B (HSA05161) pathway by the KEGG mapper, the red represents target of XBCQD in the Hepatitis B pathway.

**Table S1.** Effective targets for monkeypox virus from GeneCards database.

| Gene Symbol | Description | Category | Gifts | GC Id | Relevance score |
| --- | --- | --- | --- | --- | --- |
| CD4 | CD4 Molecule | Protein Coding | 57 | GC12P006786 | 36.63176346 |
| IRF3 | Interferon Regulatory Factor 3 | Protein Coding | 51 | GC19M049659 | 31.23347092 |
| IFNA1 | Interferon Alpha 1 | Protein Coding | 45 | GC09P021622 | 30.14718628 |
| IL6 | Interleukin 6 | Protein Coding | 58 | GC07P022725 | 22.98759651 |
| MX1 | MX Dynamin Like GTPase 1 | Protein Coding | 47 | GC21P041420 | 22.85757828 |
| CD46 | CD46 Molecule | Protein Coding | 55 | GC01P207752 | 22.83252144 |
| ESR2 | Complement C3d Receptor 2 | Protein Coding | 53 | GC01P207454 | 17.92137909 |
| ESR1 | CD8a Molecule | Protein Coding | 54 | GC02M086784 | 15.97548294 |
| CHEK1 | C-X-C Motif Chemokine Ligand 8 | Protein Coding | 50 | GC04P073740 | 15.69495678 |
| NCOA2 | Killer Cell Lectin Like Receptor K1 | Protein Coding | 48 | GC12M023372 | 14.93137741 |
| JUN | Nuclear Factor Kappa B Subunit 1 | Protein Coding | 60 | GC04P102501 | 14.28548813 |
| AR | CD55 Molecule (Cromer Blood Group) | Protein Coding | 56 | GC01P207321 | 13.18649864 |
| SCN5A | Complement C4A (Rodgers Blood Group) | Protein Coding | 50 | GC06P096121 | 8.927423477 |
| PRSS1 | Cytochrome B5 Reductase 3 | Protein Coding | 52 | GC22M063742 | 8.293395042 |
| KDR | C-C Motif Chemokine Ligand 26 | Protein Coding | 44 | GC07M075769 | 7.650188923 |
| F7 | Complement C4B (Chido Blood Group) | Protein Coding | 48 | GC06P032014 | 7.64116478 |
| TP53 | Complement C3b/C4b Receptor 1 (Knops Blood Group) | Protein Coding | 51 | GC01P207496 | 7.561980724 |
| TNF | Schlafen Family Member 13 | Protein Coding | 39 | GC17M035435 | 7.230098724 |
| PRKCE | C-X-C Motif Chemokine Ligand 1 | Protein Coding | 50 | GC04P073869 | 7.081226826 |
| PRKCD | Kelch Like Family Member 2 | Protein Coding | 46 | GC04P165207 | 6.347904205 |
| PRKCA | Ankyrin Repeat Domain 49 | Protein Coding | 43 | GC11P094493 | 5.861476898 |
| PKIA | Twinfilin Actin Binding Protein 2 | Protein Coding | 45 | GC03M052228 | 5.808580875 |
| PCNA | Schlafen Family Member 12 | Protein Coding | 39 | GC17M053823 | 5.719207287 |
| MYC | Crystallin Gamma C | Protein Coding | 46 | GC02M208128 | 5.637978554 |
| IL1B | Olfactory Receptor Family 10 Subfamily G Member 6 | Protein Coding | 26 | GC11M123994 | 5.506860733 |
| FASN | INSYN1 Antisense RNA 1 | RNA Gene | 15 | GC15P126553 | 5.506860733 |
| EIF6 | Long Intergenic Non-Protein Coding RNA 1285 | RNA Gene | 15 | GC0XP118839 | 5.506860733 |
| CDKN1A | Unknown Transcript | RNA Gene | 14 | GC0XP013310 | 5.506860733 |
| CCNB1 | Complement Factor H | Protein Coding | 54 | GC01P196621 | 5.15806675 |
| CASP3 | Heat Shock Transcription Factor 1 | Protein Coding | 53 | GC08P144291 | 3.511297941 |
| BAX | DNA Ligase 4 | Protein Coding | 53 | GC13M108207 | 3.389023066 |
| SLC6A4 | Transmembrane P24 Trafficking Protein 10 | Protein Coding | 49 | GC14M075167 | 2.883323669 |
| PON1 | Exostosin Glycosyltransferase 1 | Protein Coding | 53 | GC08M117798 | 2.876559734 |
| PGR | Transmembrane 9 Superfamily Member 2 | Protein Coding | 42 | GC13P099446 | 2.71171093 |
| PDE3A | BMS1 Pseudogene 20 | Pseudogene | 18 | GC22P022298 | 2.356837988 |
| OPRM1 | ADP-Ribosylarginine Hydrolase | Protein Coding | 43 | GC03P119579 | 1.810405135 |

**Table S2.** Effective targets for monkeypox virus from OMIM database.

| Cytogenetic location | Genomic coordinates (From NCBI/GRCh38) | Gene/Locus | Gene/Locus name | Gene/Locus MIM number | Approved Symbol | Entrez Gene ID | Ensembl Gene ID | Comments | Phenotype | Phenotype MIM number | Inheritance | Pheno map key | Mouse Gene (from MGI) |
| --- | --- | --- | --- | --- | --- | --- | --- | --- | --- | --- | --- | --- | --- |
| 1p35 | 1:27600001-34300000 | EBVS1 | Epstein-Barr virus integration site | 132850 | EBVS1 | 1888 |  |  |  |  |  |  |  |
| 1p34.2 | 1:41506365-42035934 | HIVEP3, KRC | Human immunodeficiency virus type 1 enhancer-binding protein 3 | 606649 | HIVEP3 | 59269 | ENSG00000127124,ENST00000372583;;;ENST00000372583 | | | | | Hivep3 |  |
| 1p22.1 | 1:92508696-92792410 | EVI5, NB4S | Ecotropic viral integration site 5 | 602942 | EVI5 | 7813 | ENSG00000067208,ENST00000684568;;;ENST00000684568 | | | | | Evi5 |  |
| 1q25.3 | 1:185296388-185317243 | IVNS1ABP, NS1BP, ND1, IMD70 | Influenza virus NS1A protein-binding protein | 609209 | IVNS1ABP | 10625 | ENSG00000116679,ENST00000367498;;;ENST00000367498 | | Immunodeficiency 70 | 618969 | Autosomal dominant | 3 | Ivns1abp |
| 1q32.2 | 1:207454328-207489892 | CR2, C3DR, SLEB9, CVID7 | Complement component (3d/Epstein-Barr virus) receptor-2 | 120650 | CR2 | 1380 | ENSG00000117322,ENST00000367057;;;ENST00000367057 | mutation identified in CVID7 patient | ?Immunodeficiency, common variable, 7 | 614699 | Autosomal recessive | 3 | Cr2 |
| 1q32.2 | 1:207454328-207489892 | CR2, C3DR, SLEB9, CVID7 | Complement component (3d/Epstein-Barr virus) receptor-2 | 120650 | CR2 | 1380 | ENSG00000117322,ENST00000367057;;;ENST00000367057 | mutation identified in CVID7 patient | {Systemic lupus erythematosus, susceptibility to, 9} | 610927 |  | 3 | Cr2 |
| 2p16.3 | 2:48313659-48379295 | HTLF | Human T-cell leukemia virus enhancer factor | 143089 | FOXN2 | 3344 | ENSG00000170802,ENST00000340553;;;ENST00000340553 | | | | | Foxn2 |  |
| 2q14-q21 | 2:112200001-136100000 | VIS1, HIS1 | Viral integration site 1 | 164755 | VIS1 | 7435 |  |  |  |  |  |  |  |
| 2q14.1 | 2:112645939-112663825 | SLC20A1, GLVR1 | Solute carrier family 20, phosphate transporter, member 1 (Gibbon ape leukemia virus receptor-1) | 137570 | SLC20A1 | 6574 | ENSG00000144136,ENST00000272542;;;ENST00000272542 | | | | | Slc20a1 |  |
| 2q32.2 | 2:190969149-191014171 | STAT1, CANDF7, IMD31A, IMD31B, IMD31C | Signal transducer and activator of transcription-1 | 600555 | STAT1 | 6772 | ENSG00000115415,ENST00000361099;;;ENST00000361099 | | Immunodeficiency 31A, mycobacteriosis, autosomal dominant | 614892 | Autosomal dominant | 3 | Stat1 |
| 2q32.2 | 2:190969149-191014171 | STAT1, CANDF7, IMD31A, IMD31B, IMD31C | Signal transducer and activator of transcription-1 | 600555 | STAT1 | 6772 | ENSG00000115415,ENST00000361099;;;ENST00000361099 | | Immunodeficiency 31B, mycobacterial and viral infections, autosomal recessive | 613796 | Autosomal recessive | 3 | Stat1 |
| 2q32.2 | 2:190969149-191014171 | STAT1, CANDF7, IMD31A, IMD31B, IMD31C | Signal transducer and activator of transcription-1 | 600555 | STAT1 | 6772 | ENSG00000115415,ENST00000361099;;;ENST00000361099 | | Immunodeficiency 31C, chronic mucocutaneous candidiasis, autosomal dominant | 614162 | Autosomal dominant | 3 | Stat1 |
| 2q34 | 2:211375717-212538802 | ERBB4, HER4, ALS19 | Avian erythroblastic leukemia viral (v-erb-b2) oncogene homolog 4 | 600543 | ERBB4 | 2066 | ENSG00000178568,ENST00000342788;;;ENST00000342788 | | Amyotrophic lateral sclerosis 19 | 615515 | Autosomal dominant | 3 | Erbb4 |
| 3p24.2 | 3:24117153-24495708 | THRB, ERBA2, THR1, PRTH | Thyroid hormone receptor, beta (avian erythroblastic leukemia viral (v-erb-a) oncogene homolog-2) | 190160 | THRB | 7068 | ENSG00000151090,ENST00000646209;;;ENST00000646209 | | Thyroid hormone resistance | 188570 | Autosomal dominant | 3 | Thrb |
| 3p24.2 | 3:24117153-24495708 | THRB, ERBA2, THR1, PRTH | Thyroid hormone receptor, beta (avian erythroblastic leukemia viral (v-erb-a) oncogene homolog-2) | 190160 | THRB | 7068 | ENSG00000151090,ENST00000646209;;;ENST00000646209 | | Thyroid hormone resistance, autosomal recessive | 274300 | Autosomal recessive | 3 | Thrb |
| 3p24.2 | 3:24117153-24495708 | THRB, ERBA2, THR1, PRTH | Thyroid hormone receptor, beta (avian erythroblastic leukemia viral (v-erb-a) oncogene homolog-2) | 190160 | THRB | 7068 | ENSG00000151090,ENST00000646209;;;ENST00000646209 | | Thyroid hormone resistance, selective pituitary | 145650 | Autosomal dominant | 3 | Thrb |
| 3p21.31 | 3:46370142-46376206 | CCR5, CMKBR5, CCCKR5, IDDM22 | Chemokine (C-C) receptor 5 | 601373 | CCR5 | 1234 | ENSG00000160791,ENST00000292303;;;ENST00000292303 | | {Diabetes mellitus, insulin-dependent, 22} | 612522 |  | 3 | Ccr5 |
| 3p21.31 | 3:46370142-46376206 | CCR5, CMKBR5, CCCKR5, IDDM22 | Chemokine (C-C) receptor 5 | 601373 | CCR5 | 1234 | ENSG00000160791,ENST00000292303;;;ENST00000292303 | | {Hepatitis C virus, resistance to} | 609532 |  | 3 | Ccr5 |
| 3p21.31 | 3:46370142-46376206 | CCR5, CMKBR5, CCCKR5, IDDM22 | Chemokine (C-C) receptor 5 | 601373 | CCR5 | 1234 | ENSG00000160791,ENST00000292303;;;ENST00000292303 | | {HIV infection, susceptibility/resistance to} | 609423 |  | 3 | Ccr5 |
| 3p21.31 | 3:46370142-46376206 | CCR5, CMKBR5, CCCKR5, IDDM22 | Chemokine (C-C) receptor 5 | 601373 | CCR5 | 1234 | ENSG00000160791,ENST00000292303;;;ENST00000292303 | | {West nile virus, susceptibility to} | 610379 |  | 3 | Ccr5 |
| 3q22.3 | 3:138160988-138174921 | DBR1 | Debranching RNA lariats 1 | 607024 | DBR1 | 51163 | ENSG00000138231,ENST00000260803;;;ENST00000260803 | | {Encephalitis, acute, infection (viral)-induced, susceptibility to, 11} | 619441 | Autosomal recessive | 3 | Dbr1 |
| 3q22.3 | 3:138347648-138405535 | MRAS, RRAS3, NS11 | Muscle Ras viral oncogene homolog | 608435 | MRAS | 22808 | ENSG00000158186,ENST00000423968;;;ENST00000423968 | | Noonan syndrome 11 | 618499 | Autosomal dominant | 3 | Mras |
| 4q35.1 | 4:186069156-186088073 | TLR3, IMD83 | Toll-like receptor-3 | 603029 | TLR3 | 7098 | ENSG00000164342,ENST00000296795;;;ENST00000296795 | | {HIV1 infection, resistance to} | 609423 |  | 3 | Tlr3 |
| 4q35.1 | 4:186069156-186088073 | TLR3, IMD83 | Toll-like receptor-3 | 603029 | TLR3 | 7098 | ENSG00000164342,ENST00000296795;;;ENST00000296795 | | {Immunodeficiency 83, susceptibility to viral infections} | 613002 | Autosomal dominant; Autosomal recessive | 3 | Tlr3 |
| 5p14 | 5:18400001-28900000 | MLVI2 | Moloney leukemia virus integration site-2 | 157960 |  | 4309 |  |  |  |  |  |  |  |
| 5q33.3 | 5:157029413-157069407 | HAVCR1, HAVCR | Hepatitis A virus cellular receptor 1 | 606518 | HAVCR1 | 26762 | ENSG00000113249,ENST00000523175;;;ENST00000523175 | | | | | BC053393,Gm12169,Havcr1,Timd2 | |
| 5q33.3 | 5:157085832-157109044 | HAVCR2, TIM3, SPTCL | Hepatitis A virus cellular receptor 2 | 606652 | HAVCR2 | 84868 | ENSG00000135077,ENST00000307851;;;ENST00000307851 | | T-cell lymphoma, subcutaneous panniculitis-like | 618398 | Autosomal recessive | 3 | Havcr2 |
| 6p24.1 | 6:12007693-12212048 | HIVEP1, ZNF40 | Human immunodeficiency virus type I enhancer-binding protein-1 | 194540 | HIVEP1 | 3096 | ENSG00000095951,ENST00000379388;;;ENST00000379388 | | | | | Hivep1 |  |
| 6q22.1 | 6:117287353-117425942 | ROS1, MCF3 | Avian UR2 sarcoma virus oncogene (v-ros) homolog 1 | 165020 | ROS1 | 6098 | ENSG00000047936,ENST00000368507;;;ENST00000368507 | | | | | Ros1 |  |
| 6q23.3 | 6:135181308-135219172 | MYB | Avian myeloblastosis viral (v-myb) oncogene homolog | 189990 | MYB | 4602 | ENSG00000118513,ENST00000341911;;;ENST00000341911 | | {T-cell acute lymphoblastic leukemia} | | | 3 | Myb |
| 6q23.3 | 6:137197484-137219385 | IFNGR1, IMD27A, IMD27B | Immune interferon, receptor for | 107470 | IFNGR1 | 3459 | ENSG00000027697,ENST00000367739;;;ENST00000367739 | | Immunodeficiency 27A, mycobacteriosis, AR | 209950 | Autosomal recessive | 3 | Ifngr1 |
| 6q23.3 | 6:137197484-137219385 | IFNGR1, IMD27A, IMD27B | Immune interferon, receptor for | 107470 | IFNGR1 | 3459 | ENSG00000027697,ENST00000367739;;;ENST00000367739 | | Immunodeficiency 27B, mycobacteriosis, AD | 615978 | Autosomal dominant | 3 | Ifngr1 |
| 6q23.3 | 6:137197484-137219385 | IFNGR1, IMD27A, IMD27B | Immune interferon, receptor for | 107470 | IFNGR1 | 3459 | ENSG00000027697,ENST00000367739;;;ENST00000367739 | | {H. pylori infection, susceptibility to} | 600263 |  | 3 | Ifngr1 |
| 6q23.3 | 6:137197484-137219385 | IFNGR1, IMD27A, IMD27B | Immune interferon, receptor for | 107470 | IFNGR1 | 3459 | ENSG00000027697,ENST00000367739;;;ENST00000367739 | | {Hepatitis B virus infection, susceptibility to} | 610424 |  | 3 | Ifngr1 |
| 6q23.3 | 6:137197484-137219385 | IFNGR1, IMD27A, IMD27B | Immune interferon, receptor for | 107470 | IFNGR1 | 3459 | ENSG00000027697,ENST00000367739;;;ENST00000367739 | | {Tuberculosis infection, protection against} | 607948 |  | 3 | Ifngr1 |
| 6q23.3 | 6:137197484-137219385 | IFNGR1, IMD27A, IMD27B | Immune interferon, receptor for | 107470 | IFNGR1 | 3459 | ENSG00000027697,ENST00000367739;;;ENST00000367739 | | {Tuberculosis, susceptibility to} | 607948 |  | 3 | Ifngr1 |
| 6q24.2 | 6:142751469-142946365 | HIVEP2, MRD43 | Human immunodeficiency virus type I enhancer-binding protein-2 | 143054 | HIVEP2 | 3097 | ENSG00000010818,ENST00000367603;;;ENST00000367603 | | Intellectual developmental disorder, autosomal dominant 43 | 616977 | Autosomal dominant | 3 | Hivep2 |
| Chr.6 | 6:1-170805979 | BEVI | Baboon M7 virus replication | 109180 |  | 628 |  |  |  |  |  |  |  |
| 7p15 | 7:20900001-28800000 | MYCLK1 | Avian myelocytomatosis viral (v-myc) oncogene homolog like 1 | 164865 |  |  |  |  |  |  |  |  |  |
| 8q12.1 | 8:55879835-56014169 | LYN | Yamaguchi sarcoma viral (v-yes-1) related oncogene homolog | 165120 | LYN | 4067 | ENSG00000254087,ENST00000519728;;;ENST00000519728 | | | | | Lyn |  |
| 8q13.1 | 8:66562175-66613218 | MYBL1 | Avian myeloblastosis viral (v-myb) oncogene homolog like-1 | 159405 | MYBL1 | 4603 | ENSG00000185697,ENST00000522677;;;ENST00000522677 | | | | | Mybl1 |  |
| 11p15.5 | 11:532242-535576 | HRAS | Harvey rat sarcoma viral (v-Ha-ras) oncogene homolog | 190020 | HRAS | 3265 | ENSG00000174775,ENST00000311189;;;ENST00000311189 | pseudogene HRASP on X | Bladder cancer, somatic | 109800 |  | 3 | Hras |
| 11p15.5 | 11:532242-535576 | HRAS | Harvey rat sarcoma viral (v-Ha-ras) oncogene homolog | 190020 | HRAS | 3265 | ENSG00000174775,ENST00000311189;;;ENST00000311189 | pseudogene HRASP on X | Congenital myopathy with excess of muscle spindles | 218040 | Autosomal dominant | 3 | Hras |
| 11p15.5 | 11:532242-535576 | HRAS | Harvey rat sarcoma viral (v-Ha-ras) oncogene homolog | 190020 | HRAS | 3265 | ENSG00000174775,ENST00000311189;;;ENST00000311189 | pseudogene HRASP on X | Costello syndrome | 218040 | Autosomal dominant | 3 | Hras |
| 11p15.5 | 11:532242-535576 | HRAS | Harvey rat sarcoma viral (v-Ha-ras) oncogene homolog | 190020 | HRAS | 3265 | ENSG00000174775,ENST00000311189;;;ENST00000311189 | pseudogene HRASP on X | Nevus sebaceous or woolly hair nevus, somatic | 162900 |  | 3 | Hras |
| 11p15.5 | 11:532242-535576 | HRAS | Harvey rat sarcoma viral (v-Ha-ras) oncogene homolog | 190020 | HRAS | 3265 | ENSG00000174775,ENST00000311189;;;ENST00000311189 | pseudogene HRASP on X | Schimmelpenning-Feuerstein-Mims syndrome, somatic mosaic | 163200 |  | 3 | Hras |
| 11p15.5 | 11:532242-535576 | HRAS | Harvey rat sarcoma viral (v-Ha-ras) oncogene homolog | 190020 | HRAS | 3265 | ENSG00000174775,ENST00000311189;;;ENST00000311189 | pseudogene HRASP on X | Spitz nevus or nevus spilus, somatic | 137550 |  | 3 | Hras |
| 11p15.5 | 11:532242-535576 | HRAS | Harvey rat sarcoma viral (v-Ha-ras) oncogene homolog | 190020 | HRAS | 3265 | ENSG00000174775,ENST00000311189;;;ENST00000311189 | pseudogene HRASP on X | Thyroid carcinoma, follicular, somatic | 188470 |  | 3 | Hras |
| 11p15.2 | 11:14277920-14364506 | RRAS2, TC21, NS12 | Related Ras viral oncogene homolog 2 | 600098 | RRAS2 | 22800 | ENSG00000133818,ENST00000256196;;;ENST00000256196 | | Noonan syndrome 12 | 618624 | Autosomal dominant | 3 | Rras2 |
| 11p15.2 | 11:14277920-14364506 | RRAS2, TC21, NS12 | Related Ras viral oncogene homolog 2 | 600098 | RRAS2 | 22800 | ENSG00000133818,ENST00000256196;;;ENST00000256196 | | Ovarian carcinoma | |  | 3 | Rras2 |
| 11q13.4 | 11:71998909-72008200 | IL18BP, FVH | Interleukin 18-binding protein | 604113 | IL18BP | 10068 | ENSG00000137496,ENST00000393703;;;ENST00000393703 | mutation identified in 1 FVH patient | {?Hepatitis, fulminant viral, susceptibility to} | 618549 | Autosomal recessive | 3 | Il18bp |
| 11q14.1 | 11:77660009-77872232 | HBXAP, RSF1 | Hepatitis B virus X-associated protein | 608522 | RSF1 | 51773 | ENSG00000048649,ENST00000308488;;;ENST00000308488 | | | | | Rsf1 |  |
| 12q15 | 12:68154768-68159740 | IFNG, IFG, IFI, IMD69 | Interferon, gamma | 147570 | IFNG | 3458 | ENSG00000111537,ENST00000229135;;;ENST00000229135 | mutation identified in 1 IMD69 family | ?Immunodeficiency 69, mycobacteriosis | 618963 | Autosomal recessive | 3 | Ifng |
| 12q15 | 12:68154768-68159740 | IFNG, IFG, IFI, IMD69 | Interferon, gamma | 147570 | IFNG | 3458 | ENSG00000111537,ENST00000229135;;;ENST00000229135 | mutation identified in 1 IMD69 family | {AIDS, rapid progression to} | 609423 |  | 3 | Ifng |
| 12q15 | 12:68154768-68159740 | IFNG, IFG, IFI, IMD69 | Interferon, gamma | 147570 | IFNG | 3458 | ENSG00000111537,ENST00000229135;;;ENST00000229135 | mutation identified in 1 IMD69 family | {Aplastic anemia} | 609135 |  | 3 | Ifng |
| 12q15 | 12:68154768-68159740 | IFNG, IFG, IFI, IMD69 | Interferon, gamma | 147570 | IFNG | 3458 | ENSG00000111537,ENST00000229135;;;ENST00000229135 | mutation identified in 1 IMD69 family | {Hepatitis C virus, response to therapy of} | 609532 |  | 3 | Ifng |
| 12q15 | 12:68154768-68159740 | IFNG, IFG, IFI, IMD69 | Interferon, gamma | 147570 | IFNG | 3458 | ENSG00000111537,ENST00000229135;;;ENST00000229135 | mutation identified in 1 IMD69 family | {TSC2 angiomyolipomas, renal, modifier of} | 613254 | Autosomal dominant | 3 | Ifng |
| 12q15 | 12:68154768-68159740 | IFNG, IFG, IFI, IMD69 | Interferon, gamma | 147570 | IFNG | 3458 | ENSG00000111537,ENST00000229135;;;ENST00000229135 | mutation identified in 1 IMD69 family | {Tuberculosis, protection against} | 607948 |  | 3 | Ifng |
| 12q22 | 12:94148577-94307675 | VESPR | Virus-encoded semaphorin protein receptor | 604259 | PLXNC1 | 10154 | ENSG00000136040,ENST00000258526;;;ENST00000258526 | | | | | Plxnc1 |  |
| 14q12 | 14:24161265-24166565 | IRF9, ISGF3, ISGF3G | Interferon regulatory factor 9 | 147574 | IRF9 | 10379 | ENSG00000213928,ENST00000396864;;;ENST00000396864 | | Immunodeficiency 65, susceptibility to viral infections | 618648 | Autosomal recessive | 3 | Irf9 |
| 15q26.1 | 15:90884504-90895776 | FES | Oncogene FES, feline sarcoma virus | 190030 | FES | 2242 | ENSG00000182511,ENST00000328850;;;ENST00000328850 | | | | | Fes |  |
| 16p13.2 | 16:8892097-8963906 | USP7, HAUSP, HAFOUS | Ubiquitin-specific protease-7, herpes virus-associated | 602519 | USP7 | 7874 | ENSG00000187555,ENST00000344836;;;ENST00000344836 | | Hao-Fountain syndrome | 616863 | Autosomal dominant | 3 | Usp7 |
| 16q23.1 | 16:74296814-74306288 | PSMD7, MOV34 | Proteasome 25S subunit, non-ATPase, 7 (Moloney leukemia virus-34 proviral integration homolog) | 157970 | PSMD7 | 5713 | ENSG00000103035,ENST00000219313;;;ENST00000219313 | | | | | Psmd7 |  |
| 17p13.3 | 17:1420693-1456232 | CRK | Avian sarcoma virus CT10 (v-crk) oncogene homolog | 164762 | CRK | 1398 | ENSG00000167193,ENST00000300574;;;ENST00000300574 | | | | | Crk |  |
| 17q11.2 | 17:31303770-31314054 | EVI2B, EVDB | Ecotropic viral integration site 2B | 158381 | EVI2B | 2124 | ENSG00000185862,ENST00000330927;;;ENST00000330927 | within the NF1 gene | | |  | Evi2,Evi2b | |
| 17q11.2 | 17:31316410-31321622 | EVI2A, EVI2, EVDA | Ecotropic viral integration site 2A | 158380 | EVI2A | 2123 | ENSG00000126860,ENST00000462804;;;ENST00000462804 | within the NF1 gene | | |  | Evi2a |  |
| 17q12 | 17:39688094-39728658 | ERBB2, NGL, NEU, HER2, VSCN2 | Avian erythroblastic leukemia viral (v-erb-b2) oncogene homolog 2 (neuro/glioblastoma derived oncogene homolog) | 164870 | ERBB2 | 2064 | ENSG00000141736,ENST00000269571;;;ENST00000269571 | mutation identified in 1 VSCN2 family | ?Visceral neuropathy, familial, 2, autosomal recessive | 619465 | Autosomal recessive | 3 | Erbb2 |
| 17q12 | 17:39688094-39728658 | ERBB2, NGL, NEU, HER2, VSCN2 | Avian erythroblastic leukemia viral (v-erb-b2) oncogene homolog 2 (neuro/glioblastoma derived oncogene homolog) | 164870 | ERBB2 | 2064 | ENSG00000141736,ENST00000269571;;;ENST00000269571 | mutation identified in 1 VSCN2 family | Adenocarcinoma of lung, somatic | 211980 |  | 3 | Erbb2 |
| 17q12 | 17:39688094-39728658 | ERBB2, NGL, NEU, HER2, VSCN2 | Avian erythroblastic leukemia viral (v-erb-b2) oncogene homolog 2 (neuro/glioblastoma derived oncogene homolog) | 164870 | ERBB2 | 2064 | ENSG00000141736,ENST00000269571;;;ENST00000269571 | mutation identified in 1 VSCN2 family | Gastric cancer, somatic | 613659 |  | 3 | Erbb2 |
| 17q12 | 17:39688094-39728658 | ERBB2, NGL, NEU, HER2, VSCN2 | Avian erythroblastic leukemia viral (v-erb-b2) oncogene homolog 2 (neuro/glioblastoma derived oncogene homolog) | 164870 | ERBB2 | 2064 | ENSG00000141736,ENST00000269571;;;ENST00000269571 | mutation identified in 1 VSCN2 family | Glioblastoma, somatic | 137800 |  | 3 | Erbb2 |
| 17q12 | 17:39688094-39728658 | ERBB2, NGL, NEU, HER2, VSCN2 | Avian erythroblastic leukemia viral (v-erb-b2) oncogene homolog 2 (neuro/glioblastoma derived oncogene homolog) | 164870 | ERBB2 | 2064 | ENSG00000141736,ENST00000269571;;;ENST00000269571 | mutation identified in 1 VSCN2 family | Ovarian cancer, somatic | 167000 |  | 3 | Erbb2 |
| 17q21.2 | 17:40553769-40565472 | CCR7, CMKBR7, EBI1 | Chemokine (C-C) receptor 7 (Epstein-Barr virus induced gene 1) | 600242 | CCR7 | 1236 | ENSG00000126353,ENST00000246657;;;ENST00000246657 | | | | | Ccr7 |  |
| 17q21.31 | 17:43766125-43778977 | DUSP3, VHR | Dual specificity phosphatase-3 (vaccinia virus phosphatase VH1-related) | 600183 | DUSP3 | 1845 | ENSG00000108861,ENST00000226004;;;ENST00000226004 | | | | | Dusp3 |  |
| 18q21 | 18:45900001-63900000 | SSAV1 | Simian sarcoma-associated virus-1/Gibbon ape leukemia virus | 182090 |  | 6739 |  |  |  |  |  |  |  |
| 19pter-q13 | 19:1-58617616 | CXB3S | Coxsackie virus B3 sensitivity | 120050 |  | 1526 |  |  |  |  |  |  |  |
| 19p13.3 | 19:4229523-4237528 | EBI3 | Epstein-Barr virus-induced gene 3 | 605816 | EBI3 | 10148 | ENSG00000105246,ENST00000221847;;;ENST00000221847 | | | | | Ebi3 |  |
| 19q13.2 | 19:39243455-39245250 | IFNL3, IL28B | Interferon, lambda 3 | 607402 | IFNL3 | 282617 | ENSG00000197110,ENST00000413851;;;ENST00000413851 | | {Hepatitis C virus infection, response to therapy of} | 609532 |  | 3 | Ifnl2,Ifnl3 |
| 19q13.31 | 19:44643910-44666162 | PVR, PVS | Polio virus receptor | 173850 | PVR | 5817 | ENSG00000073008,ENST00000425690;;;ENST00000425690 | | | | | Pvr |  |
| 19q13.32 | 19:45001464-45038192 | RELB, IREL, IMD53 | v-rel avian reticuloendotheliosis viral oncogene homolog B | 604758 | RELB | 5971 | ENSG00000104856,ENST00000221452;;;ENST00000221452 | mutation identified in 1 IMD53 family | ?Immunodeficiency 53 | 617585 | Autosomal recessive | 3 | Relb |
| 19q13.33 | 19:48695971-48705951 | FUT2, SE, B12QTL1 | Fucosyltransferase-2 (secretor) | 182100 | FUT2 | 2524 | ENSG00000176920,ENST00000425340;;;ENST00000425340 | H, SE = alpha-L-fucosyltransferases; from common ancestral genes; tightly linked to FUT1 | [Bombay phenotype, digenic] | 616754 | Autosomal recessive | 3 | Fut2,Sec1 |
| 19q13.33 | 19:48695971-48705951 | FUT2, SE, B12QTL1 | Fucosyltransferase-2 (secretor) | 182100 | FUT2 | 2524 | ENSG00000176920,ENST00000425340;;;ENST00000425340 | H, SE = alpha-L-fucosyltransferases; from common ancestral genes; tightly linked to FUT1 | {Norwalk virus infection, resistance to} | | | 3 | Fut2,Sec1 |
| 19q13.33 | 19:48695971-48705951 | FUT2, SE, B12QTL1 | Fucosyltransferase-2 (secretor) | 182100 | FUT2 | 2524 | ENSG00000176920,ENST00000425340;;;ENST00000425340 | H, SE = alpha-L-fucosyltransferases; from common ancestral genes; tightly linked to FUT1 | {Vitamin B12 plasma level QTL1} | 612542 |  | 3 | Fut2,Sec1 |
| 19q13.4-qter | 19:50900001-58617616 | AAVS1 | Adeno-associated virus integration site-1 | 102699 | AAVS1 | 17 |  |  |  |  |  |  |  |
| 20p11.23 | 20:18467390-18484646 | POLR3F, RPC39, RPC6, IMD101 | Polymerase III, RNA, subunit F | 617455 | POLR3F | 10621 | ENSG00000132664,ENST00000377603;;;ENST00000377603 | mutation identified in 1 IMD101 family | ?Immunodeficiency 101 (varicella zoster virus-specific) | 619872 | Autosomal dominant | 3 | Polr3f |
| 20q13.12 | 20:43667114-43716482 | MYBL2, BMYB | v-myb avian myeloblastosis viral oncogene homolog-like 2 | 601415 | MYBL2 | 4605 | ENSG00000101057,ENST00000217026;;;ENST00000217026 | | | | | Mybl2 |  |
| 21q21.1 | 21:17513043-17636262 | CXADR, CAR | Coxsackie virus and adenovirus receptor | 602621 | CXADR | 1525 | ENSG00000154639,ENST00000284878;;;ENST00000284878 | pseudogenes on chr.15 and chr.18 | | | | Cxadr |  |
| 21q22.11 | 21:33229938-33265664 | IFNAR2, IMD45 | Interferon, alpha, beta, and omega, receptor 2 | 602376 | IFNAR2 | 3455 | ENSG00000159110,ENST00000342136;;;ENST00000342136 | | Immunodeficiency 45 | 616669 | Autosomal recessive | 3 | Ifnar2 |
| 21q22.11 | 21:33229938-33265664 | IFNAR2, IMD45 | Interferon, alpha, beta, and omega, receptor 2 | 602376 | IFNAR2 | 3455 | ENSG00000159110,ENST00000342136;;;ENST00000342136 | | {Hepatitis B virus, susceptibility to} | 610424 |  | 3 | Ifnar2 |
| 21q22.11 | 21:33266367-33297221 | IL10RB, CRFB4, IBD25 | Interleukin 10 receptor, beta | 123889 | IL10RB | 3588 | ENSG00000243646,ENST00000290200;;;ENST00000290200 | 35kb distal to IFNAR; D21S58 | Inflammatory bowel disease 25, early onset, autosomal recessive | 612567 | Autosomal recessive | 3 | Il10rb |
| 21q22.11 | 21:33266367-33297221 | IL10RB, CRFB4, IBD25 | Interleukin 10 receptor, beta | 123889 | IL10RB | 3588 | ENSG00000243646,ENST00000290200;;;ENST00000290200 | 35kb distal to IFNAR; D21S58 | {Hepatitis B virus, susceptibility to} | 610424 |  | 3 | Il10rb |
| 21q22.11 | 21:33324395-33359864 | IFNAR1, IMD106 | Interferon, alpha, beta and omega, receptor 1 | 107450 | IFNAR1 | 3454 | ENSG00000142166,ENST00000270139;;;ENST00000270139 | | Immunodeficiency 106, susceptibility to viral infections | 619935 | Autosomal recessive | 3 | Ifnar1 |
| 22q11.21 | 22:20917407-20953747 | CRKL | v-crk avian sarcoma virus CT10 oncogene homolog-like | 602007 | CRKL | 1399 | ENSG00000099942,ENST00000354336;;;ENST00000354336 | | | | | Crkl |  |
| Xp11.23 | X:48826513-48829869 | HRAS2, HRASP | v-Ha-ras harvey rat sarcoma viral oncogene homolog 2 | 300437 | ERAS | 3266 | ENSG00000187682,ENST00000636362;;;ENST00000636362 | | | | | Eras |  |
| Xq21.1 | X:77825747-77895568 | MAGT1, IAP, XMEN, CDG1CC | Magnesium transporter 1 | 300715 | MAGT1 | 84061 | ENSG00000102158,ENST00000618282;;;ENST00000618282 | | Congenital disorder of glycosylation, type Icc | 301031 | X-linked recessive | 3 | Magt1 |
| Xq21.1 | X:77825747-77895568 | MAGT1, IAP, XMEN, CDG1CC | Magnesium transporter 1 | 300715 | MAGT1 | 84061 | ENSG00000102158,ENST00000618282;;;ENST00000618282 | | Immunodeficiency, X-linked, with magnesium defect, Epstein-Barr virus infection and neoplasia | 300853 | X-linked recessive | 3 | Magt1 |

**Table S3.** Results of BP GO terms enrichment of XBCQD in monkeypox virus.

| **ID** | **Description** | **GeneRatio** | **BgRatio** | **pvalue** | **p.adjust** | **qvalue** | **geneID** | **Count** |
| --- | --- | --- | --- | --- | --- | --- | --- | --- |
| GO:0048511 | rhythmic process | 10/36 | 305/18866 | 1.84518E-10 | 1.65432E-07 | 8.02245E-08 | ESR1/NCOA2/JUN/F7/TP53/PCNA/CASP3/SLC6A4/PGR/OPRM1 | 10 |
| GO:0048145 | regulation of fibroblast proliferation | 7/36 | 83/18866 | 1.85561E-10 | 1.65432E-07 | 8.02245E-08 | ESR1/JUN/TP53/MYC/CDKN1A/CCNB1/BAX | 7 |
| GO:0048144 | fibroblast proliferation | 7/36 | 84/18866 | 2.02157E-10 | 1.65432E-07 | 8.02245E-08 | ESR1/JUN/TP53/MYC/CDKN1A/CCNB1/BAX | 7 |
| GO:0006352 | DNA-templated transcription, initiation | 9/36 | 249/18866 | 7.2579E-10 | 4.45453E-07 | 2.16018E-07 | ESR2/ESR1/JUN/AR/TP53/CDKN1A/CCNB1/BAX/PGR | 9 |
| GO:0006367 | transcription initiation from RNA polymerase II promoter | 8/36 | 189/18866 | 2.08191E-09 | 8.32258E-07 | 4.03595E-07 | ESR2/ESR1/AR/TP53/CDKN1A/CCNB1/BAX/PGR | 8 |
| GO:0045787 | positive regulation of cell cycle | 10/36 | 396/18866 | 2.31621E-09 | 8.32258E-07 | 4.03595E-07 | CHEK1/TP53/PRKCE/PRKCA/PCNA/IL1B/CDKN1A/CCNB1/BAX/SLC6A4 | 10 |
| GO:0042493 | response to drug | 10/36 | 397/18866 | 2.37304E-09 | 8.32258E-07 | 4.03595E-07 | F7/TP53/MYC/IL1B/CDKN1A/CCNB1/CASP3/SLC6A4/PDE3A/KCNH2 | 10 |
| GO:0072401 | signal transduction involved in DNA integrity checkpoint | 6/36 | 73/18866 | 4.83566E-09 | 1.29014E-06 | 6.2564E-07 | CHEK1/TP53/PCNA/CDKN1A/CCNB1/BAX | 6 |
| GO:0072422 | signal transduction involved in DNA damage checkpoint | 6/36 | 73/18866 | 4.83566E-09 | 1.29014E-06 | 6.2564E-07 | CHEK1/TP53/PCNA/CDKN1A/CCNB1/BAX | 6 |
| GO:0072395 | signal transduction involved in cell cycle checkpoint | 6/36 | 74/18866 | 5.25515E-09 | 1.29014E-06 | 6.2564E-07 | CHEK1/TP53/PCNA/CDKN1A/CCNB1/BAX | 6 |
| GO:0009411 | response to UV | 7/36 | 140/18866 | 7.43206E-09 | 1.6587E-06 | 8.04369E-07 | CHEK1/TP53/PCNA/MYC/CDKN1A/CASP3/BAX | 7 |
| GO:0050673 | epithelial cell proliferation | 10/36 | 453/18866 | 8.38632E-09 | 1.7157E-06 | 8.32012E-07 | ESR1/JUN/AR/SCN5A/KDR/TNF/PRKCA/MYC/BAX/PGR | 10 |
| GO:0034644 | cellular response to UV | 6/36 | 81/18866 | 9.11886E-09 | 1.72206E-06 | 8.35096E-07 | CHEK1/TP53/PCNA/MYC/CDKN1A/BAX | 6 |
| GO:0016572 | histone phosphorylation | 5/36 | 40/18866 | 1.18779E-08 | 2.08288E-06 | 1.01007E-06 | CHEK1/PRKCD/PRKCA/IL1B/CCNB1 | 5 |
| GO:0048545 | response to steroid hormone | 9/36 | 346/18866 | 1.28762E-08 | 2.10741E-06 | 1.02196E-06 | ESR2/ESR1/NCOA2/AR/TNF/PCNA/CDKN1A/CASP3/PGR | 9 |
| GO:0034349 | glial cell apoptotic process | 4/36 | 15/18866 | 1.50116E-08 | 2.30334E-06 | 1.11698E-06 | TP53/PRKCD/PRKCA/CASP3 | 4 |
| GO:0050678 | regulation of epithelial cell proliferation | 9/36 | 395/18866 | 4.03073E-08 | 5.69682E-06 | 2.76261E-06 | JUN/AR/SCN5A/KDR/TNF/PRKCA/MYC/BAX/PGR | 9 |
| GO:0048146 | positive regulation of fibroblast proliferation | 5/36 | 51/18866 | 4.17689E-08 | 5.69682E-06 | 2.76261E-06 | ESR1/JUN/MYC/CDKN1A/CCNB1 | 5 |
| GO:0006977 | DNA damage response, signal transduction by p53 class mediator resulting in cell cycle arrest | 5/36 | 56/18866 | 6.74566E-08 | 8.63243E-06 | 4.18621E-06 | TP53/PCNA/CDKN1A/CCNB1/BAX | 5 |
| GO:0072431 | signal transduction involved in mitotic G1 DNA damage checkpoint | 5/36 | 57/18866 | 7.38416E-08 | 8.63243E-06 | 4.18621E-06 | TP53/PCNA/CDKN1A/CCNB1/BAX | 5 |
| GO:1902400 | intracellular signal transduction involved in G1 DNA damage checkpoint | 5/36 | 57/18866 | 7.38416E-08 | 8.63243E-06 | 4.18621E-06 | TP53/PCNA/CDKN1A/CCNB1/BAX | 5 |
| GO:0043525 | positive regulation of neuron apoptotic process | 5/36 | 59/18866 | 8.80482E-08 | 8.64633E-06 | 4.19295E-06 | JUN/TP53/TNF/CASP3/BAX | 5 |
| GO:0072413 | signal transduction involved in mitotic cell cycle checkpoint | 5/36 | 59/18866 | 8.80482E-08 | 8.64633E-06 | 4.19295E-06 | TP53/PCNA/CDKN1A/CCNB1/BAX | 5 |
| GO:1902402 | signal transduction involved in mitotic DNA damage checkpoint | 5/36 | 59/18866 | 8.80482E-08 | 8.64633E-06 | 4.19295E-06 | TP53/PCNA/CDKN1A/CCNB1/BAX | 5 |
| GO:1902403 | signal transduction involved in mitotic DNA integrity checkpoint | 5/36 | 59/18866 | 8.80482E-08 | 8.64633E-06 | 4.19295E-06 | TP53/PCNA/CDKN1A/CCNB1/BAX | 5 |
| GO:0001933 | negative regulation of protein phosphorylation | 9/36 | 444/18866 | 1.09409E-07 | 1.03307E-05 | 5.00977E-06 | JUN/PRKCD/PKIA/MYC/IL1B/CDKN1A/CCNB1/CASP3/BAX | 9 |
| GO:0031571 | mitotic G1 DNA damage checkpoint | 5/36 | 63/18866 | 1.22941E-07 | 1.11786E-05 | 5.42093E-06 | TP53/PCNA/CDKN1A/CCNB1/BAX | 5 |
| GO:0044783 | G1 DNA damage checkpoint | 5/36 | 64/18866 | 1.33177E-07 | 1.12742E-05 | 5.46729E-06 | TP53/PCNA/CDKN1A/CCNB1/BAX | 5 |
| GO:0044819 | mitotic G1/S transition checkpoint | 5/36 | 64/18866 | 1.33177E-07 | 1.12742E-05 | 5.46729E-06 | TP53/PCNA/CDKN1A/CCNB1/BAX | 5 |
| GO:0071482 | cellular response to light stimulus | 6/36 | 129/18866 | 1.49677E-07 | 1.22486E-05 | 5.93981E-06 | CHEK1/TP53/PCNA/MYC/CDKN1A/BAX | 6 |
| GO:0071214 | cellular response to abiotic stimulus | 8/36 | 331/18866 | 1.62744E-07 | 1.24855E-05 | 6.05471E-06 | CHEK1/TP53/PCNA/MYC/IL1B/CDKN1A/CASP3/BAX | 8 |
| GO:0104004 | cellular response to environmental stimulus | 8/36 | 331/18866 | 1.62744E-07 | 1.24855E-05 | 6.05471E-06 | CHEK1/TP53/PCNA/MYC/IL1B/CDKN1A/CASP3/BAX | 8 |
| GO:0002064 | epithelial cell development | 7/36 | 221/18866 | 1.72142E-07 | 1.28063E-05 | 6.21027E-06 | ESR1/AR/TNF/IL1B/FASN/CDKN1A/PGR | 7 |
| GO:0042770 | signal transduction in response to DNA damage | 6/36 | 133/18866 | 1.79438E-07 | 1.29565E-05 | 6.2831E-06 | CHEK1/TP53/PCNA/CDKN1A/CCNB1/BAX | 6 |
| GO:0042698 | ovulation cycle | 5/36 | 69/18866 | 1.94962E-07 | 1.36752E-05 | 6.63165E-06 | ESR1/PCNA/CASP3/PGR/OPRM1 | 5 |
| GO:0042326 | negative regulation of phosphorylation | 9/36 | 484/18866 | 2.27198E-07 | 1.54936E-05 | 7.51348E-06 | JUN/PRKCD/PKIA/MYC/IL1B/CDKN1A/CCNB1/CASP3/BAX | 9 |
| GO:0032355 | response to estradiol | 6/36 | 141/18866 | 2.53643E-07 | 1.68296E-05 | 8.16133E-06 | ESR2/ESR1/F7/PCNA/CASP3/SLC6A4 | 6 |
| GO:0000077 | DNA damage checkpoint | 6/36 | 151/18866 | 3.80163E-07 | 2.45605E-05 | 1.19104E-05 | CHEK1/TP53/PCNA/CDKN1A/CCNB1/BAX | 6 |
| GO:0071158 | positive regulation of cell cycle arrest | 5/36 | 81/18866 | 4.37222E-07 | 2.72179E-05 | 1.3199E-05 | TP53/PCNA/CDKN1A/CCNB1/BAX | 5 |
| GO:0048754 | branching morphogenesis of an epithelial tube | 6/36 | 155/18866 | 4.43469E-07 | 2.72179E-05 | 1.3199E-05 | ESR1/AR/KDR/TNF/MYC/PGR | 6 |
| GO:0031570 | DNA integrity checkpoint | 6/36 | 161/18866 | 5.54456E-07 | 3.31997E-05 | 1.60999E-05 | CHEK1/TP53/PCNA/CDKN1A/CCNB1/BAX | 6 |
| GO:0070482 | response to oxygen levels | 8/36 | 396/18866 | 6.35141E-07 | 3.71255E-05 | 1.80036E-05 | F7/TP53/PRKCE/MYC/CDKN1A/CCNB1/CASP3/SLC6A4 | 8 |
| GO:1901216 | positive regulation of neuron death | 5/36 | 97/18866 | 1.07588E-06 | 6.14252E-05 | 2.97875E-05 | JUN/TP53/TNF/CASP3/BAX | 5 |
| GO:0044773 | mitotic DNA damage checkpoint | 5/36 | 101/18866 | 1.3151E-06 | 7.2627E-05 | 3.52197E-05 | TP53/PCNA/CDKN1A/CCNB1/BAX | 5 |
| GO:0061138 | morphogenesis of a branching epithelium | 6/36 | 187/18866 | 1.33125E-06 | 7.2627E-05 | 3.52197E-05 | ESR1/AR/KDR/TNF/MYC/PGR | 6 |
| GO:0071478 | cellular response to radiation | 6/36 | 188/18866 | 1.37326E-06 | 7.32901E-05 | 3.55412E-05 | CHEK1/TP53/PCNA/MYC/CDKN1A/BAX | 6 |
| GO:0090068 | positive regulation of cell cycle process | 7/36 | 302/18866 | 1.40859E-06 | 7.35763E-05 | 3.568E-05 | TP53/PRKCE/PCNA/IL1B/CDKN1A/CCNB1/BAX | 7 |
| GO:0042391 | regulation of membrane potential | 8/36 | 443/18866 | 1.47415E-06 | 7.38578E-05 | 3.58166E-05 | JUN/SCN5A/KDR/BAX/OPRM1/KCNH2/GABRA1/CHRNA2 | 8 |
| GO:0048732 | gland development | 8/36 | 443/18866 | 1.47415E-06 | 7.38578E-05 | 3.58166E-05 | ESR1/JUN/AR/TNF/PCNA/FASN/BAX/PGR | 8 |
| GO:0060443 | mammary gland morphogenesis | 4/36 | 45/18866 | 1.57317E-06 | 7.58886E-05 | 3.68014E-05 | ESR1/AR/BAX/PGR | 4 |
| GO:0009314 | response to radiation | 8/36 | 447/18866 | 1.5765E-06 | 7.58886E-05 | 3.68014E-05 | CHEK1/TP53/PCNA/MYC/CDKN1A/CASP3/BAX/OPRM1 | 8 |
| GO:0071156 | regulation of cell cycle arrest | 5/36 | 106/18866 | 1.67106E-06 | 7.74047E-05 | 3.75366E-05 | TP53/PCNA/CDKN1A/CCNB1/BAX | 5 |
| GO:2000379 | positive regulation of reactive oxygen species metabolic process | 5/36 | 106/18866 | 1.67106E-06 | 7.74047E-05 | 3.75366E-05 | TP53/TNF/PRKCD/IL1B/CDKN1A | 5 |
| GO:0030330 | DNA damage response, signal transduction by p53 class mediator | 5/36 | 107/18866 | 1.75057E-06 | 7.95862E-05 | 3.85945E-05 | TP53/PCNA/CDKN1A/CCNB1/BAX | 5 |
| GO:0044774 | mitotic DNA integrity checkpoint | 5/36 | 108/18866 | 1.83304E-06 | 8.182E-05 | 3.96778E-05 | TP53/PCNA/CDKN1A/CCNB1/BAX | 5 |
| GO:1901654 | response to ketone | 6/36 | 200/18866 | 1.96775E-06 | 8.43503E-05 | 4.09048E-05 | NCOA2/AR/F7/PRKCE/PCNA/CDKN1A | 6 |
| GO:2000377 | regulation of reactive oxygen species metabolic process | 6/36 | 200/18866 | 1.96775E-06 | 8.43503E-05 | 4.09048E-05 | TP53/TNF/PRKCD/IL1B/EIF6/CDKN1A | 6 |
| GO:0001763 | morphogenesis of a branching structure | 6/36 | 201/18866 | 2.02552E-06 | 8.43503E-05 | 4.09048E-05 | ESR1/AR/KDR/TNF/MYC/PGR | 6 |
| GO:0009416 | response to light stimulus | 7/36 | 319/18866 | 2.02716E-06 | 8.43503E-05 | 4.09048E-05 | CHEK1/TP53/PCNA/MYC/CDKN1A/CASP3/BAX | 7 |
| GO:0090399 | replicative senescence | 3/36 | 14/18866 | 2.28934E-06 | 9.08228E-05 | 4.40436E-05 | CHEK1/TP53/CDKN1A | 3 |
| GO:1903799 | negative regulation of production of miRNAs involved in gene silencing by miRNA | 3/36 | 14/18866 | 2.28934E-06 | 9.08228E-05 | 4.40436E-05 | ESR1/TP53/TNF | 3 |
| GO:0070371 | ERK1 and ERK2 cascade | 7/36 | 325/18866 | 2.29369E-06 | 9.08228E-05 | 4.40436E-05 | JUN/KDR/TNF/PRKCA/MYC/IL1B/OPRM1 | 7 |
| GO:0060562 | epithelial tube morphogenesis | 7/36 | 331/18866 | 2.58897E-06 | 0.000100888 | 4.89244E-05 | ESR1/AR/KDR/TNF/MYC/CASP3/PGR | 7 |
| GO:0050679 | positive regulation of epithelial cell proliferation | 6/36 | 211/18866 | 2.68333E-06 | 0.000102931 | 4.99153E-05 | JUN/AR/SCN5A/KDR/PRKCA/MYC | 6 |
| GO:0045930 | negative regulation of mitotic cell cycle | 7/36 | 341/18866 | 3.15163E-06 | 0.000119035 | 5.77246E-05 | CHEK1/TP53/TNF/PCNA/CDKN1A/CCNB1/BAX | 7 |
| GO:0000075 | cell cycle checkpoint | 6/36 | 219/18866 | 3.32686E-06 | 0.000123749 | 6.00109E-05 | CHEK1/TP53/PCNA/CDKN1A/CCNB1/BAX | 6 |
| GO:0022612 | gland morphogenesis | 5/36 | 124/18866 | 3.62208E-06 | 0.00013272 | 6.4361E-05 | ESR1/AR/TNF/BAX/PGR | 5 |
| GO:0010332 | response to gamma radiation | 4/36 | 56/18866 | 3.82059E-06 | 0.000137934 | 6.68898E-05 | TP53/MYC/CDKN1A/BAX | 4 |
| GO:2000134 | negative regulation of G1/S transition of mitotic cell cycle | 5/36 | 126/18866 | 3.91815E-06 | 0.000139407 | 6.76038E-05 | TP53/PCNA/CDKN1A/CCNB1/BAX | 5 |
| GO:0001666 | response to hypoxia | 7/36 | 359/18866 | 4.42241E-06 | 0.0001551 | 7.52143E-05 | F7/TP53/PRKCE/MYC/CCNB1/CASP3/SLC6A4 | 7 |
| GO:0031663 | lipopolysaccharide-mediated signaling pathway | 4/36 | 59/18866 | 4.71503E-06 | 0.000163034 | 7.90615E-05 | TNF/PRKCE/PRKCA/IL1B | 4 |
| GO:0007050 | cell cycle arrest | 6/36 | 234/18866 | 4.87172E-06 | 0.000163588 | 7.93303E-05 | TP53/PCNA/MYC/CDKN1A/CCNB1/BAX | 6 |
| GO:1902807 | negative regulation of cell cycle G1/S phase transition | 5/36 | 132/18866 | 4.92185E-06 | 0.000163588 | 7.93303E-05 | TP53/PCNA/CDKN1A/CCNB1/BAX | 5 |
| GO:0051052 | regulation of DNA metabolic process | 7/36 | 365/18866 | 4.93096E-06 | 0.000163588 | 7.93303E-05 | CHEK1/TP53/PRKCD/PCNA/MYC/CDKN1A/BAX | 7 |
| GO:0050994 | regulation of lipid catabolic process | 4/36 | 60/18866 | 5.04496E-06 | 0.000165138 | 8.00822E-05 | TNF/PRKCE/PRKCD/IL1B | 4 |
| GO:0036293 | response to decreased oxygen levels | 7/36 | 371/18866 | 5.48742E-06 | 0.000177258 | 8.59595E-05 | F7/TP53/PRKCE/MYC/CCNB1/CASP3/SLC6A4 | 7 |
| GO:0007213 | G protein-coupled acetylcholine receptor signaling pathway | 3/36 | 19/18866 | 6.05456E-06 | 0.000193038 | 9.36119E-05 | OPRM1/CHRM4/CHRM3 | 3 |
| GO:0030879 | mammary gland development | 5/36 | 142/18866 | 7.03255E-06 | 0.000218414 | 0.000105918 | ESR1/AR/FASN/BAX/PGR | 5 |
| GO:0060965 | negative regulation of gene silencing by miRNA | 3/36 | 20/18866 | 7.11367E-06 | 0.000218414 | 0.000105918 | ESR1/TP53/TNF | 3 |
| GO:0009636 | response to toxic substance | 6/36 | 250/18866 | 7.11736E-06 | 0.000218414 | 0.000105918 | TNF/CDKN1A/CCNB1/BAX/SLC6A4/PON1 | 6 |
| GO:0010212 | response to ionizing radiation | 5/36 | 144/18866 | 7.52881E-06 | 0.000228188 | 0.000110657 | TP53/MYC/CDKN1A/CASP3/BAX | 5 |
| GO:0071901 | negative regulation of protein serine/threonine kinase activity | 5/36 | 148/18866 | 8.60344E-06 | 0.000257579 | 0.00012491 | PRKCD/PKIA/IL1B/CDKN1A/CASP3 | 5 |
| GO:0061041 | regulation of wound healing | 5/36 | 151/18866 | 9.48541E-06 | 0.000280562 | 0.000136056 | F7/TNF/PRKCE/PRKCD/PRKCA | 5 |
| GO:0035690 | cellular response to drug | 4/36 | 71/18866 | 9.90338E-06 | 0.000286033 | 0.000138709 | TP53/MYC/IL1B/KCNH2 | 4 |
| GO:0061180 | mammary gland epithelium development | 4/36 | 71/18866 | 9.90338E-06 | 0.000286033 | 0.000138709 | ESR1/AR/BAX/PGR | 4 |
| GO:0072331 | signal transduction by p53 class mediator | 6/36 | 267/18866 | 1.03589E-05 | 0.000295711 | 0.000143402 | CHEK1/TP53/PCNA/CDKN1A/CCNB1/BAX | 6 |
| GO:0060149 | negative regulation of posttranscriptional gene silencing | 3/36 | 23/18866 | 1.10077E-05 | 0.000301112 | 0.000146021 | ESR1/TP53/TNF | 3 |
| GO:0060967 | negative regulation of gene silencing by RNA | 3/36 | 23/18866 | 1.10077E-05 | 0.000301112 | 0.000146021 | ESR1/TP53/TNF | 3 |
| GO:1903798 | regulation of production of miRNAs involved in gene silencing by miRNA | 3/36 | 23/18866 | 1.10077E-05 | 0.000301112 | 0.000146021 | ESR1/TP53/TNF | 3 |
| GO:1901988 | negative regulation of cell cycle phase transition | 6/36 | 270/18866 | 1.10387E-05 | 0.000301112 | 0.000146021 | CHEK1/TP53/PCNA/CDKN1A/CCNB1/BAX | 6 |
| GO:0038034 | signal transduction in absence of ligand | 4/36 | 75/18866 | 1.23214E-05 | 0.000328794 | 0.000159445 | TNF/IL1B/CASP3/BAX | 4 |
| GO:0097192 | extrinsic apoptotic signaling pathway in absence of ligand | 4/36 | 75/18866 | 1.23214E-05 | 0.000328794 | 0.000159445 | TNF/IL1B/CASP3/BAX | 4 |
| GO:0060444 | branching involved in mammary gland duct morphogenesis | 3/36 | 24/18866 | 1.25638E-05 | 0.000331657 | 0.000160834 | ESR1/AR/PGR | 3 |
| GO:0070920 | regulation of production of small RNA involved in gene silencing by RNA | 3/36 | 25/18866 | 1.42583E-05 | 0.000368421 | 0.000178662 | ESR1/TP53/TNF | 3 |
| GO:0046651 | lymphocyte proliferation | 6/36 | 283/18866 | 1.44159E-05 | 0.000368421 | 0.000178662 | TP53/PRKCD/IL1B/CDKN1A/CASP3/BAX | 6 |
| GO:0019216 | regulation of lipid metabolic process | 7/36 | 431/18866 | 1.45589E-05 | 0.000368421 | 0.000178662 | NCOA2/TNF/PRKCE/PRKCD/IL1B/FASN/EIF6 | 7 |
| GO:0030099 | myeloid cell differentiation | 7/36 | 431/18866 | 1.45589E-05 | 0.000368421 | 0.000178662 | JUN/TNF/PRKCA/MYC/FASN/EIF6/CASP3 | 7 |
| GO:0043270 | positive regulation of ion transport | 6/36 | 284/18866 | 1.47068E-05 | 0.000368421 | 0.000178662 | SCN5A/PRKCD/IL1B/BAX/SLC6A4/KCNH2 | 6 |
| GO:0007093 | mitotic cell cycle checkpoint | 5/36 | 166/18866 | 1.50115E-05 | 0.000372256 | 0.000180522 | TP53/PCNA/CDKN1A/CCNB1/BAX | 5 |
| GO:0032943 | mononuclear cell proliferation | 6/36 | 286/18866 | 1.5303E-05 | 0.000375689 | 0.000182186 | TP53/PRKCD/IL1B/CDKN1A/CASP3/BAX | 6 |
| GO:0000082 | G1/S transition of mitotic cell cycle | 6/36 | 287/18866 | 1.56084E-05 | 0.000379391 | 0.000183982 | TP53/PCNA/MYC/CDKN1A/CCNB1/BAX | 6 |
| GO:0072593 | reactive oxygen species metabolic process | 6/36 | 288/18866 | 1.59186E-05 | 0.000383139 | 0.000185799 | TP53/TNF/PRKCD/IL1B/EIF6/CDKN1A | 6 |
| GO:0051100 | negative regulation of binding | 5/36 | 169/18866 | 1.63683E-05 | 0.000390138 | 0.000189193 | JUN/PRKCD/CDKN1A/BAX/MAP2 | 5 |
| GO:0097193 | intrinsic apoptotic signaling pathway | 6/36 | 290/18866 | 1.65541E-05 | 0.000390773 | 0.000189501 | TP53/TNF/PRKCD/CDKN1A/CASP3/BAX | 6 |
| GO:0009651 | response to salt stress | 3/36 | 27/18866 | 1.80854E-05 | 0.000418864 | 0.000203124 | TP53/TNF/BAX | 3 |
| GO:0050996 | positive regulation of lipid catabolic process | 3/36 | 27/18866 | 1.80854E-05 | 0.000418864 | 0.000203124 | PRKCE/PRKCD/IL1B | 3 |
| GO:0051348 | negative regulation of transferase activity | 6/36 | 296/18866 | 1.85848E-05 | 0.000426408 | 0.000206782 | TP53/PRKCD/PKIA/IL1B/CDKN1A/CASP3 | 6 |
| GO:0043254 | regulation of protein-containing complex assembly | 7/36 | 449/18866 | 1.89554E-05 | 0.000430885 | 0.000208953 | ESR1/TP53/TNF/PRKCE/PRKCD/BAX/MAP2 | 7 |
| GO:0095500 | acetylcholine receptor signaling pathway | 3/36 | 28/18866 | 2.02291E-05 | 0.000451927 | 0.000219157 | OPRM1/CHRM4/CHRM3 | 3 |
| GO:0006970 | response to osmotic stress | 4/36 | 85/18866 | 2.02493E-05 | 0.000451927 | 0.000219157 | TP53/TNF/CASP3/BAX | 4 |
| GO:0062012 | regulation of small molecule metabolic process | 7/36 | 456/18866 | 2.09382E-05 | 0.000463094 | 0.000224572 | NCOA2/TP53/TNF/PRKCE/IL1B/FASN/EIF6 | 7 |
| GO:0070372 | regulation of ERK1 and ERK2 cascade | 6/36 | 306/18866 | 2.24135E-05 | 0.000491296 | 0.000238249 | JUN/KDR/TNF/PRKCA/IL1B/OPRM1 | 6 |
| GO:1903034 | regulation of response to wounding | 5/36 | 183/18866 | 2.40152E-05 | 0.000514756 | 0.000249625 | F7/TNF/PRKCE/PRKCD/PRKCA | 5 |
| GO:0018105 | peptidyl-serine phosphorylation | 6/36 | 310/18866 | 2.41128E-05 | 0.000514756 | 0.000249625 | TNF/PRKCE/PRKCD/PRKCA/CCNB1/BAX | 6 |
| GO:0044843 | cell cycle G1/S phase transition | 6/36 | 310/18866 | 2.41128E-05 | 0.000514756 | 0.000249625 | TP53/PCNA/MYC/CDKN1A/CCNB1/BAX | 6 |
| GO:0048147 | negative regulation of fibroblast proliferation | 3/36 | 30/18866 | 2.50045E-05 | 0.000520221 | 0.000252276 | TP53/MYC/BAX | 3 |
| GO:1905144 | response to acetylcholine | 3/36 | 30/18866 | 2.50045E-05 | 0.000520221 | 0.000252276 | OPRM1/CHRM4/CHRM3 | 3 |
| GO:1905145 | cellular response to acetylcholine | 3/36 | 30/18866 | 2.50045E-05 | 0.000520221 | 0.000252276 | OPRM1/CHRM4/CHRM3 | 3 |
| GO:2000045 | regulation of G1/S transition of mitotic cell cycle | 5/36 | 185/18866 | 2.53022E-05 | 0.000520752 | 0.000252533 | TP53/PCNA/CDKN1A/CCNB1/BAX | 5 |
| GO:0070661 | leukocyte proliferation | 6/36 | 313/18866 | 2.54543E-05 | 0.000520752 | 0.000252533 | TP53/PRKCD/IL1B/CDKN1A/CASP3/BAX | 6 |
| GO:0031667 | response to nutrient levels | 7/36 | 473/18866 | 2.64782E-05 | 0.000533498 | 0.000258714 | JUN/F7/TP53/CDKN1A/SLC6A4/PON1/OPRM1 | 7 |
| GO:0051899 | membrane depolarization | 4/36 | 91/18866 | 2.65119E-05 | 0.000533498 | 0.000258714 | JUN/SCN5A/KDR/KCNH2 | 4 |
| GO:0010165 | response to X-ray | 3/36 | 32/18866 | 3.04674E-05 | 0.000603205 | 0.000292518 | TP53/CDKN1A/CASP3 | 3 |
| GO:0060603 | mammary gland duct morphogenesis | 3/36 | 32/18866 | 3.04674E-05 | 0.000603205 | 0.000292518 | ESR1/AR/PGR | 3 |
| GO:1901987 | regulation of cell cycle phase transition | 7/36 | 486/18866 | 3.14881E-05 | 0.000618427 | 0.0002999 | CHEK1/TP53/PKIA/PCNA/CDKN1A/CCNB1/BAX | 7 |
| GO:0042100 | B cell proliferation | 4/36 | 97/18866 | 3.40913E-05 | 0.00066424 | 0.000322116 | PRKCD/CDKN1A/CASP3/BAX | 4 |
| GO:0018209 | peptidyl-serine modification | 6/36 | 333/18866 | 3.60174E-05 | 0.000696242 | 0.000337635 | TNF/PRKCE/PRKCD/PRKCA/CCNB1/BAX | 6 |
| GO:0032496 | response to lipopolysaccharide | 6/36 | 334/18866 | 3.6626E-05 | 0.000702476 | 0.000340658 | TNF/PRKCE/PRKCA/IL1B/CASP3/OPRM1 | 6 |
| GO:0031099 | regeneration | 5/36 | 201/18866 | 3.76397E-05 | 0.000716321 | 0.000347372 | JUN/F7/PCNA/CDKN1A/CCNB1 | 5 |
| GO:0008585 | female gonad development | 4/36 | 100/18866 | 3.84251E-05 | 0.000725644 | 0.000351893 | ESR1/CASP3/BAX/PGR | 4 |
| GO:1902806 | regulation of cell cycle G1/S phase transition | 5/36 | 206/18866 | 4.23209E-05 | 0.000793114 | 0.000384612 | TP53/PCNA/CDKN1A/CCNB1/BAX | 5 |
| GO:0008630 | intrinsic apoptotic signaling pathway in response to DNA damage | 4/36 | 103/18866 | 4.31493E-05 | 0.000799426 | 0.000387673 | TP53/TNF/CDKN1A/BAX | 4 |
| GO:0046890 | regulation of lipid biosynthetic process | 5/36 | 207/18866 | 4.3309E-05 | 0.000799426 | 0.000387673 | TNF/PRKCD/IL1B/FASN/EIF6 | 5 |
| GO:0002573 | myeloid leukocyte differentiation | 5/36 | 210/18866 | 4.6381E-05 | 0.000846087 | 0.000410301 | JUN/TNF/PRKCA/MYC/FASN | 5 |
| GO:0046545 | development of primary female sexual characteristics | 4/36 | 105/18866 | 4.65262E-05 | 0.000846087 | 0.000410301 | ESR1/CASP3/BAX/PGR | 4 |
| GO:0060969 | negative regulation of gene silencing | 3/36 | 37/18866 | 4.74163E-05 | 0.000849686 | 0.000412046 | ESR1/TP53/TNF | 3 |
| GO:0098926 | postsynaptic signal transduction | 3/36 | 37/18866 | 4.74163E-05 | 0.000849686 | 0.000412046 | OPRM1/CHRM4/CHRM3 | 3 |
| GO:0032091 | negative regulation of protein binding | 4/36 | 106/18866 | 4.82852E-05 | 0.000858986 | 0.000416556 | PRKCD/CDKN1A/BAX/MAP2 | 4 |
| GO:0006913 | nucleocytoplasmic transport | 6/36 | 354/18866 | 5.06441E-05 | 0.000882651 | 0.000428033 | TP53/PRKCD/PKIA/IL1B/EIF6/CDKN1A | 6 |
| GO:0043523 | regulation of neuron apoptotic process | 5/36 | 214/18866 | 5.07366E-05 | 0.000882651 | 0.000428033 | JUN/TP53/TNF/CASP3/BAX | 5 |
| GO:0030224 | monocyte differentiation | 3/36 | 38/18866 | 5.14131E-05 | 0.000882651 | 0.000428033 | JUN/MYC/FASN | 3 |
| GO:1903131 | mononuclear cell differentiation | 3/36 | 38/18866 | 5.14131E-05 | 0.000882651 | 0.000428033 | JUN/MYC/FASN | 3 |
| GO:2000144 | positive regulation of DNA-templated transcription, initiation | 3/36 | 38/18866 | 5.14131E-05 | 0.000882651 | 0.000428033 | ESR1/JUN/TP53 | 3 |
| GO:0070374 | positive regulation of ERK1 and ERK2 cascade | 5/36 | 215/18866 | 5.18734E-05 | 0.000884369 | 0.000428865 | JUN/KDR/TNF/PRKCA/OPRM1 | 5 |
| GO:0002237 | response to molecule of bacterial origin | 6/36 | 356/18866 | 5.22543E-05 | 0.000884719 | 0.000429035 | TNF/PRKCE/PRKCA/IL1B/CASP3/OPRM1 | 6 |
| GO:0051169 | nuclear transport | 6/36 | 357/18866 | 5.30747E-05 | 0.000892454 | 0.000432786 | TP53/PRKCD/PKIA/IL1B/EIF6/CDKN1A | 6 |
| GO:2000278 | regulation of DNA biosynthetic process | 4/36 | 109/18866 | 5.38544E-05 | 0.000899162 | 0.000436039 | TP53/PCNA/MYC/CDKN1A | 4 |
| GO:1902749 | regulation of cell cycle G2/M phase transition | 5/36 | 217/18866 | 5.42061E-05 | 0.000899162 | 0.000436039 | CHEK1/TP53/PKIA/CDKN1A/CCNB1 | 5 |
| GO:0010948 | negative regulation of cell cycle process | 6/36 | 359/18866 | 5.47464E-05 | 0.00090203 | 0.00043743 | CHEK1/TP53/PCNA/CDKN1A/CCNB1/BAX | 6 |
| GO:0007623 | circadian rhythm | 5/36 | 218/18866 | 5.54025E-05 | 0.000903927 | 0.00043835 | NCOA2/JUN/F7/TP53/SLC6A4 | 5 |
| GO:0062197 | cellular response to chemical stress | 6/36 | 360/18866 | 5.5598E-05 | 0.000903927 | 0.00043835 | JUN/TP53/TNF/PRKCD/PCNA/CASP3 | 6 |
| GO:0046822 | regulation of nucleocytoplasmic transport | 4/36 | 112/18866 | 5.9879E-05 | 0.000963642 | 0.000467308 | TP53/PRKCD/PKIA/IL1B | 4 |
| GO:1902895 | positive regulation of pri-miRNA transcription by RNA polymerase II | 3/36 | 40/18866 | 6.00559E-05 | 0.000963642 | 0.000467308 | JUN/TP53/TNF | 3 |
| GO:0008406 | gonad development | 5/36 | 223/18866 | 6.16943E-05 | 0.0009798 | 0.000475143 | ESR1/AR/CASP3/BAX/PGR | 5 |
| GO:0051098 | regulation of binding | 6/36 | 367/18866 | 6.18611E-05 | 0.0009798 | 0.000475143 | JUN/PRKCD/CDKN1A/BAX/PON1/MAP2 | 6 |
| GO:0043200 | response to amino acid | 4/36 | 114/18866 | 6.41595E-05 | 0.001009689 | 0.000489638 | F7/TNF/PCNA/CASP3 | 4 |
| GO:0044706 | multi-multicellular organism process | 5/36 | 226/18866 | 6.5726E-05 | 0.001021249 | 0.000495244 | ESR1/AR/IL1B/SLC6A4/PGR | 5 |
| GO:2001020 | regulation of response to DNA damage stimulus | 5/36 | 226/18866 | 6.5726E-05 | 0.001021249 | 0.000495244 | CHEK1/TP53/PRKCD/PCNA/MYC | 5 |
| GO:0030518 | intracellular steroid hormone receptor signaling pathway | 4/36 | 116/18866 | 6.8659E-05 | 0.001060113 | 0.00051409 | ESR2/ESR1/AR/PGR | 4 |
| GO:0045137 | development of primary sexual characteristics | 5/36 | 229/18866 | 6.99584E-05 | 0.001073424 | 0.000520546 | ESR1/AR/CASP3/BAX/PGR | 5 |
| GO:0045446 | endothelial cell differentiation | 4/36 | 117/18866 | 7.09931E-05 | 0.001082246 | 0.000524824 | KDR/TNF/IL1B/FASN | 4 |
| GO:0097191 | extrinsic apoptotic signaling pathway | 5/36 | 230/18866 | 7.1415E-05 | 0.001082246 | 0.000524824 | AR/TNF/IL1B/CASP3/BAX | 5 |
| GO:0007569 | cell aging | 4/36 | 118/18866 | 7.33845E-05 | 0.00110527 | 0.000535989 | CHEK1/TP53/PRKCD/CDKN1A | 4 |
| GO:0046660 | female sex differentiation | 4/36 | 119/18866 | 7.58341E-05 | 0.0011352 | 0.000550503 | ESR1/CASP3/BAX/PGR | 4 |
| GO:0000302 | response to reactive oxygen species | 5/36 | 235/18866 | 7.90528E-05 | 0.00117621 | 0.000570391 | JUN/TNF/PRKCD/PCNA/CASP3 | 5 |
| GO:0035094 | response to nicotine | 3/36 | 45/18866 | 8.5691E-05 | 0.001267297 | 0.000614562 | TNF/CASP3/CHRNA2 | 3 |
| GO:0002761 | regulation of myeloid leukocyte differentiation | 4/36 | 123/18866 | 8.62319E-05 | 0.00126766 | 0.000614738 | JUN/TNF/PRKCA/MYC | 4 |
| GO:0061028 | establishment of endothelial barrier | 3/36 | 46/18866 | 9.15494E-05 | 0.001329904 | 0.000644923 | TNF/IL1B/FASN | 3 |
| GO:2000142 | regulation of DNA-templated transcription, initiation | 3/36 | 46/18866 | 9.15494E-05 | 0.001329904 | 0.000644923 | ESR1/JUN/TP53 | 3 |
| GO:0051402 | neuron apoptotic process | 5/36 | 245/18866 | 9.62086E-05 | 0.001383688 | 0.000671005 | JUN/TP53/TNF/CASP3/BAX | 5 |
| GO:0022602 | ovulation cycle process | 3/36 | 47/18866 | 9.76633E-05 | 0.001383688 | 0.000671005 | ESR1/CASP3/PGR | 3 |
| GO:0048806 | genitalia development | 3/36 | 47/18866 | 9.76633E-05 | 0.001383688 | 0.000671005 | ESR1/AR/BAX | 3 |
| GO:0006469 | negative regulation of protein kinase activity | 5/36 | 246/18866 | 9.807E-05 | 0.001383688 | 0.000671005 | PRKCD/PKIA/IL1B/CDKN1A/CASP3 | 5 |
| GO:0071216 | cellular response to biotic stimulus | 5/36 | 246/18866 | 9.807E-05 | 0.001383688 | 0.000671005 | TP53/TNF/PRKCE/PRKCA/IL1B | 5 |
| GO:0034763 | negative regulation of transmembrane transport | 4/36 | 128/18866 | 0.000100644 | 0.001411886 | 0.000684679 | TNF/PRKCE/IL1B/KCNH2 | 4 |
| GO:1901991 | negative regulation of mitotic cell cycle phase transition | 5/36 | 251/18866 | 0.000107797 | 0.001503643 | 0.000729176 | TP53/PCNA/CDKN1A/CCNB1/BAX | 5 |
| GO:0035196 | production of miRNAs involved in gene silencing by miRNA | 3/36 | 49/18866 | 0.000110678 | 0.001535107 | 0.000744434 | ESR1/TP53/TNF | 3 |
| GO:0001101 | response to acid chemical | 4/36 | 132/18866 | 0.000113374 | 0.001554548 | 0.000753861 | F7/TNF/PCNA/CASP3 | 4 |
| GO:0045926 | negative regulation of growth | 5/36 | 254/18866 | 0.000113979 | 0.001554548 | 0.000753861 | ESR2/TP53/CDKN1A/SLC6A4/MAP2 | 5 |
| GO:2000027 | regulation of animal organ morphogenesis | 5/36 | 254/18866 | 0.000113979 | 0.001554548 | 0.000753861 | ESR1/AR/TNF/MYC/BAX | 5 |
| GO:0097300 | programmed necrotic cell death | 3/36 | 50/18866 | 0.000117588 | 0.001594911 | 0.000773435 | TP53/TNF/BAX | 3 |
| GO:2001233 | regulation of apoptotic signaling pathway | 6/36 | 413/18866 | 0.000118493 | 0.00159836 | 0.000775108 | AR/TP53/TNF/PRKCD/IL1B/BAX | 6 |
| GO:0031334 | positive regulation of protein-containing complex assembly | 5/36 | 257/18866 | 0.00012043 | 0.001615611 | 0.000783474 | ESR1/TP53/TNF/PRKCE/BAX | 5 |
| GO:0003158 | endothelium development | 4/36 | 135/18866 | 0.000123662 | 0.001649941 | 0.000800121 | KDR/TNF/IL1B/FASN | 4 |
| GO:1902893 | regulation of pri-miRNA transcription by RNA polymerase II | 3/36 | 51/18866 | 0.000124774 | 0.001655782 | 0.000802954 | JUN/TP53/TNF | 3 |
| GO:0045637 | regulation of myeloid cell differentiation | 5/36 | 263/18866 | 0.000134173 | 0.001770936 | 0.000858797 | JUN/TNF/PRKCA/MYC/EIF6 | 5 |
| GO:0043401 | steroid hormone mediated signaling pathway | 4/36 | 139/18866 | 0.000138411 | 0.0018171 | 0.000881184 | ESR2/ESR1/AR/PGR | 4 |
| GO:0043949 | regulation of cAMP-mediated signaling | 3/36 | 53/18866 | 0.000139991 | 0.001818396 | 0.000881812 | PRKCA/PDE3A/OPRM1 | 3 |
| GO:0061614 | pri-miRNA transcription by RNA polymerase II | 3/36 | 53/18866 | 0.000139991 | 0.001818396 | 0.000881812 | JUN/TP53/TNF | 3 |
| GO:0033673 | negative regulation of kinase activity | 5/36 | 268/18866 | 0.000146518 | 0.001892796 | 0.000917891 | PRKCD/PKIA/IL1B/CDKN1A/CASP3 | 5 |
| GO:0031050 | dsRNA processing | 3/36 | 54/18866 | 0.000148031 | 0.001892796 | 0.000917891 | ESR1/TP53/TNF | 3 |
| GO:0070918 | production of small RNA involved in gene silencing by RNA | 3/36 | 54/18866 | 0.000148031 | 0.001892796 | 0.000917891 | ESR1/TP53/TNF | 3 |
| GO:0051091 | positive regulation of DNA-binding transcription factor activity | 5/36 | 270/18866 | 0.000151693 | 0.001929569 | 0.000935724 | ESR2/ESR1/AR/TNF/IL1B | 5 |
| GO:0001660 | fever generation | 2/36 | 10/18866 | 0.000157787 | 0.001966328 | 0.00095355 | TNF/IL1B | 2 |
| GO:0032070 | regulation of deoxyribonuclease activity | 2/36 | 10/18866 | 0.000157787 | 0.001966328 | 0.00095355 | PRKCD/PCNA | 2 |
| GO:0034350 | regulation of glial cell apoptotic process | 2/36 | 10/18866 | 0.000157787 | 0.001966328 | 0.00095355 | PRKCD/PRKCA | 2 |
| GO:0060068 | vagina development | 2/36 | 10/18866 | 0.000157787 | 0.001966328 | 0.00095355 | ESR1/BAX | 2 |
| GO:0044839 | cell cycle G2/M phase transition | 5/36 | 273/18866 | 0.000159717 | 0.001980332 | 0.000960341 | CHEK1/TP53/PKIA/CDKN1A/CCNB1 | 5 |
| GO:0060688 | regulation of morphogenesis of a branching structure | 3/36 | 56/18866 | 0.000165001 | 0.002035568 | 0.000987127 | ESR1/AR/TNF | 3 |
| GO:0042542 | response to hydrogen peroxide | 4/36 | 146/18866 | 0.00016723 | 0.002052749 | 0.000995459 | JUN/PRKCD/PCNA/CASP3 | 4 |
| GO:0051384 | response to glucocorticoid | 4/36 | 147/18866 | 0.000171675 | 0.002096826 | 0.001016834 | TNF/PCNA/CDKN1A/CASP3 | 4 |
| GO:0043491 | protein kinase B signaling | 5/36 | 278/18866 | 0.000173809 | 0.002103561 | 0.001020099 | ESR1/KDR/F7/TNF/IL1B | 5 |
| GO:0030520 | intracellular estrogen receptor signaling pathway | 3/36 | 57/18866 | 0.00017394 | 0.002103561 | 0.001020099 | ESR2/ESR1/AR | 3 |
| GO:0007548 | sex differentiation | 5/36 | 280/18866 | 0.000179705 | 0.002162623 | 0.001048741 | ESR1/AR/CASP3/BAX/PGR | 5 |
| GO:0031056 | regulation of histone modification | 4/36 | 149/18866 | 0.00018082 | 0.002165434 | 0.001050104 | CHEK1/TP53/IL1B/CCNB1 | 4 |
| GO:0002763 | positive regulation of myeloid leukocyte differentiation | 3/36 | 58/18866 | 0.000183188 | 0.002183134 | 0.001058688 | JUN/TNF/PRKCA | 3 |
| GO:1901990 | regulation of mitotic cell cycle phase transition | 6/36 | 448/18866 | 0.000184514 | 0.002188314 | 0.0010612 | TP53/PKIA/PCNA/CDKN1A/CCNB1/BAX | 6 |
| GO:0006606 | protein import into nucleus | 4/36 | 150/18866 | 0.000185523 | 0.002189703 | 0.001061873 | TP53/PRKCD/PKIA/CDKN1A | 4 |
| GO:0031652 | positive regulation of heat generation | 2/36 | 11/18866 | 0.000192619 | 0.002251809 | 0.001091991 | TNF/IL1B | 2 |
| GO:0045899 | positive regulation of RNA polymerase II transcription preinitiation complex assembly | 2/36 | 11/18866 | 0.000192619 | 0.002251809 | 0.001091991 | ESR1/TP53 | 2 |
| GO:0009896 | positive regulation of catabolic process | 6/36 | 454/18866 | 0.000198294 | 0.002307165 | 0.001118835 | KDR/TNF/PRKCE/PRKCD/IL1B/BAX | 6 |
| GO:0045834 | positive regulation of lipid metabolic process | 4/36 | 153/18866 | 0.00020016 | 0.002307976 | 0.001119229 | TNF/PRKCE/PRKCD/IL1B | 4 |
| GO:0051090 | regulation of DNA-binding transcription factor activity | 6/36 | 455/18866 | 0.000200668 | 0.002307976 | 0.001119229 | ESR2/ESR1/JUN/AR/TNF/IL1B | 6 |
| GO:0001836 | release of cytochrome c from mitochondria | 3/36 | 60/18866 | 0.000202628 | 0.002307976 | 0.001119229 | JUN/TP53/BAX | 3 |
| GO:0061900 | glial cell activation | 3/36 | 60/18866 | 0.000202628 | 0.002307976 | 0.001119229 | JUN/TNF/IL1B | 3 |
| GO:0019932 | second-messenger-mediated signaling | 6/36 | 456/18866 | 0.000203064 | 0.002307976 | 0.001119229 | KDR/TNF/PRKCA/PDE3A/OPRM1/CHRM3 | 6 |
| GO:0006979 | response to oxidative stress | 6/36 | 458/18866 | 0.000207925 | 0.002352333 | 0.001140739 | JUN/TP53/TNF/PRKCD/PCNA/CASP3 | 6 |
| GO:1902105 | regulation of leukocyte differentiation | 5/36 | 290/18866 | 0.000211515 | 0.002381967 | 0.00115511 | JUN/TNF/PRKCA/MYC/IL1B | 5 |
| GO:0032409 | regulation of transporter activity | 5/36 | 291/18866 | 0.000214918 | 0.002409236 | 0.001168333 | PRKCE/PRKCD/PON1/OPRM1/CHRM3 | 5 |
| GO:0050768 | negative regulation of neurogenesis | 5/36 | 295/18866 | 0.000228949 | 0.002518998 | 0.001221562 | TP53/TNF/IL1B/SLC6A4/MAP2 | 5 |
| GO:0007077 | mitotic nuclear envelope disassembly | 2/36 | 12/18866 | 0.000230865 | 0.002518998 | 0.001221562 | PRKCA/CCNB1 | 2 |
| GO:0042368 | vitamin D biosynthetic process | 2/36 | 12/18866 | 0.000230865 | 0.002518998 | 0.001221562 | TNF/IL1B | 2 |
| GO:0060736 | prostate gland growth | 2/36 | 12/18866 | 0.000230865 | 0.002518998 | 0.001221562 | ESR1/AR | 2 |
| GO:0090154 | positive regulation of sphingolipid biosynthetic process | 2/36 | 12/18866 | 0.000230865 | 0.002518998 | 0.001221562 | TNF/PRKCD | 2 |
| GO:2000304 | positive regulation of ceramide biosynthetic process | 2/36 | 12/18866 | 0.000230865 | 0.002518998 | 0.001221562 | TNF/PRKCD | 2 |
| GO:1902107 | positive regulation of leukocyte differentiation | 4/36 | 159/18866 | 0.000231911 | 0.002519213 | 0.001221666 | JUN/TNF/PRKCA/IL1B | 4 |
| GO:0016570 | histone modification | 6/36 | 468/18866 | 0.00023363 | 0.002526702 | 0.001225297 | CHEK1/TP53/PRKCD/PRKCA/IL1B/CCNB1 | 6 |
| GO:0001885 | endothelial cell development | 3/36 | 64/18866 | 0.000245417 | 0.002619564 | 0.00127033 | TNF/IL1B/FASN | 3 |
| GO:0060135 | maternal process involved in female pregnancy | 3/36 | 64/18866 | 0.000245417 | 0.002619564 | 0.00127033 | ESR1/AR/PGR | 3 |
| GO:0070265 | necrotic cell death | 3/36 | 64/18866 | 0.000245417 | 0.002619564 | 0.00127033 | TP53/TNF/BAX | 3 |
| GO:0010632 | regulation of epithelial cell migration | 5/36 | 301/18866 | 0.000251293 | 0.002670673 | 0.001295115 | JUN/KDR/TNF/PRKCE/PRKCA | 5 |
| GO:0048285 | organelle fission | 6/36 | 476/18866 | 0.000255951 | 0.002708444 | 0.001313431 | CHEK1/KDR/PRKCA/IL1B/CCNB1/PDE3A | 6 |
| GO:0031960 | response to corticosteroid | 4/36 | 164/18866 | 0.00026102 | 0.002750229 | 0.001333694 | TNF/PCNA/CDKN1A/CASP3 | 4 |
| GO:0046824 | positive regulation of nucleocytoplasmic transport | 3/36 | 66/18866 | 0.00026884 | 0.002799249 | 0.001357466 | TP53/PRKCD/IL1B | 3 |
| GO:0016569 | covalent chromatin modification | 6/36 | 481/18866 | 0.000270735 | 0.002799249 | 0.001357466 | CHEK1/TP53/PRKCD/PRKCA/IL1B/CCNB1 | 6 |
| GO:0010623 | programmed cell death involved in cell development | 2/36 | 13/18866 | 0.000272513 | 0.002799249 | 0.001357466 | IL1B/BAX | 2 |
| GO:0030656 | regulation of vitamin metabolic process | 2/36 | 13/18866 | 0.000272513 | 0.002799249 | 0.001357466 | TNF/IL1B | 2 |
| GO:0031650 | regulation of heat generation | 2/36 | 13/18866 | 0.000272513 | 0.002799249 | 0.001357466 | TNF/IL1B | 2 |
| GO:0033127 | regulation of histone phosphorylation | 2/36 | 13/18866 | 0.000272513 | 0.002799249 | 0.001357466 | IL1B/CCNB1 | 2 |
| GO:0051051 | negative regulation of transport | 6/36 | 483/18866 | 0.000276834 | 0.002831781 | 0.001373242 | TNF/PRKCE/PKIA/IL1B/OPRM1/KCNH2 | 6 |
| GO:0034250 | positive regulation of cellular amide metabolic process | 4/36 | 167/18866 | 0.000279697 | 0.002849196 | 0.001381688 | TNF/PRKCD/EIF6/CASP3 | 4 |
| GO:0034599 | cellular response to oxidative stress | 5/36 | 310/18866 | 0.000287884 | 0.002920473 | 0.001416252 | JUN/TP53/TNF/PRKCD/PCNA | 5 |
| GO:0034765 | regulation of ion transmembrane transport | 6/36 | 489/18866 | 0.000295784 | 0.002988274 | 0.001449132 | SCN5A/PRKCE/BAX/OPRM1/KCNH2/CHRM3 | 6 |
| GO:0034614 | cellular response to reactive oxygen species | 4/36 | 170/18866 | 0.000299317 | 0.003011573 | 0.00146043 | JUN/TNF/PRKCD/PCNA | 4 |
| GO:0051170 | import into nucleus | 4/36 | 171/18866 | 0.000306072 | 0.003054494 | 0.001481245 | TP53/PRKCD/PKIA/CDKN1A | 4 |
| GO:1905952 | regulation of lipid localization | 4/36 | 171/18866 | 0.000306072 | 0.003054494 | 0.001481245 | TNF/PRKCD/IL1B/PON1 | 4 |
| GO:0045898 | regulation of RNA polymerase II transcription preinitiation complex assembly | 2/36 | 14/18866 | 0.000317551 | 0.003143497 | 0.001524406 | ESR1/TP53 | 2 |
| GO:0106070 | regulation of adenylate cyclase-activating G protein-coupled receptor signaling pathway | 2/36 | 14/18866 | 0.000317551 | 0.003143497 | 0.001524406 | PRKCA/OPRM1 | 2 |
| GO:0032370 | positive regulation of lipid transport | 3/36 | 70/18866 | 0.000319917 | 0.003154206 | 0.001529599 | PRKCD/IL1B/PON1 | 3 |
| GO:1903706 | regulation of hemopoiesis | 6/36 | 498/18866 | 0.000326113 | 0.003199254 | 0.001551444 | JUN/TNF/PRKCA/MYC/IL1B/EIF6 | 6 |
| GO:0007568 | aging | 5/36 | 319/18866 | 0.000328396 | 0.003199254 | 0.001551444 | CHEK1/JUN/TP53/PRKCD/CDKN1A | 5 |
| GO:0051961 | negative regulation of nervous system development | 5/36 | 319/18866 | 0.000328396 | 0.003199254 | 0.001551444 | TP53/TNF/IL1B/SLC6A4/MAP2 | 5 |
| GO:1901214 | regulation of neuron death | 5/36 | 321/18866 | 0.000337958 | 0.003279399 | 0.00159031 | JUN/TP53/TNF/CASP3/BAX | 5 |
| GO:0010634 | positive regulation of epithelial cell migration | 4/36 | 176/18866 | 0.000341496 | 0.003300683 | 0.001600631 | JUN/KDR/PRKCE/PRKCA | 4 |
| GO:0071496 | cellular response to external stimulus | 5/36 | 326/18866 | 0.000362791 | 0.003429177 | 0.001662943 | CHEK1/JUN/TP53/IL1B/CDKN1A | 5 |
| GO:0048469 | cell maturation | 4/36 | 179/18866 | 0.000364111 | 0.003429177 | 0.001662943 | CDKN1A/CCNB1/PGR/PDE3A | 4 |
| GO:0042362 | fat-soluble vitamin biosynthetic process | 2/36 | 15/18866 | 0.000365965 | 0.003429177 | 0.001662943 | TNF/IL1B | 2 |
| GO:0044849 | estrous cycle | 2/36 | 15/18866 | 0.000365965 | 0.003429177 | 0.001662943 | PCNA/OPRM1 | 2 |
| GO:0051044 | positive regulation of membrane protein ectodomain proteolysis | 2/36 | 15/18866 | 0.000365965 | 0.003429177 | 0.001662943 | TNF/IL1B | 2 |
| GO:1900119 | positive regulation of execution phase of apoptosis | 2/36 | 15/18866 | 0.000365965 | 0.003429177 | 0.001662943 | TP53/BAX | 2 |
| GO:1901550 | regulation of endothelial cell development | 2/36 | 15/18866 | 0.000365965 | 0.003429177 | 0.001662943 | TNF/IL1B | 2 |
| GO:1903140 | regulation of establishment of endothelial barrier | 2/36 | 15/18866 | 0.000365965 | 0.003429177 | 0.001662943 | TNF/IL1B | 2 |
| GO:0042113 | B cell activation | 5/36 | 328/18866 | 0.000373102 | 0.003482756 | 0.001688926 | TP53/PRKCD/CDKN1A/CASP3/BAX | 5 |
| GO:0050796 | regulation of insulin secretion | 4/36 | 181/18866 | 0.000379772 | 0.003531588 | 0.001712606 | TNF/PRKCE/PRKCA/IL1B | 4 |
| GO:0031100 | animal organ regeneration | 3/36 | 75/18866 | 0.000392063 | 0.003618476 | 0.001754742 | F7/PCNA/CDKN1A | 3 |
| GO:0043627 | response to estrogen | 3/36 | 75/18866 | 0.000392063 | 0.003618476 | 0.001754742 | ESR1/AR/F7 | 3 |
| GO:0008202 | steroid metabolic process | 5/36 | 332/18866 | 0.00039439 | 0.003626319 | 0.001758545 | ESR1/TNF/IL1B/FASN/PON1 | 5 |
| GO:2001235 | positive regulation of apoptotic signaling pathway | 4/36 | 183/18866 | 0.000395909 | 0.003626704 | 0.001758732 | TP53/TNF/PRKCD/BAX | 4 |
| GO:0001936 | regulation of endothelial cell proliferation | 4/36 | 184/18866 | 0.000404159 | 0.003688516 | 0.001788707 | JUN/KDR/TNF/PRKCA | 4 |
| GO:0002070 | epithelial cell maturation | 2/36 | 16/18866 | 0.000417744 | 0.003729315 | 0.001808492 | CDKN1A/PGR | 2 |
| GO:0030397 | membrane disassembly | 2/36 | 16/18866 | 0.000417744 | 0.003729315 | 0.001808492 | PRKCA/CCNB1 | 2 |
| GO:0034116 | positive regulation of heterotypic cell-cell adhesion | 2/36 | 16/18866 | 0.000417744 | 0.003729315 | 0.001808492 | TNF/IL1B | 2 |
| GO:0051081 | nuclear envelope disassembly | 2/36 | 16/18866 | 0.000417744 | 0.003729315 | 0.001808492 | PRKCA/CCNB1 | 2 |
| GO:0071391 | cellular response to estrogen stimulus | 2/36 | 16/18866 | 0.000417744 | 0.003729315 | 0.001808492 | ESR1/AR | 2 |
| GO:1903729 | regulation of plasma membrane organization | 2/36 | 16/18866 | 0.000417744 | 0.003729315 | 0.001808492 | AR/PRKCD | 2 |
| GO:0150076 | neuroinflammatory response | 3/36 | 77/18866 | 0.000423613 | 0.003768005 | 0.001827254 | JUN/TNF/IL1B | 3 |
| GO:1903829 | positive regulation of cellular protein localization | 5/36 | 338/18866 | 0.000428033 | 0.00379358 | 0.001839657 | TP53/TNF/PRKCE/PRKCD/IL1B | 5 |
| GO:0051783 | regulation of nuclear division | 4/36 | 188/18866 | 0.000438394 | 0.00387143 | 0.001877409 | CHEK1/IL1B/CCNB1/PDE3A | 4 |
| GO:0072332 | intrinsic apoptotic signaling pathway by p53 class mediator | 3/36 | 78/18866 | 0.000439981 | 0.00387152 | 0.001877453 | TP53/CDKN1A/BAX | 3 |
| GO:0030308 | negative regulation of cell growth | 4/36 | 190/18866 | 0.000456267 | 0.003984523 | 0.001932252 | ESR2/TP53/CDKN1A/MAP2 | 4 |
| GO:0090398 | cellular senescence | 3/36 | 79/18866 | 0.000456751 | 0.003984523 | 0.001932252 | TP53/PRKCD/CDKN1A | 3 |
| GO:0010721 | negative regulation of cell development | 5/36 | 343/18866 | 0.000457693 | 0.003984523 | 0.001932252 | TP53/TNF/IL1B/SLC6A4/MAP2 | 5 |
| GO:0035265 | organ growth | 4/36 | 191/18866 | 0.000465396 | 0.004016985 | 0.001947995 | ESR1/AR/CCNB1/SLC6A4 | 4 |
| GO:0006978 | DNA damage response, signal transduction by p53 class mediator resulting in transcription of p21 class mediator | 2/36 | 17/18866 | 0.000472875 | 0.004016985 | 0.001947995 | TP53/CDKN1A | 2 |
| GO:0030540 | female genitalia development | 2/36 | 17/18866 | 0.000472875 | 0.004016985 | 0.001947995 | ESR1/BAX | 2 |
| GO:0030730 | sequestering of triglyceride | 2/36 | 17/18866 | 0.000472875 | 0.004016985 | 0.001947995 | TNF/IL1B | 2 |
| GO:0031649 | heat generation | 2/36 | 17/18866 | 0.000472875 | 0.004016985 | 0.001947995 | TNF/IL1B | 2 |
| GO:0070242 | thymocyte apoptotic process | 2/36 | 17/18866 | 0.000472875 | 0.004016985 | 0.001947995 | TP53/BAX | 2 |
| GO:0071850 | mitotic cell cycle arrest | 2/36 | 17/18866 | 0.000472875 | 0.004016985 | 0.001947995 | TP53/CDKN1A | 2 |
| GO:1904062 | regulation of cation transmembrane transport | 5/36 | 349/18866 | 0.000495307 | 0.00419303 | 0.002033366 | SCN5A/PRKCE/BAX/OPRM1/KCNH2 | 5 |
| GO:0042098 | T cell proliferation | 4/36 | 195/18866 | 0.00050322 | 0.004216401 | 0.002044699 | TP53/IL1B/CASP3/BAX | 4 |
| GO:1902275 | regulation of chromatin organization | 4/36 | 195/18866 | 0.00050322 | 0.004216401 | 0.002044699 | CHEK1/TP53/IL1B/CCNB1 | 4 |
| GO:1905475 | regulation of protein localization to membrane | 4/36 | 195/18866 | 0.00050322 | 0.004216401 | 0.002044699 | AR/TP53/TNF/PRKCE | 4 |
| GO:0030193 | regulation of blood coagulation | 3/36 | 82/18866 | 0.000509509 | 0.004240147 | 0.002056215 | F7/PRKCD/PRKCA | 3 |
| GO:1902930 | regulation of alcohol biosynthetic process | 3/36 | 82/18866 | 0.000509509 | 0.004240147 | 0.002056215 | TNF/IL1B/FASN | 3 |
| GO:0007565 | female pregnancy | 4/36 | 196/18866 | 0.000513009 | 0.004240527 | 0.002056399 | ESR1/AR/IL1B/PGR | 4 |
| GO:0071897 | DNA biosynthetic process | 4/36 | 196/18866 | 0.000513009 | 0.004240527 | 0.002056399 | TP53/PCNA/MYC/CDKN1A | 4 |
| GO:0050708 | regulation of protein secretion | 5/36 | 352/18866 | 0.000514968 | 0.004242434 | 0.002057323 | TNF/PRKCE/PRKCA/IL1B/OPRM1 | 5 |
| GO:0048638 | regulation of developmental growth | 5/36 | 353/18866 | 0.00052165 | 0.004276904 | 0.002074039 | AR/CDKN1A/CCNB1/SLC6A4/MAP2 | 5 |
| GO:1900046 | regulation of hemostasis | 3/36 | 83/18866 | 0.000527924 | 0.004276904 | 0.002074039 | F7/PRKCD/PRKCA | 3 |
| GO:0051222 | positive regulation of protein transport | 5/36 | 354/18866 | 0.000528398 | 0.004276904 | 0.002074039 | TP53/TNF/PRKCE/PRKCD/IL1B | 5 |
| GO:0042772 | DNA damage response, signal transduction resulting in transcription | 2/36 | 18/18866 | 0.000531346 | 0.004276904 | 0.002074039 | TP53/CDKN1A | 2 |
| GO:0070230 | positive regulation of lymphocyte apoptotic process | 2/36 | 18/18866 | 0.000531346 | 0.004276904 | 0.002074039 | TP53/BAX | 2 |
| GO:0150078 | positive regulation of neuroinflammatory response | 2/36 | 18/18866 | 0.000531346 | 0.004276904 | 0.002074039 | TNF/IL1B | 2 |
| GO:2000303 | regulation of ceramide biosynthetic process | 2/36 | 18/18866 | 0.000531346 | 0.004276904 | 0.002074039 | TNF/PRKCD | 2 |
| GO:0033044 | regulation of chromosome organization | 5/36 | 356/18866 | 0.000542091 | 0.004343745 | 0.002106453 | CHEK1/TP53/MYC/IL1B/CCNB1 | 5 |
| GO:0001935 | endothelial cell proliferation | 4/36 | 199/18866 | 0.000543189 | 0.004343745 | 0.002106453 | JUN/KDR/TNF/PRKCA | 4 |
| GO:0009755 | hormone-mediated signaling pathway | 4/36 | 200/18866 | 0.000553525 | 0.004383559 | 0.00212576 | ESR2/ESR1/AR/PGR | 4 |
| GO:0017038 | protein import | 4/36 | 200/18866 | 0.000553525 | 0.004383559 | 0.00212576 | TP53/PRKCD/PKIA/CDKN1A | 4 |
| GO:0051054 | positive regulation of DNA metabolic process | 4/36 | 200/18866 | 0.000553525 | 0.004383559 | 0.00212576 | PRKCD/PCNA/MYC/BAX | 4 |
| GO:0032386 | regulation of intracellular transport | 5/36 | 358/18866 | 0.000556051 | 0.004389405 | 0.002128596 | TP53/PRKCD/PKIA/IL1B/MAP2 | 5 |
| GO:0110110 | positive regulation of animal organ morphogenesis | 3/36 | 85/18866 | 0.000566024 | 0.004453809 | 0.002159828 | AR/MYC/BAX | 3 |
| GO:0070997 | neuron death | 5/36 | 360/18866 | 0.00057028 | 0.004472962 | 0.002169116 | JUN/TP53/TNF/CASP3/BAX | 5 |
| GO:0007193 | adenylate cyclase-inhibiting G protein-coupled receptor signaling pathway | 3/36 | 86/18866 | 0.000585716 | 0.004550538 | 0.002206736 | OPRM1/CHRM4/CHRM3 | 3 |
| GO:0002922 | positive regulation of humoral immune response | 2/36 | 19/18866 | 0.000593146 | 0.004550538 | 0.002206736 | TNF/IL1B | 2 |
| GO:0060252 | positive regulation of glial cell proliferation | 2/36 | 19/18866 | 0.000593146 | 0.004550538 | 0.002206736 | TNF/IL1B | 2 |
| GO:0060749 | mammary gland alveolus development | 2/36 | 19/18866 | 0.000593146 | 0.004550538 | 0.002206736 | ESR1/AR | 2 |
| GO:0061377 | mammary gland lobule development | 2/36 | 19/18866 | 0.000593146 | 0.004550538 | 0.002206736 | ESR1/AR | 2 |
| GO:0090153 | regulation of sphingolipid biosynthetic process | 2/36 | 19/18866 | 0.000593146 | 0.004550538 | 0.002206736 | TNF/PRKCD | 2 |
| GO:1905038 | regulation of membrane lipid metabolic process | 2/36 | 19/18866 | 0.000593146 | 0.004550538 | 0.002206736 | TNF/PRKCD | 2 |
| GO:1903708 | positive regulation of hemopoiesis | 4/36 | 204/18866 | 0.000596271 | 0.004560267 | 0.002211453 | JUN/TNF/PRKCA/IL1B | 4 |
| GO:1904705 | regulation of vascular associated smooth muscle cell proliferation | 3/36 | 87/18866 | 0.000605841 | 0.00459974 | 0.002230595 | JUN/TNF/CDKN1A | 3 |
| GO:1990874 | vascular associated smooth muscle cell proliferation | 3/36 | 87/18866 | 0.000605841 | 0.00459974 | 0.002230595 | JUN/TNF/CDKN1A | 3 |
| GO:0010631 | epithelial cell migration | 5/36 | 365/18866 | 0.000607053 | 0.00459974 | 0.002230595 | JUN/KDR/TNF/PRKCE/PRKCA | 5 |
| GO:0010038 | response to metal ion | 5/36 | 366/18866 | 0.000614617 | 0.004642724 | 0.00225144 | JUN/SCN5A/PCNA/CCNB1/CASP3 | 5 |
| GO:0071383 | cellular response to steroid hormone stimulus | 4/36 | 206/18866 | 0.000618502 | 0.00465774 | 0.002258722 | ESR2/ESR1/AR/PGR | 4 |
| GO:0046889 | positive regulation of lipid biosynthetic process | 3/36 | 88/18866 | 0.000626404 | 0.004688484 | 0.002273631 | TNF/PRKCD/IL1B | 3 |
| GO:0050818 | regulation of coagulation | 3/36 | 88/18866 | 0.000626404 | 0.004688484 | 0.002273631 | F7/PRKCD/PRKCA | 3 |
| GO:0090132 | epithelium migration | 5/36 | 368/18866 | 0.000629958 | 0.00470075 | 0.002279579 | JUN/KDR/TNF/PRKCE/PRKCA | 5 |
| GO:0071222 | cellular response to lipopolysaccharide | 4/36 | 208/18866 | 0.000641317 | 0.004756596 | 0.002306661 | TNF/PRKCE/PRKCA/IL1B | 4 |
| GO:0071456 | cellular response to hypoxia | 4/36 | 208/18866 | 0.000641317 | 0.004756596 | 0.002306661 | TP53/PRKCE/MYC/CCNB1 | 4 |
| GO:1904951 | positive regulation of establishment of protein localization | 5/36 | 370/18866 | 0.000645585 | 0.00477383 | 0.002315019 | TP53/TNF/PRKCE/PRKCD/IL1B | 5 |
| GO:0009612 | response to mechanical stimulus | 4/36 | 209/18866 | 0.000652946 | 0.00481376 | 0.002334382 | CHEK1/JUN/IL1B/CCNB1 | 4 |
| GO:1902004 | positive regulation of amyloid-beta formation | 2/36 | 20/18866 | 0.00065826 | 0.004838409 | 0.002346336 | TNF/CASP3 | 2 |
| GO:0006109 | regulation of carbohydrate metabolic process | 4/36 | 210/18866 | 0.000664724 | 0.004871334 | 0.002362302 | NCOA2/TP53/PRKCE/EIF6 | 4 |
| GO:0070542 | response to fatty acid | 3/36 | 90/18866 | 0.000668858 | 0.004872539 | 0.002362886 | PRKCE/CCNB1/PON1 | 3 |
| GO:1903351 | cellular response to dopamine | 3/36 | 90/18866 | 0.000668858 | 0.004872539 | 0.002362886 | OPRM1/CHRM4/CHRM3 | 3 |
| GO:0043393 | regulation of protein binding | 4/36 | 211/18866 | 0.000676652 | 0.004893486 | 0.002373044 | PRKCD/CDKN1A/BAX/MAP2 | 4 |
| GO:0018108 | peptidyl-tyrosine phosphorylation | 5/36 | 374/18866 | 0.000677713 | 0.004893486 | 0.002373044 | KDR/TP53/TNF/PRKCE/PRKCD | 5 |
| GO:0090130 | tissue migration | 5/36 | 374/18866 | 0.000677713 | 0.004893486 | 0.002373044 | JUN/KDR/TNF/PRKCE/PRKCA | 5 |
| GO:0034103 | regulation of tissue remodeling | 3/36 | 91/18866 | 0.000690756 | 0.004958496 | 0.00240457 | TP53/PRKCA/BAX | 3 |
| GO:1903350 | response to dopamine | 3/36 | 91/18866 | 0.000690756 | 0.004958496 | 0.00240457 | OPRM1/CHRM4/CHRM3 | 3 |
| GO:0030073 | insulin secretion | 4/36 | 213/18866 | 0.000700963 | 0.004999566 | 0.002424486 | TNF/PRKCE/PRKCA/IL1B | 4 |
| GO:0090276 | regulation of peptide hormone secretion | 4/36 | 213/18866 | 0.000700963 | 0.004999566 | 0.002424486 | TNF/PRKCE/PRKCA/IL1B | 4 |
| GO:0018212 | peptidyl-tyrosine modification | 5/36 | 377/18866 | 0.000702587 | 0.004999566 | 0.002424486 | KDR/TP53/TNF/PRKCE/PRKCD | 5 |
| GO:0031058 | positive regulation of histone modification | 3/36 | 92/18866 | 0.000713107 | 0.00505976 | 0.002453677 | TP53/IL1B/CCNB1 | 3 |
| GO:0009110 | vitamin biosynthetic process | 2/36 | 21/18866 | 0.000726678 | 0.005068168 | 0.002457755 | TNF/IL1B | 2 |
| GO:0010829 | negative regulation of glucose transmembrane transport | 2/36 | 21/18866 | 0.000726678 | 0.005068168 | 0.002457755 | TNF/IL1B | 2 |
| GO:0010893 | positive regulation of steroid biosynthetic process | 2/36 | 21/18866 | 0.000726678 | 0.005068168 | 0.002457755 | TNF/IL1B | 2 |
| GO:0046827 | positive regulation of protein export from nucleus | 2/36 | 21/18866 | 0.000726678 | 0.005068168 | 0.002457755 | TP53/IL1B | 2 |
| GO:0061042 | vascular wound healing | 2/36 | 21/18866 | 0.000726678 | 0.005068168 | 0.002457755 | KDR/TNF | 2 |
| GO:0090330 | regulation of platelet aggregation | 2/36 | 21/18866 | 0.000726678 | 0.005068168 | 0.002457755 | PRKCD/PRKCA | 2 |
| GO:0002791 | regulation of peptide secretion | 5/36 | 381/18866 | 0.000736811 | 0.005124281 | 0.002484966 | TNF/PRKCE/PRKCA/IL1B/OPRM1 | 5 |
| GO:1905954 | positive regulation of lipid localization | 3/36 | 94/18866 | 0.000759182 | 0.005264948 | 0.002553181 | PRKCD/IL1B/PON1 | 3 |
| GO:0036294 | cellular response to decreased oxygen levels | 4/36 | 218/18866 | 0.000764446 | 0.00528652 | 0.002563642 | TP53/PRKCE/MYC/CCNB1 | 4 |
| GO:0097194 | execution phase of apoptosis | 3/36 | 95/18866 | 0.000782914 | 0.005399025 | 0.0026182 | TP53/CASP3/BAX | 3 |
| GO:0032069 | regulation of nuclease activity | 2/36 | 22/18866 | 0.000798388 | 0.005459726 | 0.002647637 | PRKCD/PCNA | 2 |
| GO:0042359 | vitamin D metabolic process | 2/36 | 22/18866 | 0.000798388 | 0.005459726 | 0.002647637 | TNF/IL1B | 2 |
| GO:0045655 | regulation of monocyte differentiation | 2/36 | 22/18866 | 0.000798388 | 0.005459726 | 0.002647637 | JUN/MYC | 2 |
| GO:0032388 | positive regulation of intracellular transport | 4/36 | 222/18866 | 0.000818086 | 0.005550077 | 0.002691451 | TP53/PRKCD/IL1B/MAP2 | 4 |
| GO:0071219 | cellular response to molecule of bacterial origin | 4/36 | 222/18866 | 0.000818086 | 0.005550077 | 0.002691451 | TNF/PRKCE/PRKCA/IL1B | 4 |
| GO:0031331 | positive regulation of cellular catabolic process | 5/36 | 390/18866 | 0.000818382 | 0.005550077 | 0.002691451 | KDR/TNF/PRKCD/IL1B/BAX | 5 |
| GO:0050810 | regulation of steroid biosynthetic process | 3/36 | 98/18866 | 0.00085693 | 0.005795491 | 0.002810462 | TNF/IL1B/FASN | 3 |
| GO:0051043 | regulation of membrane protein ectodomain proteolysis | 2/36 | 23/18866 | 0.000873376 | 0.005874352 | 0.002848705 | TNF/IL1B | 2 |
| GO:2000178 | negative regulation of neural precursor cell proliferation | 2/36 | 23/18866 | 0.000873376 | 0.005874352 | 0.002848705 | TP53/SLC6A4 | 2 |
| GO:0042116 | macrophage activation | 3/36 | 101/18866 | 0.000935255 | 0.006256269 | 0.003033912 | JUN/TNF/PRKCE | 3 |
| GO:0045639 | positive regulation of myeloid cell differentiation | 3/36 | 101/18866 | 0.000935255 | 0.006256269 | 0.003033912 | JUN/TNF/PRKCA | 3 |
| GO:0044346 | fibroblast apoptotic process | 2/36 | 24/18866 | 0.000951632 | 0.006314206 | 0.003062008 | TP53/MYC | 2 |
| GO:0050995 | negative regulation of lipid catabolic process | 2/36 | 24/18866 | 0.000951632 | 0.006314206 | 0.003062008 | TNF/IL1B | 2 |
| GO:1902993 | positive regulation of amyloid precursor protein catabolic process | 2/36 | 24/18866 | 0.000951632 | 0.006314206 | 0.003062008 | TNF/CASP3 | 2 |
| GO:0000079 | regulation of cyclin-dependent protein serine/threonine kinase activity | 3/36 | 102/18866 | 0.000962337 | 0.00635091 | 0.003079807 | CDKN1A/CCNB1/CASP3 | 3 |
| GO:1903076 | regulation of protein localization to plasma membrane | 3/36 | 102/18866 | 0.000962337 | 0.00635091 | 0.003079807 | AR/TNF/PRKCE | 3 |
| GO:0007188 | adenylate cyclase-modulating G protein-coupled receptor signaling pathway | 4/36 | 233/18866 | 0.000979275 | 0.006428127 | 0.003117252 | PRKCA/OPRM1/CHRM4/CHRM3 | 4 |
| GO:2001234 | negative regulation of apoptotic signaling pathway | 4/36 | 233/18866 | 0.000979275 | 0.006428127 | 0.003117252 | AR/TNF/IL1B/BAX | 4 |
| GO:0097305 | response to alcohol | 4/36 | 234/18866 | 0.000994958 | 0.006513659 | 0.00315873 | F7/PRKCE/CDKN1A/OPRM1 | 4 |
| GO:0071453 | cellular response to oxygen levels | 4/36 | 235/18866 | 0.001010818 | 0.006582382 | 0.003192057 | TP53/PRKCE/MYC/CCNB1 | 4 |
| GO:0071695 | anatomical structure maturation | 4/36 | 235/18866 | 0.001010818 | 0.006582382 | 0.003192057 | CDKN1A/CCNB1/PGR/PDE3A | 4 |
| GO:0043279 | response to alkaloid | 3/36 | 104/18866 | 0.001017985 | 0.006611519 | 0.003206186 | PRKCE/CASP3/OPRM1 | 3 |
| GO:0050927 | positive regulation of positive chemotaxis | 2/36 | 25/18866 | 0.001033143 | 0.006692256 | 0.003245339 | KDR/F7 | 2 |
| GO:2001022 | positive regulation of response to DNA damage stimulus | 3/36 | 105/18866 | 0.001046558 | 0.006761314 | 0.003278828 | PRKCD/PCNA/MYC | 3 |
| GO:0071868 | cellular response to monoamine stimulus | 3/36 | 106/18866 | 0.001075634 | 0.006876772 | 0.003334818 | OPRM1/CHRM4/CHRM3 | 3 |
| GO:0071870 | cellular response to catecholamine stimulus | 3/36 | 106/18866 | 0.001075634 | 0.006876772 | 0.003334818 | OPRM1/CHRM4/CHRM3 | 3 |
| GO:1904029 | regulation of cyclin-dependent protein kinase activity | 3/36 | 106/18866 | 0.001075634 | 0.006876772 | 0.003334818 | CDKN1A/CCNB1/CASP3 | 3 |
| GO:1905269 | positive regulation of chromatin organization | 3/36 | 106/18866 | 0.001075634 | 0.006876772 | 0.003334818 | TP53/IL1B/CCNB1 | 3 |
| GO:0010639 | negative regulation of organelle organization | 5/36 | 416/18866 | 0.001092212 | 0.006964622 | 0.00337742 | CHEK1/TP53/PRKCD/CCNB1/MAP2 | 5 |
| GO:2001237 | negative regulation of extrinsic apoptotic signaling pathway | 3/36 | 107/18866 | 0.001105217 | 0.006983297 | 0.003386476 | AR/TNF/IL1B | 3 |
| GO:0006309 | apoptotic DNA fragmentation | 2/36 | 26/18866 | 0.001117896 | 0.006983297 | 0.003386476 | CASP3/BAX | 2 |
| GO:0034114 | regulation of heterotypic cell-cell adhesion | 2/36 | 26/18866 | 0.001117896 | 0.006983297 | 0.003386476 | TNF/IL1B | 2 |
| GO:0043951 | negative regulation of cAMP-mediated signaling | 2/36 | 26/18866 | 0.001117896 | 0.006983297 | 0.003386476 | PDE3A/OPRM1 | 2 |
| GO:0050926 | regulation of positive chemotaxis | 2/36 | 26/18866 | 0.001117896 | 0.006983297 | 0.003386476 | KDR/F7 | 2 |
| GO:0051123 | RNA polymerase II preinitiation complex assembly | 2/36 | 26/18866 | 0.001117896 | 0.006983297 | 0.003386476 | ESR1/TP53 | 2 |
| GO:0060307 | regulation of ventricular cardiac muscle cell membrane repolarization | 2/36 | 26/18866 | 0.001117896 | 0.006983297 | 0.003386476 | SCN5A/KCNH2 | 2 |
| GO:0060740 | prostate gland epithelium morphogenesis | 2/36 | 26/18866 | 0.001117896 | 0.006983297 | 0.003386476 | ESR1/AR | 2 |
| GO:0071887 | leukocyte apoptotic process | 3/36 | 108/18866 | 0.00113531 | 0.007074076 | 0.003430498 | TP53/CASP3/BAX | 3 |
| GO:0033002 | muscle cell proliferation | 4/36 | 244/18866 | 0.001161675 | 0.007220031 | 0.003501278 | JUN/TNF/CDKN1A/CCNB1 | 4 |
| GO:0043618 | regulation of transcription from RNA polymerase II promoter in response to stress | 3/36 | 109/18866 | 0.001165917 | 0.007228097 | 0.003505189 | CHEK1/JUN/TP53 | 3 |
| GO:0071867 | response to monoamine | 3/36 | 110/18866 | 0.001197042 | 0.007383762 | 0.003580677 | OPRM1/CHRM4/CHRM3 | 3 |
| GO:0071869 | response to catecholamine | 3/36 | 110/18866 | 0.001197042 | 0.007383762 | 0.003580677 | OPRM1/CHRM4/CHRM3 | 3 |
| GO:0001556 | oocyte maturation | 2/36 | 27/18866 | 0.001205881 | 0.007401097 | 0.003589084 | CCNB1/PDE3A | 2 |
| GO:0048143 | astrocyte activation | 2/36 | 27/18866 | 0.001205881 | 0.007401097 | 0.003589084 | TNF/IL1B | 2 |
| GO:0002526 | acute inflammatory response | 3/36 | 111/18866 | 0.001228687 | 0.007522259 | 0.00364784 | TNF/IL1B/OPRM1 | 3 |
| GO:0000280 | nuclear division | 5/36 | 428/18866 | 0.001239375 | 0.00755004 | 0.003661312 | CHEK1/PRKCA/IL1B/CCNB1/PDE3A | 5 |
| GO:0045785 | positive regulation of cell adhesion | 5/36 | 428/18866 | 0.001239375 | 0.00755004 | 0.003661312 | KDR/TNF/PRKCE/PRKCA/IL1B | 5 |
| GO:0006275 | regulation of DNA replication | 3/36 | 112/18866 | 0.001260856 | 0.007661883 | 0.003715549 | JUN/TP53/PCNA | 3 |
| GO:0001938 | positive regulation of endothelial cell proliferation | 3/36 | 113/18866 | 0.001293552 | 0.007785683 | 0.003775585 | JUN/KDR/PRKCA | 3 |
| GO:0033598 | mammary gland epithelial cell proliferation | 2/36 | 28/18866 | 0.001297085 | 0.007785683 | 0.003775585 | ESR1/BAX | 2 |
| GO:0060512 | prostate gland morphogenesis | 2/36 | 28/18866 | 0.001297085 | 0.007785683 | 0.003775585 | ESR1/AR | 2 |
| GO:0090200 | positive regulation of release of cytochrome c from mitochondria | 2/36 | 28/18866 | 0.001297085 | 0.007785683 | 0.003775585 | TP53/BAX | 2 |
| GO:1902932 | positive regulation of alcohol biosynthetic process | 2/36 | 28/18866 | 0.001297085 | 0.007785683 | 0.003775585 | TNF/IL1B | 2 |
| GO:0043122 | regulation of I-kappaB kinase/NF-kappaB signaling | 4/36 | 252/18866 | 0.001308486 | 0.007834959 | 0.003799481 | ESR1/TNF/PRKCE/IL1B | 4 |
| GO:0034329 | cell junction assembly | 5/36 | 434/18866 | 0.001318252 | 0.00787423 | 0.003818525 | KDR/TNF/PRKCA/IL1B/GABRA1 | 5 |
| GO:0043620 | regulation of DNA-templated transcription in response to stress | 3/36 | 115/18866 | 0.001360539 | 0.008087468 | 0.003921932 | CHEK1/JUN/TP53 | 3 |
| GO:0048640 | negative regulation of developmental growth | 3/36 | 115/18866 | 0.001360539 | 0.008087468 | 0.003921932 | CDKN1A/SLC6A4/MAP2 | 3 |
| GO:0022407 | regulation of cell-cell adhesion | 5/36 | 439/18866 | 0.00138678 | 0.008211811 | 0.003982231 | TNF/PRKCD/PRKCA/IL1B/CASP3 | 5 |
| GO:0001782 | B cell homeostasis | 2/36 | 29/18866 | 0.001391497 | 0.008211811 | 0.003982231 | CASP3/BAX | 2 |
| GO:2000108 | positive regulation of leukocyte apoptotic process | 2/36 | 29/18866 | 0.001391497 | 0.008211811 | 0.003982231 | TP53/BAX | 2 |
| GO:0060964 | regulation of gene silencing by miRNA | 3/36 | 116/18866 | 0.001394837 | 0.008211811 | 0.003982231 | ESR1/TP53/TNF | 3 |
| GO:0010876 | lipid localization | 5/36 | 440/18866 | 0.001400797 | 0.008227168 | 0.003989678 | NCOA2/TNF/PRKCD/IL1B/PON1 | 5 |
| GO:0030072 | peptide hormone secretion | 4/36 | 257/18866 | 0.001406581 | 0.008241426 | 0.003996593 | TNF/PRKCE/PRKCA/IL1B | 4 |
| GO:0018107 | peptidyl-threonine phosphorylation | 3/36 | 117/18866 | 0.001429675 | 0.008356792 | 0.004052538 | CHEK1/PRKCD/PRKCA | 3 |
| GO:0048608 | reproductive structure development | 5/36 | 443/18866 | 0.001443477 | 0.008417428 | 0.004081943 | ESR1/AR/CASP3/BAX/PGR | 5 |
| GO:0001844 | protein insertion into mitochondrial membrane involved in apoptotic signaling pathway | 2/36 | 30/18866 | 0.001489103 | 0.008561471 | 0.004151795 | TP53/BAX | 2 |
| GO:0007271 | synaptic transmission, cholinergic | 2/36 | 30/18866 | 0.001489103 | 0.008561471 | 0.004151795 | CHRNA2/CHRM3 | 2 |
| GO:0034110 | regulation of homotypic cell-cell adhesion | 2/36 | 30/18866 | 0.001489103 | 0.008561471 | 0.004151795 | PRKCD/PRKCA | 2 |
| GO:0060055 | angiogenesis involved in wound healing | 2/36 | 30/18866 | 0.001489103 | 0.008561471 | 0.004151795 | KDR/TNF | 2 |
| GO:0071480 | cellular response to gamma radiation | 2/36 | 30/18866 | 0.001489103 | 0.008561471 | 0.004151795 | TP53/CDKN1A | 2 |
| GO:0099625 | ventricular cardiac muscle cell membrane repolarization | 2/36 | 30/18866 | 0.001489103 | 0.008561471 | 0.004151795 | SCN5A/KCNH2 | 2 |
| GO:0010822 | positive regulation of mitochondrion organization | 3/36 | 119/18866 | 0.001500985 | 0.008594645 | 0.004167882 | KDR/TP53/BAX | 3 |
| GO:0061458 | reproductive system development | 5/36 | 447/18866 | 0.001501875 | 0.008594645 | 0.004167882 | ESR1/AR/CASP3/BAX/PGR | 5 |
| GO:0007187 | G protein-coupled receptor signaling pathway, coupled to cyclic nucleotide second messenger | 4/36 | 263/18866 | 0.001530961 | 0.008717024 | 0.004227228 | PRKCA/OPRM1/CHRM4/CHRM3 | 4 |
| GO:0050730 | regulation of peptidyl-tyrosine phosphorylation | 4/36 | 263/18866 | 0.001530961 | 0.008717024 | 0.004227228 | TP53/TNF/PRKCE/PRKCD | 4 |
| GO:0060147 | regulation of posttranscriptional gene silencing | 3/36 | 120/18866 | 0.001537463 | 0.008717024 | 0.004227228 | ESR1/TP53/TNF | 3 |
| GO:0060966 | regulation of gene silencing by RNA | 3/36 | 120/18866 | 0.001537463 | 0.008717024 | 0.004227228 | ESR1/TP53/TNF | 3 |
| GO:0033157 | regulation of intracellular protein transport | 4/36 | 264/18866 | 0.001552413 | 0.008781507 | 0.004258499 | TP53/PRKCD/PKIA/IL1B | 4 |
| GO:0030522 | intracellular receptor signaling pathway | 4/36 | 265/18866 | 0.001574074 | 0.008830742 | 0.004282375 | ESR2/ESR1/AR/PGR | 4 |
| GO:0002675 | positive regulation of acute inflammatory response | 2/36 | 31/18866 | 0.001589893 | 0.008830742 | 0.004282375 | TNF/IL1B | 2 |
| GO:0014072 | response to isoquinoline alkaloid | 2/36 | 31/18866 | 0.001589893 | 0.008830742 | 0.004282375 | PRKCE/OPRM1 | 2 |
| GO:0032770 | positive regulation of monooxygenase activity | 2/36 | 31/18866 | 0.001589893 | 0.008830742 | 0.004282375 | TNF/IL1B | 2 |
| GO:0043278 | response to morphine | 2/36 | 31/18866 | 0.001589893 | 0.008830742 | 0.004282375 | PRKCE/OPRM1 | 2 |
| GO:0045940 | positive regulation of steroid metabolic process | 2/36 | 31/18866 | 0.001589893 | 0.008830742 | 0.004282375 | TNF/IL1B | 2 |
| GO:0060261 | positive regulation of transcription initiation from RNA polymerase II promoter | 2/36 | 31/18866 | 0.001589893 | 0.008830742 | 0.004282375 | ESR1/TP53 | 2 |
| GO:0099623 | regulation of cardiac muscle cell membrane repolarization | 2/36 | 31/18866 | 0.001589893 | 0.008830742 | 0.004282375 | SCN5A/KCNH2 | 2 |
| GO:0046883 | regulation of hormone secretion | 4/36 | 267/18866 | 0.001618029 | 0.00896673 | 0.004348321 | TNF/PRKCE/PRKCA/IL1B | 4 |
| GO:1904375 | regulation of protein localization to cell periphery | 3/36 | 123/18866 | 0.001650227 | 0.009124566 | 0.004424862 | AR/TNF/PRKCE | 3 |
| GO:1901889 | negative regulation of cell junction assembly | 2/36 | 32/18866 | 0.001693855 | 0.00934475 | 0.004531638 | TNF/IL1B | 2 |
| GO:0008637 | apoptotic mitochondrial changes | 3/36 | 125/18866 | 0.001728209 | 0.009470433 | 0.004592586 | JUN/TP53/BAX | 3 |
| GO:0043500 | muscle adaptation | 3/36 | 125/18866 | 0.001728209 | 0.009470433 | 0.004592586 | SCN5A/PRKCA/IL1B | 3 |
| GO:1903578 | regulation of ATP metabolic process | 3/36 | 125/18866 | 0.001728209 | 0.009470433 | 0.004592586 | TP53/EIF6/CCNB1 | 3 |
| GO:0009306 | protein secretion | 5/36 | 462/18866 | 0.001736542 | 0.0094949 | 0.004604451 | TNF/PRKCE/PRKCA/IL1B/OPRM1 | 5 |
| GO:0035592 | establishment of protein localization to extracellular region | 5/36 | 463/18866 | 0.001753093 | 0.009553427 | 0.004632833 | TNF/PRKCE/PRKCA/IL1B/OPRM1 | 5 |
| GO:0006260 | DNA replication | 4/36 | 273/18866 | 0.001755029 | 0.009553427 | 0.004632833 | CHEK1/JUN/TP53/PCNA | 4 |
| GO:0018210 | peptidyl-threonine modification | 3/36 | 126/18866 | 0.001768051 | 0.009603022 | 0.004656884 | CHEK1/PRKCD/PRKCA | 3 |
| GO:0045736 | negative regulation of cyclin-dependent protein serine/threonine kinase activity | 2/36 | 33/18866 | 0.001800977 | 0.009717357 | 0.00471233 | CDKN1A/CASP3 | 2 |
| GO:0046627 | negative regulation of insulin receptor signaling pathway | 2/36 | 33/18866 | 0.001800977 | 0.009717357 | 0.00471233 | PRKCD/IL1B | 2 |
| GO:1902003 | regulation of amyloid-beta formation | 2/36 | 33/18866 | 0.001800977 | 0.009717357 | 0.00471233 | TNF/CASP3 | 2 |
| GO:1905477 | positive regulation of protein localization to membrane | 3/36 | 127/18866 | 0.001808465 | 0.009736362 | 0.004721546 | TP53/TNF/PRKCE | 3 |
| GO:0003012 | muscle system process | 5/36 | 467/18866 | 0.001820462 | 0.009779507 | 0.004742469 | SCN5A/PRKCA/IL1B/KCNH2/CHRM3 | 5 |
| GO:0034504 | protein localization to nucleus | 4/36 | 277/18866 | 0.001850728 | 0.009920387 | 0.004810787 | TP53/PRKCD/PKIA/CDKN1A | 4 |
| GO:0071692 | protein localization to extracellular region | 5/36 | 470/18866 | 0.001872228 | 0.010013767 | 0.004856071 | TNF/PRKCE/PRKCA/IL1B/OPRM1 | 5 |
| GO:0000737 | DNA catabolic process, endonucleolytic | 2/36 | 34/18866 | 0.001911246 | 0.010156081 | 0.004925084 | CASP3/BAX | 2 |
| GO:0098664 | G protein-coupled serotonin receptor signaling pathway | 2/36 | 34/18866 | 0.001911246 | 0.010156081 | 0.004925084 | CHRM4/CHRM3 | 2 |
| GO:1904030 | negative regulation of cyclin-dependent protein kinase activity | 2/36 | 34/18866 | 0.001911246 | 0.010156081 | 0.004925084 | CDKN1A/CASP3 | 2 |
| GO:0006766 | vitamin metabolic process | 3/36 | 131/18866 | 0.001975891 | 0.010454337 | 0.00506972 | PRSS1/TNF/IL1B | 3 |
| GO:0019218 | regulation of steroid metabolic process | 3/36 | 131/18866 | 0.001975891 | 0.010454337 | 0.00506972 | TNF/IL1B/FASN | 3 |
| GO:0016579 | protein deubiquitination | 4/36 | 283/18866 | 0.002001 | 0.010553124 | 0.005117626 | ESR1/AR/TP53/MYC | 4 |
| GO:0030262 | apoptotic nuclear changes | 2/36 | 35/18866 | 0.002024652 | 0.010553124 | 0.005117626 | CASP3/BAX | 2 |
| GO:0060251 | regulation of glial cell proliferation | 2/36 | 35/18866 | 0.002024652 | 0.010553124 | 0.005117626 | TNF/IL1B | 2 |
| GO:0071312 | cellular response to alkaloid | 2/36 | 35/18866 | 0.002024652 | 0.010553124 | 0.005117626 | CASP3/OPRM1 | 2 |
| GO:1900077 | negative regulation of cellular response to insulin stimulus | 2/36 | 35/18866 | 0.002024652 | 0.010553124 | 0.005117626 | PRKCD/IL1B | 2 |
| GO:1901030 | positive regulation of mitochondrial outer membrane permeabilization involved in apoptotic signaling pathway | 2/36 | 35/18866 | 0.002024652 | 0.010553124 | 0.005117626 | TP53/BAX | 2 |
| GO:1904031 | positive regulation of cyclin-dependent protein kinase activity | 2/36 | 35/18866 | 0.002024652 | 0.010553124 | 0.005117626 | CDKN1A/CCNB1 | 2 |
| GO:0001667 | ameboidal-type cell migration | 5/36 | 481/18866 | 0.002071347 | 0.010768978 | 0.005222302 | JUN/KDR/TNF/PRKCE/PRKCA | 5 |
| GO:0007249 | I-kappaB kinase/NF-kappaB signaling | 4/36 | 286/18866 | 0.002079224 | 0.010768978 | 0.005222302 | ESR1/TNF/PRKCE/IL1B | 4 |
| GO:0140014 | mitotic nuclear division | 4/36 | 286/18866 | 0.002079224 | 0.010768978 | 0.005222302 | CHEK1/PRKCA/IL1B/CCNB1 | 4 |
| GO:0021700 | developmental maturation | 4/36 | 287/18866 | 0.002105764 | 0.010847309 | 0.005260288 | CDKN1A/CCNB1/PGR/PDE3A | 4 |
| GO:0043280 | positive regulation of cysteine-type endopeptidase activity involved in apoptotic process | 3/36 | 134/18866 | 0.002107604 | 0.010847309 | 0.005260288 | TNF/MYC/BAX | 3 |
| GO:0046683 | response to organophosphorus | 3/36 | 134/18866 | 0.002107604 | 0.010847309 | 0.005260288 | IL1B/SLC6A4/PDE3A | 3 |
| GO:0010543 | regulation of platelet activation | 2/36 | 36/18866 | 0.002141183 | 0.010928492 | 0.005299657 | PRKCD/PRKCA | 2 |
| GO:0031572 | G2 DNA damage checkpoint | 2/36 | 36/18866 | 0.002141183 | 0.010928492 | 0.005299657 | CHEK1/CDKN1A | 2 |
| GO:1901099 | negative regulation of signal transduction in absence of ligand | 2/36 | 36/18866 | 0.002141183 | 0.010928492 | 0.005299657 | TNF/IL1B | 2 |
| GO:2001240 | negative regulation of extrinsic apoptotic signaling pathway in absence of ligand | 2/36 | 36/18866 | 0.002141183 | 0.010928492 | 0.005299657 | TNF/IL1B | 2 |
| GO:0032368 | regulation of lipid transport | 3/36 | 135/18866 | 0.002152693 | 0.010941742 | 0.005306082 | PRKCD/IL1B/PON1 | 3 |
| GO:0048565 | digestive tract development | 3/36 | 135/18866 | 0.002152693 | 0.010941742 | 0.005306082 | TNF/CDKN1A/CCNB1 | 3 |
| GO:0006921 | cellular component disassembly involved in execution phase of apoptosis | 2/36 | 37/18866 | 0.002260827 | 0.011350368 | 0.005504241 | CASP3/BAX | 2 |
| GO:0032941 | secretion by tissue | 2/36 | 37/18866 | 0.002260827 | 0.011350368 | 0.005504241 | PRKCE/CHRM3 | 2 |
| GO:0060260 | regulation of transcription initiation from RNA polymerase II promoter | 2/36 | 37/18866 | 0.002260827 | 0.011350368 | 0.005504241 | ESR1/TP53 | 2 |
| GO:0070897 | transcription preinitiation complex assembly | 2/36 | 37/18866 | 0.002260827 | 0.011350368 | 0.005504241 | ESR1/TP53 | 2 |
| GO:0086010 | membrane depolarization during action potential | 2/36 | 37/18866 | 0.002260827 | 0.011350368 | 0.005504241 | SCN5A/KCNH2 | 2 |
| GO:0090322 | regulation of superoxide metabolic process | 2/36 | 37/18866 | 0.002260827 | 0.011350368 | 0.005504241 | TNF/PRKCD | 2 |
| GO:0005996 | monosaccharide metabolic process | 4/36 | 296/18866 | 0.002355285 | 0.011776424 | 0.005710853 | NCOA2/TP53/TNF/PRKCE | 4 |
| GO:0031647 | regulation of protein stability | 4/36 | 296/18866 | 0.002355285 | 0.011776424 | 0.005710853 | TP53/PRKCD/CDKN1A/CASP3 | 4 |
| GO:0043029 | T cell homeostasis | 2/36 | 38/18866 | 0.002383572 | 0.011815468 | 0.005729786 | CASP3/BAX | 2 |
| GO:0060306 | regulation of membrane repolarization | 2/36 | 38/18866 | 0.002383572 | 0.011815468 | 0.005729786 | SCN5A/KCNH2 | 2 |
| GO:0071392 | cellular response to estradiol stimulus | 2/36 | 38/18866 | 0.002383572 | 0.011815468 | 0.005729786 | ESR2/ESR1 | 2 |
| GO:0086005 | ventricular cardiac muscle cell action potential | 2/36 | 38/18866 | 0.002383572 | 0.011815468 | 0.005729786 | SCN5A/KCNH2 | 2 |
| GO:0060968 | regulation of gene silencing | 3/36 | 140/18866 | 0.002387158 | 0.011815468 | 0.005729786 | ESR1/TP53/TNF | 3 |
| GO:0008584 | male gonad development | 3/36 | 141/18866 | 0.002435873 | 0.012032331 | 0.005834952 | ESR1/AR/BAX | 3 |
| GO:0070646 | protein modification by small protein removal | 4/36 | 300/18866 | 0.002472475 | 0.012188606 | 0.005910736 | ESR1/AR/TP53/MYC | 4 |
| GO:0007292 | female gamete generation | 3/36 | 142/18866 | 0.002485203 | 0.012199197 | 0.005915872 | CCNB1/PGR/PDE3A | 3 |
| GO:0046546 | development of primary male sexual characteristics | 3/36 | 142/18866 | 0.002485203 | 0.012199197 | 0.005915872 | ESR1/AR/BAX | 3 |
| GO:0007210 | serotonin receptor signaling pathway | 2/36 | 39/18866 | 0.002509407 | 0.012199197 | 0.005915872 | CHRM4/CHRM3 | 2 |
| GO:0033146 | regulation of intracellular estrogen receptor signaling pathway | 2/36 | 39/18866 | 0.002509407 | 0.012199197 | 0.005915872 | ESR1/AR | 2 |
| GO:0034205 | amyloid-beta formation | 2/36 | 39/18866 | 0.002509407 | 0.012199197 | 0.005915872 | TNF/CASP3 | 2 |
| GO:0086091 | regulation of heart rate by cardiac conduction | 2/36 | 39/18866 | 0.002509407 | 0.012199197 | 0.005915872 | SCN5A/KCNH2 | 2 |
| GO:2000279 | negative regulation of DNA biosynthetic process | 2/36 | 39/18866 | 0.002509407 | 0.012199197 | 0.005915872 | TP53/CDKN1A | 2 |
| GO:0050890 | cognition | 4/36 | 302/18866 | 0.002532554 | 0.01228739 | 0.00595864 | JUN/TNF/CASP3/SLC6A4 | 4 |
| GO:0060078 | regulation of postsynaptic membrane potential | 3/36 | 144/18866 | 0.002585713 | 0.012520565 | 0.006071716 | OPRM1/GABRA1/CHRNA2 | 3 |
| GO:0045740 | positive regulation of DNA replication | 2/36 | 40/18866 | 0.00263832 | 0.012725101 | 0.006170903 | JUN/PCNA | 2 |
| GO:1902991 | regulation of amyloid precursor protein catabolic process | 2/36 | 40/18866 | 0.00263832 | 0.012725101 | 0.006170903 | TNF/CASP3 | 2 |
| GO:0007043 | cell-cell junction assembly | 3/36 | 147/18866 | 0.00274115 | 0.013169321 | 0.006386323 | TNF/PRKCA/IL1B | 3 |
| GO:0055123 | digestive system development | 3/36 | 147/18866 | 0.00274115 | 0.013169321 | 0.006386323 | TNF/CDKN1A/CCNB1 | 3 |
| GO:0046825 | regulation of protein export from nucleus | 2/36 | 41/18866 | 0.0027703 | 0.013231687 | 0.006416567 | TP53/IL1B | 2 |
| GO:0099622 | cardiac muscle cell membrane repolarization | 2/36 | 41/18866 | 0.0027703 | 0.013231687 | 0.006416567 | SCN5A/KCNH2 | 2 |
| GO:1900117 | regulation of execution phase of apoptosis | 2/36 | 41/18866 | 0.0027703 | 0.013231687 | 0.006416567 | TP53/BAX | 2 |
| GO:0010675 | regulation of cellular carbohydrate metabolic process | 3/36 | 148/18866 | 0.002794218 | 0.013320009 | 0.006459397 | NCOA2/TP53/PRKCE | 3 |
| GO:0060326 | cell chemotaxis | 4/36 | 311/18866 | 0.002815412 | 0.013395032 | 0.006495779 | KDR/F7/PRKCD/IL1B | 4 |
| GO:0014074 | response to purine-containing compound | 3/36 | 149/18866 | 0.002847918 | 0.013523477 | 0.006558068 | IL1B/SLC6A4/PDE3A | 3 |
| GO:1903532 | positive regulation of secretion by cell | 4/36 | 313/18866 | 0.002881098 | 0.013654624 | 0.006621666 | TNF/PRKCE/IL1B/SLC6A4 | 4 |
| GO:0006509 | membrane protein ectodomain proteolysis | 2/36 | 42/18866 | 0.002905335 | 0.013742964 | 0.006664505 | TNF/IL1B | 2 |
| GO:0046879 | hormone secretion | 4/36 | 314/18866 | 0.002914333 | 0.013759014 | 0.006672288 | TNF/PRKCE/PRKCA/IL1B | 4 |
| GO:0051224 | negative regulation of protein transport | 3/36 | 151/18866 | 0.002957224 | 0.013908017 | 0.006744546 | PKIA/IL1B/OPRM1 | 3 |
| GO:2001056 | positive regulation of cysteine-type endopeptidase activity | 3/36 | 151/18866 | 0.002957224 | 0.013908017 | 0.006744546 | TNF/MYC/BAX | 3 |
| GO:0006308 | DNA catabolic process | 2/36 | 43/18866 | 0.003043414 | 0.014258744 | 0.006914627 | CASP3/BAX | 2 |
| GO:0150077 | regulation of neuroinflammatory response | 2/36 | 43/18866 | 0.003043414 | 0.014258744 | 0.006914627 | TNF/IL1B | 2 |
| GO:0062013 | positive regulation of small molecule metabolic process | 3/36 | 154/18866 | 0.003125987 | 0.01461771 | 0.007088704 | TNF/PRKCE/IL1B | 3 |
| GO:1904950 | negative regulation of establishment of protein localization | 3/36 | 155/18866 | 0.003183532 | 0.0148585 | 0.007205473 | PKIA/IL1B/OPRM1 | 3 |
| GO:0009914 | hormone transport | 4/36 | 323/18866 | 0.003225394 | 0.015025317 | 0.007286369 | TNF/PRKCE/PRKCA/IL1B | 4 |
| GO:0030902 | hindbrain development | 3/36 | 156/18866 | 0.003241726 | 0.0150728 | 0.007309395 | SCN5A/TP53/SLC6A4 | 3 |
| GO:0051235 | maintenance of location | 4/36 | 324/18866 | 0.003261303 | 0.015135157 | 0.007339635 | TNF/PRKCE/IL1B/BAX | 4 |
| GO:0006775 | fat-soluble vitamin metabolic process | 2/36 | 45/18866 | 0.003328657 | 0.015189318 | 0.007365899 | TNF/IL1B | 2 |
| GO:0014002 | astrocyte development | 2/36 | 45/18866 | 0.003328657 | 0.015189318 | 0.007365899 | TNF/IL1B | 2 |
| GO:0032722 | positive regulation of chemokine production | 2/36 | 45/18866 | 0.003328657 | 0.015189318 | 0.007365899 | TNF/IL1B | 2 |
| GO:0034105 | positive regulation of tissue remodeling | 2/36 | 45/18866 | 0.003328657 | 0.015189318 | 0.007365899 | PRKCA/BAX | 2 |
| GO:0042771 | intrinsic apoptotic signaling pathway in response to DNA damage by p53 class mediator | 2/36 | 45/18866 | 0.003328657 | 0.015189318 | 0.007365899 | TP53/CDKN1A | 2 |
| GO:0045429 | positive regulation of nitric oxide biosynthetic process | 2/36 | 45/18866 | 0.003328657 | 0.015189318 | 0.007365899 | TNF/IL1B | 2 |
| GO:0051204 | protein insertion into mitochondrial membrane | 2/36 | 45/18866 | 0.003328657 | 0.015189318 | 0.007365899 | TP53/BAX | 2 |
| GO:0070266 | necroptotic process | 2/36 | 45/18866 | 0.003328657 | 0.015189318 | 0.007365899 | TP53/TNF | 2 |
| GO:1901028 | regulation of mitochondrial outer membrane permeabilization involved in apoptotic signaling pathway | 2/36 | 45/18866 | 0.003328657 | 0.015189318 | 0.007365899 | TP53/BAX | 2 |
| GO:0030168 | platelet activation | 3/36 | 158/18866 | 0.003360073 | 0.015304229 | 0.007421624 | PRKCE/PRKCD/PRKCA | 3 |
| GO:0051092 | positive regulation of NF-kappaB transcription factor activity | 3/36 | 159/18866 | 0.00342023 | 0.01554938 | 0.007540507 | AR/TNF/IL1B | 3 |
| GO:1904407 | positive regulation of nitric oxide metabolic process | 2/36 | 46/18866 | 0.003475798 | 0.0157728 | 0.007648853 | TNF/IL1B | 2 |
| GO:0001655 | urogenital system development | 4/36 | 330/18866 | 0.003482525 | 0.015774166 | 0.007649515 | ESR1/AR/MYC/BAX | 4 |
| GO:0043467 | regulation of generation of precursor metabolites and energy | 3/36 | 162/18866 | 0.003604663 | 0.016267369 | 0.007888689 | TP53/EIF6/CCNB1 | 3 |
| GO:2001236 | regulation of extrinsic apoptotic signaling pathway | 3/36 | 162/18866 | 0.003604663 | 0.016267369 | 0.007888689 | AR/TNF/IL1B | 3 |
| GO:0006953 | acute-phase response | 2/36 | 47/18866 | 0.003625938 | 0.016273632 | 0.007891726 | TNF/IL1B | 2 |
| GO:0046677 | response to antibiotic | 2/36 | 47/18866 | 0.003625938 | 0.016273632 | 0.007891726 | TP53/CASP3 | 2 |
| GO:0048599 | oocyte development | 2/36 | 47/18866 | 0.003625938 | 0.016273632 | 0.007891726 | CCNB1/PDE3A | 2 |
| GO:0006665 | sphingolipid metabolic process | 3/36 | 163/18866 | 0.00366747 | 0.016370251 | 0.00793858 | TNF/PRKCD/BAX | 3 |
| GO:0007088 | regulation of mitotic nuclear division | 3/36 | 163/18866 | 0.00366747 | 0.016370251 | 0.00793858 | CHEK1/IL1B/CCNB1 | 3 |
| GO:0009267 | cellular response to starvation | 3/36 | 163/18866 | 0.00366747 | 0.016370251 | 0.00793858 | JUN/TP53/CDKN1A | 3 |
| GO:0043535 | regulation of blood vessel endothelial cell migration | 3/36 | 164/18866 | 0.003730944 | 0.016563232 | 0.008032164 | KDR/TNF/PRKCA | 3 |
| GO:0046165 | alcohol biosynthetic process | 3/36 | 164/18866 | 0.003730944 | 0.016563232 | 0.008032164 | TNF/IL1B/FASN | 3 |
| GO:0046661 | male sex differentiation | 3/36 | 164/18866 | 0.003730944 | 0.016563232 | 0.008032164 | ESR1/AR/BAX | 3 |
| GO:0045601 | regulation of endothelial cell differentiation | 2/36 | 48/18866 | 0.003779064 | 0.016656376 | 0.008077333 | TNF/IL1B | 2 |
| GO:0051932 | synaptic transmission, GABAergic | 2/36 | 48/18866 | 0.003779064 | 0.016656376 | 0.008077333 | PRKCE/GABRA1 | 2 |
| GO:0051972 | regulation of telomerase activity | 2/36 | 48/18866 | 0.003779064 | 0.016656376 | 0.008077333 | TP53/MYC | 2 |
| GO:2001239 | regulation of extrinsic apoptotic signaling pathway in absence of ligand | 2/36 | 48/18866 | 0.003779064 | 0.016656376 | 0.008077333 | TNF/IL1B | 2 |
| GO:0051047 | positive regulation of secretion | 4/36 | 340/18866 | 0.003873669 | 0.017042755 | 0.008264704 | TNF/PRKCE/IL1B/SLC6A4 | 4 |
| GO:0002673 | regulation of acute inflammatory response | 2/36 | 49/18866 | 0.003935165 | 0.017068605 | 0.00827724 | TNF/IL1B | 2 |
| GO:0003254 | regulation of membrane depolarization | 2/36 | 49/18866 | 0.003935165 | 0.017068605 | 0.00827724 | SCN5A/KDR | 2 |
| GO:0030850 | prostate gland development | 2/36 | 49/18866 | 0.003935165 | 0.017068605 | 0.00827724 | ESR1/AR | 2 |
| GO:0048512 | circadian behavior | 2/36 | 49/18866 | 0.003935165 | 0.017068605 | 0.00827724 | NCOA2/TP53 | 2 |
| GO:0070231 | T cell apoptotic process | 2/36 | 49/18866 | 0.003935165 | 0.017068605 | 0.00827724 | TP53/BAX | 2 |
| GO:0086009 | membrane repolarization | 2/36 | 49/18866 | 0.003935165 | 0.017068605 | 0.00827724 | SCN5A/KCNH2 | 2 |
| GO:0090151 | establishment of protein localization to mitochondrial membrane | 2/36 | 49/18866 | 0.003935165 | 0.017068605 | 0.00827724 | TP53/BAX | 2 |
| GO:0090199 | regulation of release of cytochrome c from mitochondria | 2/36 | 49/18866 | 0.003935165 | 0.017068605 | 0.00827724 | TP53/BAX | 2 |
| GO:0045931 | positive regulation of mitotic cell cycle | 3/36 | 168/18866 | 0.003991565 | 0.017243707 | 0.008362154 | PRKCA/IL1B/CCNB1 | 3 |
| GO:0007596 | blood coagulation | 4/36 | 343/18866 | 0.003996607 | 0.017243707 | 0.008362154 | F7/PRKCE/PRKCD/PRKCA | 4 |
| GO:0016042 | lipid catabolic process | 4/36 | 343/18866 | 0.003996607 | 0.017243707 | 0.008362154 | TNF/PRKCE/PRKCD/IL1B | 4 |
| GO:0001774 | microglial cell activation | 2/36 | 50/18866 | 0.004094231 | 0.017480586 | 0.008477025 | JUN/TNF | 2 |
| GO:0002269 | leukocyte activation involved in inflammatory response | 2/36 | 50/18866 | 0.004094231 | 0.017480586 | 0.008477025 | JUN/TNF | 2 |
| GO:0007622 | rhythmic behavior | 2/36 | 50/18866 | 0.004094231 | 0.017480586 | 0.008477025 | NCOA2/TP53 | 2 |
| GO:0014009 | glial cell proliferation | 2/36 | 50/18866 | 0.004094231 | 0.017480586 | 0.008477025 | TNF/IL1B | 2 |
| GO:0042987 | amyloid precursor protein catabolic process | 2/36 | 50/18866 | 0.004094231 | 0.017480586 | 0.008477025 | TNF/CASP3 | 2 |
| GO:1904707 | positive regulation of vascular associated smooth muscle cell proliferation | 2/36 | 50/18866 | 0.004094231 | 0.017480586 | 0.008477025 | JUN/TNF | 2 |
| GO:2000241 | regulation of reproductive process | 3/36 | 170/18866 | 0.004125941 | 0.017585393 | 0.008527851 | ESR1/AR/PDE3A | 3 |
| GO:0051302 | regulation of cell division | 3/36 | 171/18866 | 0.004194153 | 0.017845142 | 0.008653813 | PRKCE/MYC/IL1B | 3 |
| GO:0007599 | hemostasis | 4/36 | 348/18866 | 0.00420736 | 0.017870363 | 0.008666044 | F7/PRKCE/PRKCD/PRKCA | 4 |
| GO:0050817 | coagulation | 4/36 | 349/18866 | 0.004250399 | 0.018015677 | 0.008736512 | F7/PRKCE/PRKCD/PRKCA | 4 |
| GO:0050435 | amyloid-beta metabolic process | 2/36 | 51/18866 | 0.00425625 | 0.018015677 | 0.008736512 | TNF/CASP3 | 2 |
| GO:0006633 | fatty acid biosynthetic process | 3/36 | 173/18866 | 0.004332636 | 0.01827598 | 0.008862744 | IL1B/FASN/EIF6 | 3 |
| GO:0048660 | regulation of smooth muscle cell proliferation | 3/36 | 173/18866 | 0.004332636 | 0.01827598 | 0.008862744 | JUN/TNF/CDKN1A | 3 |
| GO:0010469 | regulation of signaling receptor activity | 3/36 | 174/18866 | 0.00440291 | 0.018459303 | 0.008951644 | ESR2/PRKCD/OPRM1 | 3 |
| GO:0035725 | sodium ion transmembrane transport | 3/36 | 174/18866 | 0.00440291 | 0.018459303 | 0.008951644 | SCN5A/PRKCE/SLC6A4 | 3 |
| GO:0006998 | nuclear envelope organization | 2/36 | 52/18866 | 0.00442121 | 0.018459303 | 0.008951644 | PRKCA/CCNB1 | 2 |
| GO:0009994 | oocyte differentiation | 2/36 | 52/18866 | 0.00442121 | 0.018459303 | 0.008951644 | CCNB1/PDE3A | 2 |
| GO:0045839 | negative regulation of mitotic nuclear division | 2/36 | 52/18866 | 0.00442121 | 0.018459303 | 0.008951644 | CHEK1/CCNB1 | 2 |
| GO:0050999 | regulation of nitric-oxide synthase activity | 2/36 | 52/18866 | 0.00442121 | 0.018459303 | 0.008951644 | TNF/IL1B | 2 |
| GO:0048659 | smooth muscle cell proliferation | 3/36 | 175/18866 | 0.004473876 | 0.018615875 | 0.009027572 | JUN/TNF/CDKN1A | 3 |
| GO:1901568 | fatty acid derivative metabolic process | 3/36 | 175/18866 | 0.004473876 | 0.018615875 | 0.009027572 | IL1B/FASN/PON1 | 3 |
| GO:0016241 | regulation of macroautophagy | 3/36 | 176/18866 | 0.004545536 | 0.018882049 | 0.00915665 | KDR/TP53/CASP3 | 3 |
| GO:0048771 | tissue remodeling | 3/36 | 178/18866 | 0.004690945 | 0.019420353 | 0.009417695 | TP53/PRKCA/BAX | 3 |
| GO:0051897 | positive regulation of protein kinase B signaling | 3/36 | 178/18866 | 0.004690945 | 0.019420353 | 0.009417695 | ESR1/F7/TNF | 3 |
| GO:0032757 | positive regulation of interleukin-8 production | 2/36 | 54/18866 | 0.004759911 | 0.019672697 | 0.009540067 | TNF/IL1B | 2 |
| GO:0010950 | positive regulation of endopeptidase activity | 3/36 | 181/18866 | 0.00491431 | 0.020186769 | 0.00978936 | TNF/MYC/BAX | 3 |
| GO:1905330 | regulation of morphogenesis of an epithelium | 3/36 | 181/18866 | 0.00491431 | 0.020186769 | 0.00978936 | ESR1/AR/TNF | 3 |
| GO:0035065 | regulation of histone acetylation | 2/36 | 55/18866 | 0.00493363 | 0.020186769 | 0.00978936 | CHEK1/IL1B | 2 |
| GO:0042304 | regulation of fatty acid biosynthetic process | 2/36 | 55/18866 | 0.00493363 | 0.020186769 | 0.00978936 | IL1B/EIF6 | 2 |
| GO:0051353 | positive regulation of oxidoreductase activity | 2/36 | 55/18866 | 0.00493363 | 0.020186769 | 0.00978936 | TNF/IL1B | 2 |
| GO:0097345 | mitochondrial outer membrane permeabilization | 2/36 | 55/18866 | 0.00493363 | 0.020186769 | 0.00978936 | TP53/BAX | 2 |
| GO:0090316 | positive regulation of intracellular protein transport | 3/36 | 182/18866 | 0.004990174 | 0.020350293 | 0.00986866 | TP53/PRKCD/IL1B | 3 |
| GO:2001252 | positive regulation of chromosome organization | 3/36 | 182/18866 | 0.004990174 | 0.020350293 | 0.00986866 | TP53/IL1B/CCNB1 | 3 |
| GO:0070228 | regulation of lymphocyte apoptotic process | 2/36 | 56/18866 | 0.005110246 | 0.020770949 | 0.010072652 | TP53/BAX | 2 |
| GO:0086002 | cardiac muscle cell action potential involved in contraction | 2/36 | 56/18866 | 0.005110246 | 0.020770949 | 0.010072652 | SCN5A/KCNH2 | 2 |
| GO:0006611 | protein export from nucleus | 3/36 | 184/18866 | 0.005144025 | 0.020873689 | 0.010122475 | TP53/IL1B/EIF6 | 3 |
| GO:0045862 | positive regulation of proteolysis | 4/36 | 370/18866 | 0.005224569 | 0.021165541 | 0.010264005 | TNF/MYC/IL1B/BAX | 4 |
| GO:0001541 | ovarian follicle development | 2/36 | 57/18866 | 0.005289748 | 0.021359097 | 0.010357868 | ESR1/BAX | 2 |
| GO:0071398 | cellular response to fatty acid | 2/36 | 57/18866 | 0.005289748 | 0.021359097 | 0.010357868 | PRKCE/CCNB1 | 2 |
| GO:0043409 | negative regulation of MAPK cascade | 3/36 | 187/18866 | 0.005380141 | 0.021652864 | 0.010500327 | PRKCD/MYC/IL1B | 3 |
| GO:0050821 | protein stabilization | 3/36 | 187/18866 | 0.005380141 | 0.021652864 | 0.010500327 | TP53/PRKCD/CDKN1A | 3 |
| GO:0043123 | positive regulation of I-kappaB kinase/NF-kappaB signaling | 3/36 | 188/18866 | 0.005460278 | 0.021939414 | 0.010639287 | TNF/PRKCE/IL1B | 3 |
| GO:1903793 | positive regulation of anion transport | 2/36 | 58/18866 | 0.005472125 | 0.021951089 | 0.010644949 | PRKCD/IL1B | 2 |
| GO:0010565 | regulation of cellular ketone metabolic process | 3/36 | 189/18866 | 0.005541133 | 0.022155506 | 0.010744079 | PRKCE/IL1B/EIF6 | 3 |
| GO:0043534 | blood vessel endothelial cell migration | 3/36 | 189/18866 | 0.005541133 | 0.022155506 | 0.010744079 | KDR/TNF/PRKCA | 3 |
| GO:0003018 | vascular process in circulatory system | 3/36 | 190/18866 | 0.005622708 | 0.022445119 | 0.010884524 | SLC6A4/PDE3A/CHRM3 | 3 |
| GO:0042306 | regulation of protein import into nucleus | 2/36 | 59/18866 | 0.005657366 | 0.022546809 | 0.010933837 | PRKCD/PKIA | 2 |
| GO:0010821 | regulation of mitochondrion organization | 3/36 | 191/18866 | 0.005705006 | 0.02269982 | 0.011008038 | KDR/TP53/BAX | 3 |
| GO:0006909 | phagocytosis | 4/36 | 382/18866 | 0.005843694 | 0.023146139 | 0.011224475 | TNF/PRKCE/PRKCD/IL1B | 4 |
| GO:0051784 | negative regulation of nuclear division | 2/36 | 60/18866 | 0.005845461 | 0.023146139 | 0.011224475 | CHEK1/CCNB1 | 2 |
| GO:1903428 | positive regulation of reactive oxygen species biosynthetic process | 2/36 | 60/18866 | 0.005845461 | 0.023146139 | 0.011224475 | TNF/IL1B | 2 |
| GO:0071248 | cellular response to metal ion | 3/36 | 193/18866 | 0.005871774 | 0.023212892 | 0.011256847 | JUN/SCN5A/CCNB1 | 3 |
| GO:0006066 | alcohol metabolic process | 4/36 | 385/18866 | 0.006005825 | 0.023635336 | 0.011461706 | TNF/IL1B/FASN/PON1 | 4 |
| GO:0033619 | membrane protein proteolysis | 2/36 | 61/18866 | 0.006036397 | 0.023635336 | 0.011461706 | TNF/IL1B | 2 |
| GO:0090303 | positive regulation of wound healing | 2/36 | 61/18866 | 0.006036397 | 0.023635336 | 0.011461706 | F7/PRKCE | 2 |
| GO:1902110 | positive regulation of mitochondrial membrane permeability involved in apoptotic process | 2/36 | 61/18866 | 0.006036397 | 0.023635336 | 0.011461706 | TP53/BAX | 2 |
| GO:1903078 | positive regulation of protein localization to plasma membrane | 2/36 | 61/18866 | 0.006036397 | 0.023635336 | 0.011461706 | TNF/PRKCE | 2 |
| GO:2000756 | regulation of peptidyl-lysine acetylation | 2/36 | 61/18866 | 0.006036397 | 0.023635336 | 0.011461706 | CHEK1/IL1B | 2 |
| GO:0006694 | steroid biosynthetic process | 3/36 | 197/18866 | 0.006214053 | 0.024086702 | 0.011680592 | TNF/IL1B/FASN | 3 |
| GO:0002260 | lymphocyte homeostasis | 2/36 | 62/18866 | 0.006230165 | 0.024086702 | 0.011680592 | CASP3/BAX | 2 |
| GO:0030888 | regulation of B cell proliferation | 2/36 | 62/18866 | 0.006230165 | 0.024086702 | 0.011680592 | CDKN1A/CASP3 | 2 |
| GO:0034113 | heterotypic cell-cell adhesion | 2/36 | 62/18866 | 0.006230165 | 0.024086702 | 0.011680592 | TNF/IL1B | 2 |
| GO:0070527 | platelet aggregation | 2/36 | 62/18866 | 0.006230165 | 0.024086702 | 0.011680592 | PRKCD/PRKCA | 2 |
| GO:1904589 | regulation of protein import | 2/36 | 62/18866 | 0.006230165 | 0.024086702 | 0.011680592 | PRKCD/PKIA | 2 |
| GO:2000351 | regulation of endothelial cell apoptotic process | 2/36 | 62/18866 | 0.006230165 | 0.024086702 | 0.011680592 | KDR/TNF | 2 |
| GO:2001244 | positive regulation of intrinsic apoptotic signaling pathway | 2/36 | 62/18866 | 0.006230165 | 0.024086702 | 0.011680592 | TP53/BAX | 2 |
| GO:0007626 | locomotory behavior | 3/36 | 198/18866 | 0.006301454 | 0.02432401 | 0.011795672 | NCOA2/PRKCE/OPRM1 | 3 |
| GO:0019933 | cAMP-mediated signaling | 3/36 | 199/18866 | 0.006389591 | 0.024625504 | 0.011941878 | PRKCA/PDE3A/OPRM1 | 3 |
| GO:0070059 | intrinsic apoptotic signaling pathway in response to endoplasmic reticulum stress | 2/36 | 63/18866 | 0.006426754 | 0.024691205 | 0.011973739 | TP53/BAX | 2 |
| GO:1902686 | mitochondrial outer membrane permeabilization involved in programmed cell death | 2/36 | 63/18866 | 0.006426754 | 0.024691205 | 0.011973739 | TP53/BAX | 2 |
| GO:0006869 | lipid transport | 4/36 | 393/18866 | 0.006452854 | 0.024752746 | 0.012003583 | NCOA2/PRKCD/IL1B/PON1 | 4 |
| GO:0010389 | regulation of G2/M transition of mitotic cell cycle | 3/36 | 200/18866 | 0.006478465 | 0.02477357 | 0.012013681 | PKIA/CDKN1A/CCNB1 | 3 |
| GO:1901888 | regulation of cell junction assembly | 3/36 | 200/18866 | 0.006478465 | 0.02477357 | 0.012013681 | KDR/TNF/IL1B | 3 |
| GO:0010952 | positive regulation of peptidase activity | 3/36 | 201/18866 | 0.006568078 | 0.025077189 | 0.012160918 | TNF/MYC/BAX | 3 |
| GO:0006631 | fatty acid metabolic process | 4/36 | 396/18866 | 0.006626064 | 0.025103707 | 0.012173777 | IL1B/FASN/EIF6/PON1 | 4 |
| GO:0010959 | regulation of metal ion transport | 4/36 | 396/18866 | 0.006626064 | 0.025103707 | 0.012173777 | SCN5A/PRKCE/BAX/KCNH2 | 4 |
| GO:0002066 | columnar/cuboidal epithelial cell development | 2/36 | 64/18866 | 0.006626152 | 0.025103707 | 0.012173777 | FASN/CDKN1A | 2 |
| GO:0002437 | inflammatory response to antigenic stimulus | 2/36 | 64/18866 | 0.006626152 | 0.025103707 | 0.012173777 | TNF/OPRM1 | 2 |
| GO:0046686 | response to cadmium ion | 2/36 | 64/18866 | 0.006626152 | 0.025103707 | 0.012173777 | JUN/PCNA | 2 |
| GO:0006687 | glycosphingolipid metabolic process | 2/36 | 65/18866 | 0.006828348 | 0.025632408 | 0.012430166 | PRKCD/BAX | 2 |
| GO:0010573 | vascular endothelial growth factor production | 2/36 | 65/18866 | 0.006828348 | 0.025632408 | 0.012430166 | TNF/IL1B | 2 |
| GO:0032729 | positive regulation of interferon-gamma production | 2/36 | 65/18866 | 0.006828348 | 0.025632408 | 0.012430166 | TNF/IL1B | 2 |
| GO:0035794 | positive regulation of mitochondrial membrane permeability | 2/36 | 65/18866 | 0.006828348 | 0.025632408 | 0.012430166 | TP53/BAX | 2 |
| GO:0051205 | protein insertion into membrane | 2/36 | 65/18866 | 0.006828348 | 0.025632408 | 0.012430166 | TP53/BAX | 2 |
| GO:1902305 | regulation of sodium ion transmembrane transport | 2/36 | 65/18866 | 0.006828348 | 0.025632408 | 0.012430166 | SCN5A/PRKCE | 2 |
| GO:0051168 | nuclear export | 3/36 | 204/18866 | 0.006841368 | 0.025642072 | 0.012434852 | TP53/IL1B/EIF6 | 3 |
| GO:0042594 | response to starvation | 3/36 | 206/18866 | 0.007027284 | 0.02619496 | 0.012702969 | JUN/TP53/CDKN1A | 3 |
| GO:0032768 | regulation of monooxygenase activity | 2/36 | 66/18866 | 0.007033333 | 0.02619496 | 0.012702969 | TNF/IL1B | 2 |
| GO:0046626 | regulation of insulin receptor signaling pathway | 2/36 | 66/18866 | 0.007033333 | 0.02619496 | 0.012702969 | PRKCD/IL1B | 2 |
| GO:1902108 | regulation of mitochondrial membrane permeability involved in apoptotic process | 2/36 | 66/18866 | 0.007033333 | 0.02619496 | 0.012702969 | TP53/BAX | 2 |
| GO:0045765 | regulation of angiogenesis | 4/36 | 403/18866 | 0.00704223 | 0.02619496 | 0.012702969 | KDR/TNF/PRKCA/IL1B | 4 |
| GO:0045766 | positive regulation of angiogenesis | 3/36 | 208/18866 | 0.007216194 | 0.026772421 | 0.012983003 | KDR/PRKCA/IL1B | 3 |
| GO:0046513 | ceramide biosynthetic process | 2/36 | 67/18866 | 0.007241095 | 0.026772421 | 0.012983003 | TNF/PRKCD | 2 |
| GO:0050918 | positive chemotaxis | 2/36 | 67/18866 | 0.007241095 | 0.026772421 | 0.012983003 | KDR/F7 | 2 |
| GO:1905710 | positive regulation of membrane permeability | 2/36 | 67/18866 | 0.007241095 | 0.026772421 | 0.012983003 | TP53/BAX | 2 |
| GO:0045216 | cell-cell junction organization | 3/36 | 210/18866 | 0.00740811 | 0.02726339 | 0.013221093 | TNF/PRKCA/IL1B | 3 |
| GO:0032642 | regulation of chemokine production | 2/36 | 68/18866 | 0.007451623 | 0.02726339 | 0.013221093 | TNF/IL1B | 2 |
| GO:0045428 | regulation of nitric oxide biosynthetic process | 2/36 | 68/18866 | 0.007451623 | 0.02726339 | 0.013221093 | TNF/IL1B | 2 |
| GO:0050766 | positive regulation of phagocytosis | 2/36 | 68/18866 | 0.007451623 | 0.02726339 | 0.013221093 | TNF/IL1B | 2 |
| GO:0071479 | cellular response to ionizing radiation | 2/36 | 68/18866 | 0.007451623 | 0.02726339 | 0.013221093 | TP53/CDKN1A | 2 |
| GO:0072577 | endothelial cell apoptotic process | 2/36 | 68/18866 | 0.007451623 | 0.02726339 | 0.013221093 | KDR/TNF | 2 |
| GO:1904377 | positive regulation of protein localization to cell periphery | 2/36 | 68/18866 | 0.007451623 | 0.02726339 | 0.013221093 | TNF/PRKCE | 2 |
| GO:0006643 | membrane lipid metabolic process | 3/36 | 212/18866 | 0.007603042 | 0.027775995 | 0.013469675 | TNF/PRKCD/BAX | 3 |
| GO:2000573 | positive regulation of DNA biosynthetic process | 2/36 | 69/18866 | 0.007664907 | 0.027960397 | 0.013559099 | PCNA/MYC | 2 |
| GO:0006006 | glucose metabolic process | 3/36 | 214/18866 | 0.007801003 | 0.028414632 | 0.013779376 | NCOA2/TP53/TNF | 3 |
| GO:0043281 | regulation of cysteine-type endopeptidase activity involved in apoptotic process | 3/36 | 215/18866 | 0.007901122 | 0.028736675 | 0.013935547 | TNF/MYC/BAX | 3 |
| GO:0042982 | amyloid precursor protein metabolic process | 2/36 | 71/18866 | 0.0080997 | 0.02941533 | 0.014264653 | TNF/CASP3 | 2 |
| GO:0001558 | regulation of cell growth | 4/36 | 420/18866 | 0.008124495 | 0.029461795 | 0.014287186 | ESR2/TP53/CDKN1A/MAP2 | 4 |
| GO:0050670 | regulation of lymphocyte proliferation | 3/36 | 219/18866 | 0.008309227 | 0.030087245 | 0.014590492 | IL1B/CDKN1A/CASP3 | 3 |
| GO:0050727 | regulation of inflammatory response | 4/36 | 425/18866 | 0.008462554 | 0.030597305 | 0.01483784 | ESR1/TNF/PRKCD/IL1B | 4 |
| GO:0031669 | cellular response to nutrient levels | 3/36 | 221/18866 | 0.008517874 | 0.030661847 | 0.014869139 | JUN/TP53/CDKN1A | 3 |
| GO:0032944 | regulation of mononuclear cell proliferation | 3/36 | 221/18866 | 0.008517874 | 0.030661847 | 0.014869139 | IL1B/CDKN1A/CASP3 | 3 |
| GO:0071241 | cellular response to inorganic substance | 3/36 | 221/18866 | 0.008517874 | 0.030661847 | 0.014869139 | JUN/SCN5A/CCNB1 | 3 |
| GO:0070227 | lymphocyte apoptotic process | 2/36 | 73/18866 | 0.008545388 | 0.030715851 | 0.014895328 | TP53/BAX | 2 |
| GO:0019722 | calcium-mediated signaling | 3/36 | 222/18866 | 0.00862335 | 0.030950767 | 0.015009248 | KDR/TNF/CHRM3 | 3 |
| GO:0006801 | superoxide metabolic process | 2/36 | 74/18866 | 0.008772291 | 0.031302287 | 0.015179714 | TNF/PRKCD | 2 |
| GO:0051881 | regulation of mitochondrial membrane potential | 2/36 | 74/18866 | 0.008772291 | 0.031302287 | 0.015179714 | KDR/BAX | 2 |
| GO:0086003 | cardiac muscle cell contraction | 2/36 | 74/18866 | 0.008772291 | 0.031302287 | 0.015179714 | SCN5A/KCNH2 | 2 |
| GO:1903036 | positive regulation of response to wounding | 2/36 | 74/18866 | 0.008772291 | 0.031302287 | 0.015179714 | F7/PRKCE | 2 |
| GO:0019935 | cyclic-nucleotide-mediated signaling | 3/36 | 224/18866 | 0.008836616 | 0.031486056 | 0.01526883 | PRKCA/PDE3A/OPRM1 | 3 |
| GO:0032602 | chemokine production | 2/36 | 75/18866 | 0.009001886 | 0.031798029 | 0.015420118 | TNF/IL1B | 2 |
| GO:0033143 | regulation of intracellular steroid hormone receptor signaling pathway | 2/36 | 75/18866 | 0.009001886 | 0.031798029 | 0.015420118 | ESR1/AR | 2 |
| GO:0050805 | negative regulation of synaptic transmission | 2/36 | 75/18866 | 0.009001886 | 0.031798029 | 0.015420118 | IL1B/SLC6A4 | 2 |
| GO:0086001 | cardiac muscle cell action potential | 2/36 | 75/18866 | 0.009001886 | 0.031798029 | 0.015420118 | SCN5A/KCNH2 | 2 |
| GO:1900076 | regulation of cellular response to insulin stimulus | 2/36 | 75/18866 | 0.009001886 | 0.031798029 | 0.015420118 | PRKCD/IL1B | 2 |
| GO:1901983 | regulation of protein acetylation | 2/36 | 75/18866 | 0.009001886 | 0.031798029 | 0.015420118 | CHEK1/IL1B | 2 |
| GO:0033077 | T cell differentiation in thymus | 2/36 | 76/18866 | 0.009234163 | 0.032571652 | 0.015795279 | TP53/IL1B | 2 |
| GO:0014015 | positive regulation of gliogenesis | 2/36 | 77/18866 | 0.009469111 | 0.033304681 | 0.016150753 | TNF/IL1B | 2 |
| GO:0046902 | regulation of mitochondrial membrane permeability | 2/36 | 77/18866 | 0.009469111 | 0.033304681 | 0.016150753 | TP53/BAX | 2 |
| GO:0032271 | regulation of protein polymerization | 3/36 | 231/18866 | 0.009607459 | 0.033742934 | 0.016363279 | PRKCE/PRKCD/MAP2 | 3 |
| GO:0042310 | vasoconstriction | 2/36 | 78/18866 | 0.00970672 | 0.033994289 | 0.016485171 | SLC6A4/CHRM3 | 2 |
| GO:0061045 | negative regulation of wound healing | 2/36 | 78/18866 | 0.00970672 | 0.033994289 | 0.016485171 | TNF/PRKCD | 2 |
| GO:1901342 | regulation of vasculature development | 4/36 | 444/18866 | 0.009831563 | 0.034344553 | 0.016655028 | KDR/TNF/PRKCA/IL1B | 4 |
| GO:0009743 | response to carbohydrate | 3/36 | 233/18866 | 0.009834713 | 0.034344553 | 0.016655028 | PRKCE/IL1B/CASP3 | 3 |
| GO:0006809 | nitric oxide biosynthetic process | 2/36 | 79/18866 | 0.009946978 | 0.03439413 | 0.01667907 | TNF/IL1B | 2 |
| GO:0010827 | regulation of glucose transmembrane transport | 2/36 | 79/18866 | 0.009946978 | 0.03439413 | 0.01667907 | TNF/IL1B | 2 |
| GO:0032272 | negative regulation of protein polymerization | 2/36 | 79/18866 | 0.009946978 | 0.03439413 | 0.01667907 | PRKCD/MAP2 | 2 |
| GO:0042246 | tissue regeneration | 2/36 | 79/18866 | 0.009946978 | 0.03439413 | 0.01667907 | CDKN1A/CCNB1 | 2 |
| GO:0043407 | negative regulation of MAP kinase activity | 2/36 | 79/18866 | 0.009946978 | 0.03439413 | 0.01667907 | PRKCD/IL1B | 2 |
| GO:0043536 | positive regulation of blood vessel endothelial cell migration | 2/36 | 79/18866 | 0.009946978 | 0.03439413 | 0.01667907 | KDR/PRKCA | 2 |
| GO:0071260 | cellular response to mechanical stimulus | 2/36 | 79/18866 | 0.009946978 | 0.03439413 | 0.01667907 | CHEK1/IL1B | 2 |
| GO:0043434 | response to peptide hormone | 4/36 | 447/18866 | 0.010060168 | 0.034704811 | 0.016829731 | F7/PRKCD/IL1B/EIF6 | 4 |
| GO:1904018 | positive regulation of vasculature development | 3/36 | 235/18866 | 0.010065102 | 0.034704811 | 0.016829731 | KDR/PRKCA/IL1B | 3 |
| GO:0048678 | response to axon injury | 2/36 | 80/18866 | 0.010189877 | 0.035036623 | 0.01699064 | JUN/BAX | 2 |
| GO:1901224 | positive regulation of NIK/NF-kappaB signaling | 2/36 | 80/18866 | 0.010189877 | 0.035036623 | 0.01699064 | TNF/IL1B | 2 |
| GO:0032872 | regulation of stress-activated MAPK cascade | 3/36 | 237/18866 | 0.010298633 | 0.035361041 | 0.017147963 | TNF/MYC/IL1B | 3 |
| GO:0010594 | regulation of endothelial cell migration | 3/36 | 238/18866 | 0.01041658 | 0.035680948 | 0.017303099 | KDR/TNF/PRKCA | 3 |
| GO:0019915 | lipid storage | 2/36 | 81/18866 | 0.010435406 | 0.035680948 | 0.017303099 | TNF/IL1B | 2 |
| GO:0051279 | regulation of release of sequestered calcium ion into cytosol | 2/36 | 81/18866 | 0.010435406 | 0.035680948 | 0.017303099 | PRKCE/BAX | 2 |
| GO:2000116 | regulation of cysteine-type endopeptidase activity | 3/36 | 239/18866 | 0.010535316 | 0.035972461 | 0.017444465 | TNF/MYC/BAX | 3 |
| GO:0050804 | modulation of chemical synaptic transmission | 4/36 | 454/18866 | 0.010607038 | 0.036167054 | 0.017538831 | TNF/PRKCE/IL1B/SLC6A4 | 4 |
| GO:0070302 | regulation of stress-activated protein kinase signaling cascade | 3/36 | 240/18866 | 0.010654841 | 0.036229411 | 0.01756907 | TNF/MYC/IL1B | 3 |
| GO:0070663 | regulation of leukocyte proliferation | 3/36 | 240/18866 | 0.010654841 | 0.036229411 | 0.01756907 | IL1B/CDKN1A/CASP3 | 3 |
| GO:0099177 | regulation of trans-synaptic signaling | 4/36 | 455/18866 | 0.010686711 | 0.036287519 | 0.017597249 | TNF/PRKCE/IL1B/SLC6A4 | 4 |
| GO:0006814 | sodium ion transport | 3/36 | 241/18866 | 0.010775157 | 0.036486913 | 0.017693943 | SCN5A/PRKCE/SLC6A4 | 3 |
| GO:0038093 | Fc receptor signaling pathway | 3/36 | 241/18866 | 0.010775157 | 0.036486913 | 0.017693943 | JUN/PRKCE/PRKCD | 3 |
| GO:0002718 | regulation of cytokine production involved in immune response | 2/36 | 83/18866 | 0.010934309 | 0.036822674 | 0.017856767 | TNF/IL1B | 2 |
| GO:0048708 | astrocyte differentiation | 2/36 | 83/18866 | 0.010934309 | 0.036822674 | 0.017856767 | TNF/IL1B | 2 |
| GO:0071277 | cellular response to calcium ion | 2/36 | 83/18866 | 0.010934309 | 0.036822674 | 0.017856767 | JUN/SCN5A | 2 |
| GO:2000243 | positive regulation of reproductive process | 2/36 | 83/18866 | 0.010934309 | 0.036822674 | 0.017856767 | AR/PDE3A | 2 |
| GO:0046209 | nitric oxide metabolic process | 2/36 | 84/18866 | 0.011187664 | 0.037624267 | 0.018245491 | TNF/IL1B | 2 |
| GO:0072330 | monocarboxylic acid biosynthetic process | 3/36 | 246/18866 | 0.011388637 | 0.038247748 | 0.018547841 | IL1B/FASN/EIF6 | 3 |
| GO:0001776 | leukocyte homeostasis | 2/36 | 86/18866 | 0.011702128 | 0.039033594 | 0.018928929 | CASP3/BAX | 2 |
| GO:0006919 | activation of cysteine-type endopeptidase activity involved in apoptotic process | 2/36 | 86/18866 | 0.011702128 | 0.039033594 | 0.018928929 | TNF/BAX | 2 |
| GO:0034109 | homotypic cell-cell adhesion | 2/36 | 86/18866 | 0.011702128 | 0.039033594 | 0.018928929 | PRKCD/PRKCA | 2 |
| GO:0097756 | negative regulation of blood vessel diameter | 2/36 | 86/18866 | 0.011702128 | 0.039033594 | 0.018928929 | SLC6A4/CHRM3 | 2 |
| GO:2000106 | regulation of leukocyte apoptotic process | 2/36 | 86/18866 | 0.011702128 | 0.039033594 | 0.018928929 | TP53/BAX | 2 |
| GO:0010256 | endomembrane system organization | 4/36 | 468/18866 | 0.011758159 | 0.039167272 | 0.018993755 | AR/PRKCD/PRKCA/CCNB1 | 4 |
| GO:0060249 | anatomical structure homeostasis | 4/36 | 469/18866 | 0.011843348 | 0.039397589 | 0.019105444 | PRKCA/PCNA/MYC/BAX | 4 |
| GO:0051781 | positive regulation of cell division | 2/36 | 87/18866 | 0.011963218 | 0.039688782 | 0.019246656 | PRKCE/IL1B | 2 |
| GO:2001057 | reactive nitrogen species metabolic process | 2/36 | 87/18866 | 0.011963218 | 0.039688782 | 0.019246656 | TNF/IL1B | 2 |
| GO:0071560 | cellular response to transforming growth factor beta stimulus | 3/36 | 252/18866 | 0.012151111 | 0.040257729 | 0.01952256 | JUN/TP53/PDE3A | 3 |
| GO:0002028 | regulation of sodium ion transport | 2/36 | 88/18866 | 0.012226864 | 0.040291211 | 0.019538797 | SCN5A/PRKCE | 2 |
| GO:0014068 | positive regulation of phosphatidylinositol 3-kinase signaling | 2/36 | 88/18866 | 0.012226864 | 0.040291211 | 0.019538797 | KDR/TNF | 2 |
| GO:0032755 | positive regulation of interleukin-6 production | 2/36 | 88/18866 | 0.012226864 | 0.040291211 | 0.019538797 | TNF/IL1B | 2 |
| GO:0090559 | regulation of membrane permeability | 2/36 | 88/18866 | 0.012226864 | 0.040291211 | 0.019538797 | TP53/BAX | 2 |
| GO:0031668 | cellular response to extracellular stimulus | 3/36 | 253/18866 | 0.012280992 | 0.040361224 | 0.019572749 | JUN/TP53/CDKN1A | 3 |
| GO:0051896 | regulation of protein kinase B signaling | 3/36 | 253/18866 | 0.012280992 | 0.040361224 | 0.019572749 | ESR1/F7/TNF | 3 |
| GO:0000086 | G2/M transition of mitotic cell cycle | 3/36 | 254/18866 | 0.012411675 | 0.040681793 | 0.019728206 | PKIA/CDKN1A/CCNB1 | 3 |
| GO:0019318 | hexose metabolic process | 3/36 | 254/18866 | 0.012411675 | 0.040681793 | 0.019728206 | NCOA2/TP53/TNF | 3 |
| GO:0008625 | extrinsic apoptotic signaling pathway via death domain receptors | 2/36 | 89/18866 | 0.012493059 | 0.040893946 | 0.019831087 | TNF/BAX | 2 |
| GO:0090257 | regulation of muscle system process | 3/36 | 256/18866 | 0.012675454 | 0.041435739 | 0.020093824 | SCN5A/PRKCA/CHRM3 | 3 |
| GO:0043470 | regulation of carbohydrate catabolic process | 2/36 | 90/18866 | 0.012761791 | 0.041662494 | 0.020203787 | TP53/EIF6 | 2 |
| GO:0071559 | response to transforming growth factor beta | 3/36 | 258/18866 | 0.012942454 | 0.042140219 | 0.020435454 | JUN/TP53/PDE3A | 3 |
| GO:1901617 | organic hydroxy compound biosynthetic process | 3/36 | 258/18866 | 0.012942454 | 0.042140219 | 0.020435454 | TNF/IL1B/FASN | 3 |
| GO:0042475 | odontogenesis of dentin-containing tooth | 2/36 | 91/18866 | 0.01303305 | 0.042155648 | 0.020442936 | SCN5A/BAX | 2 |
| GO:0048477 | oogenesis | 2/36 | 91/18866 | 0.01303305 | 0.042155648 | 0.020442936 | CCNB1/PDE3A | 2 |
| GO:0060333 | interferon-gamma-mediated signaling pathway | 2/36 | 91/18866 | 0.01303305 | 0.042155648 | 0.020442936 | TP53/PRKCD | 2 |
| GO:1904063 | negative regulation of cation transmembrane transport | 2/36 | 91/18866 | 0.01303305 | 0.042155648 | 0.020442936 | PRKCE/KCNH2 | 2 |
| GO:2000177 | regulation of neural precursor cell proliferation | 2/36 | 91/18866 | 0.01303305 | 0.042155648 | 0.020442936 | TP53/SLC6A4 | 2 |
| GO:0021537 | telencephalon development | 3/36 | 259/18866 | 0.013077164 | 0.042190639 | 0.020459905 | SCN5A/CASP3/BAX | 3 |
| GO:0042110 | T cell activation | 4/36 | 483/18866 | 0.013078239 | 0.042190639 | 0.020459905 | TP53/IL1B/CASP3/BAX | 4 |
| GO:0007611 | learning or memory | 3/36 | 260/18866 | 0.013212681 | 0.042512624 | 0.020616048 | JUN/CASP3/SLC6A4 | 3 |
| GO:0042180 | cellular ketone metabolic process | 3/36 | 260/18866 | 0.013212681 | 0.042512624 | 0.020616048 | PRKCE/IL1B/EIF6 | 3 |
| GO:0032677 | regulation of interleukin-8 production | 2/36 | 93/18866 | 0.01358311 | 0.043590242 | 0.021138627 | TNF/IL1B | 2 |
| GO:1903035 | negative regulation of response to wounding | 2/36 | 93/18866 | 0.01358311 | 0.043590242 | 0.021138627 | TNF/PRKCD | 2 |
| GO:0016049 | cell growth | 4/36 | 490/18866 | 0.013725606 | 0.043990028 | 0.021332499 | ESR2/TP53/CDKN1A/MAP2 | 4 |
| GO:1904035 | regulation of epithelial cell apoptotic process | 2/36 | 94/18866 | 0.013861891 | 0.044368895 | 0.021516227 | KDR/TNF | 2 |
| GO:0032412 | regulation of ion transmembrane transporter activity | 3/36 | 265/18866 | 0.013902407 | 0.044440636 | 0.021551017 | PRKCE/OPRM1/CHRM3 | 3 |
| GO:0007589 | body fluid secretion | 2/36 | 95/18866 | 0.014143159 | 0.045092801 | 0.021867277 | PRKCE/CHRM3 | 2 |
| GO:0050709 | negative regulation of protein secretion | 2/36 | 95/18866 | 0.014143159 | 0.045092801 | 0.021867277 | IL1B/OPRM1 | 2 |
| GO:0015850 | organic hydroxy compound transport | 3/36 | 268/18866 | 0.014325979 | 0.045616443 | 0.022121212 | NCOA2/SLC6A4/PON1 | 3 |
| GO:0050764 | regulation of phagocytosis | 2/36 | 97/18866 | 0.014713118 | 0.04672795 | 0.022660226 | TNF/IL1B | 2 |
| GO:1901655 | cellular response to ketone | 2/36 | 97/18866 | 0.014713118 | 0.04672795 | 0.022660226 | AR/PRKCE | 2 |
| GO:0045833 | negative regulation of lipid metabolic process | 2/36 | 98/18866 | 0.015001789 | 0.047521795 | 0.023045193 | TNF/IL1B | 2 |
| GO:0048010 | vascular endothelial growth factor receptor signaling pathway | 2/36 | 98/18866 | 0.015001789 | 0.047521795 | 0.023045193 | KDR/IL1B | 2 |
| GO:0007281 | germ cell development | 3/36 | 275/18866 | 0.015342826 | 0.048539481 | 0.023538708 | CCNB1/BAX/PDE3A | 3 |
| GO:0022898 | regulation of transmembrane transporter activity | 3/36 | 276/18866 | 0.015491357 | 0.048946308 | 0.023735995 | PRKCE/OPRM1/CHRM3 | 3 |
| GO:0006672 | ceramide metabolic process | 2/36 | 100/18866 | 0.015586463 | 0.049120368 | 0.023820404 | TNF/PRKCD | 2 |
| GO:0070301 | cellular response to hydrogen peroxide | 2/36 | 100/18866 | 0.015586463 | 0.049120368 | 0.023820404 | PRKCD/PCNA | 2 |
| GO:0015980 | energy derivation by oxidation of organic compounds | 3/36 | 278/18866 | 0.015790876 | 0.049637133 | 0.024071003 | TP53/MYC/CCNB1 | 3 |
| GO:0045165 | cell fate commitment | 3/36 | 278/18866 | 0.015790876 | 0.049637133 | 0.024071003 | AR/TP53/CASP3 | 3 |
| GO:0002792 | negative regulation of peptide secretion | 2/36 | 101/18866 | 0.015882447 | 0.049670583 | 0.024087224 | IL1B/OPRM1 | 2 |
| GO:0010522 | regulation of calcium ion transport into cytosol | 2/36 | 101/18866 | 0.015882447 | 0.049670583 | 0.024087224 | PRKCE/BAX | 2 |
| GO:0019217 | regulation of fatty acid metabolic process | 2/36 | 101/18866 | 0.015882447 | 0.049670583 | 0.024087224 | IL1B/EIF6 | 2 |
| GO:0032637 | interleukin-8 production | 2/36 | 101/18866 | 0.015882447 | 0.049670583 | 0.024087224 | TNF/IL1B | 2 |
| GO:0043502 | regulation of muscle adaptation | 2/36 | 102/18866 | 0.016180849 | 0.050539421 | 0.024508558 | SCN5A/PRKCA | 2 |
| GO:0072659 | protein localization to plasma membrane | 3/36 | 281/18866 | 0.016246303 | 0.050679383 | 0.024576431 | AR/TNF/PRKCE | 3 |
| GO:0071824 | protein-DNA complex subunit organization | 3/36 | 282/18866 | 0.016399754 | 0.051088983 | 0.024775063 | ESR1/TP53/MYC | 3 |
| GO:0034766 | negative regulation of ion transmembrane transport | 2/36 | 103/18866 | 0.01648166 | 0.051088983 | 0.024775063 | PRKCE/KCNH2 | 2 |
| GO:0048661 | positive regulation of smooth muscle cell proliferation | 2/36 | 103/18866 | 0.01648166 | 0.051088983 | 0.024775063 | JUN/TNF | 2 |
| GO:0060079 | excitatory postsynaptic potential | 2/36 | 103/18866 | 0.01648166 | 0.051088983 | 0.024775063 | OPRM1/CHRNA2 | 2 |
| GO:1901570 | fatty acid derivative biosynthetic process | 2/36 | 103/18866 | 0.01648166 | 0.051088983 | 0.024775063 | IL1B/FASN | 2 |
| GO:0032868 | response to insulin | 3/36 | 283/18866 | 0.016554026 | 0.051248594 | 0.024852464 | PRKCD/IL1B/EIF6 | 3 |
| GO:0002027 | regulation of heart rate | 2/36 | 104/18866 | 0.016784869 | 0.051637661 | 0.025041138 | SCN5A/KCNH2 | 2 |
| GO:0002367 | cytokine production involved in immune response | 2/36 | 104/18866 | 0.016784869 | 0.051637661 | 0.025041138 | TNF/IL1B | 2 |
| GO:0007200 | phospholipase C-activating G protein-coupled receptor signaling pathway | 2/36 | 104/18866 | 0.016784869 | 0.051637661 | 0.025041138 | ESR1/OPRM1 | 2 |
| GO:0032649 | regulation of interferon-gamma production | 2/36 | 104/18866 | 0.016784869 | 0.051637661 | 0.025041138 | TNF/IL1B | 2 |
| GO:0048525 | negative regulation of viral process | 2/36 | 104/18866 | 0.016784869 | 0.051637661 | 0.025041138 | JUN/TNF | 2 |
| GO:0043542 | endothelial cell migration | 3/36 | 286/18866 | 0.017021778 | 0.052235581 | 0.025331093 | KDR/TNF/PRKCA | 3 |
| GO:0051403 | stress-activated MAPK cascade | 3/36 | 286/18866 | 0.017021778 | 0.052235581 | 0.025331093 | TNF/MYC/IL1B | 3 |
| GO:0002824 | positive regulation of adaptive immune response based on somatic recombination of immune receptors built from immunoglobulin superfamily domains | 2/36 | 105/18866 | 0.017090467 | 0.052250433 | 0.025338295 | TNF/IL1B | 2 |
| GO:0021549 | cerebellum development | 2/36 | 105/18866 | 0.017090467 | 0.052250433 | 0.025338295 | SCN5A/TP53 | 2 |
| GO:0044070 | regulation of anion transport | 2/36 | 105/18866 | 0.017090467 | 0.052250433 | 0.025338295 | PRKCD/IL1B | 2 |
| GO:0002708 | positive regulation of lymphocyte mediated immunity | 2/36 | 106/18866 | 0.017398445 | 0.053059854 | 0.025730816 | TNF/IL1B | 2 |
| GO:0032611 | interleukin-1 beta production | 2/36 | 106/18866 | 0.017398445 | 0.053059854 | 0.025730816 | TNF/IL1B | 2 |
| GO:0030148 | sphingolipid biosynthetic process | 2/36 | 107/18866 | 0.017708792 | 0.053872472 | 0.026124886 | TNF/PRKCD | 2 |
| GO:1903426 | regulation of reactive oxygen species biosynthetic process | 2/36 | 107/18866 | 0.017708792 | 0.053872472 | 0.026124886 | TNF/IL1B | 2 |
| GO:1902750 | negative regulation of cell cycle G2/M phase transition | 2/36 | 108/18866 | 0.018021499 | 0.054755917 | 0.026553304 | CHEK1/CDKN1A | 2 |
| GO:0034976 | response to endoplasmic reticulum stress | 3/36 | 294/18866 | 0.018305377 | 0.055481112 | 0.026904979 | JUN/TP53/BAX | 3 |
| GO:0044262 | cellular carbohydrate metabolic process | 3/36 | 294/18866 | 0.018305377 | 0.055481112 | 0.026904979 | NCOA2/TP53/PRKCE | 3 |
| GO:0021761 | limbic system development | 2/36 | 109/18866 | 0.018336557 | 0.055507087 | 0.026917575 | CASP3/BAX | 2 |
| GO:0002821 | positive regulation of adaptive immune response | 2/36 | 110/18866 | 0.018653955 | 0.05577064 | 0.027045383 | TNF/IL1B | 2 |
| GO:0006289 | nucleotide-excision repair | 2/36 | 110/18866 | 0.018653955 | 0.05577064 | 0.027045383 | TP53/PCNA | 2 |
| GO:0006664 | glycolipid metabolic process | 2/36 | 110/18866 | 0.018653955 | 0.05577064 | 0.027045383 | PRKCD/BAX | 2 |
| GO:1904659 | glucose transmembrane transport | 2/36 | 110/18866 | 0.018653955 | 0.05577064 | 0.027045383 | TNF/IL1B | 2 |
| GO:0002676 | regulation of chronic inflammatory response | 1/36 | 10/18866 | 0.018923398 | 0.05577064 | 0.027045383 | TNF | 1 |
| GO:0002934 | desmosome organization | 1/36 | 10/18866 | 0.018923398 | 0.05577064 | 0.027045383 | PRKCA | 1 |
| GO:0014877 | response to muscle inactivity involved in regulation of muscle adaptation | 1/36 | 10/18866 | 0.018923398 | 0.05577064 | 0.027045383 | SCN5A | 1 |
| GO:0014894 | response to denervation involved in regulation of muscle adaptation | 1/36 | 10/18866 | 0.018923398 | 0.05577064 | 0.027045383 | SCN5A | 1 |
| GO:0021936 | regulation of cerebellar granule cell precursor proliferation | 1/36 | 10/18866 | 0.018923398 | 0.05577064 | 0.027045383 | SLC6A4 | 1 |
| GO:0032025 | response to cobalt ion | 1/36 | 10/18866 | 0.018923398 | 0.05577064 | 0.027045383 | CASP3 | 1 |
| GO:0032308 | positive regulation of prostaglandin secretion | 1/36 | 10/18866 | 0.018923398 | 0.05577064 | 0.027045383 | IL1B | 1 |
| GO:0033083 | regulation of immature T cell proliferation | 1/36 | 10/18866 | 0.018923398 | 0.05577064 | 0.027045383 | IL1B | 1 |
| GO:0051901 | positive regulation of mitochondrial depolarization | 1/36 | 10/18866 | 0.018923398 | 0.05577064 | 0.027045383 | KDR | 1 |
| GO:0060513 | prostatic bud formation | 1/36 | 10/18866 | 0.018923398 | 0.05577064 | 0.027045383 | AR | 1 |
| GO:0060768 | regulation of epithelial cell proliferation involved in prostate gland development | 1/36 | 10/18866 | 0.018923398 | 0.05577064 | 0.027045383 | AR | 1 |
| GO:0071394 | cellular response to testosterone stimulus | 1/36 | 10/18866 | 0.018923398 | 0.05577064 | 0.027045383 | AR | 1 |
| GO:0072203 | cell proliferation involved in metanephros development | 1/36 | 10/18866 | 0.018923398 | 0.05577064 | 0.027045383 | MYC | 1 |
| GO:0086070 | SA node cell to atrial cardiac muscle cell communication | 1/36 | 10/18866 | 0.018923398 | 0.05577064 | 0.027045383 | SCN5A | 1 |
| GO:1900222 | negative regulation of amyloid-beta clearance | 1/36 | 10/18866 | 0.018923398 | 0.05577064 | 0.027045383 | TNF | 1 |
| GO:1903862 | positive regulation of oxidative phosphorylation | 1/36 | 10/18866 | 0.018923398 | 0.05577064 | 0.027045383 | CCNB1 | 1 |
| GO:1904526 | regulation of microtubule binding | 1/36 | 10/18866 | 0.018923398 | 0.05577064 | 0.027045383 | MAP2 | 1 |
| GO:1904672 | regulation of somatic stem cell population maintenance | 1/36 | 10/18866 | 0.018923398 | 0.05577064 | 0.027045383 | MYC | 1 |
| GO:1903509 | liposaccharide metabolic process | 2/36 | 111/18866 | 0.018973685 | 0.055851795 | 0.027084738 | PRKCD/BAX | 2 |
| GO:0099565 | chemical synaptic transmission, postsynaptic | 2/36 | 112/18866 | 0.019295737 | 0.056616875 | 0.027455756 | OPRM1/CHRNA2 | 2 |
| GO:0031098 | stress-activated protein kinase signaling cascade | 3/36 | 300/18866 | 0.01930278 | 0.056616875 | 0.027455756 | TNF/MYC/IL1B | 3 |
| GO:0051258 | protein polymerization | 3/36 | 300/18866 | 0.01930278 | 0.056616875 | 0.027455756 | PRKCE/PRKCD/MAP2 | 3 |
| GO:0007009 | plasma membrane organization | 2/36 | 113/18866 | 0.0196201 | 0.05743009 | 0.027850115 | AR/PRKCD | 2 |
| GO:1903522 | regulation of blood circulation | 3/36 | 303/18866 | 0.01981266 | 0.05743009 | 0.027850115 | SCN5A/KCNH2/CHRM3 | 3 |
| GO:0008645 | hexose transmembrane transport | 2/36 | 114/18866 | 0.019946767 | 0.05743009 | 0.027850115 | TNF/IL1B | 2 |
| GO:0022037 | metencephalon development | 2/36 | 114/18866 | 0.019946767 | 0.05743009 | 0.027850115 | SCN5A/TP53 | 2 |
| GO:0032411 | positive regulation of transporter activity | 2/36 | 114/18866 | 0.019946767 | 0.05743009 | 0.027850115 | PRKCD/PON1 | 2 |
| GO:0032609 | interferon-gamma production | 2/36 | 115/18866 | 0.020275727 | 0.05743009 | 0.027850115 | TNF/IL1B | 2 |
| GO:0015749 | monosaccharide transmembrane transport | 2/36 | 116/18866 | 0.02060697 | 0.05743009 | 0.027850115 | TNF/IL1B | 2 |
| GO:0046620 | regulation of organ growth | 2/36 | 116/18866 | 0.02060697 | 0.05743009 | 0.027850115 | CCNB1/SLC6A4 | 2 |
| GO:0001820 | serotonin secretion | 1/36 | 11/18866 | 0.020796476 | 0.05743009 | 0.027850115 | SLC6A4 | 1 |
| GO:0007191 | adenylate cyclase-activating dopamine receptor signaling pathway | 1/36 | 11/18866 | 0.020796476 | 0.05743009 | 0.027850115 | OPRM1 | 1 |
| GO:0008611 | ether lipid biosynthetic process | 1/36 | 11/18866 | 0.020796476 | 0.05743009 | 0.027850115 | FASN | 1 |
| GO:0014870 | response to muscle inactivity | 1/36 | 11/18866 | 0.020796476 | 0.05743009 | 0.027850115 | SCN5A | 1 |
| GO:0017085 | response to insecticide | 1/36 | 11/18866 | 0.020796476 | 0.05743009 | 0.027850115 | CCNB1 | 1 |
| GO:0031442 | positive regulation of mRNA 3'-end processing | 1/36 | 11/18866 | 0.020796476 | 0.05743009 | 0.027850115 | CCNB1 | 1 |
| GO:0032253 | dense core granule localization | 1/36 | 11/18866 | 0.020796476 | 0.05743009 | 0.027850115 | MAP2 | 1 |
| GO:0032306 | regulation of prostaglandin secretion | 1/36 | 11/18866 | 0.020796476 | 0.05743009 | 0.027850115 | IL1B | 1 |
| GO:0033079 | immature T cell proliferation | 1/36 | 11/18866 | 0.020796476 | 0.05743009 | 0.027850115 | IL1B | 1 |
| GO:0033148 | positive regulation of intracellular estrogen receptor signaling pathway | 1/36 | 11/18866 | 0.020796476 | 0.05743009 | 0.027850115 | AR | 1 |
| GO:0033327 | Leydig cell differentiation | 1/36 | 11/18866 | 0.020796476 | 0.05743009 | 0.027850115 | AR | 1 |
| GO:0034776 | response to histamine | 1/36 | 11/18866 | 0.020796476 | 0.05743009 | 0.027850115 | GABRA1 | 1 |
| GO:0043471 | regulation of cellular carbohydrate catabolic process | 1/36 | 11/18866 | 0.020796476 | 0.05743009 | 0.027850115 | TP53 | 1 |
| GO:0045657 | positive regulation of monocyte differentiation | 1/36 | 11/18866 | 0.020796476 | 0.05743009 | 0.027850115 | JUN | 1 |
| GO:0045945 | positive regulation of transcription by RNA polymerase III | 1/36 | 11/18866 | 0.020796476 | 0.05743009 | 0.027850115 | AR | 1 |
| GO:0046504 | glycerol ether biosynthetic process | 1/36 | 11/18866 | 0.020796476 | 0.05743009 | 0.027850115 | FASN | 1 |
| GO:0051610 | serotonin uptake | 1/36 | 11/18866 | 0.020796476 | 0.05743009 | 0.027850115 | SLC6A4 | 1 |
| GO:0051974 | negative regulation of telomerase activity | 1/36 | 11/18866 | 0.020796476 | 0.05743009 | 0.027850115 | TP53 | 1 |
| GO:0060281 | regulation of oocyte development | 1/36 | 11/18866 | 0.020796476 | 0.05743009 | 0.027850115 | PDE3A | 1 |
| GO:0060525 | prostate glandular acinus development | 1/36 | 11/18866 | 0.020796476 | 0.05743009 | 0.027850115 | ESR1 | 1 |
| GO:0060767 | epithelial cell proliferation involved in prostate gland development | 1/36 | 11/18866 | 0.020796476 | 0.05743009 | 0.027850115 | AR | 1 |
| GO:0070254 | mucus secretion | 1/36 | 11/18866 | 0.020796476 | 0.05743009 | 0.027850115 | PRKCE | 1 |
| GO:0071281 | cellular response to iron ion | 1/36 | 11/18866 | 0.020796476 | 0.05743009 | 0.027850115 | CCNB1 | 1 |
| GO:0071803 | positive regulation of podosome assembly | 1/36 | 11/18866 | 0.020796476 | 0.05743009 | 0.027850115 | TNF | 1 |
| GO:0086016 | AV node cell action potential | 1/36 | 11/18866 | 0.020796476 | 0.05743009 | 0.027850115 | SCN5A | 1 |
| GO:0086027 | AV node cell to bundle of His cell signaling | 1/36 | 11/18866 | 0.020796476 | 0.05743009 | 0.027850115 | SCN5A | 1 |
| GO:0090557 | establishment of endothelial intestinal barrier | 1/36 | 11/18866 | 0.020796476 | 0.05743009 | 0.027850115 | FASN | 1 |
| GO:0097384 | cellular lipid biosynthetic process | 1/36 | 11/18866 | 0.020796476 | 0.05743009 | 0.027850115 | FASN | 1 |
| GO:0099519 | dense core granule cytoskeletal transport | 1/36 | 11/18866 | 0.020796476 | 0.05743009 | 0.027850115 | MAP2 | 1 |
| GO:0099624 | atrial cardiac muscle cell membrane repolarization | 1/36 | 11/18866 | 0.020796476 | 0.05743009 | 0.027850115 | SCN5A | 1 |
| GO:0106072 | negative regulation of adenylate cyclase-activating G protein-coupled receptor signaling pathway | 1/36 | 11/18866 | 0.020796476 | 0.05743009 | 0.027850115 | OPRM1 | 1 |
| GO:1901033 | positive regulation of response to reactive oxygen species | 1/36 | 11/18866 | 0.020796476 | 0.05743009 | 0.027850115 | TNF | 1 |
| GO:1901950 | dense core granule transport | 1/36 | 11/18866 | 0.020796476 | 0.05743009 | 0.027850115 | MAP2 | 1 |
| GO:1902065 | response to L-glutamate | 1/36 | 11/18866 | 0.020796476 | 0.05743009 | 0.027850115 | PCNA | 1 |
| GO:1902513 | regulation of organelle transport along microtubule | 1/36 | 11/18866 | 0.020796476 | 0.05743009 | 0.027850115 | MAP2 | 1 |
| GO:1903800 | positive regulation of production of miRNAs involved in gene silencing by miRNA | 1/36 | 11/18866 | 0.020796476 | 0.05743009 | 0.027850115 | TP53 | 1 |
| GO:1905879 | regulation of oogenesis | 1/36 | 11/18866 | 0.020796476 | 0.05743009 | 0.027850115 | PDE3A | 1 |
| GO:1990440 | positive regulation of transcription from RNA polymerase II promoter in response to endoplasmic reticulum stress | 1/36 | 11/18866 | 0.020796476 | 0.05743009 | 0.027850115 | TP53 | 1 |
| GO:2000480 | negative regulation of cAMP-dependent protein kinase activity | 1/36 | 11/18866 | 0.020796476 | 0.05743009 | 0.027850115 | PKIA | 1 |
| GO:2000574 | regulation of microtubule motor activity | 1/36 | 11/18866 | 0.020796476 | 0.05743009 | 0.027850115 | MAP2 | 1 |
| GO:2000615 | regulation of histone H3-K9 acetylation | 1/36 | 11/18866 | 0.020796476 | 0.05743009 | 0.027850115 | CHEK1 | 1 |
| GO:2001138 | regulation of phospholipid transport | 1/36 | 11/18866 | 0.020796476 | 0.05743009 | 0.027850115 | PRKCD | 1 |
| GO:2001140 | positive regulation of phospholipid transport | 1/36 | 11/18866 | 0.020796476 | 0.05743009 | 0.027850115 | PRKCD | 1 |
| GO:2001269 | positive regulation of cysteine-type endopeptidase activity involved in apoptotic signaling pathway | 1/36 | 11/18866 | 0.020796476 | 0.05743009 | 0.027850115 | BAX | 1 |
| GO:0051341 | regulation of oxidoreductase activity | 2/36 | 117/18866 | 0.020940488 | 0.057697979 | 0.027980025 | TNF/IL1B | 2 |
| GO:1904019 | epithelial cell apoptotic process | 2/36 | 117/18866 | 0.020940488 | 0.057697979 | 0.027980025 | KDR/TNF | 2 |
| GO:0016236 | macroautophagy | 3/36 | 310/18866 | 0.021031413 | 0.057883542 | 0.028070012 | KDR/TP53/CASP3 | 3 |
| GO:0046034 | ATP metabolic process | 3/36 | 311/18866 | 0.021208842 | 0.058306503 | 0.028275123 | TP53/EIF6/CCNB1 | 3 |
| GO:0034219 | carbohydrate transmembrane transport | 2/36 | 118/18866 | 0.021276272 | 0.05836117 | 0.028301633 | TNF/IL1B | 2 |
| GO:0051209 | release of sequestered calcium ion into cytosol | 2/36 | 118/18866 | 0.021276272 | 0.05836117 | 0.028301633 | PRKCE/BAX | 2 |
| GO:0070588 | calcium ion transmembrane transport | 3/36 | 312/18866 | 0.021387101 | 0.058599703 | 0.028417307 | PRKCE/BAX/OPRM1 | 3 |
| GO:0090305 | nucleic acid phosphodiester bond hydrolysis | 3/36 | 313/18866 | 0.021566192 | 0.059024527 | 0.028623321 | PCNA/CASP3/BAX | 3 |
| GO:0051283 | negative regulation of sequestering of calcium ion | 2/36 | 119/18866 | 0.021614311 | 0.05902462 | 0.028623366 | PRKCE/BAX | 2 |
| GO:1901222 | regulation of NIK/NF-kappaB signaling | 2/36 | 119/18866 | 0.021614311 | 0.05902462 | 0.028623366 | TNF/IL1B | 2 |
| GO:0002065 | columnar/cuboidal epithelial cell differentiation | 2/36 | 121/18866 | 0.022297119 | 0.059577323 | 0.028891394 | FASN/CDKN1A | 2 |
| GO:0010906 | regulation of glucose metabolic process | 2/36 | 121/18866 | 0.022297119 | 0.059577323 | 0.028891394 | NCOA2/TP53 | 2 |
| GO:0032612 | interleukin-1 production | 2/36 | 121/18866 | 0.022297119 | 0.059577323 | 0.028891394 | TNF/IL1B | 2 |
| GO:0051282 | regulation of sequestering of calcium ion | 2/36 | 121/18866 | 0.022297119 | 0.059577323 | 0.028891394 | PRKCE/BAX | 2 |
| GO:0006690 | icosanoid metabolic process | 2/36 | 122/18866 | 0.02264187 | 0.059577323 | 0.028891394 | IL1B/PON1 | 2 |
| GO:0010811 | positive regulation of cell-substrate adhesion | 2/36 | 122/18866 | 0.02264187 | 0.059577323 | 0.028891394 | KDR/PRKCE | 2 |
| GO:0021782 | glial cell development | 2/36 | 122/18866 | 0.02264187 | 0.059577323 | 0.028891394 | TNF/IL1B | 2 |
| GO:0045471 | response to ethanol | 2/36 | 122/18866 | 0.02264187 | 0.059577323 | 0.028891394 | PRKCE/OPRM1 | 2 |
| GO:0000185 | activation of MAPKKK activity | 1/36 | 12/18866 | 0.022666077 | 0.059577323 | 0.028891394 | TNF | 1 |
| GO:0002863 | positive regulation of inflammatory response to antigenic stimulus | 1/36 | 12/18866 | 0.022666077 | 0.059577323 | 0.028891394 | TNF | 1 |
| GO:0002923 | regulation of humoral immune response mediated by circulating immunoglobulin | 1/36 | 12/18866 | 0.022666077 | 0.059577323 | 0.028891394 | TNF | 1 |
| GO:0006983 | ER overload response | 1/36 | 12/18866 | 0.022666077 | 0.059577323 | 0.028891394 | TP53 | 1 |
| GO:0007320 | insemination | 1/36 | 12/18866 | 0.022666077 | 0.059577323 | 0.028891394 | SLC6A4 | 1 |
| GO:0031392 | regulation of prostaglandin biosynthetic process | 1/36 | 12/18866 | 0.022666077 | 0.059577323 | 0.028891394 | IL1B | 1 |
| GO:0035404 | histone-serine phosphorylation | 1/36 | 12/18866 | 0.022666077 | 0.059577323 | 0.028891394 | CCNB1 | 1 |
| GO:0038003 | opioid receptor signaling pathway | 1/36 | 12/18866 | 0.022666077 | 0.059577323 | 0.028891394 | OPRM1 | 1 |
| GO:0045760 | positive regulation of action potential | 1/36 | 12/18866 | 0.022666077 | 0.059577323 | 0.028891394 | SCN5A | 1 |
| GO:0046541 | saliva secretion | 1/36 | 12/18866 | 0.022666077 | 0.059577323 | 0.028891394 | CHRM3 | 1 |
| GO:0051095 | regulation of helicase activity | 1/36 | 12/18866 | 0.022666077 | 0.059577323 | 0.028891394 | TP53 | 1 |
| GO:0051798 | positive regulation of hair follicle development | 1/36 | 12/18866 | 0.022666077 | 0.059577323 | 0.028891394 | TNF | 1 |
| GO:0055015 | ventricular cardiac muscle cell development | 1/36 | 12/18866 | 0.022666077 | 0.059577323 | 0.028891394 | CCNB1 | 1 |
| GO:0060442 | branching involved in prostate gland morphogenesis | 1/36 | 12/18866 | 0.022666077 | 0.059577323 | 0.028891394 | ESR1 | 1 |
| GO:0060742 | epithelial cell differentiation involved in prostate gland development | 1/36 | 12/18866 | 0.022666077 | 0.059577323 | 0.028891394 | AR | 1 |
| GO:0070243 | regulation of thymocyte apoptotic process | 1/36 | 12/18866 | 0.022666077 | 0.059577323 | 0.028891394 | TP53 | 1 |
| GO:0071639 | positive regulation of monocyte chemotactic protein-1 production | 1/36 | 12/18866 | 0.022666077 | 0.059577323 | 0.028891394 | IL1B | 1 |
| GO:0072520 | seminiferous tubule development | 1/36 | 12/18866 | 0.022666077 | 0.059577323 | 0.028891394 | AR | 1 |
| GO:0086067 | AV node cell to bundle of His cell communication | 1/36 | 12/18866 | 0.022666077 | 0.059577323 | 0.028891394 | SCN5A | 1 |
| GO:0090331 | negative regulation of platelet aggregation | 1/36 | 12/18866 | 0.022666077 | 0.059577323 | 0.028891394 | PRKCD | 1 |
| GO:0097201 | negative regulation of transcription from RNA polymerase II promoter in response to stress | 1/36 | 12/18866 | 0.022666077 | 0.059577323 | 0.028891394 | JUN | 1 |
| GO:1901503 | ether biosynthetic process | 1/36 | 12/18866 | 0.022666077 | 0.059577323 | 0.028891394 | FASN | 1 |
| GO:1902337 | regulation of apoptotic process involved in morphogenesis | 1/36 | 12/18866 | 0.022666077 | 0.059577323 | 0.028891394 | BAX | 1 |
| GO:1902510 | regulation of apoptotic DNA fragmentation | 1/36 | 12/18866 | 0.022666077 | 0.059577323 | 0.028891394 | BAX | 1 |
| GO:1904181 | positive regulation of membrane depolarization | 1/36 | 12/18866 | 0.022666077 | 0.059577323 | 0.028891394 | KDR | 1 |
| GO:2000650 | negative regulation of sodium ion transmembrane transporter activity | 1/36 | 12/18866 | 0.022666077 | 0.059577323 | 0.028891394 | PRKCE | 1 |
| GO:2001214 | positive regulation of vasculogenesis | 1/36 | 12/18866 | 0.022666077 | 0.059577323 | 0.028891394 | KDR | 1 |
| GO:0034612 | response to tumor necrosis factor | 3/36 | 320/18866 | 0.022843102 | 0.059978413 | 0.029085898 | TP53/TNF/CASP3 | 3 |
| GO:0070252 | actin-mediated cell contraction | 2/36 | 123/18866 | 0.022988839 | 0.060296581 | 0.02924019 | SCN5A/KCNH2 | 2 |
| GO:0007204 | positive regulation of cytosolic calcium ion concentration | 3/36 | 322/18866 | 0.023215419 | 0.060825885 | 0.029496871 | ESR1/PRKCE/BAX | 3 |
| GO:0051208 | sequestering of calcium ion | 2/36 | 124/18866 | 0.023338018 | 0.061081912 | 0.029621028 | PRKCE/BAX | 2 |
| GO:0009895 | negative regulation of catabolic process | 3/36 | 323/18866 | 0.023402826 | 0.061186302 | 0.029671651 | TP53/TNF/IL1B | 3 |
| GO:0014066 | regulation of phosphatidylinositol 3-kinase signaling | 2/36 | 127/18866 | 0.024398719 | 0.062313685 | 0.030218363 | KDR/TNF | 2 |
| GO:0051053 | negative regulation of DNA metabolic process | 2/36 | 127/18866 | 0.024398719 | 0.062313685 | 0.030218363 | TP53/CDKN1A | 2 |
| GO:1900180 | regulation of protein localization to nucleus | 2/36 | 127/18866 | 0.024398719 | 0.062313685 | 0.030218363 | PRKCD/PKIA | 2 |
| GO:0006684 | sphingomyelin metabolic process | 1/36 | 13/18866 | 0.024532208 | 0.062313685 | 0.030218363 | PRKCD | 1 |
| GO:0009886 | post-embryonic animal morphogenesis | 1/36 | 13/18866 | 0.024532208 | 0.062313685 | 0.030218363 | BAX | 1 |
| GO:0016264 | gap junction assembly | 1/36 | 13/18866 | 0.024532208 | 0.062313685 | 0.030218363 | IL1B | 1 |
| GO:0031115 | negative regulation of microtubule polymerization | 1/36 | 13/18866 | 0.024532208 | 0.062313685 | 0.030218363 | MAP2 | 1 |
| GO:0031953 | negative regulation of protein autophosphorylation | 1/36 | 13/18866 | 0.024532208 | 0.062313685 | 0.030218363 | JUN | 1 |
| GO:0032230 | positive regulation of synaptic transmission, GABAergic | 1/36 | 13/18866 | 0.024532208 | 0.062313685 | 0.030218363 | PRKCE | 1 |
| GO:0032725 | positive regulation of granulocyte macrophage colony-stimulating factor production | 1/36 | 13/18866 | 0.024532208 | 0.062313685 | 0.030218363 | IL1B | 1 |
| GO:0033089 | positive regulation of T cell differentiation in thymus | 1/36 | 13/18866 | 0.024532208 | 0.062313685 | 0.030218363 | IL1B | 1 |
| GO:0033145 | positive regulation of intracellular steroid hormone receptor signaling pathway | 1/36 | 13/18866 | 0.024532208 | 0.062313685 | 0.030218363 | AR | 1 |
| GO:0035641 | locomotory exploration behavior | 1/36 | 13/18866 | 0.024532208 | 0.062313685 | 0.030218363 | PRKCE | 1 |
| GO:0042308 | negative regulation of protein import into nucleus | 1/36 | 13/18866 | 0.024532208 | 0.062313685 | 0.030218363 | PKIA | 1 |
| GO:0042635 | positive regulation of hair cycle | 1/36 | 13/18866 | 0.024532208 | 0.062313685 | 0.030218363 | TNF | 1 |
| GO:0043568 | positive regulation of insulin-like growth factor receptor signaling pathway | 1/36 | 13/18866 | 0.024532208 | 0.062313685 | 0.030218363 | AR | 1 |
| GO:0043970 | histone H3-K9 acetylation | 1/36 | 13/18866 | 0.024532208 | 0.062313685 | 0.030218363 | CHEK1 | 1 |
| GO:0048096 | chromatin-mediated maintenance of transcription | 1/36 | 13/18866 | 0.024532208 | 0.062313685 | 0.030218363 | CHEK1 | 1 |
| GO:0051988 | regulation of attachment of spindle microtubules to kinetochore | 1/36 | 13/18866 | 0.024532208 | 0.062313685 | 0.030218363 | CCNB1 | 1 |
| GO:0060601 | lateral sprouting from an epithelium | 1/36 | 13/18866 | 0.024532208 | 0.062313685 | 0.030218363 | AR | 1 |
| GO:0061029 | eyelid development in camera-type eye | 1/36 | 13/18866 | 0.024532208 | 0.062313685 | 0.030218363 | JUN | 1 |
| GO:0061043 | regulation of vascular wound healing | 1/36 | 13/18866 | 0.024532208 | 0.062313685 | 0.030218363 | TNF | 1 |
| GO:1900103 | positive regulation of endoplasmic reticulum unfolded protein response | 1/36 | 13/18866 | 0.024532208 | 0.062313685 | 0.030218363 | BAX | 1 |
| GO:1902306 | negative regulation of sodium ion transmembrane transport | 1/36 | 13/18866 | 0.024532208 | 0.062313685 | 0.030218363 | PRKCE | 1 |
| GO:1903651 | positive regulation of cytoplasmic transport | 1/36 | 13/18866 | 0.024532208 | 0.062313685 | 0.030218363 | MAP2 | 1 |
| GO:1904590 | negative regulation of protein import | 1/36 | 13/18866 | 0.024532208 | 0.062313685 | 0.030218363 | PKIA | 1 |
| GO:2000121 | regulation of removal of superoxide radicals | 1/36 | 13/18866 | 0.024532208 | 0.062313685 | 0.030218363 | TNF | 1 |
| GO:1903037 | regulation of leukocyte cell-cell adhesion | 3/36 | 329/18866 | 0.024544739 | 0.062313685 | 0.030218363 | TNF/IL1B/CASP3 | 3 |
| GO:0014013 | regulation of gliogenesis | 2/36 | 128/18866 | 0.024756644 | 0.062721941 | 0.030416342 | TNF/IL1B | 2 |
| GO:1903409 | reactive oxygen species biosynthetic process | 2/36 | 128/18866 | 0.024756644 | 0.062721941 | 0.030416342 | TNF/IL1B | 2 |
| GO:0032147 | activation of protein kinase activity | 3/36 | 331/18866 | 0.024932034 | 0.063101178 | 0.03060025 | TNF/PRKCD/IL1B | 3 |
| GO:0006282 | regulation of DNA repair | 2/36 | 132/18866 | 0.02620989 | 0.064670075 | 0.03136107 | CHEK1/PCNA | 2 |
| GO:0010595 | positive regulation of endothelial cell migration | 2/36 | 132/18866 | 0.02620989 | 0.064670075 | 0.03136107 | KDR/PRKCA | 2 |
| GO:0042476 | odontogenesis | 2/36 | 132/18866 | 0.02620989 | 0.064670075 | 0.03136107 | SCN5A/BAX | 2 |
| GO:0000054 | ribosomal subunit export from nucleus | 1/36 | 14/18866 | 0.026394874 | 0.064670075 | 0.03136107 | EIF6 | 1 |
| GO:0007100 | mitotic centrosome separation | 1/36 | 14/18866 | 0.026394874 | 0.064670075 | 0.03136107 | CHEK1 | 1 |
| GO:0010248 | establishment or maintenance of transmembrane electrochemical gradient | 1/36 | 14/18866 | 0.026394874 | 0.064670075 | 0.03136107 | BAX | 1 |
| GO:0010917 | negative regulation of mitochondrial membrane potential | 1/36 | 14/18866 | 0.026394874 | 0.064670075 | 0.03136107 | BAX | 1 |
| GO:0014854 | response to inactivity | 1/36 | 14/18866 | 0.026394874 | 0.064670075 | 0.03136107 | SCN5A | 1 |
| GO:0014874 | response to stimulus involved in regulation of muscle adaptation | 1/36 | 14/18866 | 0.026394874 | 0.064670075 | 0.03136107 | SCN5A | 1 |
| GO:0030213 | hyaluronan biosynthetic process | 1/36 | 14/18866 | 0.026394874 | 0.064670075 | 0.03136107 | IL1B | 1 |
| GO:0032042 | mitochondrial DNA metabolic process | 1/36 | 14/18866 | 0.026394874 | 0.064670075 | 0.03136107 | TP53 | 1 |
| GO:0032310 | prostaglandin secretion | 1/36 | 14/18866 | 0.026394874 | 0.064670075 | 0.03136107 | IL1B | 1 |
| GO:0033750 | ribosome localization | 1/36 | 14/18866 | 0.026394874 | 0.064670075 | 0.03136107 | EIF6 | 1 |
| GO:0035112 | genitalia morphogenesis | 1/36 | 14/18866 | 0.026394874 | 0.064670075 | 0.03136107 | AR | 1 |
| GO:0042659 | regulation of cell fate specification | 1/36 | 14/18866 | 0.026394874 | 0.064670075 | 0.03136107 | AR | 1 |
| GO:0043922 | negative regulation by host of viral transcription | 1/36 | 14/18866 | 0.026394874 | 0.064670075 | 0.03136107 | JUN | 1 |
| GO:0046007 | negative regulation of activated T cell proliferation | 1/36 | 14/18866 | 0.026394874 | 0.064670075 | 0.03136107 | CASP3 | 1 |
| GO:0070234 | positive regulation of T cell apoptotic process | 1/36 | 14/18866 | 0.026394874 | 0.064670075 | 0.03136107 | TP53 | 1 |
| GO:0070486 | leukocyte aggregation | 1/36 | 14/18866 | 0.026394874 | 0.064670075 | 0.03136107 | IL1B | 1 |
| GO:0071236 | cellular response to antibiotic | 1/36 | 14/18866 | 0.026394874 | 0.064670075 | 0.03136107 | TP53 | 1 |
| GO:0071361 | cellular response to ethanol | 1/36 | 14/18866 | 0.026394874 | 0.064670075 | 0.03136107 | PRKCE | 1 |
| GO:0072216 | positive regulation of metanephros development | 1/36 | 14/18866 | 0.026394874 | 0.064670075 | 0.03136107 | MYC | 1 |
| GO:0072425 | signal transduction involved in G2 DNA damage checkpoint | 1/36 | 14/18866 | 0.026394874 | 0.064670075 | 0.03136107 | CHEK1 | 1 |
| GO:0086069 | bundle of His cell to Purkinje myocyte communication | 1/36 | 14/18866 | 0.026394874 | 0.064670075 | 0.03136107 | SCN5A | 1 |
| GO:0090231 | regulation of spindle checkpoint | 1/36 | 14/18866 | 0.026394874 | 0.064670075 | 0.03136107 | CCNB1 | 1 |
| GO:0090266 | regulation of mitotic cell cycle spindle assembly checkpoint | 1/36 | 14/18866 | 0.026394874 | 0.064670075 | 0.03136107 | CCNB1 | 1 |
| GO:1901722 | regulation of cell proliferation involved in kidney development | 1/36 | 14/18866 | 0.026394874 | 0.064670075 | 0.03136107 | MYC | 1 |
| GO:1903504 | regulation of mitotic spindle checkpoint | 1/36 | 14/18866 | 0.026394874 | 0.064670075 | 0.03136107 | CCNB1 | 1 |
| GO:1903624 | regulation of DNA catabolic process | 1/36 | 14/18866 | 0.026394874 | 0.064670075 | 0.03136107 | BAX | 1 |
| GO:1903894 | regulation of IRE1-mediated unfolded protein response | 1/36 | 14/18866 | 0.026394874 | 0.064670075 | 0.03136107 | BAX | 1 |
| GO:1904748 | regulation of apoptotic process involved in development | 1/36 | 14/18866 | 0.026394874 | 0.064670075 | 0.03136107 | BAX | 1 |
| GO:2001279 | regulation of unsaturated fatty acid biosynthetic process | 1/36 | 14/18866 | 0.026394874 | 0.064670075 | 0.03136107 | IL1B | 1 |
| GO:0006997 | nucleus organization | 2/36 | 133/18866 | 0.026578543 | 0.065055158 | 0.031547812 | PRKCA/CCNB1 | 2 |
| GO:1990778 | protein localization to cell periphery | 3/36 | 340/18866 | 0.026716043 | 0.06532658 | 0.031679435 | AR/TNF/PRKCE | 3 |
| GO:0002920 | regulation of humoral immune response | 2/36 | 134/18866 | 0.026949315 | 0.065831411 | 0.031924247 | TNF/IL1B | 2 |
| GO:0043405 | regulation of MAP kinase activity | 3/36 | 342/18866 | 0.027121638 | 0.066186503 | 0.032096445 | TNF/PRKCD/IL1B | 3 |
| GO:1903311 | regulation of mRNA metabolic process | 3/36 | 344/18866 | 0.027530558 | 0.066888881 | 0.032437056 | PRKCD/PRKCA/CCNB1 | 3 |
| GO:0045727 | positive regulation of translation | 2/36 | 136/18866 | 0.027697179 | 0.066888881 | 0.032437056 | TNF/EIF6 | 2 |
| GO:0050671 | positive regulation of lymphocyte proliferation | 2/36 | 136/18866 | 0.027697179 | 0.066888881 | 0.032437056 | IL1B/CDKN1A | 2 |
| GO:0071902 | positive regulation of protein serine/threonine kinase activity | 3/36 | 345/18866 | 0.027736265 | 0.066888881 | 0.032437056 | TNF/IL1B/CCNB1 | 3 |
| GO:0007265 | Ras protein signal transduction | 3/36 | 346/18866 | 0.027942802 | 0.066888881 | 0.032437056 | JUN/TP53/CDKN1A | 3 |
| GO:0030183 | B cell differentiation | 2/36 | 137/18866 | 0.028074253 | 0.066888881 | 0.032437056 | TP53/BAX | 2 |
| GO:0032946 | positive regulation of mononuclear cell proliferation | 2/36 | 137/18866 | 0.028074253 | 0.066888881 | 0.032437056 | IL1B/CDKN1A | 2 |
| GO:0010506 | regulation of autophagy | 3/36 | 347/18866 | 0.02815017 | 0.066888881 | 0.032437056 | KDR/TP53/CASP3 | 3 |
| GO:0002281 | macrophage activation involved in immune response | 1/36 | 15/18866 | 0.028254081 | 0.066888881 | 0.032437056 | PRKCE | 1 |
| GO:0006089 | lactate metabolic process | 1/36 | 15/18866 | 0.028254081 | 0.066888881 | 0.032437056 | TP53 | 1 |
| GO:0010763 | positive regulation of fibroblast migration | 1/36 | 15/18866 | 0.028254081 | 0.066888881 | 0.032437056 | PRKCE | 1 |
| GO:0015671 | oxygen transport | 1/36 | 15/18866 | 0.028254081 | 0.066888881 | 0.032437056 | MYC | 1 |
| GO:0021924 | cell proliferation in external granule layer | 1/36 | 15/18866 | 0.028254081 | 0.066888881 | 0.032437056 | SLC6A4 | 1 |
| GO:0021930 | cerebellar granule cell precursor proliferation | 1/36 | 15/18866 | 0.028254081 | 0.066888881 | 0.032437056 | SLC6A4 | 1 |
| GO:0030238 | male sex determination | 1/36 | 15/18866 | 0.028254081 | 0.066888881 | 0.032437056 | AR | 1 |
| GO:0032252 | secretory granule localization | 1/36 | 15/18866 | 0.028254081 | 0.066888881 | 0.032437056 | MAP2 | 1 |
| GO:0032645 | regulation of granulocyte macrophage colony-stimulating factor production | 1/36 | 15/18866 | 0.028254081 | 0.066888881 | 0.032437056 | IL1B | 1 |
| GO:0034111 | negative regulation of homotypic cell-cell adhesion | 1/36 | 15/18866 | 0.028254081 | 0.066888881 | 0.032437056 | PRKCD | 1 |
| GO:0045475 | locomotor rhythm | 1/36 | 15/18866 | 0.028254081 | 0.066888881 | 0.032437056 | NCOA2 | 1 |
| GO:0045651 | positive regulation of macrophage differentiation | 1/36 | 15/18866 | 0.028254081 | 0.066888881 | 0.032437056 | PRKCA | 1 |
| GO:0045837 | negative regulation of membrane potential | 1/36 | 15/18866 | 0.028254081 | 0.066888881 | 0.032437056 | BAX | 1 |
| GO:0046479 | glycosphingolipid catabolic process | 1/36 | 15/18866 | 0.028254081 | 0.066888881 | 0.032437056 | PRKCD | 1 |
| GO:0048070 | regulation of developmental pigmentation | 1/36 | 15/18866 | 0.028254081 | 0.066888881 | 0.032437056 | BAX | 1 |
| GO:0048569 | post-embryonic animal organ development | 1/36 | 15/18866 | 0.028254081 | 0.066888881 | 0.032437056 | BAX | 1 |
| GO:0051299 | centrosome separation | 1/36 | 15/18866 | 0.028254081 | 0.066888881 | 0.032437056 | CHEK1 | 1 |
| GO:0071428 | rRNA-containing ribonucleoprotein complex export from nucleus | 1/36 | 15/18866 | 0.028254081 | 0.066888881 | 0.032437056 | EIF6 | 1 |
| GO:0071801 | regulation of podosome assembly | 1/36 | 15/18866 | 0.028254081 | 0.066888881 | 0.032437056 | TNF | 1 |
| GO:0072075 | metanephric mesenchyme development | 1/36 | 15/18866 | 0.028254081 | 0.066888881 | 0.032437056 | MYC | 1 |
| GO:0072567 | chemokine (C-X-C motif) ligand 2 production | 1/36 | 15/18866 | 0.028254081 | 0.066888881 | 0.032437056 | TNF | 1 |
| GO:2000341 | regulation of chemokine (C-X-C motif) ligand 2 production | 1/36 | 15/18866 | 0.028254081 | 0.066888881 | 0.032437056 | TNF | 1 |
| GO:2001028 | positive regulation of endothelial cell chemotaxis | 1/36 | 15/18866 | 0.028254081 | 0.066888881 | 0.032437056 | KDR | 1 |
| GO:0001508 | action potential | 2/36 | 138/18866 | 0.02845341 | 0.067166464 | 0.032571667 | SCN5A/KCNH2 | 2 |
| GO:0002705 | positive regulation of leukocyte mediated immunity | 2/36 | 138/18866 | 0.02845341 | 0.067166464 | 0.032571667 | TNF/IL1B | 2 |
| GO:0046330 | positive regulation of JNK cascade | 2/36 | 138/18866 | 0.02845341 | 0.067166464 | 0.032571667 | TNF/IL1B | 2 |
| GO:0002433 | immune response-regulating cell surface receptor signaling pathway involved in phagocytosis | 2/36 | 139/18866 | 0.028834642 | 0.06787061 | 0.032913135 | PRKCE/PRKCD | 2 |
| GO:0035304 | regulation of protein dephosphorylation | 2/36 | 139/18866 | 0.028834642 | 0.06787061 | 0.032913135 | TNF/PRKCD | 2 |
| GO:0038096 | Fc-gamma receptor signaling pathway involved in phagocytosis | 2/36 | 139/18866 | 0.028834642 | 0.06787061 | 0.032913135 | PRKCE/PRKCD | 2 |
| GO:0060048 | cardiac muscle contraction | 2/36 | 140/18866 | 0.029217939 | 0.068575564 | 0.033254995 | SCN5A/KCNH2 | 2 |
| GO:0072655 | establishment of protein localization to mitochondrion | 2/36 | 140/18866 | 0.029217939 | 0.068575564 | 0.033254995 | TP53/BAX | 2 |
| GO:0097553 | calcium ion transmembrane import into cytosol | 2/36 | 140/18866 | 0.029217939 | 0.068575564 | 0.033254995 | PRKCE/BAX | 2 |
| GO:0001889 | liver development | 2/36 | 141/18866 | 0.029603292 | 0.069213156 | 0.033564189 | JUN/PCNA | 2 |
| GO:0007586 | digestion | 2/36 | 141/18866 | 0.029603292 | 0.069213156 | 0.033564189 | PRSS1/CHRM3 | 2 |
| GO:0002687 | positive regulation of leukocyte migration | 2/36 | 142/18866 | 0.029990694 | 0.069213156 | 0.033564189 | F7/TNF | 2 |
| GO:0007006 | mitochondrial membrane organization | 2/36 | 142/18866 | 0.029990694 | 0.069213156 | 0.033564189 | TP53/BAX | 2 |
| GO:0031333 | negative regulation of protein-containing complex assembly | 2/36 | 142/18866 | 0.029990694 | 0.069213156 | 0.033564189 | PRKCD/MAP2 | 2 |
| GO:0038094 | Fc-gamma receptor signaling pathway | 2/36 | 142/18866 | 0.029990694 | 0.069213156 | 0.033564189 | PRKCE/PRKCD | 2 |
| GO:0006098 | pentose-phosphate shunt | 1/36 | 16/18866 | 0.030109837 | 0.069213156 | 0.033564189 | TP53 | 1 |
| GO:0010225 | response to UV-C | 1/36 | 16/18866 | 0.030109837 | 0.069213156 | 0.033564189 | TP53 | 1 |
| GO:0019372 | lipoxygenase pathway | 1/36 | 16/18866 | 0.030109837 | 0.069213156 | 0.033564189 | PON1 | 1 |
| GO:0021534 | cell proliferation in hindbrain | 1/36 | 16/18866 | 0.030109837 | 0.069213156 | 0.033564189 | SLC6A4 | 1 |
| GO:0032225 | regulation of synaptic transmission, dopaminergic | 1/36 | 16/18866 | 0.030109837 | 0.069213156 | 0.033564189 | SLC6A4 | 1 |
| GO:0032604 | granulocyte macrophage colony-stimulating factor production | 1/36 | 16/18866 | 0.030109837 | 0.069213156 | 0.033564189 | IL1B | 1 |
| GO:0043116 | negative regulation of vascular permeability | 1/36 | 16/18866 | 0.030109837 | 0.069213156 | 0.033564189 | PDE3A | 1 |
| GO:0046325 | negative regulation of glucose import | 1/36 | 16/18866 | 0.030109837 | 0.069213156 | 0.033564189 | TNF | 1 |
| GO:0051770 | positive regulation of nitric-oxide synthase biosynthetic process | 1/36 | 16/18866 | 0.030109837 | 0.069213156 | 0.033564189 | KDR | 1 |
| GO:0060572 | morphogenesis of an epithelial bud | 1/36 | 16/18866 | 0.030109837 | 0.069213156 | 0.033564189 | AR | 1 |
| GO:0060576 | intestinal epithelial cell development | 1/36 | 16/18866 | 0.030109837 | 0.069213156 | 0.033564189 | CDKN1A | 1 |
| GO:0070886 | positive regulation of calcineurin-NFAT signaling cascade | 1/36 | 16/18866 | 0.030109837 | 0.069213156 | 0.033564189 | TNF | 1 |
| GO:0071380 | cellular response to prostaglandin E stimulus | 1/36 | 16/18866 | 0.030109837 | 0.069213156 | 0.033564189 | PRKCE | 1 |
| GO:0106058 | positive regulation of calcineurin-mediated signaling | 1/36 | 16/18866 | 0.030109837 | 0.069213156 | 0.033564189 | TNF | 1 |
| GO:1904862 | inhibitory synapse assembly | 1/36 | 16/18866 | 0.030109837 | 0.069213156 | 0.033564189 | GABRA1 | 1 |
| GO:2001212 | regulation of vasculogenesis | 1/36 | 16/18866 | 0.030109837 | 0.069213156 | 0.033564189 | KDR | 1 |
| GO:0051480 | regulation of cytosolic calcium ion concentration | 3/36 | 357/18866 | 0.030269492 | 0.069515064 | 0.033710596 | ESR1/PRKCE/BAX | 3 |
| GO:0008286 | insulin receptor signaling pathway | 2/36 | 143/18866 | 0.030380134 | 0.069573907 | 0.033739131 | PRKCD/IL1B | 2 |
| GO:0035296 | regulation of tube diameter | 2/36 | 143/18866 | 0.030380134 | 0.069573907 | 0.033739131 | SLC6A4/CHRM3 | 2 |
| GO:0097746 | regulation of blood vessel diameter | 2/36 | 143/18866 | 0.030380134 | 0.069573907 | 0.033739131 | SLC6A4/CHRM3 | 2 |
| GO:0002700 | regulation of production of molecular mediator of immune response | 2/36 | 144/18866 | 0.030771605 | 0.07007819 | 0.033983678 | TNF/IL1B | 2 |
| GO:0035150 | regulation of tube size | 2/36 | 144/18866 | 0.030771605 | 0.07007819 | 0.033983678 | SLC6A4/CHRM3 | 2 |
| GO:0046434 | organophosphate catabolic process | 2/36 | 144/18866 | 0.030771605 | 0.07007819 | 0.033983678 | PRKCD/PON1 | 2 |
| GO:0050921 | positive regulation of chemotaxis | 2/36 | 144/18866 | 0.030771605 | 0.07007819 | 0.033983678 | KDR/F7 | 2 |
| GO:0061008 | hepaticobiliary system development | 2/36 | 144/18866 | 0.030771605 | 0.07007819 | 0.033983678 | JUN/PCNA | 2 |
| GO:0070585 | protein localization to mitochondrion | 2/36 | 144/18866 | 0.030771605 | 0.07007819 | 0.033983678 | TP53/BAX | 2 |
| GO:0002431 | Fc receptor mediated stimulatory signaling pathway | 2/36 | 145/18866 | 0.031165097 | 0.070842882 | 0.034354507 | PRKCE/PRKCD | 2 |
| GO:0033135 | regulation of peptidyl-serine phosphorylation | 2/36 | 145/18866 | 0.031165097 | 0.070842882 | 0.034354507 | TNF/BAX | 2 |
| GO:0006936 | muscle contraction | 3/36 | 362/18866 | 0.03136023 | 0.071204239 | 0.034529744 | SCN5A/KCNH2/CHRM3 | 3 |
| GO:0007159 | leukocyte cell-cell adhesion | 3/36 | 364/18866 | 0.031802316 | 0.071204239 | 0.034529744 | TNF/IL1B/CASP3 | 3 |
| GO:0007189 | adenylate cyclase-activating G protein-coupled receptor signaling pathway | 2/36 | 147/18866 | 0.031958112 | 0.071204239 | 0.034529744 | PRKCA/OPRM1 | 2 |
| GO:0046467 | membrane lipid biosynthetic process | 2/36 | 147/18866 | 0.031958112 | 0.071204239 | 0.034529744 | TNF/PRKCD | 2 |
| GO:0051592 | response to calcium ion | 2/36 | 147/18866 | 0.031958112 | 0.071204239 | 0.034529744 | JUN/SCN5A | 2 |
| GO:1904064 | positive regulation of cation transmembrane transport | 2/36 | 147/18866 | 0.031958112 | 0.071204239 | 0.034529744 | BAX/KCNH2 | 2 |
| GO:0008340 | determination of adult lifespan | 1/36 | 17/18866 | 0.031962147 | 0.071204239 | 0.034529744 | TP53 | 1 |
| GO:0009299 | mRNA transcription | 1/36 | 17/18866 | 0.031962147 | 0.071204239 | 0.034529744 | TP53 | 1 |
| GO:0010224 | response to UV-B | 1/36 | 17/18866 | 0.031962147 | 0.071204239 | 0.034529744 | CDKN1A | 1 |
| GO:0015732 | prostaglandin transport | 1/36 | 17/18866 | 0.031962147 | 0.071204239 | 0.034529744 | IL1B | 1 |
| GO:0019377 | glycolipid catabolic process | 1/36 | 17/18866 | 0.031962147 | 0.071204239 | 0.034529744 | PRKCD | 1 |
| GO:0030889 | negative regulation of B cell proliferation | 1/36 | 17/18866 | 0.031962147 | 0.071204239 | 0.034529744 | CASP3 | 1 |
| GO:0030949 | positive regulation of vascular endothelial growth factor receptor signaling pathway | 1/36 | 17/18866 | 0.031962147 | 0.071204239 | 0.034529744 | IL1B | 1 |
| GO:0031065 | positive regulation of histone deacetylation | 1/36 | 17/18866 | 0.031962147 | 0.071204239 | 0.034529744 | TP53 | 1 |
| GO:0032305 | positive regulation of icosanoid secretion | 1/36 | 17/18866 | 0.031962147 | 0.071204239 | 0.034529744 | IL1B | 1 |
| GO:0033599 | regulation of mammary gland epithelial cell proliferation | 1/36 | 17/18866 | 0.031962147 | 0.071204239 | 0.034529744 | BAX | 1 |
| GO:0043117 | positive regulation of vascular permeability | 1/36 | 17/18866 | 0.031962147 | 0.071204239 | 0.034529744 | PDE3A | 1 |
| GO:0043923 | positive regulation by host of viral transcription | 1/36 | 17/18866 | 0.031962147 | 0.071204239 | 0.034529744 | JUN | 1 |
| GO:0055012 | ventricular cardiac muscle cell differentiation | 1/36 | 17/18866 | 0.031962147 | 0.071204239 | 0.034529744 | CCNB1 | 1 |
| GO:0090141 | positive regulation of mitochondrial fission | 1/36 | 17/18866 | 0.031962147 | 0.071204239 | 0.034529744 | KDR | 1 |
| GO:1905331 | negative regulation of morphogenesis of an epithelium | 1/36 | 17/18866 | 0.031962147 | 0.071204239 | 0.034529744 | TNF | 1 |
| GO:2001267 | regulation of cysteine-type endopeptidase activity involved in apoptotic signaling pathway | 1/36 | 17/18866 | 0.031962147 | 0.071204239 | 0.034529744 | BAX | 1 |
| GO:0002449 | lymphocyte mediated immunity | 3/36 | 366/18866 | 0.032247707 | 0.071775268 | 0.034806658 | TNF/PRKCD/IL1B | 3 |
| GO:0008277 | regulation of G protein-coupled receptor signaling pathway | 2/36 | 148/18866 | 0.032357617 | 0.071889548 | 0.034862077 | PRKCA/OPRM1 | 2 |
| GO:0061337 | cardiac conduction | 2/36 | 148/18866 | 0.032357617 | 0.071889548 | 0.034862077 | SCN5A/KCNH2 | 2 |
| GO:0046394 | carboxylic acid biosynthetic process | 3/36 | 367/18866 | 0.032471641 | 0.072077649 | 0.034953294 | IL1B/FASN/EIF6 | 3 |
| GO:0016053 | organic acid biosynthetic process | 3/36 | 368/18866 | 0.032696401 | 0.072445545 | 0.035131702 | IL1B/FASN/EIF6 | 3 |
| GO:0030098 | lymphocyte differentiation | 3/36 | 368/18866 | 0.032696401 | 0.072445545 | 0.035131702 | TP53/IL1B/BAX | 3 |
| GO:0043524 | negative regulation of neuron apoptotic process | 2/36 | 149/18866 | 0.03275911 | 0.07251904 | 0.035167342 | JUN/BAX | 2 |
| GO:0002460 | adaptive immune response based on somatic recombination of immune receptors built from immunoglobulin superfamily domains | 3/36 | 370/18866 | 0.033148395 | 0.073214151 | 0.035504429 | TNF/PRKCD/IL1B | 3 |
| GO:0035264 | multicellular organism growth | 2/36 | 150/18866 | 0.033162581 | 0.073214151 | 0.035504429 | AR/TP53 | 2 |
| GO:0070665 | positive regulation of leukocyte proliferation | 2/36 | 150/18866 | 0.033162581 | 0.073214151 | 0.035504429 | IL1B/CDKN1A | 2 |
| GO:0008643 | carbohydrate transport | 2/36 | 151/18866 | 0.033568022 | 0.073568604 | 0.035676317 | TNF/IL1B | 2 |
| GO:0016331 | morphogenesis of embryonic epithelium | 2/36 | 151/18866 | 0.033568022 | 0.073568604 | 0.035676317 | AR/CASP3 | 2 |
| GO:0006677 | glycosylceramide metabolic process | 1/36 | 18/18866 | 0.033811018 | 0.073568604 | 0.035676317 | PRKCD | 1 |
| GO:0006740 | NADPH regeneration | 1/36 | 18/18866 | 0.033811018 | 0.073568604 | 0.035676317 | TP53 | 1 |
| GO:0010766 | negative regulation of sodium ion transport | 1/36 | 18/18866 | 0.033811018 | 0.073568604 | 0.035676317 | PRKCE | 1 |
| GO:0032780 | negative regulation of ATPase activity | 1/36 | 18/18866 | 0.033811018 | 0.073568604 | 0.035676317 | TP53 | 1 |
| GO:0033194 | response to hydroperoxide | 1/36 | 18/18866 | 0.033811018 | 0.073568604 | 0.035676317 | PRKCD | 1 |
| GO:0035994 | response to muscle stretch | 1/36 | 18/18866 | 0.033811018 | 0.073568604 | 0.035676317 | JUN | 1 |
| GO:0042136 | neurotransmitter biosynthetic process | 1/36 | 18/18866 | 0.033811018 | 0.073568604 | 0.035676317 | SLC6A4 | 1 |
| GO:0051023 | regulation of immunoglobulin secretion | 1/36 | 18/18866 | 0.033811018 | 0.073568604 | 0.035676317 | TNF | 1 |
| GO:0051782 | negative regulation of cell division | 1/36 | 18/18866 | 0.033811018 | 0.073568604 | 0.035676317 | MYC | 1 |
| GO:0052695 | cellular glucuronidation | 1/36 | 18/18866 | 0.033811018 | 0.073568604 | 0.035676317 | PRKCE | 1 |
| GO:0071605 | monocyte chemotactic protein-1 production | 1/36 | 18/18866 | 0.033811018 | 0.073568604 | 0.035676317 | IL1B | 1 |
| GO:0071637 | regulation of monocyte chemotactic protein-1 production | 1/36 | 18/18866 | 0.033811018 | 0.073568604 | 0.035676317 | IL1B | 1 |
| GO:1900221 | regulation of amyloid-beta clearance | 1/36 | 18/18866 | 0.033811018 | 0.073568604 | 0.035676317 | TNF | 1 |
| GO:1901524 | regulation of mitophagy | 1/36 | 18/18866 | 0.033811018 | 0.073568604 | 0.035676317 | TP53 | 1 |
| GO:1902903 | regulation of supramolecular fiber organization | 3/36 | 373/18866 | 0.033832568 | 0.073568604 | 0.035676317 | PRKCE/PRKCD/MAP2 | 3 |
| GO:0002224 | toll-like receptor signaling pathway | 2/36 | 152/18866 | 0.033975425 | 0.0737486 | 0.035763605 | ESR1/PRKCE | 2 |
| GO:1903169 | regulation of calcium ion transmembrane transport | 2/36 | 152/18866 | 0.033975425 | 0.0737486 | 0.035763605 | PRKCE/BAX | 2 |
| GO:0022412 | cellular process involved in reproduction in multicellular organism | 3/36 | 375/18866 | 0.0342928 | 0.074371752 | 0.036065796 | CCNB1/BAX/PDE3A | 3 |
| GO:0002822 | regulation of adaptive immune response based on somatic recombination of immune receptors built from immunoglobulin superfamily domains | 2/36 | 153/18866 | 0.03438478 | 0.074374128 | 0.036066947 | TNF/IL1B | 2 |
| GO:0008203 | cholesterol metabolic process | 2/36 | 153/18866 | 0.03438478 | 0.074374128 | 0.036066947 | FASN/PON1 | 2 |
| GO:0030048 | actin filament-based movement | 2/36 | 153/18866 | 0.03438478 | 0.074374128 | 0.036066947 | SCN5A/KCNH2 | 2 |
| GO:0002262 | myeloid cell homeostasis | 2/36 | 154/18866 | 0.03479608 | 0.074999452 | 0.036370192 | CASP3/BAX | 2 |
| GO:0002706 | regulation of lymphocyte mediated immunity | 2/36 | 154/18866 | 0.03479608 | 0.074999452 | 0.036370192 | TNF/IL1B | 2 |
| GO:0014065 | phosphatidylinositol 3-kinase signaling | 2/36 | 154/18866 | 0.03479608 | 0.074999452 | 0.036370192 | KDR/TNF | 2 |
| GO:0061351 | neural precursor cell proliferation | 2/36 | 154/18866 | 0.03479608 | 0.074999452 | 0.036370192 | TP53/SLC6A4 | 2 |
| GO:0000187 | activation of MAPK activity | 2/36 | 156/18866 | 0.035624479 | 0.075462584 | 0.036594783 | TNF/IL1B | 2 |
| GO:1902904 | negative regulation of supramolecular fiber organization | 2/36 | 156/18866 | 0.035624479 | 0.075462584 | 0.036594783 | PRKCD/MAP2 | 2 |
| GO:0006271 | DNA strand elongation involved in DNA replication | 1/36 | 19/18866 | 0.035656455 | 0.075462584 | 0.036594783 | PCNA | 1 |
| GO:0010544 | negative regulation of platelet activation | 1/36 | 19/18866 | 0.035656455 | 0.075462584 | 0.036594783 | PRKCD | 1 |
| GO:0010663 | positive regulation of striated muscle cell apoptotic process | 1/36 | 19/18866 | 0.035656455 | 0.075462584 | 0.036594783 | TP53 | 1 |
| GO:0010666 | positive regulation of cardiac muscle cell apoptotic process | 1/36 | 19/18866 | 0.035656455 | 0.075462584 | 0.036594783 | TP53 | 1 |
| GO:0032095 | regulation of response to food | 1/36 | 19/18866 | 0.035656455 | 0.075462584 | 0.036594783 | OPRM1 | 1 |
| GO:0032930 | positive regulation of superoxide anion generation | 1/36 | 19/18866 | 0.035656455 | 0.075462584 | 0.036594783 | PRKCD | 1 |
| GO:0045780 | positive regulation of bone resorption | 1/36 | 19/18866 | 0.035656455 | 0.075462584 | 0.036594783 | PRKCA | 1 |
| GO:0046485 | ether lipid metabolic process | 1/36 | 19/18866 | 0.035656455 | 0.075462584 | 0.036594783 | FASN | 1 |
| GO:0046852 | positive regulation of bone remodeling | 1/36 | 19/18866 | 0.035656455 | 0.075462584 | 0.036594783 | PRKCA | 1 |
| GO:0048490 | anterograde synaptic vesicle transport | 1/36 | 19/18866 | 0.035656455 | 0.075462584 | 0.036594783 | MAP2 | 1 |
| GO:0060602 | branch elongation of an epithelium | 1/36 | 19/18866 | 0.035656455 | 0.075462584 | 0.036594783 | ESR1 | 1 |
| GO:0071800 | podosome assembly | 1/36 | 19/18866 | 0.035656455 | 0.075462584 | 0.036594783 | TNF | 1 |
| GO:0072074 | kidney mesenchyme development | 1/36 | 19/18866 | 0.035656455 | 0.075462584 | 0.036594783 | MYC | 1 |
| GO:0097623 | potassium ion export across plasma membrane | 1/36 | 19/18866 | 0.035656455 | 0.075462584 | 0.036594783 | KCNH2 | 1 |
| GO:0098915 | membrane repolarization during ventricular cardiac muscle cell action potential | 1/36 | 19/18866 | 0.035656455 | 0.075462584 | 0.036594783 | KCNH2 | 1 |
| GO:0099514 | synaptic vesicle cytoskeletal transport | 1/36 | 19/18866 | 0.035656455 | 0.075462584 | 0.036594783 | MAP2 | 1 |
| GO:0099517 | synaptic vesicle transport along microtubule | 1/36 | 19/18866 | 0.035656455 | 0.075462584 | 0.036594783 | MAP2 | 1 |
| GO:2000010 | positive regulation of protein localization to cell surface | 1/36 | 19/18866 | 0.035656455 | 0.075462584 | 0.036594783 | TNF | 1 |
| GO:2000647 | negative regulation of stem cell proliferation | 1/36 | 19/18866 | 0.035656455 | 0.075462584 | 0.036594783 | TP53 | 1 |
| GO:0043271 | negative regulation of ion transport | 2/36 | 157/18866 | 0.036041562 | 0.076211916 | 0.036958164 | PRKCE/KCNH2 | 2 |
| GO:0001701 | in utero embryonic development | 3/36 | 383/18866 | 0.036166593 | 0.076344785 | 0.037022597 | AR/TP53/CCNB1 | 3 |
| GO:0032535 | regulation of cellular component size | 3/36 | 383/18866 | 0.036166593 | 0.076344785 | 0.037022597 | PRKCE/PRKCD/MAP2 | 3 |
| GO:0050729 | positive regulation of inflammatory response | 2/36 | 158/18866 | 0.036460555 | 0.076833188 | 0.037259443 | TNF/IL1B | 2 |
| GO:0060402 | calcium ion transport into cytosol | 2/36 | 158/18866 | 0.036460555 | 0.076833188 | 0.037259443 | PRKCE/BAX | 2 |
| GO:0031349 | positive regulation of defense response | 3/36 | 385/18866 | 0.03664324 | 0.077151934 | 0.037414016 | TNF/PRKCD/IL1B | 3 |
| GO:0016573 | histone acetylation | 2/36 | 159/18866 | 0.036881451 | 0.077490516 | 0.037578207 | CHEK1/IL1B | 2 |
| GO:0032675 | regulation of interleukin-6 production | 2/36 | 159/18866 | 0.036881451 | 0.077490516 | 0.037578207 | TNF/IL1B | 2 |
| GO:0002544 | chronic inflammatory response | 1/36 | 20/18866 | 0.037498466 | 0.077490516 | 0.037578207 | TNF | 1 |
| GO:0002726 | positive regulation of T cell cytokine production | 1/36 | 20/18866 | 0.037498466 | 0.077490516 | 0.037578207 | IL1B | 1 |
| GO:0002827 | positive regulation of T-helper 1 type immune response | 1/36 | 20/18866 | 0.037498466 | 0.077490516 | 0.037578207 | IL1B | 1 |
| GO:0002902 | regulation of B cell apoptotic process | 1/36 | 20/18866 | 0.037498466 | 0.077490516 | 0.037578207 | BAX | 1 |
| GO:0007252 | I-kappaB phosphorylation | 1/36 | 20/18866 | 0.037498466 | 0.077490516 | 0.037578207 | TNF | 1 |
| GO:0007620 | copulation | 1/36 | 20/18866 | 0.037498466 | 0.077490516 | 0.037578207 | SLC6A4 | 1 |
| GO:0010875 | positive regulation of cholesterol efflux | 1/36 | 20/18866 | 0.037498466 | 0.077490516 | 0.037578207 | PON1 | 1 |
| GO:0015669 | gas transport | 1/36 | 20/18866 | 0.037498466 | 0.077490516 | 0.037578207 | MYC | 1 |
| GO:0032303 | regulation of icosanoid secretion | 1/36 | 20/18866 | 0.037498466 | 0.077490516 | 0.037578207 | IL1B | 1 |
| GO:0032986 | protein-DNA complex disassembly | 1/36 | 20/18866 | 0.037498466 | 0.077490516 | 0.037578207 | MYC | 1 |
| GO:0043950 | positive regulation of cAMP-mediated signaling | 1/36 | 20/18866 | 0.037498466 | 0.077490516 | 0.037578207 | PRKCA | 1 |
| GO:0051412 | response to corticosterone | 1/36 | 20/18866 | 0.037498466 | 0.077490516 | 0.037578207 | CDKN1A | 1 |
| GO:0051767 | nitric-oxide synthase biosynthetic process | 1/36 | 20/18866 | 0.037498466 | 0.077490516 | 0.037578207 | KDR | 1 |
| GO:0051769 | regulation of nitric-oxide synthase biosynthetic process | 1/36 | 20/18866 | 0.037498466 | 0.077490516 | 0.037578207 | KDR | 1 |
| GO:0060353 | regulation of cell adhesion molecule production | 1/36 | 20/18866 | 0.037498466 | 0.077490516 | 0.037578207 | IL1B | 1 |
| GO:0086014 | atrial cardiac muscle cell action potential | 1/36 | 20/18866 | 0.037498466 | 0.077490516 | 0.037578207 | SCN5A | 1 |
| GO:0086026 | atrial cardiac muscle cell to AV node cell signaling | 1/36 | 20/18866 | 0.037498466 | 0.077490516 | 0.037578207 | SCN5A | 1 |
| GO:0086066 | atrial cardiac muscle cell to AV node cell communication | 1/36 | 20/18866 | 0.037498466 | 0.077490516 | 0.037578207 | SCN5A | 1 |
| GO:1900409 | positive regulation of cellular response to oxidative stress | 1/36 | 20/18866 | 0.037498466 | 0.077490516 | 0.037578207 | TNF | 1 |
| GO:2000193 | positive regulation of fatty acid transport | 1/36 | 20/18866 | 0.037498466 | 0.077490516 | 0.037578207 | IL1B | 1 |
| GO:0000723 | telomere maintenance | 2/36 | 161/18866 | 0.037728917 | 0.077835707 | 0.037745604 | PCNA/MYC | 2 |
| GO:0034767 | positive regulation of ion transmembrane transport | 2/36 | 161/18866 | 0.037728917 | 0.077835707 | 0.037745604 | BAX/KCNH2 | 2 |
| GO:0030900 | forebrain development | 3/36 | 391/18866 | 0.038092803 | 0.07852043 | 0.038077653 | SCN5A/CASP3/BAX | 3 |
| GO:1902652 | secondary alcohol metabolic process | 2/36 | 162/18866 | 0.03815547 | 0.078583623 | 0.038108298 | FASN/PON1 | 2 |
| GO:0051494 | negative regulation of cytoskeleton organization | 2/36 | 163/18866 | 0.038583892 | 0.079399376 | 0.038503889 | PRKCD/MAP2 | 2 |
| GO:0018393 | internal peptidyl-lysine acetylation | 2/36 | 164/18866 | 0.039014176 | 0.079811959 | 0.038703966 | CHEK1/IL1B | 2 |
| GO:0030198 | extracellular matrix organization | 3/36 | 395/18866 | 0.039075489 | 0.079811959 | 0.038703966 | PRSS1/KDR/TNF | 3 |
| GO:0043062 | extracellular structure organization | 3/36 | 396/18866 | 0.039323194 | 0.079811959 | 0.038703966 | PRSS1/KDR/TNF | 3 |
| GO:0006662 | glycerol ether metabolic process | 1/36 | 21/18866 | 0.039337055 | 0.079811959 | 0.038703966 | FASN | 1 |
| GO:0006837 | serotonin transport | 1/36 | 21/18866 | 0.039337055 | 0.079811959 | 0.038703966 | SLC6A4 | 1 |
| GO:0021854 | hypothalamus development | 1/36 | 21/18866 | 0.039337055 | 0.079811959 | 0.038703966 | BAX | 1 |
| GO:0030220 | platelet formation | 1/36 | 21/18866 | 0.039337055 | 0.079811959 | 0.038703966 | CASP3 | 1 |
| GO:0030728 | ovulation | 1/36 | 21/18866 | 0.039337055 | 0.079811959 | 0.038703966 | PGR | 1 |
| GO:0042276 | error-prone translesion synthesis | 1/36 | 21/18866 | 0.039337055 | 0.079811959 | 0.038703966 | PCNA | 1 |
| GO:0046514 | ceramide catabolic process | 1/36 | 21/18866 | 0.039337055 | 0.079811959 | 0.038703966 | PRKCD | 1 |
| GO:0046823 | negative regulation of nucleocytoplasmic transport | 1/36 | 21/18866 | 0.039337055 | 0.079811959 | 0.038703966 | PKIA | 1 |
| GO:0048305 | immunoglobulin secretion | 1/36 | 21/18866 | 0.039337055 | 0.079811959 | 0.038703966 | TNF | 1 |
| GO:0051797 | regulation of hair follicle development | 1/36 | 21/18866 | 0.039337055 | 0.079811959 | 0.038703966 | TNF | 1 |
| GO:0060065 | uterus development | 1/36 | 21/18866 | 0.039337055 | 0.079811959 | 0.038703966 | ESR1 | 1 |
| GO:0072111 | cell proliferation involved in kidney development | 1/36 | 21/18866 | 0.039337055 | 0.079811959 | 0.038703966 | MYC | 1 |
| GO:0090312 | positive regulation of protein deacetylation | 1/36 | 21/18866 | 0.039337055 | 0.079811959 | 0.038703966 | TP53 | 1 |
| GO:2000269 | regulation of fibroblast apoptotic process | 1/36 | 21/18866 | 0.039337055 | 0.079811959 | 0.038703966 | TP53 | 1 |
| GO:1901653 | cellular response to peptide | 3/36 | 398/18866 | 0.039821043 | 0.080647543 | 0.039109174 | TP53/PRKCD/IL1B | 3 |
| GO:0006475 | internal protein amino acid acetylation | 2/36 | 166/18866 | 0.039880292 | 0.080647543 | 0.039109174 | CHEK1/IL1B | 2 |
| GO:1903531 | negative regulation of secretion by cell | 2/36 | 166/18866 | 0.039880292 | 0.080647543 | 0.039109174 | IL1B/OPRM1 | 2 |
| GO:2001242 | regulation of intrinsic apoptotic signaling pathway | 2/36 | 166/18866 | 0.039880292 | 0.080647543 | 0.039109174 | TP53/BAX | 2 |
| GO:0042129 | regulation of T cell proliferation | 2/36 | 167/18866 | 0.040316109 | 0.081461768 | 0.039504024 | IL1B/CASP3 | 2 |
| GO:0002819 | regulation of adaptive immune response | 2/36 | 168/18866 | 0.040753754 | 0.082218987 | 0.039871229 | TNF/IL1B | 2 |
| GO:0009235 | cobalamin metabolic process | 1/36 | 22/18866 | 0.04117223 | 0.082218987 | 0.039871229 | PRSS1 | 1 |
| GO:0010888 | negative regulation of lipid storage | 1/36 | 22/18866 | 0.04117223 | 0.082218987 | 0.039871229 | TNF | 1 |
| GO:0032928 | regulation of superoxide anion generation | 1/36 | 22/18866 | 0.04117223 | 0.082218987 | 0.039871229 | PRKCD | 1 |
| GO:0035584 | calcium-mediated signaling using intracellular calcium source | 1/36 | 22/18866 | 0.04117223 | 0.082218987 | 0.039871229 | KDR | 1 |
| GO:0036344 | platelet morphogenesis | 1/36 | 22/18866 | 0.04117223 | 0.082218987 | 0.039871229 | CASP3 | 1 |
| GO:0051000 | positive regulation of nitric-oxide synthase activity | 1/36 | 22/18866 | 0.04117223 | 0.082218987 | 0.039871229 | TNF | 1 |
| GO:0051900 | regulation of mitochondrial depolarization | 1/36 | 22/18866 | 0.04117223 | 0.082218987 | 0.039871229 | KDR | 1 |
| GO:0055093 | response to hyperoxia | 1/36 | 22/18866 | 0.04117223 | 0.082218987 | 0.039871229 | CDKN1A | 1 |
| GO:0060352 | cell adhesion molecule production | 1/36 | 22/18866 | 0.04117223 | 0.082218987 | 0.039871229 | IL1B | 1 |
| GO:0070987 | error-free translesion synthesis | 1/36 | 22/18866 | 0.04117223 | 0.082218987 | 0.039871229 | PCNA | 1 |
| GO:0071379 | cellular response to prostaglandin stimulus | 1/36 | 22/18866 | 0.04117223 | 0.082218987 | 0.039871229 | PRKCE | 1 |
| GO:1902884 | positive regulation of response to oxidative stress | 1/36 | 22/18866 | 0.04117223 | 0.082218987 | 0.039871229 | TNF | 1 |
| GO:2000479 | regulation of cAMP-dependent protein kinase activity | 1/36 | 22/18866 | 0.04117223 | 0.082218987 | 0.039871229 | PKIA | 1 |
| GO:0016125 | sterol metabolic process | 2/36 | 169/18866 | 0.04119322 | 0.082218987 | 0.039871229 | FASN/PON1 | 2 |
| GO:0032970 | regulation of actin filament-based process | 3/36 | 405/18866 | 0.041589043 | 0.082897558 | 0.040200295 | SCN5A/PRKCE/PRKCD | 3 |
| GO:0032635 | interleukin-6 production | 2/36 | 170/18866 | 0.041634497 | 0.082897558 | 0.040200295 | TNF/IL1B | 2 |
| GO:0032874 | positive regulation of stress-activated MAPK cascade | 2/36 | 170/18866 | 0.041634497 | 0.082897558 | 0.040200295 | TNF/IL1B | 2 |
| GO:0007584 | response to nutrient | 2/36 | 171/18866 | 0.042077578 | 0.083392425 | 0.040440275 | F7/SLC6A4 | 2 |
| GO:0030856 | regulation of epithelial cell differentiation | 2/36 | 171/18866 | 0.042077578 | 0.083392425 | 0.040440275 | TNF/IL1B | 2 |
| GO:0002244 | hematopoietic progenitor cell differentiation | 2/36 | 172/18866 | 0.042522454 | 0.083392425 | 0.040440275 | KDR/TP53 | 2 |
| GO:0018394 | peptidyl-lysine acetylation | 2/36 | 172/18866 | 0.042522454 | 0.083392425 | 0.040440275 | CHEK1/IL1B | 2 |
| GO:0050714 | positive regulation of protein secretion | 2/36 | 172/18866 | 0.042522454 | 0.083392425 | 0.040440275 | TNF/PRKCE | 2 |
| GO:0050806 | positive regulation of synaptic transmission | 2/36 | 172/18866 | 0.042522454 | 0.083392425 | 0.040440275 | TNF/PRKCE | 2 |
| GO:0070304 | positive regulation of stress-activated protein kinase signaling cascade | 2/36 | 172/18866 | 0.042522454 | 0.083392425 | 0.040440275 | TNF/IL1B | 2 |
| GO:0002831 | regulation of response to biotic stimulus | 3/36 | 409/18866 | 0.042617105 | 0.083392425 | 0.040440275 | PRKCD/PRKCA/IL1B | 3 |
| GO:0006937 | regulation of muscle contraction | 2/36 | 173/18866 | 0.042969119 | 0.083392425 | 0.040440275 | SCN5A/CHRM3 | 2 |
| GO:0021543 | pallium development | 2/36 | 173/18866 | 0.042969119 | 0.083392425 | 0.040440275 | CASP3/BAX | 2 |
| GO:0033209 | tumor necrosis factor-mediated signaling pathway | 2/36 | 173/18866 | 0.042969119 | 0.083392425 | 0.040440275 | TP53/TNF | 2 |
| GO:0000002 | mitochondrial genome maintenance | 1/36 | 23/18866 | 0.043003996 | 0.083392425 | 0.040440275 | TP53 | 1 |
| GO:0006063 | uronic acid metabolic process | 1/36 | 23/18866 | 0.043003996 | 0.083392425 | 0.040440275 | PRKCE | 1 |
| GO:0006297 | nucleotide-excision repair, DNA gap filling | 1/36 | 23/18866 | 0.043003996 | 0.083392425 | 0.040440275 | PCNA | 1 |
| GO:0006359 | regulation of transcription by RNA polymerase III | 1/36 | 23/18866 | 0.043003996 | 0.083392425 | 0.040440275 | AR | 1 |
| GO:0007413 | axonal fasciculation | 1/36 | 23/18866 | 0.043003996 | 0.083392425 | 0.040440275 | CASP3 | 1 |
| GO:0019585 | glucuronate metabolic process | 1/36 | 23/18866 | 0.043003996 | 0.083392425 | 0.040440275 | PRKCE | 1 |
| GO:0030539 | male genitalia development | 1/36 | 23/18866 | 0.043003996 | 0.083392425 | 0.040440275 | AR | 1 |
| GO:0032098 | regulation of appetite | 1/36 | 23/18866 | 0.043003996 | 0.083392425 | 0.040440275 | OPRM1 | 1 |
| GO:0035162 | embryonic hemopoiesis | 1/36 | 23/18866 | 0.043003996 | 0.083392425 | 0.040440275 | KDR | 1 |
| GO:0035743 | CD4-positive, alpha-beta T cell cytokine production | 1/36 | 23/18866 | 0.043003996 | 0.083392425 | 0.040440275 | IL1B | 1 |
| GO:0045649 | regulation of macrophage differentiation | 1/36 | 23/18866 | 0.043003996 | 0.083392425 | 0.040440275 | PRKCA | 1 |
| GO:0045723 | positive regulation of fatty acid biosynthetic process | 1/36 | 23/18866 | 0.043003996 | 0.083392425 | 0.040440275 | IL1B | 1 |
| GO:0060445 | branching involved in salivary gland morphogenesis | 1/36 | 23/18866 | 0.043003996 | 0.083392425 | 0.040440275 | TNF | 1 |
| GO:0070584 | mitochondrion morphogenesis | 1/36 | 23/18866 | 0.043003996 | 0.083392425 | 0.040440275 | BAX | 1 |
| GO:0072215 | regulation of metanephros development | 1/36 | 23/18866 | 0.043003996 | 0.083392425 | 0.040440275 | MYC | 1 |
| GO:0086012 | membrane depolarization during cardiac muscle cell action potential | 1/36 | 23/18866 | 0.043003996 | 0.083392425 | 0.040440275 | SCN5A | 1 |
| GO:0090343 | positive regulation of cell aging | 1/36 | 23/18866 | 0.043003996 | 0.083392425 | 0.040440275 | TP53 | 1 |
| GO:0106030 | neuron projection fasciculation | 1/36 | 23/18866 | 0.043003996 | 0.083392425 | 0.040440275 | CASP3 | 1 |
| GO:1900017 | positive regulation of cytokine production involved in inflammatory response | 1/36 | 23/18866 | 0.043003996 | 0.083392425 | 0.040440275 | TNF | 1 |
| GO:1901522 | positive regulation of transcription from RNA polymerase II promoter involved in cellular response to chemical stimulus | 1/36 | 23/18866 | 0.043003996 | 0.083392425 | 0.040440275 | TP53 | 1 |
| GO:1904996 | positive regulation of leukocyte adhesion to vascular endothelial cell | 1/36 | 23/18866 | 0.043003996 | 0.083392425 | 0.040440275 | TNF | 1 |
| GO:2000810 | regulation of bicellular tight junction assembly | 1/36 | 23/18866 | 0.043003996 | 0.083392425 | 0.040440275 | TNF | 1 |
| GO:0001659 | temperature homeostasis | 2/36 | 174/18866 | 0.043417563 | 0.083863193 | 0.040668569 | TNF/IL1B | 2 |
| GO:0003205 | cardiac chamber development | 2/36 | 174/18866 | 0.043417563 | 0.083863193 | 0.040668569 | SCN5A/TP53 | 2 |
| GO:0015718 | monocarboxylic acid transport | 2/36 | 174/18866 | 0.043417563 | 0.083863193 | 0.040668569 | NCOA2/IL1B | 2 |
| GO:0030833 | regulation of actin filament polymerization | 2/36 | 174/18866 | 0.043417563 | 0.083863193 | 0.040668569 | PRKCE/PRKCD | 2 |
| GO:0032200 | telomere organization | 2/36 | 174/18866 | 0.043417563 | 0.083863193 | 0.040668569 | PCNA/MYC | 2 |
| GO:0050680 | negative regulation of epithelial cell proliferation | 2/36 | 175/18866 | 0.043867779 | 0.084666194 | 0.041057976 | AR/TNF | 2 |
| GO:0030324 | lung development | 2/36 | 177/18866 | 0.044773495 | 0.085786003 | 0.041601016 | TNF/PGR | 2 |
| GO:0001759 | organ induction | 1/36 | 24/18866 | 0.044832359 | 0.085786003 | 0.041601016 | AR | 1 |
| GO:0002438 | acute inflammatory response to antigenic stimulus | 1/36 | 24/18866 | 0.044832359 | 0.085786003 | 0.041601016 | OPRM1 | 1 |
| GO:0002719 | negative regulation of cytokine production involved in immune response | 1/36 | 24/18866 | 0.044832359 | 0.085786003 | 0.041601016 | TNF | 1 |
| GO:0007095 | mitotic G2 DNA damage checkpoint | 1/36 | 24/18866 | 0.044832359 | 0.085786003 | 0.041601016 | CDKN1A | 1 |
| GO:0010640 | regulation of platelet-derived growth factor receptor signaling pathway | 1/36 | 24/18866 | 0.044832359 | 0.085786003 | 0.041601016 | F7 | 1 |
| GO:0032469 | endoplasmic reticulum calcium ion homeostasis | 1/36 | 24/18866 | 0.044832359 | 0.085786003 | 0.041601016 | BAX | 1 |
| GO:0034695 | response to prostaglandin E | 1/36 | 24/18866 | 0.044832359 | 0.085786003 | 0.041601016 | PRKCE | 1 |
| GO:0036003 | positive regulation of transcription from RNA polymerase II promoter in response to stress | 1/36 | 24/18866 | 0.044832359 | 0.085786003 | 0.041601016 | TP53 | 1 |
| GO:0051882 | mitochondrial depolarization | 1/36 | 24/18866 | 0.044832359 | 0.085786003 | 0.041601016 | KDR | 1 |
| GO:2000637 | positive regulation of gene silencing by miRNA | 1/36 | 24/18866 | 0.044832359 | 0.085786003 | 0.041601016 | TP53 | 1 |
| GO:0006941 | striated muscle contraction | 2/36 | 178/18866 | 0.045228978 | 0.086410227 | 0.041903727 | SCN5A/KCNH2 | 2 |
| GO:0060401 | cytosolic calcium ion transport | 2/36 | 178/18866 | 0.045228978 | 0.086410227 | 0.041903727 | PRKCE/BAX | 2 |
| GO:1901796 | regulation of signal transduction by p53 class mediator | 2/36 | 180/18866 | 0.046145157 | 0.088042842 | 0.042695446 | CHEK1/TP53 | 2 |
| GO:0030323 | respiratory tube development | 2/36 | 181/18866 | 0.046605837 | 0.088042842 | 0.042695446 | TNF/PGR | 2 |
| GO:0000423 | mitophagy | 1/36 | 25/18866 | 0.046657327 | 0.088042842 | 0.042695446 | TP53 | 1 |
| GO:0002053 | positive regulation of mesenchymal cell proliferation | 1/36 | 25/18866 | 0.046657327 | 0.088042842 | 0.042695446 | MYC | 1 |
| GO:0007530 | sex determination | 1/36 | 25/18866 | 0.046657327 | 0.088042842 | 0.042695446 | AR | 1 |
| GO:0014829 | vascular associated smooth muscle contraction | 1/36 | 25/18866 | 0.046657327 | 0.088042842 | 0.042695446 | CHRM3 | 1 |
| GO:0032104 | regulation of response to extracellular stimulus | 1/36 | 25/18866 | 0.046657327 | 0.088042842 | 0.042695446 | OPRM1 | 1 |
| GO:0032107 | regulation of response to nutrient levels | 1/36 | 25/18866 | 0.046657327 | 0.088042842 | 0.042695446 | OPRM1 | 1 |
| GO:0045662 | negative regulation of myoblast differentiation | 1/36 | 25/18866 | 0.046657327 | 0.088042842 | 0.042695446 | TNF | 1 |
| GO:0051894 | positive regulation of focal adhesion assembly | 1/36 | 25/18866 | 0.046657327 | 0.088042842 | 0.042695446 | KDR | 1 |
| GO:0060148 | positive regulation of posttranscriptional gene silencing | 1/36 | 25/18866 | 0.046657327 | 0.088042842 | 0.042695446 | TP53 | 1 |
| GO:0060571 | morphogenesis of an epithelial fold | 1/36 | 25/18866 | 0.046657327 | 0.088042842 | 0.042695446 | AR | 1 |
| GO:0060575 | intestinal epithelial cell differentiation | 1/36 | 25/18866 | 0.046657327 | 0.088042842 | 0.042695446 | CDKN1A | 1 |
| GO:0071677 | positive regulation of mononuclear cell migration | 1/36 | 25/18866 | 0.046657327 | 0.088042842 | 0.042695446 | TNF | 1 |
| GO:1904385 | cellular response to angiotensin | 1/36 | 25/18866 | 0.046657327 | 0.088042842 | 0.042695446 | PRKCD | 1 |
| GO:2001026 | regulation of endothelial cell chemotaxis | 1/36 | 25/18866 | 0.046657327 | 0.088042842 | 0.042695446 | KDR | 1 |
| GO:0071346 | cellular response to interferon-gamma | 2/36 | 182/18866 | 0.047068233 | 0.08875001 | 0.04303838 | TP53/PRKCD | 2 |
| GO:0006816 | calcium ion transport | 3/36 | 426/18866 | 0.047129546 | 0.088797418 | 0.04306137 | PRKCE/BAX/OPRM1 | 3 |
| GO:0038061 | NIK/NF-kappaB signaling | 2/36 | 184/18866 | 0.047998144 | 0.090163416 | 0.043723796 | TNF/IL1B | 2 |
| GO:0046328 | regulation of JNK cascade | 2/36 | 184/18866 | 0.047998144 | 0.090163416 | 0.043723796 | TNF/IL1B | 2 |
| GO:0001783 | B cell apoptotic process | 1/36 | 26/18866 | 0.048478904 | 0.090163416 | 0.043723796 | BAX | 1 |
| GO:0002068 | glandular epithelial cell development | 1/36 | 26/18866 | 0.048478904 | 0.090163416 | 0.043723796 | FASN | 1 |
| GO:0018904 | ether metabolic process | 1/36 | 26/18866 | 0.048478904 | 0.090163416 | 0.043723796 | FASN | 1 |
| GO:0019430 | removal of superoxide radicals | 1/36 | 26/18866 | 0.048478904 | 0.090163416 | 0.043723796 | TNF | 1 |
| GO:0022616 | DNA strand elongation | 1/36 | 26/18866 | 0.048478904 | 0.090163416 | 0.043723796 | PCNA | 1 |
| GO:0031664 | regulation of lipopolysaccharide-mediated signaling pathway | 1/36 | 26/18866 | 0.048478904 | 0.090163416 | 0.043723796 | PRKCA | 1 |
| GO:0032743 | positive regulation of interleukin-2 production | 1/36 | 26/18866 | 0.048478904 | 0.090163416 | 0.043723796 | IL1B | 1 |
| GO:0035640 | exploration behavior | 1/36 | 26/18866 | 0.048478904 | 0.090163416 | 0.043723796 | PRKCE | 1 |
| GO:0043567 | regulation of insulin-like growth factor receptor signaling pathway | 1/36 | 26/18866 | 0.048478904 | 0.090163416 | 0.043723796 | AR | 1 |
| GO:0051156 | glucose 6-phosphate metabolic process | 1/36 | 26/18866 | 0.048478904 | 0.090163416 | 0.043723796 | TP53 | 1 |
| GO:0060561 | apoptotic process involved in morphogenesis | 1/36 | 26/18866 | 0.048478904 | 0.090163416 | 0.043723796 | BAX | 1 |
| GO:0097066 | response to thyroid hormone | 1/36 | 26/18866 | 0.048478904 | 0.090163416 | 0.043723796 | F7 | 1 |
| GO:1900739 | regulation of protein insertion into mitochondrial membrane involved in apoptotic signaling pathway | 1/36 | 26/18866 | 0.048478904 | 0.090163416 | 0.043723796 | TP53 | 1 |
| GO:1900740 | positive regulation of protein insertion into mitochondrial membrane involved in apoptotic signaling pathway | 1/36 | 26/18866 | 0.048478904 | 0.090163416 | 0.043723796 | TP53 | 1 |
| GO:1903649 | regulation of cytoplasmic transport | 1/36 | 26/18866 | 0.048478904 | 0.090163416 | 0.043723796 | MAP2 | 1 |
| GO:0051656 | establishment of organelle localization | 3/36 | 432/18866 | 0.048777096 | 0.090649334 | 0.043959437 | EIF6/CCNB1/MAP2 | 3 |
| GO:0043488 | regulation of mRNA stability | 2/36 | 186/18866 | 0.048934827 | 0.090873676 | 0.044068229 | PRKCD/PRKCA | 2 |
| GO:0052548 | regulation of endopeptidase activity | 3/36 | 434/18866 | 0.049332599 | 0.091543106 | 0.044392862 | TNF/MYC/BAX | 3 |
| GO:0031345 | negative regulation of cell projection organization | 2/36 | 188/18866 | 0.049878219 | 0.092485671 | 0.044849949 | PRKCD/MAP2 | 2 |
| GO:0001963 | synaptic transmission, dopaminergic | 1/36 | 27/18866 | 0.050297097 | 0.092563248 | 0.04488757 | SLC6A4 | 1 |
| GO:0002825 | regulation of T-helper 1 type immune response | 1/36 | 27/18866 | 0.050297097 | 0.092563248 | 0.04488757 | IL1B | 1 |
| GO:0007214 | gamma-aminobutyric acid signaling pathway | 1/36 | 27/18866 | 0.050297097 | 0.092563248 | 0.04488757 | GABRA1 | 1 |
| GO:0008053 | mitochondrial fusion | 1/36 | 27/18866 | 0.050297097 | 0.092563248 | 0.04488757 | BAX | 1 |
| GO:0010971 | positive regulation of G2/M transition of mitotic cell cycle | 1/36 | 27/18866 | 0.050297097 | 0.092563248 | 0.04488757 | CCNB1 | 1 |
| GO:0032201 | telomere maintenance via semi-conservative replication | 1/36 | 27/18866 | 0.050297097 | 0.092563248 | 0.04488757 | PCNA | 1 |
| GO:0032897 | negative regulation of viral transcription | 1/36 | 27/18866 | 0.050297097 | 0.092563248 | 0.04488757 | JUN | 1 |
| GO:0033081 | regulation of T cell differentiation in thymus | 1/36 | 27/18866 | 0.050297097 | 0.092563248 | 0.04488757 | IL1B | 1 |
| GO:0045672 | positive regulation of osteoclast differentiation | 1/36 | 27/18866 | 0.050297097 | 0.092563248 | 0.04488757 | TNF | 1 |
| GO:0090140 | regulation of mitochondrial fission | 1/36 | 27/18866 | 0.050297097 | 0.092563248 | 0.04488757 | KDR | 1 |
| GO:0008064 | regulation of actin polymerization or depolymerization | 2/36 | 190/18866 | 0.050828261 | 0.093330875 | 0.045259822 | PRKCE/PRKCD | 2 |
| GO:0051048 | negative regulation of secretion | 2/36 | 190/18866 | 0.050828261 | 0.093330875 | 0.045259822 | IL1B/OPRM1 | 2 |
| GO:0070507 | regulation of microtubule cytoskeleton organization | 2/36 | 190/18866 | 0.050828261 | 0.093330875 | 0.045259822 | CHEK1/MAP2 | 2 |
| GO:0022408 | negative regulation of cell-cell adhesion | 2/36 | 191/18866 | 0.051305755 | 0.094066938 | 0.045616768 | PRKCD/CASP3 | 2 |
| GO:0030832 | regulation of actin filament length | 2/36 | 191/18866 | 0.051305755 | 0.094066938 | 0.045616768 | PRKCE/PRKCD | 2 |
| GO:0006413 | translational initiation | 2/36 | 192/18866 | 0.051784889 | 0.094138885 | 0.045651658 | TNF/EIF6 | 2 |
| GO:0048015 | phosphatidylinositol-mediated signaling | 2/36 | 192/18866 | 0.051784889 | 0.094138885 | 0.045651658 | KDR/TNF | 2 |
| GO:0000470 | maturation of LSU-rRNA | 1/36 | 28/18866 | 0.052111912 | 0.094138885 | 0.045651658 | EIF6 | 1 |
| GO:0002360 | T cell lineage commitment | 1/36 | 28/18866 | 0.052111912 | 0.094138885 | 0.045651658 | TP53 | 1 |
| GO:0010575 | positive regulation of vascular endothelial growth factor production | 1/36 | 28/18866 | 0.052111912 | 0.094138885 | 0.045651658 | IL1B | 1 |
| GO:0030194 | positive regulation of blood coagulation | 1/36 | 28/18866 | 0.052111912 | 0.094138885 | 0.045651658 | F7 | 1 |
| GO:0031440 | regulation of mRNA 3'-end processing | 1/36 | 28/18866 | 0.052111912 | 0.094138885 | 0.045651658 | CCNB1 | 1 |
| GO:0032800 | receptor biosynthetic process | 1/36 | 28/18866 | 0.052111912 | 0.094138885 | 0.045651658 | TNF | 1 |
| GO:0045745 | positive regulation of G protein-coupled receptor signaling pathway | 1/36 | 28/18866 | 0.052111912 | 0.094138885 | 0.045651658 | PRKCA | 1 |
| GO:0071168 | protein localization to chromatin | 1/36 | 28/18866 | 0.052111912 | 0.094138885 | 0.045651658 | ESR1 | 1 |
| GO:0071450 | cellular response to oxygen radical | 1/36 | 28/18866 | 0.052111912 | 0.094138885 | 0.045651658 | TNF | 1 |
| GO:0071451 | cellular response to superoxide | 1/36 | 28/18866 | 0.052111912 | 0.094138885 | 0.045651658 | TNF | 1 |
| GO:0072376 | protein activation cascade | 1/36 | 28/18866 | 0.052111912 | 0.094138885 | 0.045651658 | F7 | 1 |
| GO:0072378 | blood coagulation, fibrin clot formation | 1/36 | 28/18866 | 0.052111912 | 0.094138885 | 0.045651658 | F7 | 1 |
| GO:0086013 | membrane repolarization during cardiac muscle cell action potential | 1/36 | 28/18866 | 0.052111912 | 0.094138885 | 0.045651658 | KCNH2 | 1 |
| GO:1900048 | positive regulation of hemostasis | 1/36 | 28/18866 | 0.052111912 | 0.094138885 | 0.045651658 | F7 | 1 |
| GO:1900101 | regulation of endoplasmic reticulum unfolded protein response | 1/36 | 28/18866 | 0.052111912 | 0.094138885 | 0.045651658 | BAX | 1 |
| GO:1903579 | negative regulation of ATP metabolic process | 1/36 | 28/18866 | 0.052111912 | 0.094138885 | 0.045651658 | TP53 | 1 |
| GO:1990776 | response to angiotensin | 1/36 | 28/18866 | 0.052111912 | 0.094138885 | 0.045651658 | PRKCD | 1 |
| GO:2000191 | regulation of fatty acid transport | 1/36 | 28/18866 | 0.052111912 | 0.094138885 | 0.045651658 | IL1B | 1 |
| GO:0002793 | positive regulation of peptide secretion | 2/36 | 193/18866 | 0.052265655 | 0.094277871 | 0.045719058 | TNF/PRKCE | 2 |
| GO:0030041 | actin filament polymerization | 2/36 | 193/18866 | 0.052265655 | 0.094277871 | 0.045719058 | PRKCE/PRKCD | 2 |
| GO:0043487 | regulation of RNA stability | 2/36 | 195/18866 | 0.053232051 | 0.095809886 | 0.046461992 | PRKCD/PRKCA | 2 |
| GO:0050731 | positive regulation of peptidyl-tyrosine phosphorylation | 2/36 | 195/18866 | 0.053232051 | 0.095809886 | 0.046461992 | TP53/TNF | 2 |
| GO:0050864 | regulation of B cell activation | 2/36 | 195/18866 | 0.053232051 | 0.095809886 | 0.046461992 | CDKN1A/CASP3 | 2 |
| GO:0009566 | fertilization | 2/36 | 196/18866 | 0.053717666 | 0.095928868 | 0.046519691 | AR/BAX | 2 |
| GO:0048017 | inositol lipid-mediated signaling | 2/36 | 196/18866 | 0.053717666 | 0.095928868 | 0.046519691 | KDR/TNF | 2 |
| GO:0008209 | androgen metabolic process | 1/36 | 29/18866 | 0.053923356 | 0.095928868 | 0.046519691 | ESR1 | 1 |
| GO:0015721 | bile acid and bile salt transport | 1/36 | 29/18866 | 0.053923356 | 0.095928868 | 0.046519691 | NCOA2 | 1 |
| GO:0031063 | regulation of histone deacetylation | 1/36 | 29/18866 | 0.053923356 | 0.095928868 | 0.046519691 | TP53 | 1 |
| GO:0032373 | positive regulation of sterol transport | 1/36 | 29/18866 | 0.053923356 | 0.095928868 | 0.046519691 | PON1 | 1 |
| GO:0032376 | positive regulation of cholesterol transport | 1/36 | 29/18866 | 0.053923356 | 0.095928868 | 0.046519691 | PON1 | 1 |
| GO:0036296 | response to increased oxygen levels | 1/36 | 29/18866 | 0.053923356 | 0.095928868 | 0.046519691 | CDKN1A | 1 |
| GO:0042634 | regulation of hair cycle | 1/36 | 29/18866 | 0.053923356 | 0.095928868 | 0.046519691 | TNF | 1 |
| GO:0043153 | entrainment of circadian clock by photoperiod | 1/36 | 29/18866 | 0.053923356 | 0.095928868 | 0.046519691 | TP53 | 1 |
| GO:0048873 | homeostasis of number of cells within a tissue | 1/36 | 29/18866 | 0.053923356 | 0.095928868 | 0.046519691 | BAX | 1 |
| GO:0050820 | positive regulation of coagulation | 1/36 | 29/18866 | 0.053923356 | 0.095928868 | 0.046519691 | F7 | 1 |
| GO:0050901 | leukocyte tethering or rolling | 1/36 | 29/18866 | 0.053923356 | 0.095928868 | 0.046519691 | TNF | 1 |
| GO:0055075 | potassium ion homeostasis | 1/36 | 29/18866 | 0.053923356 | 0.095928868 | 0.046519691 | KCNH2 | 1 |
| GO:0060441 | epithelial tube branching involved in lung morphogenesis | 1/36 | 29/18866 | 0.053923356 | 0.095928868 | 0.046519691 | TNF | 1 |
| GO:0150117 | positive regulation of cell-substrate junction organization | 1/36 | 29/18866 | 0.053923356 | 0.095928868 | 0.046519691 | KDR | 1 |
| GO:0048568 | embryonic organ development | 3/36 | 451/18866 | 0.0541809 | 0.096290149 | 0.046694891 | KDR/TP53/TNF | 3 |
| GO:0009749 | response to glucose | 2/36 | 197/18866 | 0.054204882 | 0.096290149 | 0.046694891 | PRKCE/CASP3 | 2 |
| GO:0006874 | cellular calcium ion homeostasis | 3/36 | 456/18866 | 0.055649599 | 0.097868862 | 0.047460471 | ESR1/PRKCE/BAX | 3 |
| GO:0016052 | carbohydrate catabolic process | 2/36 | 200/18866 | 0.055676066 | 0.097868862 | 0.047460471 | TP53/EIF6 | 2 |
| GO:0000303 | response to superoxide | 1/36 | 30/18866 | 0.055731433 | 0.097868862 | 0.047460471 | TNF | 1 |
| GO:0001516 | prostaglandin biosynthetic process | 1/36 | 30/18866 | 0.055731433 | 0.097868862 | 0.047460471 | IL1B | 1 |
| GO:0003401 | axis elongation | 1/36 | 30/18866 | 0.055731433 | 0.097868862 | 0.047460471 | ESR1 | 1 |
| GO:0019934 | cGMP-mediated signaling | 1/36 | 30/18866 | 0.055731433 | 0.097868862 | 0.047460471 | PDE3A | 1 |
| GO:0033137 | negative regulation of peptidyl-serine phosphorylation | 1/36 | 30/18866 | 0.055731433 | 0.097868862 | 0.047460471 | BAX | 1 |
| GO:0035066 | positive regulation of histone acetylation | 1/36 | 30/18866 | 0.055731433 | 0.097868862 | 0.047460471 | IL1B | 1 |
| GO:0042133 | neurotransmitter metabolic process | 1/36 | 30/18866 | 0.055731433 | 0.097868862 | 0.047460471 | SLC6A4 | 1 |
| GO:0043457 | regulation of cellular respiration | 1/36 | 30/18866 | 0.055731433 | 0.097868862 | 0.047460471 | CCNB1 | 1 |
| GO:0044030 | regulation of DNA methylation | 1/36 | 30/18866 | 0.055731433 | 0.097868862 | 0.047460471 | MYC | 1 |
| GO:0046457 | prostanoid biosynthetic process | 1/36 | 30/18866 | 0.055731433 | 0.097868862 | 0.047460471 | IL1B | 1 |
| GO:0070723 | response to cholesterol | 1/36 | 30/18866 | 0.055731433 | 0.097868862 | 0.047460471 | F7 | 1 |
| GO:0120033 | negative regulation of plasma membrane bounded cell projection assembly | 1/36 | 30/18866 | 0.055731433 | 0.097868862 | 0.047460471 | PRKCD | 1 |
| GO:1902751 | positive regulation of cell cycle G2/M phase transition | 1/36 | 30/18866 | 0.055731433 | 0.097868862 | 0.047460471 | CCNB1 | 1 |
| GO:1904837 | beta-catenin-TCF complex assembly | 1/36 | 30/18866 | 0.055731433 | 0.097868862 | 0.047460471 | MYC | 1 |
| GO:0002221 | pattern recognition receptor signaling pathway | 2/36 | 201/18866 | 0.056169614 | 0.098567835 | 0.04779943 | ESR1/PRKCE | 2 |
| GO:0007179 | transforming growth factor beta receptor signaling pathway | 2/36 | 202/18866 | 0.056664725 | 0.099223895 | 0.04811758 | JUN/TP53 | 2 |
| GO:0009746 | response to hexose | 2/36 | 202/18866 | 0.056664725 | 0.099223895 | 0.04811758 | PRKCE/CASP3 | 2 |
| GO:0034341 | response to interferon-gamma | 2/36 | 202/18866 | 0.056664725 | 0.099223895 | 0.04811758 | TP53/PRKCD | 2 |
| GO:0006473 | protein acetylation | 2/36 | 203/18866 | 0.057161394 | 0.100022254 | 0.048504735 | CHEK1/IL1B | 2 |
| GO:0000305 | response to oxygen radical | 1/36 | 31/18866 | 0.057536151 | 0.100107195 | 0.048545927 | TNF | 1 |
| GO:0045737 | positive regulation of cyclin-dependent protein serine/threonine kinase activity | 1/36 | 31/18866 | 0.057536151 | 0.100107195 | 0.048545927 | CCNB1 | 1 |
| GO:0045948 | positive regulation of translational initiation | 1/36 | 31/18866 | 0.057536151 | 0.100107195 | 0.048545927 | TNF | 1 |
| GO:0045987 | positive regulation of smooth muscle contraction | 1/36 | 31/18866 | 0.057536151 | 0.100107195 | 0.048545927 | CHRM3 | 1 |
| GO:0086011 | membrane repolarization during action potential | 1/36 | 31/18866 | 0.057536151 | 0.100107195 | 0.048545927 | KCNH2 | 1 |
| GO:1900181 | negative regulation of protein localization to nucleus | 1/36 | 31/18866 | 0.057536151 | 0.100107195 | 0.048545927 | PKIA | 1 |
| GO:1900745 | positive regulation of p38MAPK cascade | 1/36 | 31/18866 | 0.057536151 | 0.100107195 | 0.048545927 | IL1B | 1 |
| GO:1902253 | regulation of intrinsic apoptotic signaling pathway by p53 class mediator | 1/36 | 31/18866 | 0.057536151 | 0.100107195 | 0.048545927 | TP53 | 1 |
| GO:0060541 | respiratory system development | 2/36 | 204/18866 | 0.057659612 | 0.100250954 | 0.048615641 | TNF/PGR | 2 |
| GO:0002683 | negative regulation of immune system process | 3/36 | 463/18866 | 0.057738058 | 0.100316301 | 0.048647331 | TNF/MYC/CASP3 | 3 |
| GO:0052547 | regulation of peptidase activity | 3/36 | 466/18866 | 0.058644574 | 0.101727375 | 0.049331616 | TNF/MYC/BAX | 3 |
| GO:0035637 | multicellular organismal signaling | 2/36 | 206/18866 | 0.058660667 | 0.101727375 | 0.049331616 | SCN5A/KCNH2 | 2 |
| GO:0034284 | response to monosaccharide | 2/36 | 207/18866 | 0.05916349 | 0.101727375 | 0.049331616 | PRKCE/CASP3 | 2 |
| GO:0050866 | negative regulation of cell activation | 2/36 | 207/18866 | 0.05916349 | 0.101727375 | 0.049331616 | PRKCD/CASP3 | 2 |
| GO:0055074 | calcium ion homeostasis | 3/36 | 468/18866 | 0.059252719 | 0.101727375 | 0.049331616 | ESR1/PRKCE/BAX | 3 |
| GO:0002082 | regulation of oxidative phosphorylation | 1/36 | 32/18866 | 0.059337516 | 0.101727375 | 0.049331616 | CCNB1 | 1 |
| GO:0002724 | regulation of T cell cytokine production | 1/36 | 32/18866 | 0.059337516 | 0.101727375 | 0.049331616 | IL1B | 1 |
| GO:0034694 | response to prostaglandin | 1/36 | 32/18866 | 0.059337516 | 0.101727375 | 0.049331616 | PRKCE | 1 |
| GO:0035767 | endothelial cell chemotaxis | 1/36 | 32/18866 | 0.059337516 | 0.101727375 | 0.049331616 | KDR | 1 |
| GO:0038128 | ERBB2 signaling pathway | 1/36 | 32/18866 | 0.059337516 | 0.101727375 | 0.049331616 | PRKCA | 1 |
| GO:0044818 | mitotic G2/M transition checkpoint | 1/36 | 32/18866 | 0.059337516 | 0.101727375 | 0.049331616 | CDKN1A | 1 |
| GO:0046949 | fatty-acyl-CoA biosynthetic process | 1/36 | 32/18866 | 0.059337516 | 0.101727375 | 0.049331616 | FASN | 1 |
| GO:0050869 | negative regulation of B cell activation | 1/36 | 32/18866 | 0.059337516 | 0.101727375 | 0.049331616 | CASP3 | 1 |
| GO:0051385 | response to mineralocorticoid | 1/36 | 32/18866 | 0.059337516 | 0.101727375 | 0.049331616 | CDKN1A | 1 |
| GO:0071353 | cellular response to interleukin-4 | 1/36 | 32/18866 | 0.059337516 | 0.101727375 | 0.049331616 | FASN | 1 |
| GO:0086019 | cell-cell signaling involved in cardiac conduction | 1/36 | 32/18866 | 0.059337516 | 0.101727375 | 0.049331616 | SCN5A | 1 |
| GO:0097421 | liver regeneration | 1/36 | 32/18866 | 0.059337516 | 0.101727375 | 0.049331616 | PCNA | 1 |
| GO:0098810 | neurotransmitter reuptake | 1/36 | 32/18866 | 0.059337516 | 0.101727375 | 0.049331616 | SLC6A4 | 1 |
| GO:1901976 | regulation of cell cycle checkpoint | 1/36 | 32/18866 | 0.059337516 | 0.101727375 | 0.049331616 | CCNB1 | 1 |
| GO:0061013 | regulation of mRNA catabolic process | 2/36 | 208/18866 | 0.059667832 | 0.102222281 | 0.049571615 | PRKCD/PRKCA | 2 |
| GO:0070838 | divalent metal ion transport | 3/36 | 471/18866 | 0.060170622 | 0.102945229 | 0.049922201 | PRKCE/BAX/OPRM1 | 3 |
| GO:0035303 | regulation of dephosphorylation | 2/36 | 209/18866 | 0.060173688 | 0.102945229 | 0.049922201 | TNF/PRKCD | 2 |
| GO:0034764 | positive regulation of transmembrane transport | 2/36 | 210/18866 | 0.060681049 | 0.103458145 | 0.050170935 | BAX/KCNH2 | 2 |
| GO:0051346 | negative regulation of hydrolase activity | 3/36 | 473/18866 | 0.060786335 | 0.103458145 | 0.050170935 | TP53/TNF/MAP2 | 3 |
| GO:0002861 | regulation of inflammatory response to antigenic stimulus | 1/36 | 33/18866 | 0.061135533 | 0.103458145 | 0.050170935 | TNF | 1 |
| GO:0009303 | rRNA transcription | 1/36 | 33/18866 | 0.061135533 | 0.103458145 | 0.050170935 | TP53 | 1 |
| GO:0009648 | photoperiodism | 1/36 | 33/18866 | 0.061135533 | 0.103458145 | 0.050170935 | TP53 | 1 |
| GO:0010039 | response to iron ion | 1/36 | 33/18866 | 0.061135533 | 0.103458145 | 0.050170935 | CCNB1 | 1 |
| GO:0010464 | regulation of mesenchymal cell proliferation | 1/36 | 33/18866 | 0.061135533 | 0.103458145 | 0.050170935 | MYC | 1 |
| GO:0030149 | sphingolipid catabolic process | 1/36 | 33/18866 | 0.061135533 | 0.103458145 | 0.050170935 | PRKCD | 1 |
| GO:0040020 | regulation of meiotic nuclear division | 1/36 | 33/18866 | 0.061135533 | 0.103458145 | 0.050170935 | PDE3A | 1 |
| GO:0042755 | eating behavior | 1/36 | 33/18866 | 0.061135533 | 0.103458145 | 0.050170935 | OPRM1 | 1 |
| GO:0048011 | neurotrophin TRK receptor signaling pathway | 1/36 | 33/18866 | 0.061135533 | 0.103458145 | 0.050170935 | CASP3 | 1 |
| GO:0050685 | positive regulation of mRNA processing | 1/36 | 33/18866 | 0.061135533 | 0.103458145 | 0.050170935 | CCNB1 | 1 |
| GO:0051984 | positive regulation of chromosome segregation | 1/36 | 33/18866 | 0.061135533 | 0.103458145 | 0.050170935 | CCNB1 | 1 |
| GO:1901380 | negative regulation of potassium ion transmembrane transport | 1/36 | 33/18866 | 0.061135533 | 0.103458145 | 0.050170935 | KCNH2 | 1 |
| GO:2000758 | positive regulation of peptidyl-lysine acetylation | 1/36 | 33/18866 | 0.061135533 | 0.103458145 | 0.050170935 | IL1B | 1 |
| GO:0002703 | regulation of leukocyte mediated immunity | 2/36 | 211/18866 | 0.061189909 | 0.103458145 | 0.050170935 | TNF/IL1B | 2 |
| GO:0070555 | response to interleukin-1 | 2/36 | 211/18866 | 0.061189909 | 0.103458145 | 0.050170935 | PRKCA/IL1B | 2 |
| GO:0002685 | regulation of leukocyte migration | 2/36 | 212/18866 | 0.06170026 | 0.104177537 | 0.050519796 | F7/TNF | 2 |
| GO:1901215 | negative regulation of neuron death | 2/36 | 212/18866 | 0.06170026 | 0.104177537 | 0.050519796 | JUN/BAX | 2 |
| GO:0007254 | JNK cascade | 2/36 | 213/18866 | 0.062212096 | 0.104387609 | 0.050621668 | TNF/IL1B | 2 |
| GO:0072511 | divalent inorganic cation transport | 3/36 | 478/18866 | 0.062338788 | 0.104387609 | 0.050621668 | PRKCE/BAX/OPRM1 | 3 |
| GO:0002756 | MyD88-independent toll-like receptor signaling pathway | 1/36 | 34/18866 | 0.062930208 | 0.104387609 | 0.050621668 | PRKCE | 1 |
| GO:0007094 | mitotic spindle assembly checkpoint | 1/36 | 34/18866 | 0.062930208 | 0.104387609 | 0.050621668 | CCNB1 | 1 |
| GO:0008156 | negative regulation of DNA replication | 1/36 | 34/18866 | 0.062930208 | 0.104387609 | 0.050621668 | TP53 | 1 |
| GO:0008608 | attachment of spindle microtubules to kinetochore | 1/36 | 34/18866 | 0.062930208 | 0.104387609 | 0.050621668 | CCNB1 | 1 |
| GO:0009649 | entrainment of circadian clock | 1/36 | 34/18866 | 0.062930208 | 0.104387609 | 0.050621668 | TP53 | 1 |
| GO:0010762 | regulation of fibroblast migration | 1/36 | 34/18866 | 0.062930208 | 0.104387609 | 0.050621668 | PRKCE | 1 |
| GO:0010765 | positive regulation of sodium ion transport | 1/36 | 34/18866 | 0.062930208 | 0.104387609 | 0.050621668 | SCN5A | 1 |
| GO:0016242 | negative regulation of macroautophagy | 1/36 | 34/18866 | 0.062930208 | 0.104387609 | 0.050621668 | TP53 | 1 |
| GO:0019228 | neuronal action potential | 1/36 | 34/18866 | 0.062930208 | 0.104387609 | 0.050621668 | SCN5A | 1 |
| GO:0030851 | granulocyte differentiation | 1/36 | 34/18866 | 0.062930208 | 0.104387609 | 0.050621668 | FASN | 1 |
| GO:0031577 | spindle checkpoint | 1/36 | 34/18866 | 0.062930208 | 0.104387609 | 0.050621668 | CCNB1 | 1 |
| GO:0033198 | response to ATP | 1/36 | 34/18866 | 0.062930208 | 0.104387609 | 0.050621668 | IL1B | 1 |
| GO:0043516 | regulation of DNA damage response, signal transduction by p53 class mediator | 1/36 | 34/18866 | 0.062930208 | 0.104387609 | 0.050621668 | TP53 | 1 |
| GO:0045907 | positive regulation of vasoconstriction | 1/36 | 34/18866 | 0.062930208 | 0.104387609 | 0.050621668 | CHRM3 | 1 |
| GO:0046685 | response to arsenic-containing substance | 1/36 | 34/18866 | 0.062930208 | 0.104387609 | 0.050621668 | CDKN1A | 1 |
| GO:0048566 | embryonic digestive tract development | 1/36 | 34/18866 | 0.062930208 | 0.104387609 | 0.050621668 | TNF | 1 |
| GO:0048854 | brain morphogenesis | 1/36 | 34/18866 | 0.062930208 | 0.104387609 | 0.050621668 | SLC6A4 | 1 |
| GO:0050715 | positive regulation of cytokine secretion | 1/36 | 34/18866 | 0.062930208 | 0.104387609 | 0.050621668 | TNF | 1 |
| GO:0070232 | regulation of T cell apoptotic process | 1/36 | 34/18866 | 0.062930208 | 0.104387609 | 0.050621668 | TP53 | 1 |
| GO:0071173 | spindle assembly checkpoint | 1/36 | 34/18866 | 0.062930208 | 0.104387609 | 0.050621668 | CCNB1 | 1 |
| GO:0071174 | mitotic spindle checkpoint | 1/36 | 34/18866 | 0.062930208 | 0.104387609 | 0.050621668 | CCNB1 | 1 |
| GO:1902692 | regulation of neuroblast proliferation | 1/36 | 34/18866 | 0.062930208 | 0.104387609 | 0.050621668 | TP53 | 1 |
| GO:2000036 | regulation of stem cell population maintenance | 1/36 | 34/18866 | 0.062930208 | 0.104387609 | 0.050621668 | MYC | 1 |
| GO:2000352 | negative regulation of endothelial cell apoptotic process | 1/36 | 34/18866 | 0.062930208 | 0.104387609 | 0.050621668 | KDR | 1 |
| GO:0002429 | immune response-activating cell surface receptor signaling pathway | 3/36 | 481/18866 | 0.063279251 | 0.10482494 | 0.050833747 | PRKCE/PRKCD/BAX | 3 |
| GO:0002757 | immune response-activating signal transduction | 3/36 | 481/18866 | 0.063279251 | 0.10482494 | 0.050833747 | PRKCE/PRKCD/BAX | 3 |
| GO:0051651 | maintenance of location in cell | 2/36 | 216/18866 | 0.063756434 | 0.105544199 | 0.051182544 | PRKCE/BAX | 2 |
| GO:0010810 | regulation of cell-substrate adhesion | 2/36 | 217/18866 | 0.064274135 | 0.10613988 | 0.051471414 | KDR/PRKCE | 2 |
| GO:0050792 | regulation of viral process | 2/36 | 217/18866 | 0.064274135 | 0.10613988 | 0.051471414 | JUN/TNF | 2 |
| GO:0050769 | positive regulation of neurogenesis | 3/36 | 485/18866 | 0.064543636 | 0.10613988 | 0.051471414 | TNF/IL1B/OPRM1 | 3 |
| GO:0000083 | regulation of transcription involved in G1/S transition of mitotic cell cycle | 1/36 | 35/18866 | 0.064721548 | 0.10613988 | 0.051471414 | PCNA | 1 |
| GO:0006739 | NADP metabolic process | 1/36 | 35/18866 | 0.064721548 | 0.10613988 | 0.051471414 | TP53 | 1 |
| GO:0007435 | salivary gland morphogenesis | 1/36 | 35/18866 | 0.064721548 | 0.10613988 | 0.051471414 | TNF | 1 |
| GO:0010259 | multicellular organism aging | 1/36 | 35/18866 | 0.064721548 | 0.10613988 | 0.051471414 | TP53 | 1 |
| GO:0030947 | regulation of vascular endothelial growth factor receptor signaling pathway | 1/36 | 35/18866 | 0.064721548 | 0.10613988 | 0.051471414 | IL1B | 1 |
| GO:0031297 | replication fork processing | 1/36 | 35/18866 | 0.064721548 | 0.10613988 | 0.051471414 | PCNA | 1 |
| GO:0032228 | regulation of synaptic transmission, GABAergic | 1/36 | 35/18866 | 0.064721548 | 0.10613988 | 0.051471414 | PRKCE | 1 |
| GO:0036314 | response to sterol | 1/36 | 35/18866 | 0.064721548 | 0.10613988 | 0.051471414 | F7 | 1 |
| GO:0070670 | response to interleukin-4 | 1/36 | 35/18866 | 0.064721548 | 0.10613988 | 0.051471414 | FASN | 1 |
| GO:0070884 | regulation of calcineurin-NFAT signaling cascade | 1/36 | 35/18866 | 0.064721548 | 0.10613988 | 0.051471414 | TNF | 1 |
| GO:0110111 | negative regulation of animal organ morphogenesis | 1/36 | 35/18866 | 0.064721548 | 0.10613988 | 0.051471414 | TNF | 1 |
| GO:0006338 | chromatin remodeling | 2/36 | 218/18866 | 0.064793284 | 0.10618659 | 0.051494065 | ESR1/MYC | 2 |
| GO:0008154 | actin polymerization or depolymerization | 2/36 | 221/18866 | 0.066359351 | 0.107990057 | 0.052368637 | PRKCE/PRKCD | 2 |
| GO:0000460 | maturation of 5.8S rRNA | 1/36 | 36/18866 | 0.066509558 | 0.107990057 | 0.052368637 | EIF6 | 1 |
| GO:0001569 | branching involved in blood vessel morphogenesis | 1/36 | 36/18866 | 0.066509558 | 0.107990057 | 0.052368637 | KDR | 1 |
| GO:0002701 | negative regulation of production of molecular mediator of immune response | 1/36 | 36/18866 | 0.066509558 | 0.107990057 | 0.052368637 | TNF | 1 |
| GO:0003382 | epithelial cell morphogenesis | 1/36 | 36/18866 | 0.066509558 | 0.107990057 | 0.052368637 | AR | 1 |
| GO:0006007 | glucose catabolic process | 1/36 | 36/18866 | 0.066509558 | 0.107990057 | 0.052368637 | TP53 | 1 |
| GO:0010092 | specification of animal organ identity | 1/36 | 36/18866 | 0.066509558 | 0.107990057 | 0.052368637 | AR | 1 |
| GO:0031128 | developmental induction | 1/36 | 36/18866 | 0.066509558 | 0.107990057 | 0.052368637 | AR | 1 |
| GO:0042554 | superoxide anion generation | 1/36 | 36/18866 | 0.066509558 | 0.107990057 | 0.052368637 | PRKCD | 1 |
| GO:0045841 | negative regulation of mitotic metaphase/anaphase transition | 1/36 | 36/18866 | 0.066509558 | 0.107990057 | 0.052368637 | CCNB1 | 1 |
| GO:0051973 | positive regulation of telomerase activity | 1/36 | 36/18866 | 0.066509558 | 0.107990057 | 0.052368637 | MYC | 1 |
| GO:0106056 | regulation of calcineurin-mediated signaling | 1/36 | 36/18866 | 0.066509558 | 0.107990057 | 0.052368637 | TNF | 1 |
| GO:1905332 | positive regulation of morphogenesis of an epithelium | 1/36 | 36/18866 | 0.066509558 | 0.107990057 | 0.052368637 | AR | 1 |
| GO:2000310 | regulation of NMDA receptor activity | 1/36 | 36/18866 | 0.066509558 | 0.107990057 | 0.052368637 | OPRM1 | 1 |
| GO:0016311 | dephosphorylation | 3/36 | 492/18866 | 0.066784824 | 0.108293754 | 0.052515912 | TNF/PRKCD/PON1 | 3 |
| GO:0072503 | cellular divalent inorganic cation homeostasis | 3/36 | 492/18866 | 0.066784824 | 0.108293754 | 0.052515912 | ESR1/PRKCE/BAX | 3 |
| GO:0002699 | positive regulation of immune effector process | 2/36 | 223/18866 | 0.06741051 | 0.109164118 | 0.052937986 | TNF/IL1B | 2 |
| GO:0016485 | protein processing | 2/36 | 223/18866 | 0.06741051 | 0.109164118 | 0.052937986 | F7/CASP3 | 2 |
| GO:0006296 | nucleotide-excision repair, DNA incision, 5'-to lesion | 1/36 | 37/18866 | 0.068294245 | 0.110086916 | 0.053385487 | PCNA | 1 |
| GO:0030212 | hyaluronan metabolic process | 1/36 | 37/18866 | 0.068294245 | 0.110086916 | 0.053385487 | IL1B | 1 |
| GO:0043243 | positive regulation of protein-containing complex disassembly | 1/36 | 37/18866 | 0.068294245 | 0.110086916 | 0.053385487 | TNF | 1 |
| GO:0090050 | positive regulation of cell migration involved in sprouting angiogenesis | 1/36 | 37/18866 | 0.068294245 | 0.110086916 | 0.053385487 | KDR | 1 |
| GO:0097242 | amyloid-beta clearance | 1/36 | 37/18866 | 0.068294245 | 0.110086916 | 0.053385487 | TNF | 1 |
| GO:1902100 | negative regulation of metaphase/anaphase transition of cell cycle | 1/36 | 37/18866 | 0.068294245 | 0.110086916 | 0.053385487 | CCNB1 | 1 |
| GO:1904994 | regulation of leukocyte adhesion to vascular endothelial cell | 1/36 | 37/18866 | 0.068294245 | 0.110086916 | 0.053385487 | TNF | 1 |
| GO:0045665 | negative regulation of neuron differentiation | 2/36 | 225/18866 | 0.068467295 | 0.110221121 | 0.053450568 | SLC6A4/MAP2 | 2 |
| GO:0045732 | positive regulation of protein catabolic process | 2/36 | 225/18866 | 0.068467295 | 0.110221121 | 0.053450568 | TNF/IL1B | 2 |
| GO:0032869 | cellular response to insulin stimulus | 2/36 | 226/18866 | 0.06899778 | 0.111002327 | 0.053829406 | PRKCD/IL1B | 2 |
| GO:0016064 | immunoglobulin mediated immune response | 2/36 | 227/18866 | 0.069529651 | 0.111711451 | 0.054173288 | TNF/PRKCD | 2 |
| GO:0003156 | regulation of animal organ formation | 1/36 | 38/18866 | 0.070075615 | 0.111711451 | 0.054173288 | AR | 1 |
| GO:0006084 | acetyl-CoA metabolic process | 1/36 | 38/18866 | 0.070075615 | 0.111711451 | 0.054173288 | FASN | 1 |
| GO:0006298 | mismatch repair | 1/36 | 38/18866 | 0.070075615 | 0.111711451 | 0.054173288 | PCNA | 1 |
| GO:0010661 | positive regulation of muscle cell apoptotic process | 1/36 | 38/18866 | 0.070075615 | 0.111711451 | 0.054173288 | TP53 | 1 |
| GO:0032094 | response to food | 1/36 | 38/18866 | 0.070075615 | 0.111711451 | 0.054173288 | OPRM1 | 1 |
| GO:0042769 | DNA damage response, detection of DNA damage | 1/36 | 38/18866 | 0.070075615 | 0.111711451 | 0.054173288 | PCNA | 1 |
| GO:0045923 | positive regulation of fatty acid metabolic process | 1/36 | 38/18866 | 0.070075615 | 0.111711451 | 0.054173288 | IL1B | 1 |
| GO:0046466 | membrane lipid catabolic process | 1/36 | 38/18866 | 0.070075615 | 0.111711451 | 0.054173288 | PRKCD | 1 |
| GO:0048009 | insulin-like growth factor receptor signaling pathway | 1/36 | 38/18866 | 0.070075615 | 0.111711451 | 0.054173288 | AR | 1 |
| GO:0051930 | regulation of sensory perception of pain | 1/36 | 38/18866 | 0.070075615 | 0.111711451 | 0.054173288 | OPRM1 | 1 |
| GO:0060045 | positive regulation of cardiac muscle cell proliferation | 1/36 | 38/18866 | 0.070075615 | 0.111711451 | 0.054173288 | CCNB1 | 1 |
| GO:0071276 | cellular response to cadmium ion | 1/36 | 38/18866 | 0.070075615 | 0.111711451 | 0.054173288 | JUN | 1 |
| GO:1905898 | positive regulation of response to endoplasmic reticulum stress | 1/36 | 38/18866 | 0.070075615 | 0.111711451 | 0.054173288 | BAX | 1 |
| GO:0050920 | regulation of chemotaxis | 2/36 | 229/18866 | 0.070597523 | 0.11247042 | 0.054541342 | KDR/F7 | 2 |
| GO:0010001 | glial cell differentiation | 2/36 | 230/18866 | 0.07113351 | 0.113104123 | 0.05484865 | TNF/IL1B | 2 |
| GO:0019724 | B cell mediated immunity | 2/36 | 230/18866 | 0.07113351 | 0.113104123 | 0.05484865 | TNF/PRKCD | 2 |
| GO:0098657 | import into cell | 2/36 | 230/18866 | 0.07113351 | 0.113104123 | 0.05484865 | SLC6A4/KCNH2 | 2 |
| GO:0001649 | osteoblast differentiation | 2/36 | 231/18866 | 0.071670855 | 0.113368102 | 0.054976664 | TNF/FASN | 2 |
| GO:0043903 | regulation of symbiotic process | 2/36 | 231/18866 | 0.071670855 | 0.113368102 | 0.054976664 | JUN/TNF | 2 |
| GO:0007431 | salivary gland development | 1/36 | 39/18866 | 0.071853673 | 0.113368102 | 0.054976664 | TNF | 1 |
| GO:0010453 | regulation of cell fate commitment | 1/36 | 39/18866 | 0.071853673 | 0.113368102 | 0.054976664 | AR | 1 |
| GO:0030866 | cortical actin cytoskeleton organization | 1/36 | 39/18866 | 0.071853673 | 0.113368102 | 0.054976664 | TNF | 1 |
| GO:0031111 | negative regulation of microtubule polymerization or depolymerization | 1/36 | 39/18866 | 0.071853673 | 0.113368102 | 0.054976664 | MAP2 | 1 |
| GO:0033683 | nucleotide-excision repair, DNA incision | 1/36 | 39/18866 | 0.071853673 | 0.113368102 | 0.054976664 | PCNA | 1 |
| GO:0038179 | neurotrophin signaling pathway | 1/36 | 39/18866 | 0.071853673 | 0.113368102 | 0.054976664 | CASP3 | 1 |
| GO:0051931 | regulation of sensory perception | 1/36 | 39/18866 | 0.071853673 | 0.113368102 | 0.054976664 | OPRM1 | 1 |
| GO:0060416 | response to growth hormone | 1/36 | 39/18866 | 0.071853673 | 0.113368102 | 0.054976664 | F7 | 1 |
| GO:1904706 | negative regulation of vascular associated smooth muscle cell proliferation | 1/36 | 39/18866 | 0.071853673 | 0.113368102 | 0.054976664 | CDKN1A | 1 |
| GO:2000816 | negative regulation of mitotic sister chromatid separation | 1/36 | 39/18866 | 0.071853673 | 0.113368102 | 0.054976664 | CCNB1 | 1 |
| GO:0030595 | leukocyte chemotaxis | 2/36 | 232/18866 | 0.072209552 | 0.113856423 | 0.055213469 | F7/IL1B | 2 |
| GO:0002714 | positive regulation of B cell mediated immunity | 1/36 | 40/18866 | 0.073628426 | 0.114912769 | 0.055725733 | TNF | 1 |
| GO:0002891 | positive regulation of immunoglobulin mediated immune response | 1/36 | 40/18866 | 0.073628426 | 0.114912769 | 0.055725733 | TNF | 1 |
| GO:0006284 | base-excision repair | 1/36 | 40/18866 | 0.073628426 | 0.114912769 | 0.055725733 | PCNA | 1 |
| GO:0010613 | positive regulation of cardiac muscle hypertrophy | 1/36 | 40/18866 | 0.073628426 | 0.114912769 | 0.055725733 | PRKCA | 1 |
| GO:0032467 | positive regulation of cytokinesis | 1/36 | 40/18866 | 0.073628426 | 0.114912769 | 0.055725733 | PRKCE | 1 |
| GO:0032892 | positive regulation of organic acid transport | 1/36 | 40/18866 | 0.073628426 | 0.114912769 | 0.055725733 | IL1B | 1 |
| GO:0034142 | toll-like receptor 4 signaling pathway | 1/36 | 40/18866 | 0.073628426 | 0.114912769 | 0.055725733 | PRKCE | 1 |
| GO:0042307 | positive regulation of protein import into nucleus | 1/36 | 40/18866 | 0.073628426 | 0.114912769 | 0.055725733 | PRKCD | 1 |
| GO:0043902 | positive regulation of multi-organism process | 1/36 | 40/18866 | 0.073628426 | 0.114912769 | 0.055725733 | PDE3A | 1 |
| GO:0050691 | regulation of defense response to virus by host | 1/36 | 40/18866 | 0.073628426 | 0.114912769 | 0.055725733 | IL1B | 1 |
| GO:0051281 | positive regulation of release of sequestered calcium ion into cytosol | 1/36 | 40/18866 | 0.073628426 | 0.114912769 | 0.055725733 | BAX | 1 |
| GO:0090184 | positive regulation of kidney development | 1/36 | 40/18866 | 0.073628426 | 0.114912769 | 0.055725733 | MYC | 1 |
| GO:0140353 | lipid export from cell | 1/36 | 40/18866 | 0.073628426 | 0.114912769 | 0.055725733 | IL1B | 1 |
| GO:1901381 | positive regulation of potassium ion transmembrane transport | 1/36 | 40/18866 | 0.073628426 | 0.114912769 | 0.055725733 | KCNH2 | 1 |
| GO:1902742 | apoptotic process involved in development | 1/36 | 40/18866 | 0.073628426 | 0.114912769 | 0.055725733 | BAX | 1 |
| GO:1905819 | negative regulation of chromosome separation | 1/36 | 40/18866 | 0.073628426 | 0.114912769 | 0.055725733 | CCNB1 | 1 |
| GO:1903039 | positive regulation of leukocyte cell-cell adhesion | 2/36 | 235/18866 | 0.073833685 | 0.115159909 | 0.055845581 | TNF/IL1B | 2 |
| GO:0060560 | developmental growth involved in morphogenesis | 2/36 | 236/18866 | 0.074377721 | 0.115934797 | 0.056221355 | ESR1/MAP2 | 2 |
| GO:0046777 | protein autophosphorylation | 2/36 | 237/18866 | 0.074923075 | 0.11641931 | 0.056456314 | JUN/KDR | 2 |
| GO:0006734 | NADH metabolic process | 1/36 | 41/18866 | 0.075399879 | 0.11641931 | 0.056456314 | TP53 | 1 |
| GO:0007212 | dopamine receptor signaling pathway | 1/36 | 41/18866 | 0.075399879 | 0.11641931 | 0.056456314 | OPRM1 | 1 |
| GO:0007618 | mating | 1/36 | 41/18866 | 0.075399879 | 0.11641931 | 0.056456314 | SLC6A4 | 1 |
| GO:0014742 | positive regulation of muscle hypertrophy | 1/36 | 41/18866 | 0.075399879 | 0.11641931 | 0.056456314 | PRKCA | 1 |
| GO:0030890 | positive regulation of B cell proliferation | 1/36 | 41/18866 | 0.075399879 | 0.11641931 | 0.056456314 | CDKN1A | 1 |
| GO:0033574 | response to testosterone | 1/36 | 41/18866 | 0.075399879 | 0.11641931 | 0.056456314 | AR | 1 |
| GO:0035337 | fatty-acyl-CoA metabolic process | 1/36 | 41/18866 | 0.075399879 | 0.11641931 | 0.056456314 | FASN | 1 |
| GO:0038083 | peptidyl-tyrosine autophosphorylation | 1/36 | 41/18866 | 0.075399879 | 0.11641931 | 0.056456314 | KDR | 1 |
| GO:0043267 | negative regulation of potassium ion transport | 1/36 | 41/18866 | 0.075399879 | 0.11641931 | 0.056456314 | KCNH2 | 1 |
| GO:0048286 | lung alveolus development | 1/36 | 41/18866 | 0.075399879 | 0.11641931 | 0.056456314 | PGR | 1 |
| GO:0050434 | positive regulation of viral transcription | 1/36 | 41/18866 | 0.075399879 | 0.11641931 | 0.056456314 | JUN | 1 |
| GO:0070317 | negative regulation of G0 to G1 transition | 1/36 | 41/18866 | 0.075399879 | 0.11641931 | 0.056456314 | CHEK1 | 1 |
| GO:0071548 | response to dexamethasone | 1/36 | 41/18866 | 0.075399879 | 0.11641931 | 0.056456314 | PCNA | 1 |
| GO:2000008 | regulation of protein localization to cell surface | 1/36 | 41/18866 | 0.075399879 | 0.11641931 | 0.056456314 | TNF | 1 |
| GO:0006323 | DNA packaging | 2/36 | 240/18866 | 0.076566978 | 0.118072821 | 0.057258167 | TP53/CCNB1 | 2 |
| GO:0032886 | regulation of microtubule-based process | 2/36 | 240/18866 | 0.076566978 | 0.118072821 | 0.057258167 | CHEK1/MAP2 | 2 |
| GO:0000266 | mitochondrial fission | 1/36 | 42/18866 | 0.077168038 | 0.118404709 | 0.057419113 | KDR | 1 |
| GO:0001504 | neurotransmitter uptake | 1/36 | 42/18866 | 0.077168038 | 0.118404709 | 0.057419113 | SLC6A4 | 1 |
| GO:0019985 | translesion synthesis | 1/36 | 42/18866 | 0.077168038 | 0.118404709 | 0.057419113 | PCNA | 1 |
| GO:0033048 | negative regulation of mitotic sister chromatid segregation | 1/36 | 42/18866 | 0.077168038 | 0.118404709 | 0.057419113 | CCNB1 | 1 |
| GO:0046006 | regulation of activated T cell proliferation | 1/36 | 42/18866 | 0.077168038 | 0.118404709 | 0.057419113 | CASP3 | 1 |
| GO:0046621 | negative regulation of organ growth | 1/36 | 42/18866 | 0.077168038 | 0.118404709 | 0.057419113 | SLC6A4 | 1 |
| GO:0071470 | cellular response to osmotic stress | 1/36 | 42/18866 | 0.077168038 | 0.118404709 | 0.057419113 | CASP3 | 1 |
| GO:1904591 | positive regulation of protein import | 1/36 | 42/18866 | 0.077168038 | 0.118404709 | 0.057419113 | PRKCD | 1 |
| GO:0065004 | protein-DNA complex assembly | 2/36 | 243/18866 | 0.078222503 | 0.119947686 | 0.058167363 | ESR1/TP53 | 2 |
| GO:0002369 | T cell cytokine production | 1/36 | 43/18866 | 0.07893291 | 0.120211101 | 0.058295104 | IL1B | 1 |
| GO:0008631 | intrinsic apoptotic signaling pathway in response to oxidative stress | 1/36 | 43/18866 | 0.07893291 | 0.120211101 | 0.058295104 | PRKCD | 1 |
| GO:0030517 | negative regulation of axon extension | 1/36 | 43/18866 | 0.07893291 | 0.120211101 | 0.058295104 | MAP2 | 1 |
| GO:0030521 | androgen receptor signaling pathway | 1/36 | 43/18866 | 0.07893291 | 0.120211101 | 0.058295104 | AR | 1 |
| GO:0040019 | positive regulation of embryonic development | 1/36 | 43/18866 | 0.07893291 | 0.120211101 | 0.058295104 | AR | 1 |
| GO:0045124 | regulation of bone resorption | 1/36 | 43/18866 | 0.07893291 | 0.120211101 | 0.058295104 | PRKCA | 1 |
| GO:0050850 | positive regulation of calcium-mediated signaling | 1/36 | 43/18866 | 0.07893291 | 0.120211101 | 0.058295104 | TNF | 1 |
| GO:0086004 | regulation of cardiac muscle cell contraction | 1/36 | 43/18866 | 0.07893291 | 0.120211101 | 0.058295104 | SCN5A | 1 |
| GO:0090317 | negative regulation of intracellular protein transport | 1/36 | 43/18866 | 0.07893291 | 0.120211101 | 0.058295104 | PKIA | 1 |
| GO:1901031 | regulation of response to reactive oxygen species | 1/36 | 43/18866 | 0.07893291 | 0.120211101 | 0.058295104 | TNF | 1 |
| GO:1903146 | regulation of autophagy of mitochondrion | 1/36 | 43/18866 | 0.07893291 | 0.120211101 | 0.058295104 | TP53 | 1 |
| GO:0010463 | mesenchymal cell proliferation | 1/36 | 44/18866 | 0.080694501 | 0.121985837 | 0.059155743 | MYC | 1 |
| GO:0033046 | negative regulation of sister chromatid segregation | 1/36 | 44/18866 | 0.080694501 | 0.121985837 | 0.059155743 | CCNB1 | 1 |
| GO:0033173 | calcineurin-NFAT signaling cascade | 1/36 | 44/18866 | 0.080694501 | 0.121985837 | 0.059155743 | TNF | 1 |
| GO:0035307 | positive regulation of protein dephosphorylation | 1/36 | 44/18866 | 0.080694501 | 0.121985837 | 0.059155743 | PRKCD | 1 |
| GO:0035329 | hippo signaling | 1/36 | 44/18866 | 0.080694501 | 0.121985837 | 0.059155743 | CASP3 | 1 |
| GO:0042088 | T-helper 1 type immune response | 1/36 | 44/18866 | 0.080694501 | 0.121985837 | 0.059155743 | IL1B | 1 |
| GO:0044060 | regulation of endocrine process | 1/36 | 44/18866 | 0.080694501 | 0.121985837 | 0.059155743 | IL1B | 1 |
| GO:0045005 | DNA-dependent DNA replication maintenance of fidelity | 1/36 | 44/18866 | 0.080694501 | 0.121985837 | 0.059155743 | PCNA | 1 |
| GO:0045332 | phospholipid translocation | 1/36 | 44/18866 | 0.080694501 | 0.121985837 | 0.059155743 | PRKCD | 1 |
| GO:0045684 | positive regulation of epidermis development | 1/36 | 44/18866 | 0.080694501 | 0.121985837 | 0.059155743 | TNF | 1 |
| GO:0048489 | synaptic vesicle transport | 1/36 | 44/18866 | 0.080694501 | 0.121985837 | 0.059155743 | MAP2 | 1 |
| GO:1901985 | positive regulation of protein acetylation | 1/36 | 44/18866 | 0.080694501 | 0.121985837 | 0.059155743 | IL1B | 1 |
| GO:0043543 | protein acylation | 2/36 | 248/18866 | 0.081007058 | 0.12238297 | 0.059348328 | CHEK1/IL1B | 2 |
| GO:0001974 | blood vessel remodeling | 1/36 | 45/18866 | 0.082452815 | 0.124108928 | 0.060185314 | BAX | 1 |
| GO:0034198 | cellular response to amino acid starvation | 1/36 | 45/18866 | 0.082452815 | 0.124108928 | 0.060185314 | CDKN1A | 1 |
| GO:0043114 | regulation of vascular permeability | 1/36 | 45/18866 | 0.082452815 | 0.124108928 | 0.060185314 | PDE3A | 1 |
| GO:0051985 | negative regulation of chromosome segregation | 1/36 | 45/18866 | 0.082452815 | 0.124108928 | 0.060185314 | CCNB1 | 1 |
| GO:0070316 | regulation of G0 to G1 transition | 1/36 | 45/18866 | 0.082452815 | 0.124108928 | 0.060185314 | CHEK1 | 1 |
| GO:0090311 | regulation of protein deacetylation | 1/36 | 45/18866 | 0.082452815 | 0.124108928 | 0.060185314 | TP53 | 1 |
| GO:0002833 | positive regulation of response to biotic stimulus | 2/36 | 251/18866 | 0.082692711 | 0.124393754 | 0.060323437 | PRKCD/PRKCA | 2 |
| GO:0030217 | T cell differentiation | 2/36 | 253/18866 | 0.08382259 | 0.125671913 | 0.060943267 | TP53/IL1B | 2 |
| GO:0051924 | regulation of calcium ion transport | 2/36 | 253/18866 | 0.08382259 | 0.125671913 | 0.060943267 | PRKCE/BAX | 2 |
| GO:0010761 | fibroblast migration | 1/36 | 46/18866 | 0.08420786 | 0.125671913 | 0.060943267 | PRKCE | 1 |
| GO:0010863 | positive regulation of phospholipase C activity | 1/36 | 46/18866 | 0.08420786 | 0.125671913 | 0.060943267 | ESR1 | 1 |
| GO:0010874 | regulation of cholesterol efflux | 1/36 | 46/18866 | 0.08420786 | 0.125671913 | 0.060943267 | PON1 | 1 |
| GO:0032309 | icosanoid secretion | 1/36 | 46/18866 | 0.08420786 | 0.125671913 | 0.060943267 | IL1B | 1 |
| GO:0032570 | response to progesterone | 1/36 | 46/18866 | 0.08420786 | 0.125671913 | 0.060943267 | NCOA2 | 1 |
| GO:0044275 | cellular carbohydrate catabolic process | 1/36 | 46/18866 | 0.08420786 | 0.125671913 | 0.060943267 | TP53 | 1 |
| GO:0045023 | G0 to G1 transition | 1/36 | 46/18866 | 0.08420786 | 0.125671913 | 0.060943267 | CHEK1 | 1 |
| GO:0048066 | developmental pigmentation | 1/36 | 46/18866 | 0.08420786 | 0.125671913 | 0.060943267 | BAX | 1 |
| GO:0050798 | activated T cell proliferation | 1/36 | 46/18866 | 0.08420786 | 0.125671913 | 0.060943267 | CASP3 | 1 |
| GO:0051489 | regulation of filopodium assembly | 1/36 | 46/18866 | 0.08420786 | 0.125671913 | 0.060943267 | PRKCD | 1 |
| GO:1900744 | regulation of p38MAPK cascade | 1/36 | 46/18866 | 0.08420786 | 0.125671913 | 0.060943267 | IL1B | 1 |
| GO:0008016 | regulation of heart contraction | 2/36 | 256/18866 | 0.085526446 | 0.127357223 | 0.06176054 | SCN5A/KCNH2 | 2 |
| GO:0048872 | homeostasis of number of cells | 2/36 | 256/18866 | 0.085526446 | 0.127357223 | 0.06176054 | CASP3/BAX | 2 |
| GO:0006383 | transcription by RNA polymerase III | 1/36 | 47/18866 | 0.085959641 | 0.127357223 | 0.06176054 | AR | 1 |
| GO:0007080 | mitotic metaphase plate congression | 1/36 | 47/18866 | 0.085959641 | 0.127357223 | 0.06176054 | CCNB1 | 1 |
| GO:0010569 | regulation of double-strand break repair via homologous recombination | 1/36 | 47/18866 | 0.085959641 | 0.127357223 | 0.06176054 | CHEK1 | 1 |
| GO:0030225 | macrophage differentiation | 1/36 | 47/18866 | 0.085959641 | 0.127357223 | 0.06176054 | PRKCA | 1 |
| GO:0030261 | chromosome condensation | 1/36 | 47/18866 | 0.085959641 | 0.127357223 | 0.06176054 | CCNB1 | 1 |
| GO:0034204 | lipid translocation | 1/36 | 47/18866 | 0.085959641 | 0.127357223 | 0.06176054 | PRKCD | 1 |
| GO:0043268 | positive regulation of potassium ion transport | 1/36 | 47/18866 | 0.085959641 | 0.127357223 | 0.06176054 | KCNH2 | 1 |
| GO:0045933 | positive regulation of muscle contraction | 1/36 | 47/18866 | 0.085959641 | 0.127357223 | 0.06176054 | CHRM3 | 1 |
| GO:0060986 | endocrine hormone secretion | 1/36 | 47/18866 | 0.085959641 | 0.127357223 | 0.06176054 | IL1B | 1 |
| GO:1903115 | regulation of actin filament-based movement | 1/36 | 47/18866 | 0.085959641 | 0.127357223 | 0.06176054 | SCN5A | 1 |
| GO:0003007 | heart morphogenesis | 2/36 | 258/18866 | 0.086668301 | 0.128329722 | 0.062232143 | JUN/TP53 | 2 |
| GO:0008089 | anterograde axonal transport | 1/36 | 48/18866 | 0.087708164 | 0.129168292 | 0.062638799 | MAP2 | 1 |
| GO:0031952 | regulation of protein autophosphorylation | 1/36 | 48/18866 | 0.087708164 | 0.129168292 | 0.062638799 | JUN | 1 |
| GO:0042220 | response to cocaine | 1/36 | 48/18866 | 0.087708164 | 0.129168292 | 0.062638799 | OPRM1 | 1 |
| GO:0055023 | positive regulation of cardiac muscle tissue growth | 1/36 | 48/18866 | 0.087708164 | 0.129168292 | 0.062638799 | CCNB1 | 1 |
| GO:0060632 | regulation of microtubule-based movement | 1/36 | 48/18866 | 0.087708164 | 0.129168292 | 0.062638799 | MAP2 | 1 |
| GO:1900274 | regulation of phospholipase C activity | 1/36 | 48/18866 | 0.087708164 | 0.129168292 | 0.062638799 | ESR1 | 1 |
| GO:1904036 | negative regulation of epithelial cell apoptotic process | 1/36 | 48/18866 | 0.087708164 | 0.129168292 | 0.062638799 | KDR | 1 |
| GO:1990573 | potassium ion import across plasma membrane | 1/36 | 48/18866 | 0.087708164 | 0.129168292 | 0.062638799 | KCNH2 | 1 |
| GO:1990928 | response to amino acid starvation | 1/36 | 48/18866 | 0.087708164 | 0.129168292 | 0.062638799 | CDKN1A | 1 |
| GO:0001894 | tissue homeostasis | 2/36 | 261/18866 | 0.088389883 | 0.130094223 | 0.063087819 | PRKCA/BAX | 2 |
| GO:0006302 | double-strand break repair | 2/36 | 262/18866 | 0.088966066 | 0.130797012 | 0.063428629 | CHEK1/TP53 | 2 |
| GO:0002067 | glandular epithelial cell differentiation | 1/36 | 49/18866 | 0.089453435 | 0.130797012 | 0.063428629 | FASN | 1 |
| GO:0006692 | prostanoid metabolic process | 1/36 | 49/18866 | 0.089453435 | 0.130797012 | 0.063428629 | IL1B | 1 |
| GO:0006693 | prostaglandin metabolic process | 1/36 | 49/18866 | 0.089453435 | 0.130797012 | 0.063428629 | IL1B | 1 |
| GO:0009395 | phospholipid catabolic process | 1/36 | 49/18866 | 0.089453435 | 0.130797012 | 0.063428629 | PRKCD | 1 |
| GO:0015695 | organic cation transport | 1/36 | 49/18866 | 0.089453435 | 0.130797012 | 0.063428629 | SLC6A4 | 1 |
| GO:0032663 | regulation of interleukin-2 production | 1/36 | 49/18866 | 0.089453435 | 0.130797012 | 0.063428629 | IL1B | 1 |
| GO:0042149 | cellular response to glucose starvation | 1/36 | 49/18866 | 0.089453435 | 0.130797012 | 0.063428629 | TP53 | 1 |
| GO:0047496 | vesicle transport along microtubule | 1/36 | 49/18866 | 0.089453435 | 0.130797012 | 0.063428629 | MAP2 | 1 |
| GO:0061647 | histone H3-K9 modification | 1/36 | 49/18866 | 0.089453435 | 0.130797012 | 0.063428629 | CHEK1 | 1 |
| GO:0097720 | calcineurin-mediated signaling | 1/36 | 49/18866 | 0.089453435 | 0.130797012 | 0.063428629 | TNF | 1 |
| GO:0043406 | positive regulation of MAP kinase activity | 2/36 | 264/18866 | 0.090121877 | 0.131617613 | 0.063826571 | TNF/IL1B | 2 |
| GO:0048863 | stem cell differentiation | 2/36 | 264/18866 | 0.090121877 | 0.131617613 | 0.063826571 | ESR1/TP53 | 2 |
| GO:0002711 | positive regulation of T cell mediated immunity | 1/36 | 50/18866 | 0.091195459 | 0.132241496 | 0.064129117 | IL1B | 1 |
| GO:0008038 | neuron recognition | 1/36 | 50/18866 | 0.091195459 | 0.132241496 | 0.064129117 | CASP3 | 1 |
| GO:0010718 | positive regulation of epithelial to mesenchymal transition | 1/36 | 50/18866 | 0.091195459 | 0.132241496 | 0.064129117 | IL1B | 1 |
| GO:0019674 | NAD metabolic process | 1/36 | 50/18866 | 0.091195459 | 0.132241496 | 0.064129117 | TP53 | 1 |
| GO:0030195 | negative regulation of blood coagulation | 1/36 | 50/18866 | 0.091195459 | 0.132241496 | 0.064129117 | PRKCD | 1 |
| GO:0045540 | regulation of cholesterol biosynthetic process | 1/36 | 50/18866 | 0.091195459 | 0.132241496 | 0.064129117 | FASN | 1 |
| GO:0046850 | regulation of bone remodeling | 1/36 | 50/18866 | 0.091195459 | 0.132241496 | 0.064129117 | PRKCA | 1 |
| GO:0071715 | icosanoid transport | 1/36 | 50/18866 | 0.091195459 | 0.132241496 | 0.064129117 | IL1B | 1 |
| GO:0106118 | regulation of sterol biosynthetic process | 1/36 | 50/18866 | 0.091195459 | 0.132241496 | 0.064129117 | FASN | 1 |
| GO:1901571 | fatty acid derivative transport | 1/36 | 50/18866 | 0.091195459 | 0.132241496 | 0.064129117 | IL1B | 1 |
| GO:1903580 | positive regulation of ATP metabolic process | 1/36 | 50/18866 | 0.091195459 | 0.132241496 | 0.064129117 | CCNB1 | 1 |
| GO:1903727 | positive regulation of phospholipid metabolic process | 1/36 | 50/18866 | 0.091195459 | 0.132241496 | 0.064129117 | PRKCD | 1 |
| GO:0002762 | negative regulation of myeloid leukocyte differentiation | 1/36 | 51/18866 | 0.092934244 | 0.133892939 | 0.064929966 | MYC | 1 |
| GO:0006984 | ER-nucleus signaling pathway | 1/36 | 51/18866 | 0.092934244 | 0.133892939 | 0.064929966 | TP53 | 1 |
| GO:0031113 | regulation of microtubule polymerization | 1/36 | 51/18866 | 0.092934244 | 0.133892939 | 0.064929966 | MAP2 | 1 |
| GO:0032873 | negative regulation of stress-activated MAPK cascade | 1/36 | 51/18866 | 0.092934244 | 0.133892939 | 0.064929966 | MYC | 1 |
| GO:0035176 | social behavior | 1/36 | 51/18866 | 0.092934244 | 0.133892939 | 0.064929966 | SLC6A4 | 1 |
| GO:0035272 | exocrine system development | 1/36 | 51/18866 | 0.092934244 | 0.133892939 | 0.064929966 | TNF | 1 |
| GO:0038084 | vascular endothelial growth factor signaling pathway | 1/36 | 51/18866 | 0.092934244 | 0.133892939 | 0.064929966 | KDR | 1 |
| GO:0045912 | negative regulation of carbohydrate metabolic process | 1/36 | 51/18866 | 0.092934244 | 0.133892939 | 0.064929966 | TP53 | 1 |
| GO:0070303 | negative regulation of stress-activated protein kinase signaling cascade | 1/36 | 51/18866 | 0.092934244 | 0.133892939 | 0.064929966 | MYC | 1 |
| GO:0071675 | regulation of mononuclear cell migration | 1/36 | 51/18866 | 0.092934244 | 0.133892939 | 0.064929966 | TNF | 1 |
| GO:1900047 | negative regulation of hemostasis | 1/36 | 51/18866 | 0.092934244 | 0.133892939 | 0.064929966 | PRKCD | 1 |
| GO:0006839 | mitochondrial transport | 2/36 | 271/18866 | 0.094202746 | 0.135518567 | 0.065718297 | TP53/BAX | 2 |
| GO:0006301 | postreplication repair | 1/36 | 52/18866 | 0.094669793 | 0.135518567 | 0.065718297 | PCNA | 1 |
| GO:0010883 | regulation of lipid storage | 1/36 | 52/18866 | 0.094669793 | 0.135518567 | 0.065718297 | TNF | 1 |
| GO:0031103 | axon regeneration | 1/36 | 52/18866 | 0.094669793 | 0.135518567 | 0.065718297 | JUN | 1 |
| GO:0038066 | p38MAPK cascade | 1/36 | 52/18866 | 0.094669793 | 0.135518567 | 0.065718297 | IL1B | 1 |
| GO:0051445 | regulation of meiotic cell cycle | 1/36 | 52/18866 | 0.094669793 | 0.135518567 | 0.065718297 | PDE3A | 1 |
| GO:0060421 | positive regulation of heart growth | 1/36 | 52/18866 | 0.094669793 | 0.135518567 | 0.065718297 | CCNB1 | 1 |
| GO:0060425 | lung morphogenesis | 1/36 | 52/18866 | 0.094669793 | 0.135518567 | 0.065718297 | TNF | 1 |
| GO:0072132 | mesenchyme morphogenesis | 1/36 | 52/18866 | 0.094669793 | 0.135518567 | 0.065718297 | MYC | 1 |
| GO:0090329 | regulation of DNA-dependent DNA replication | 1/36 | 52/18866 | 0.094669793 | 0.135518567 | 0.065718297 | PCNA | 1 |
| GO:2001238 | positive regulation of extrinsic apoptotic signaling pathway | 1/36 | 52/18866 | 0.094669793 | 0.135518567 | 0.065718297 | TNF | 1 |
| GO:0000731 | DNA synthesis involved in DNA repair | 1/36 | 53/18866 | 0.096402114 | 0.136880966 | 0.066378979 | PCNA | 1 |
| GO:0032731 | positive regulation of interleukin-1 beta production | 1/36 | 53/18866 | 0.096402114 | 0.136880966 | 0.066378979 | TNF | 1 |
| GO:0035384 | thioester biosynthetic process | 1/36 | 53/18866 | 0.096402114 | 0.136880966 | 0.066378979 | FASN | 1 |
| GO:0043124 | negative regulation of I-kappaB kinase/NF-kappaB signaling | 1/36 | 53/18866 | 0.096402114 | 0.136880966 | 0.066378979 | ESR1 | 1 |
| GO:0043392 | negative regulation of DNA binding | 1/36 | 53/18866 | 0.096402114 | 0.136880966 | 0.066378979 | JUN | 1 |
| GO:0045599 | negative regulation of fat cell differentiation | 1/36 | 53/18866 | 0.096402114 | 0.136880966 | 0.066378979 | TNF | 1 |
| GO:0045661 | regulation of myoblast differentiation | 1/36 | 53/18866 | 0.096402114 | 0.136880966 | 0.066378979 | TNF | 1 |
| GO:0045668 | negative regulation of osteoblast differentiation | 1/36 | 53/18866 | 0.096402114 | 0.136880966 | 0.066378979 | TNF | 1 |
| GO:0045744 | negative regulation of G protein-coupled receptor signaling pathway | 1/36 | 53/18866 | 0.096402114 | 0.136880966 | 0.066378979 | OPRM1 | 1 |
| GO:0051703 | intraspecies interaction between organisms | 1/36 | 53/18866 | 0.096402114 | 0.136880966 | 0.066378979 | SLC6A4 | 1 |
| GO:0061756 | leukocyte adhesion to vascular endothelial cell | 1/36 | 53/18866 | 0.096402114 | 0.136880966 | 0.066378979 | TNF | 1 |
| GO:0071616 | acyl-CoA biosynthetic process | 1/36 | 53/18866 | 0.096402114 | 0.136880966 | 0.066378979 | FASN | 1 |
| GO:0097035 | regulation of membrane lipid distribution | 1/36 | 53/18866 | 0.096402114 | 0.136880966 | 0.066378979 | PRKCD | 1 |
| GO:2000772 | regulation of cellular senescence | 1/36 | 53/18866 | 0.096402114 | 0.136880966 | 0.066378979 | TP53 | 1 |
| GO:0010524 | positive regulation of calcium ion transport into cytosol | 1/36 | 54/18866 | 0.098131212 | 0.139175116 | 0.067491502 | BAX | 1 |
| GO:0032623 | interleukin-2 production | 1/36 | 54/18866 | 0.098131212 | 0.139175116 | 0.067491502 | IL1B | 1 |
| GO:0110053 | regulation of actin filament organization | 2/36 | 278/18866 | 0.098337299 | 0.139386876 | 0.067594193 | PRKCE/PRKCD | 2 |
| GO:0022409 | positive regulation of cell-cell adhesion | 2/36 | 279/18866 | 0.098932212 | 0.140149209 | 0.067963879 | TNF/IL1B | 2 |
| GO:0001954 | positive regulation of cell-matrix adhesion | 1/36 | 55/18866 | 0.099857094 | 0.141052454 | 0.068401897 | KDR | 1 |
| GO:0002720 | positive regulation of cytokine production involved in immune response | 1/36 | 55/18866 | 0.099857094 | 0.141052454 | 0.068401897 | IL1B | 1 |
| GO:0002931 | response to ischemia | 1/36 | 55/18866 | 0.099857094 | 0.141052454 | 0.068401897 | TP53 | 1 |
| GO:0050819 | negative regulation of coagulation | 1/36 | 55/18866 | 0.099857094 | 0.141052454 | 0.068401897 | PRKCD | 1 |
| GO:2000649 | regulation of sodium ion transmembrane transporter activity | 1/36 | 55/18866 | 0.099857094 | 0.141052454 | 0.068401897 | PRKCE | 1 |
| GO:0001822 | kidney development | 2/36 | 283/18866 | 0.101322283 | 0.142746605 | 0.069223458 | MYC/BAX | 2 |
| GO:0002712 | regulation of B cell mediated immunity | 1/36 | 56/18866 | 0.101579764 | 0.142746605 | 0.069223458 | TNF | 1 |
| GO:0002889 | regulation of immunoglobulin mediated immune response | 1/36 | 56/18866 | 0.101579764 | 0.142746605 | 0.069223458 | TNF | 1 |
| GO:0010665 | regulation of cardiac muscle cell apoptotic process | 1/36 | 56/18866 | 0.101579764 | 0.142746605 | 0.069223458 | TP53 | 1 |
| GO:0019320 | hexose catabolic process | 1/36 | 56/18866 | 0.101579764 | 0.142746605 | 0.069223458 | TP53 | 1 |
| GO:0030071 | regulation of mitotic metaphase/anaphase transition | 1/36 | 56/18866 | 0.101579764 | 0.142746605 | 0.069223458 | CCNB1 | 1 |
| GO:0050732 | negative regulation of peptidyl-tyrosine phosphorylation | 1/36 | 56/18866 | 0.101579764 | 0.142746605 | 0.069223458 | PRKCD | 1 |
| GO:0086065 | cell communication involved in cardiac conduction | 1/36 | 56/18866 | 0.101579764 | 0.142746605 | 0.069223458 | SCN5A | 1 |
| GO:0090183 | regulation of kidney development | 1/36 | 56/18866 | 0.101579764 | 0.142746605 | 0.069223458 | MYC | 1 |
| GO:0032615 | interleukin-12 production | 1/36 | 57/18866 | 0.103299228 | 0.144666061 | 0.070154278 | PRKCD | 1 |
| GO:0045840 | positive regulation of mitotic nuclear division | 1/36 | 57/18866 | 0.103299228 | 0.144666061 | 0.070154278 | IL1B | 1 |
| GO:0048016 | inositol phosphate-mediated signaling | 1/36 | 57/18866 | 0.103299228 | 0.144666061 | 0.070154278 | TNF | 1 |
| GO:0061178 | regulation of insulin secretion involved in cellular response to glucose stimulus | 1/36 | 57/18866 | 0.103299228 | 0.144666061 | 0.070154278 | PRKCE | 1 |
| GO:0097479 | synaptic vesicle localization | 1/36 | 57/18866 | 0.103299228 | 0.144666061 | 0.070154278 | MAP2 | 1 |
| GO:0098900 | regulation of action potential | 1/36 | 57/18866 | 0.103299228 | 0.144666061 | 0.070154278 | SCN5A | 1 |
| GO:0060047 | heart contraction | 2/36 | 287/18866 | 0.103728745 | 0.145184759 | 0.070405815 | SCN5A/KCNH2 | 2 |
| GO:0007091 | metaphase/anaphase transition of mitotic cell cycle | 1/36 | 58/18866 | 0.105015493 | 0.146401497 | 0.070995859 | CCNB1 | 1 |
| GO:0007566 | embryo implantation | 1/36 | 58/18866 | 0.105015493 | 0.146401497 | 0.070995859 | IL1B | 1 |
| GO:0010662 | regulation of striated muscle cell apoptotic process | 1/36 | 58/18866 | 0.105015493 | 0.146401497 | 0.070995859 | TP53 | 1 |
| GO:0035306 | positive regulation of dephosphorylation | 1/36 | 58/18866 | 0.105015493 | 0.146401497 | 0.070995859 | PRKCD | 1 |
| GO:1902099 | regulation of metaphase/anaphase transition of cell cycle | 1/36 | 58/18866 | 0.105015493 | 0.146401497 | 0.070995859 | CCNB1 | 1 |
| GO:1903749 | positive regulation of establishment of protein localization to mitochondrion | 1/36 | 58/18866 | 0.105015493 | 0.146401497 | 0.070995859 | TP53 | 1 |
| GO:2000272 | negative regulation of signaling receptor activity | 1/36 | 58/18866 | 0.105015493 | 0.146401497 | 0.070995859 | ESR2 | 1 |
| GO:0060485 | mesenchyme development | 2/36 | 290/18866 | 0.105544124 | 0.147054952 | 0.071312745 | MYC/IL1B | 2 |
| GO:0001658 | branching involved in ureteric bud morphogenesis | 1/36 | 59/18866 | 0.106728564 | 0.147658648 | 0.071605501 | MYC | 1 |
| GO:0006636 | unsaturated fatty acid biosynthetic process | 1/36 | 59/18866 | 0.106728564 | 0.147658648 | 0.071605501 | IL1B | 1 |
| GO:0010659 | cardiac muscle cell apoptotic process | 1/36 | 59/18866 | 0.106728564 | 0.147658648 | 0.071605501 | TP53 | 1 |
| GO:0031102 | neuron projection regeneration | 1/36 | 59/18866 | 0.106728564 | 0.147658648 | 0.071605501 | JUN | 1 |
| GO:0032387 | negative regulation of intracellular transport | 1/36 | 59/18866 | 0.106728564 | 0.147658648 | 0.071605501 | PKIA | 1 |
| GO:0045815 | positive regulation of gene expression, epigenetic | 1/36 | 59/18866 | 0.106728564 | 0.147658648 | 0.071605501 | CHEK1 | 1 |
| GO:0046456 | icosanoid biosynthetic process | 1/36 | 59/18866 | 0.106728564 | 0.147658648 | 0.071605501 | IL1B | 1 |
| GO:0046605 | regulation of centrosome cycle | 1/36 | 59/18866 | 0.106728564 | 0.147658648 | 0.071605501 | CHEK1 | 1 |
| GO:0048008 | platelet-derived growth factor receptor signaling pathway | 1/36 | 59/18866 | 0.106728564 | 0.147658648 | 0.071605501 | F7 | 1 |
| GO:0050707 | regulation of cytokine secretion | 1/36 | 59/18866 | 0.106728564 | 0.147658648 | 0.071605501 | TNF | 1 |
| GO:0060043 | regulation of cardiac muscle cell proliferation | 1/36 | 59/18866 | 0.106728564 | 0.147658648 | 0.071605501 | CCNB1 | 1 |
| GO:0098930 | axonal transport | 1/36 | 59/18866 | 0.106728564 | 0.147658648 | 0.071605501 | MAP2 | 1 |
| GO:0072001 | renal system development | 2/36 | 292/18866 | 0.106759307 | 0.147658648 | 0.071605501 | MYC/BAX | 2 |
| GO:0051604 | protein maturation | 2/36 | 293/18866 | 0.107368361 | 0.148417413 | 0.071973457 | F7/CASP3 | 2 |
| GO:0010823 | negative regulation of mitochondrion organization | 1/36 | 60/18866 | 0.108438447 | 0.149224433 | 0.072364812 | TP53 | 1 |
| GO:0019229 | regulation of vasoconstriction | 1/36 | 60/18866 | 0.108438447 | 0.149224433 | 0.072364812 | CHRM3 | 1 |
| GO:0030865 | cortical cytoskeleton organization | 1/36 | 60/18866 | 0.108438447 | 0.149224433 | 0.072364812 | TNF | 1 |
| GO:0032515 | negative regulation of phosphoprotein phosphatase activity | 1/36 | 60/18866 | 0.108438447 | 0.149224433 | 0.072364812 | TNF | 1 |
| GO:0032732 | positive regulation of interleukin-1 production | 1/36 | 60/18866 | 0.108438447 | 0.149224433 | 0.072364812 | TNF | 1 |
| GO:0033260 | nuclear DNA replication | 1/36 | 60/18866 | 0.108438447 | 0.149224433 | 0.072364812 | PCNA | 1 |
| GO:0043966 | histone H3 acetylation | 1/36 | 60/18866 | 0.108438447 | 0.149224433 | 0.072364812 | CHEK1 | 1 |
| GO:0044784 | metaphase/anaphase transition of cell cycle | 1/36 | 60/18866 | 0.108438447 | 0.149224433 | 0.072364812 | CCNB1 | 1 |
| GO:0051146 | striated muscle cell differentiation | 2/36 | 295/18866 | 0.108589363 | 0.149348396 | 0.072424927 | CCNB1/CASP3 | 2 |
| GO:0003015 | heart process | 2/36 | 297/18866 | 0.109814188 | 0.150309249 | 0.072890882 | SCN5A/KCNH2 | 2 |
| GO:0071356 | cellular response to tumor necrosis factor | 2/36 | 297/18866 | 0.109814188 | 0.150309249 | 0.072890882 | TP53/TNF | 2 |
| GO:0010574 | regulation of vascular endothelial growth factor production | 1/36 | 61/18866 | 0.110145148 | 0.150309249 | 0.072890882 | IL1B | 1 |
| GO:0010658 | striated muscle cell apoptotic process | 1/36 | 61/18866 | 0.110145148 | 0.150309249 | 0.072890882 | TP53 | 1 |
| GO:0010965 | regulation of mitotic sister chromatid separation | 1/36 | 61/18866 | 0.110145148 | 0.150309249 | 0.072890882 | CCNB1 | 1 |
| GO:0033013 | tetrapyrrole metabolic process | 1/36 | 61/18866 | 0.110145148 | 0.150309249 | 0.072890882 | PRSS1 | 1 |
| GO:0042733 | embryonic digit morphogenesis | 1/36 | 61/18866 | 0.110145148 | 0.150309249 | 0.072890882 | BAX | 1 |
| GO:0045071 | negative regulation of viral genome replication | 1/36 | 61/18866 | 0.110145148 | 0.150309249 | 0.072890882 | TNF | 1 |
| GO:0046324 | regulation of glucose import | 1/36 | 61/18866 | 0.110145148 | 0.150309249 | 0.072890882 | TNF | 1 |
| GO:0046847 | filopodium assembly | 1/36 | 61/18866 | 0.110145148 | 0.150309249 | 0.072890882 | PRKCD | 1 |
| GO:0046888 | negative regulation of hormone secretion | 1/36 | 61/18866 | 0.110145148 | 0.150309249 | 0.072890882 | IL1B | 1 |
| GO:0051310 | metaphase plate congression | 1/36 | 61/18866 | 0.110145148 | 0.150309249 | 0.072890882 | CCNB1 | 1 |
| GO:0061912 | selective autophagy | 1/36 | 61/18866 | 0.110145148 | 0.150309249 | 0.072890882 | TP53 | 1 |
| GO:0090342 | regulation of cell aging | 1/36 | 61/18866 | 0.110145148 | 0.150309249 | 0.072890882 | TP53 | 1 |
| GO:0007405 | neuroblast proliferation | 1/36 | 62/18866 | 0.111848673 | 0.152042354 | 0.073731333 | TP53 | 1 |
| GO:0010518 | positive regulation of phospholipase activity | 1/36 | 62/18866 | 0.111848673 | 0.152042354 | 0.073731333 | ESR1 | 1 |
| GO:0010676 | positive regulation of cellular carbohydrate metabolic process | 1/36 | 62/18866 | 0.111848673 | 0.152042354 | 0.073731333 | PRKCE | 1 |
| GO:0010803 | regulation of tumor necrosis factor-mediated signaling pathway | 1/36 | 62/18866 | 0.111848673 | 0.152042354 | 0.073731333 | TNF | 1 |
| GO:0051851 | modulation by host of symbiont process | 1/36 | 62/18866 | 0.111848673 | 0.152042354 | 0.073731333 | JUN | 1 |
| GO:0055025 | positive regulation of cardiac muscle tissue development | 1/36 | 62/18866 | 0.111848673 | 0.152042354 | 0.073731333 | CCNB1 | 1 |
| GO:0140115 | export across plasma membrane | 1/36 | 62/18866 | 0.111848673 | 0.152042354 | 0.073731333 | KCNH2 | 1 |
| GO:0007162 | negative regulation of cell adhesion | 2/36 | 301/18866 | 0.112275129 | 0.1525376 | 0.073971498 | PRKCD/CASP3 | 2 |
| GO:0032613 | interleukin-10 production | 1/36 | 63/18866 | 0.113549026 | 0.153927586 | 0.074645557 | PRKCD | 1 |
| GO:0046622 | positive regulation of organ growth | 1/36 | 63/18866 | 0.113549026 | 0.153927586 | 0.074645557 | CCNB1 | 1 |
| GO:0051893 | regulation of focal adhesion assembly | 1/36 | 63/18866 | 0.113549026 | 0.153927586 | 0.074645557 | KDR | 1 |
| GO:0090109 | regulation of cell-substrate junction assembly | 1/36 | 63/18866 | 0.113549026 | 0.153927586 | 0.074645557 | KDR | 1 |
| GO:0033344 | cholesterol efflux | 1/36 | 64/18866 | 0.115246215 | 0.155798159 | 0.075552671 | PON1 | 1 |
| GO:0042130 | negative regulation of T cell proliferation | 1/36 | 64/18866 | 0.115246215 | 0.155798159 | 0.075552671 | CASP3 | 1 |
| GO:0045453 | bone resorption | 1/36 | 64/18866 | 0.115246215 | 0.155798159 | 0.075552671 | PRKCA | 1 |
| GO:0051306 | mitotic sister chromatid separation | 1/36 | 64/18866 | 0.115246215 | 0.155798159 | 0.075552671 | CCNB1 | 1 |
| GO:1900449 | regulation of glutamate receptor signaling pathway | 1/36 | 64/18866 | 0.115246215 | 0.155798159 | 0.075552671 | OPRM1 | 1 |
| GO:0002440 | production of molecular mediator of immune response | 2/36 | 306/18866 | 0.115372019 | 0.155882392 | 0.075593519 | TNF/IL1B | 2 |
| GO:0042063 | gliogenesis | 2/36 | 307/18866 | 0.115994103 | 0.156636701 | 0.075959314 | TNF/IL1B | 2 |
| GO:0006940 | regulation of smooth muscle contraction | 1/36 | 65/18866 | 0.116940244 | 0.157050491 | 0.076159977 | CHRM3 | 1 |
| GO:0030837 | negative regulation of actin filament polymerization | 1/36 | 65/18866 | 0.116940244 | 0.157050491 | 0.076159977 | PRKCD | 1 |
| GO:0035308 | negative regulation of protein dephosphorylation | 1/36 | 65/18866 | 0.116940244 | 0.157050491 | 0.076159977 | TNF | 1 |
| GO:0035773 | insulin secretion involved in cellular response to glucose stimulus | 1/36 | 65/18866 | 0.116940244 | 0.157050491 | 0.076159977 | PRKCE | 1 |
| GO:0044786 | cell cycle DNA replication | 1/36 | 65/18866 | 0.116940244 | 0.157050491 | 0.076159977 | PCNA | 1 |
| GO:0046782 | regulation of viral transcription | 1/36 | 65/18866 | 0.116940244 | 0.157050491 | 0.076159977 | JUN | 1 |
| GO:0048645 | animal organ formation | 1/36 | 65/18866 | 0.116940244 | 0.157050491 | 0.076159977 | AR | 1 |
| GO:0060675 | ureteric bud morphogenesis | 1/36 | 65/18866 | 0.116940244 | 0.157050491 | 0.076159977 | MYC | 1 |
| GO:0090181 | regulation of cholesterol metabolic process | 1/36 | 65/18866 | 0.116940244 | 0.157050491 | 0.076159977 | FASN | 1 |
| GO:1905953 | negative regulation of lipid localization | 1/36 | 65/18866 | 0.116940244 | 0.157050491 | 0.076159977 | TNF | 1 |
| GO:0090287 | regulation of cellular response to growth factor stimulus | 2/36 | 310/18866 | 0.117865673 | 0.158206794 | 0.076720714 | TP53/IL1B | 2 |
| GO:0032715 | negative regulation of interleukin-6 production | 1/36 | 66/18866 | 0.118631119 | 0.158540772 | 0.076882672 | TNF | 1 |
| GO:0032922 | circadian regulation of gene expression | 1/36 | 66/18866 | 0.118631119 | 0.158540772 | 0.076882672 | NCOA2 | 1 |
| GO:0034394 | protein localization to cell surface | 1/36 | 66/18866 | 0.118631119 | 0.158540772 | 0.076882672 | TNF | 1 |
| GO:0046365 | monosaccharide catabolic process | 1/36 | 66/18866 | 0.118631119 | 0.158540772 | 0.076882672 | TP53 | 1 |
| GO:0072171 | mesonephric tubule morphogenesis | 1/36 | 66/18866 | 0.118631119 | 0.158540772 | 0.076882672 | MYC | 1 |
| GO:0150116 | regulation of cell-substrate junction organization | 1/36 | 66/18866 | 0.118631119 | 0.158540772 | 0.076882672 | KDR | 1 |
| GO:1905818 | regulation of chromosome separation | 1/36 | 66/18866 | 0.118631119 | 0.158540772 | 0.076882672 | CCNB1 | 1 |
| GO:2000378 | negative regulation of reactive oxygen species metabolic process | 1/36 | 66/18866 | 0.118631119 | 0.158540772 | 0.076882672 | TP53 | 1 |
| GO:0032890 | regulation of organic acid transport | 1/36 | 67/18866 | 0.120318847 | 0.159839161 | 0.077512313 | IL1B | 1 |
| GO:0033866 | nucleoside bisphosphate biosynthetic process | 1/36 | 67/18866 | 0.120318847 | 0.159839161 | 0.077512313 | FASN | 1 |
| GO:0034030 | ribonucleoside bisphosphate biosynthetic process | 1/36 | 67/18866 | 0.120318847 | 0.159839161 | 0.077512313 | FASN | 1 |
| GO:0034033 | purine nucleoside bisphosphate biosynthetic process | 1/36 | 67/18866 | 0.120318847 | 0.159839161 | 0.077512313 | FASN | 1 |
| GO:0036498 | IRE1-mediated unfolded protein response | 1/36 | 67/18866 | 0.120318847 | 0.159839161 | 0.077512313 | BAX | 1 |
| GO:0042255 | ribosome assembly | 1/36 | 67/18866 | 0.120318847 | 0.159839161 | 0.077512313 | EIF6 | 1 |
| GO:0045123 | cellular extravasation | 1/36 | 67/18866 | 0.120318847 | 0.159839161 | 0.077512313 | TNF | 1 |
| GO:0045670 | regulation of osteoclast differentiation | 1/36 | 67/18866 | 0.120318847 | 0.159839161 | 0.077512313 | TNF | 1 |
| GO:0071300 | cellular response to retinoic acid | 1/36 | 67/18866 | 0.120318847 | 0.159839161 | 0.077512313 | SLC6A4 | 1 |
| GO:1900015 | regulation of cytokine production involved in inflammatory response | 1/36 | 67/18866 | 0.120318847 | 0.159839161 | 0.077512313 | TNF | 1 |
| GO:1903672 | positive regulation of sprouting angiogenesis | 1/36 | 67/18866 | 0.120318847 | 0.159839161 | 0.077512313 | KDR | 1 |
| GO:0002637 | regulation of immunoglobulin production | 1/36 | 68/18866 | 0.122003432 | 0.161901852 | 0.078512593 | TNF | 1 |
| GO:0016239 | positive regulation of macroautophagy | 1/36 | 68/18866 | 0.122003432 | 0.161901852 | 0.078512593 | KDR | 1 |
| GO:0048662 | negative regulation of smooth muscle cell proliferation | 1/36 | 69/18866 | 0.123684881 | 0.163867449 | 0.079465788 | CDKN1A | 1 |
| GO:0060038 | cardiac muscle cell proliferation | 1/36 | 69/18866 | 0.123684881 | 0.163867449 | 0.079465788 | CCNB1 | 1 |
| GO:0071230 | cellular response to amino acid stimulus | 1/36 | 69/18866 | 0.123684881 | 0.163867449 | 0.079465788 | TNF | 1 |
| GO:0001756 | somitogenesis | 1/36 | 70/18866 | 0.125363199 | 0.165554951 | 0.080284125 | TP53 | 1 |
| GO:0002534 | cytokine production involved in inflammatory response | 1/36 | 70/18866 | 0.125363199 | 0.165554951 | 0.080284125 | TNF | 1 |
| GO:0006879 | cellular iron ion homeostasis | 1/36 | 70/18866 | 0.125363199 | 0.165554951 | 0.080284125 | MYC | 1 |
| GO:0008088 | axo-dendritic transport | 1/36 | 70/18866 | 0.125363199 | 0.165554951 | 0.080284125 | MAP2 | 1 |
| GO:0032374 | regulation of cholesterol transport | 1/36 | 70/18866 | 0.125363199 | 0.165554951 | 0.080284125 | PON1 | 1 |
| GO:0050771 | negative regulation of axonogenesis | 1/36 | 70/18866 | 0.125363199 | 0.165554951 | 0.080284125 | MAP2 | 1 |
| GO:0006470 | protein dephosphorylation | 2/36 | 323/18866 | 0.1260648 | 0.166391981 | 0.080690033 | TNF/PRKCD | 2 |
| GO:0032371 | regulation of sterol transport | 1/36 | 71/18866 | 0.127038392 | 0.167317196 | 0.081138707 | PON1 | 1 |
| GO:0042531 | positive regulation of tyrosine phosphorylation of STAT protein | 1/36 | 71/18866 | 0.127038392 | 0.167317196 | 0.081138707 | TNF | 1 |
| GO:0045739 | positive regulation of DNA repair | 1/36 | 71/18866 | 0.127038392 | 0.167317196 | 0.081138707 | PCNA | 1 |
| GO:0051785 | positive regulation of nuclear division | 1/36 | 71/18866 | 0.127038392 | 0.167317196 | 0.081138707 | IL1B | 1 |
| GO:0019226 | transmission of nerve impulse | 1/36 | 72/18866 | 0.128710465 | 0.16915642 | 0.082030619 | SCN5A | 1 |
| GO:0033047 | regulation of mitotic sister chromatid segregation | 1/36 | 72/18866 | 0.128710465 | 0.16915642 | 0.082030619 | CCNB1 | 1 |
| GO:0050663 | cytokine secretion | 1/36 | 72/18866 | 0.128710465 | 0.16915642 | 0.082030619 | TNF | 1 |
| GO:1904427 | positive regulation of calcium ion transmembrane transport | 1/36 | 72/18866 | 0.128710465 | 0.16915642 | 0.082030619 | BAX | 1 |
| GO:0002709 | regulation of T cell mediated immunity | 1/36 | 73/18866 | 0.130379425 | 0.17061913 | 0.082739945 | IL1B | 1 |
| GO:0006283 | transcription-coupled nucleotide-excision repair | 1/36 | 73/18866 | 0.130379425 | 0.17061913 | 0.082739945 | PCNA | 1 |
| GO:0010517 | regulation of phospholipase activity | 1/36 | 73/18866 | 0.130379425 | 0.17061913 | 0.082739945 | ESR1 | 1 |
| GO:0010611 | regulation of cardiac muscle hypertrophy | 1/36 | 73/18866 | 0.130379425 | 0.17061913 | 0.082739945 | PRKCA | 1 |
| GO:0051966 | regulation of synaptic transmission, glutamatergic | 1/36 | 73/18866 | 0.130379425 | 0.17061913 | 0.082739945 | TNF | 1 |
| GO:0072078 | nephron tubule morphogenesis | 1/36 | 73/18866 | 0.130379425 | 0.17061913 | 0.082739945 | MYC | 1 |
| GO:1902117 | positive regulation of organelle assembly | 1/36 | 73/18866 | 0.130379425 | 0.17061913 | 0.082739945 | TNF | 1 |
| GO:1903747 | regulation of establishment of protein localization to mitochondrion | 1/36 | 73/18866 | 0.130379425 | 0.17061913 | 0.082739945 | TP53 | 1 |
| GO:0032984 | protein-containing complex disassembly | 2/36 | 330/18866 | 0.130536797 | 0.170643151 | 0.082751593 | TNF/MYC | 2 |
| GO:0071375 | cellular response to peptide hormone stimulus | 2/36 | 330/18866 | 0.130536797 | 0.170643151 | 0.082751593 | PRKCD/IL1B | 2 |
| GO:0043010 | camera-type eye development | 2/36 | 332/18866 | 0.13182151 | 0.171791814 | 0.083308625 | JUN/BAX | 2 |
| GO:0050863 | regulation of T cell activation | 2/36 | 332/18866 | 0.13182151 | 0.171791814 | 0.083308625 | IL1B/CASP3 | 2 |
| GO:0006305 | DNA alkylation | 1/36 | 74/18866 | 0.132045276 | 0.171791814 | 0.083308625 | MYC | 1 |
| GO:0006306 | DNA methylation | 1/36 | 74/18866 | 0.132045276 | 0.171791814 | 0.083308625 | MYC | 1 |
| GO:0021536 | diencephalon development | 1/36 | 74/18866 | 0.132045276 | 0.171791814 | 0.083308625 | BAX | 1 |
| GO:0032024 | positive regulation of insulin secretion | 1/36 | 74/18866 | 0.132045276 | 0.171791814 | 0.083308625 | PRKCE | 1 |
| GO:0034121 | regulation of toll-like receptor signaling pathway | 1/36 | 74/18866 | 0.132045276 | 0.171791814 | 0.083308625 | ESR1 | 1 |
| GO:0042273 | ribosomal large subunit biogenesis | 1/36 | 74/18866 | 0.132045276 | 0.171791814 | 0.083308625 | EIF6 | 1 |
| GO:0043507 | positive regulation of JUN kinase activity | 1/36 | 74/18866 | 0.132045276 | 0.171791814 | 0.083308625 | TNF | 1 |
| GO:0006695 | cholesterol biosynthetic process | 1/36 | 75/18866 | 0.133708025 | 0.173312144 | 0.084045893 | FASN | 1 |
| GO:0035019 | somatic stem cell population maintenance | 1/36 | 75/18866 | 0.133708025 | 0.173312144 | 0.084045893 | MYC | 1 |
| GO:0035924 | cellular response to vascular endothelial growth factor stimulus | 1/36 | 75/18866 | 0.133708025 | 0.173312144 | 0.084045893 | KDR | 1 |
| GO:0046323 | glucose import | 1/36 | 75/18866 | 0.133708025 | 0.173312144 | 0.084045893 | TNF | 1 |
| GO:0072088 | nephron epithelium morphogenesis | 1/36 | 75/18866 | 0.133708025 | 0.173312144 | 0.084045893 | MYC | 1 |
| GO:0099518 | vesicle cytoskeletal trafficking | 1/36 | 75/18866 | 0.133708025 | 0.173312144 | 0.084045893 | MAP2 | 1 |
| GO:1902653 | secondary alcohol biosynthetic process | 1/36 | 75/18866 | 0.133708025 | 0.173312144 | 0.084045893 | FASN | 1 |
| GO:0014743 | regulation of muscle hypertrophy | 1/36 | 76/18866 | 0.135367676 | 0.175001393 | 0.084865077 | PRKCA | 1 |
| GO:0060193 | positive regulation of lipase activity | 1/36 | 76/18866 | 0.135367676 | 0.175001393 | 0.084865077 | ESR1 | 1 |
| GO:0071229 | cellular response to acid chemical | 1/36 | 76/18866 | 0.135367676 | 0.175001393 | 0.084865077 | TNF | 1 |
| GO:0072091 | regulation of stem cell proliferation | 1/36 | 76/18866 | 0.135367676 | 0.175001393 | 0.084865077 | TP53 | 1 |
| GO:1903524 | positive regulation of blood circulation | 1/36 | 76/18866 | 0.135367676 | 0.175001393 | 0.084865077 | CHRM3 | 1 |
| GO:0032413 | negative regulation of ion transmembrane transporter activity | 1/36 | 77/18866 | 0.137024236 | 0.176492392 | 0.085588121 | PRKCE | 1 |
| GO:0046785 | microtubule polymerization | 1/36 | 77/18866 | 0.137024236 | 0.176492392 | 0.085588121 | MAP2 | 1 |
| GO:0060411 | cardiac septum morphogenesis | 1/36 | 77/18866 | 0.137024236 | 0.176492392 | 0.085588121 | TP53 | 1 |
| GO:0061333 | renal tubule morphogenesis | 1/36 | 77/18866 | 0.137024236 | 0.176492392 | 0.085588121 | MYC | 1 |
| GO:0061418 | regulation of transcription from RNA polymerase II promoter in response to hypoxia | 1/36 | 77/18866 | 0.137024236 | 0.176492392 | 0.085588121 | TP53 | 1 |
| GO:0070830 | bicellular tight junction assembly | 1/36 | 77/18866 | 0.137024236 | 0.176492392 | 0.085588121 | TNF | 1 |
| GO:0072028 | nephron morphogenesis | 1/36 | 77/18866 | 0.137024236 | 0.176492392 | 0.085588121 | MYC | 1 |
| GO:0090150 | establishment of protein localization to membrane | 2/36 | 342/18866 | 0.138289826 | 0.178029116 | 0.086333339 | TP53/BAX | 2 |
| GO:0043900 | regulation of multi-organism process | 1/36 | 78/18866 | 0.13867771 | 0.178248052 | 0.08643951 | PDE3A | 1 |
| GO:0050688 | regulation of defense response to virus | 1/36 | 78/18866 | 0.13867771 | 0.178248052 | 0.08643951 | IL1B | 1 |
| GO:0060395 | SMAD protein signal transduction | 1/36 | 78/18866 | 0.13867771 | 0.178248052 | 0.08643951 | JUN | 1 |
| GO:0000422 | autophagy of mitochondrion | 1/36 | 79/18866 | 0.140328104 | 0.179429946 | 0.087012657 | TP53 | 1 |
| GO:0006446 | regulation of translational initiation | 1/36 | 79/18866 | 0.140328104 | 0.179429946 | 0.087012657 | TNF | 1 |
| GO:0016575 | histone deacetylation | 1/36 | 79/18866 | 0.140328104 | 0.179429946 | 0.087012657 | TP53 | 1 |
| GO:0043462 | regulation of ATPase activity | 1/36 | 79/18866 | 0.140328104 | 0.179429946 | 0.087012657 | TP53 | 1 |
| GO:0050000 | chromosome localization | 1/36 | 79/18866 | 0.140328104 | 0.179429946 | 0.087012657 | CCNB1 | 1 |
| GO:0050672 | negative regulation of lymphocyte proliferation | 1/36 | 79/18866 | 0.140328104 | 0.179429946 | 0.087012657 | CASP3 | 1 |
| GO:0051303 | establishment of chromosome localization | 1/36 | 79/18866 | 0.140328104 | 0.179429946 | 0.087012657 | CCNB1 | 1 |
| GO:0061726 | mitochondrion disassembly | 1/36 | 79/18866 | 0.140328104 | 0.179429946 | 0.087012657 | TP53 | 1 |
| GO:0099601 | regulation of neurotransmitter receptor activity | 1/36 | 79/18866 | 0.140328104 | 0.179429946 | 0.087012657 | OPRM1 | 1 |
| GO:0120192 | tight junction assembly | 1/36 | 79/18866 | 0.140328104 | 0.179429946 | 0.087012657 | TNF | 1 |
| GO:0003151 | outflow tract morphogenesis | 1/36 | 80/18866 | 0.141975424 | 0.180876837 | 0.087714311 | JUN | 1 |
| GO:0006110 | regulation of glycolytic process | 1/36 | 80/18866 | 0.141975424 | 0.180876837 | 0.087714311 | EIF6 | 1 |
| GO:0031397 | negative regulation of protein ubiquitination | 1/36 | 80/18866 | 0.141975424 | 0.180876837 | 0.087714311 | PRKCE | 1 |
| GO:0032204 | regulation of telomere maintenance | 1/36 | 80/18866 | 0.141975424 | 0.180876837 | 0.087714311 | MYC | 1 |
| GO:0032945 | negative regulation of mononuclear cell proliferation | 1/36 | 80/18866 | 0.141975424 | 0.180876837 | 0.087714311 | CASP3 | 1 |
| GO:0055021 | regulation of cardiac muscle tissue growth | 1/36 | 80/18866 | 0.141975424 | 0.180876837 | 0.087714311 | CCNB1 | 1 |
| GO:1900182 | positive regulation of protein localization to nucleus | 1/36 | 80/18866 | 0.141975424 | 0.180876837 | 0.087714311 | PRKCD | 1 |
| GO:0042742 | defense response to bacterium | 2/36 | 348/18866 | 0.142205199 | 0.181075604 | 0.087810701 | TNF/PRKCD | 2 |
| GO:0009615 | response to virus | 2/36 | 349/18866 | 0.142860172 | 0.181815305 | 0.088169411 | TNF/IL1B | 2 |
| GO:0046942 | carboxylic acid transport | 2/36 | 350/18866 | 0.143515821 | 0.182309358 | 0.088408998 | NCOA2/IL1B | 2 |
| GO:0001937 | negative regulation of endothelial cell proliferation | 1/36 | 81/18866 | 0.143619674 | 0.182309358 | 0.088408998 | TNF | 1 |
| GO:0016126 | sterol biosynthetic process | 1/36 | 81/18866 | 0.143619674 | 0.182309358 | 0.088408998 | FASN | 1 |
| GO:0021766 | hippocampus development | 1/36 | 81/18866 | 0.143619674 | 0.182309358 | 0.088408998 | CASP3 | 1 |
| GO:0043242 | negative regulation of protein-containing complex disassembly | 1/36 | 81/18866 | 0.143619674 | 0.182309358 | 0.088408998 | TNF | 1 |
| GO:0021954 | central nervous system neuron development | 1/36 | 82/18866 | 0.145260861 | 0.18391718 | 0.089188694 | MAP2 | 1 |
| GO:0022617 | extracellular matrix disassembly | 1/36 | 82/18866 | 0.145260861 | 0.18391718 | 0.089188694 | PRSS1 | 1 |
| GO:0045652 | regulation of megakaryocyte differentiation | 1/36 | 82/18866 | 0.145260861 | 0.18391718 | 0.089188694 | EIF6 | 1 |
| GO:0120193 | tight junction organization | 1/36 | 82/18866 | 0.145260861 | 0.18391718 | 0.089188694 | TNF | 1 |
| GO:1903313 | positive regulation of mRNA metabolic process | 1/36 | 82/18866 | 0.145260861 | 0.18391718 | 0.089188694 | CCNB1 | 1 |
| GO:0015849 | organic acid transport | 2/36 | 353/18866 | 0.145486776 | 0.184108266 | 0.089281359 | NCOA2/IL1B | 2 |
| GO:0003002 | regionalization | 2/36 | 355/18866 | 0.14680404 | 0.18513194 | 0.089777778 | AR/TP53 | 2 |
| GO:0071103 | DNA conformation change | 2/36 | 355/18866 | 0.14680404 | 0.18513194 | 0.089777778 | TP53/CCNB1 | 2 |
| GO:0001570 | vasculogenesis | 1/36 | 83/18866 | 0.146898989 | 0.18513194 | 0.089777778 | KDR | 1 |
| GO:0014855 | striated muscle cell proliferation | 1/36 | 83/18866 | 0.146898989 | 0.18513194 | 0.089777778 | CCNB1 | 1 |
| GO:0031145 | anaphase-promoting complex-dependent catabolic process | 1/36 | 83/18866 | 0.146898989 | 0.18513194 | 0.089777778 | CCNB1 | 1 |
| GO:0048041 | focal adhesion assembly | 1/36 | 83/18866 | 0.146898989 | 0.18513194 | 0.089777778 | KDR | 1 |
| GO:0055013 | cardiac muscle cell development | 1/36 | 83/18866 | 0.146898989 | 0.18513194 | 0.089777778 | CCNB1 | 1 |
| GO:0055117 | regulation of cardiac muscle contraction | 1/36 | 83/18866 | 0.146898989 | 0.18513194 | 0.089777778 | SCN5A | 1 |
| GO:0051251 | positive regulation of lymphocyte activation | 2/36 | 357/18866 | 0.148123895 | 0.186579867 | 0.090479936 | IL1B/CDKN1A | 2 |
| GO:0015844 | monoamine transport | 1/36 | 84/18866 | 0.148534065 | 0.186617774 | 0.090498318 | SLC6A4 | 1 |
| GO:0030279 | negative regulation of ossification | 1/36 | 84/18866 | 0.148534065 | 0.186617774 | 0.090498318 | TNF | 1 |
| GO:0033045 | regulation of sister chromatid segregation | 1/36 | 84/18866 | 0.148534065 | 0.186617774 | 0.090498318 | CCNB1 | 1 |
| GO:0045913 | positive regulation of carbohydrate metabolic process | 1/36 | 84/18866 | 0.148534065 | 0.186617774 | 0.090498318 | PRKCE | 1 |
| GO:0050886 | endocrine process | 1/36 | 84/18866 | 0.148534065 | 0.186617774 | 0.090498318 | IL1B | 1 |
| GO:0007178 | transmembrane receptor protein serine/threonine kinase signaling pathway | 2/36 | 359/18866 | 0.149446303 | 0.187421333 | 0.090887996 | JUN/TP53 | 2 |
| GO:0031589 | cell-substrate adhesion | 2/36 | 359/18866 | 0.149446303 | 0.187421333 | 0.090887996 | KDR/PRKCE | 2 |
| GO:0032956 | regulation of actin cytoskeleton organization | 2/36 | 360/18866 | 0.150108453 | 0.187421333 | 0.090887996 | PRKCE/PRKCD | 2 |
| GO:0006767 | water-soluble vitamin metabolic process | 1/36 | 85/18866 | 0.150166095 | 0.187421333 | 0.090887996 | PRSS1 | 1 |
| GO:0010507 | negative regulation of autophagy | 1/36 | 85/18866 | 0.150166095 | 0.187421333 | 0.090887996 | TP53 | 1 |
| GO:0031110 | regulation of microtubule polymerization or depolymerization | 1/36 | 85/18866 | 0.150166095 | 0.187421333 | 0.090887996 | MAP2 | 1 |
| GO:0034502 | protein localization to chromosome | 1/36 | 85/18866 | 0.150166095 | 0.187421333 | 0.090887996 | ESR1 | 1 |
| GO:0043154 | negative regulation of cysteine-type endopeptidase activity involved in apoptotic process | 1/36 | 85/18866 | 0.150166095 | 0.187421333 | 0.090887996 | TNF | 1 |
| GO:0043297 | apical junction assembly | 1/36 | 85/18866 | 0.150166095 | 0.187421333 | 0.090887996 | TNF | 1 |
| GO:0045844 | positive regulation of striated muscle tissue development | 1/36 | 85/18866 | 0.150166095 | 0.187421333 | 0.090887996 | CCNB1 | 1 |
| GO:0048636 | positive regulation of muscle organ development | 1/36 | 85/18866 | 0.150166095 | 0.187421333 | 0.090887996 | CCNB1 | 1 |
| GO:0072384 | organelle transport along microtubule | 1/36 | 85/18866 | 0.150166095 | 0.187421333 | 0.090887996 | MAP2 | 1 |
| GO:2000779 | regulation of double-strand break repair | 1/36 | 85/18866 | 0.150166095 | 0.187421333 | 0.090887996 | CHEK1 | 1 |
| GO:0006112 | energy reserve metabolic process | 1/36 | 86/18866 | 0.151795083 | 0.188782638 | 0.091548146 | MYC | 1 |
| GO:0009791 | post-embryonic development | 1/36 | 86/18866 | 0.151795083 | 0.188782638 | 0.091548146 | BAX | 1 |
| GO:0042509 | regulation of tyrosine phosphorylation of STAT protein | 1/36 | 86/18866 | 0.151795083 | 0.188782638 | 0.091548146 | TNF | 1 |
| GO:0045445 | myoblast differentiation | 1/36 | 86/18866 | 0.151795083 | 0.188782638 | 0.091548146 | TNF | 1 |
| GO:0070664 | negative regulation of leukocyte proliferation | 1/36 | 86/18866 | 0.151795083 | 0.188782638 | 0.091548146 | CASP3 | 1 |
| GO:1901863 | positive regulation of muscle tissue development | 1/36 | 86/18866 | 0.151795083 | 0.188782638 | 0.091548146 | CCNB1 | 1 |
| GO:1903901 | negative regulation of viral life cycle | 1/36 | 86/18866 | 0.151795083 | 0.188782638 | 0.091548146 | TNF | 1 |
| GO:0001942 | hair follicle development | 1/36 | 87/18866 | 0.153421034 | 0.19013056 | 0.092201807 | TNF | 1 |
| GO:0030512 | negative regulation of transforming growth factor beta receptor signaling pathway | 1/36 | 87/18866 | 0.153421034 | 0.19013056 | 0.092201807 | TP53 | 1 |
| GO:0051262 | protein tetramerization | 1/36 | 87/18866 | 0.153421034 | 0.19013056 | 0.092201807 | TP53 | 1 |
| GO:0055072 | iron ion homeostasis | 1/36 | 87/18866 | 0.153421034 | 0.19013056 | 0.092201807 | MYC | 1 |
| GO:0060218 | hematopoietic stem cell differentiation | 1/36 | 87/18866 | 0.153421034 | 0.19013056 | 0.092201807 | TP53 | 1 |
| GO:0060420 | regulation of heart growth | 1/36 | 87/18866 | 0.153421034 | 0.19013056 | 0.092201807 | CCNB1 | 1 |
| GO:0090049 | regulation of cell migration involved in sprouting angiogenesis | 1/36 | 87/18866 | 0.153421034 | 0.19013056 | 0.092201807 | KDR | 1 |
| GO:0032410 | negative regulation of transporter activity | 1/36 | 88/18866 | 0.155043956 | 0.191948014 | 0.093083162 | PRKCE | 1 |
| GO:0046470 | phosphatidylcholine metabolic process | 1/36 | 88/18866 | 0.155043956 | 0.191948014 | 0.093083162 | PON1 | 1 |
| GO:0045861 | negative regulation of proteolysis | 2/36 | 369/18866 | 0.156095357 | 0.193077188 | 0.093630743 | TP53/TNF | 2 |
| GO:0007260 | tyrosine phosphorylation of STAT protein | 1/36 | 89/18866 | 0.156663852 | 0.193077188 | 0.093630743 | TNF | 1 |
| GO:0022404 | molting cycle process | 1/36 | 89/18866 | 0.156663852 | 0.193077188 | 0.093630743 | TNF | 1 |
| GO:0022405 | hair cycle process | 1/36 | 89/18866 | 0.156663852 | 0.193077188 | 0.093630743 | TNF | 1 |
| GO:0043506 | regulation of JUN kinase activity | 1/36 | 89/18866 | 0.156663852 | 0.193077188 | 0.093630743 | TNF | 1 |
| GO:0043537 | negative regulation of blood vessel endothelial cell migration | 1/36 | 89/18866 | 0.156663852 | 0.193077188 | 0.093630743 | TNF | 1 |
| GO:0055006 | cardiac cell development | 1/36 | 89/18866 | 0.156663852 | 0.193077188 | 0.093630743 | CCNB1 | 1 |
| GO:0061053 | somite development | 1/36 | 89/18866 | 0.156663852 | 0.193077188 | 0.093630743 | TP53 | 1 |
| GO:1903845 | negative regulation of cellular response to transforming growth factor beta stimulus | 1/36 | 89/18866 | 0.156663852 | 0.193077188 | 0.093630743 | TP53 | 1 |
| GO:0032465 | regulation of cytokinesis | 1/36 | 90/18866 | 0.15828073 | 0.194776537 | 0.094454824 | PRKCE | 1 |
| GO:0046427 | positive regulation of receptor signaling pathway via JAK-STAT | 1/36 | 90/18866 | 0.15828073 | 0.194776537 | 0.094454824 | TNF | 1 |
| GO:0098773 | skin epidermis development | 1/36 | 90/18866 | 0.15828073 | 0.194776537 | 0.094454824 | TNF | 1 |
| GO:0001656 | metanephros development | 1/36 | 91/18866 | 0.159894593 | 0.195976648 | 0.095036805 | MYC | 1 |
| GO:0001843 | neural tube closure | 1/36 | 91/18866 | 0.159894593 | 0.195976648 | 0.095036805 | CASP3 | 1 |
| GO:0015914 | phospholipid transport | 1/36 | 91/18866 | 0.159894593 | 0.195976648 | 0.095036805 | PRKCD | 1 |
| GO:0033273 | response to vitamin | 1/36 | 91/18866 | 0.159894593 | 0.195976648 | 0.095036805 | F7 | 1 |
| GO:0045682 | regulation of epidermis development | 1/36 | 91/18866 | 0.159894593 | 0.195976648 | 0.095036805 | TNF | 1 |
| GO:0046849 | bone remodeling | 1/36 | 91/18866 | 0.159894593 | 0.195976648 | 0.095036805 | PRKCA | 1 |
| GO:0097306 | cellular response to alcohol | 1/36 | 91/18866 | 0.159894593 | 0.195976648 | 0.095036805 | PRKCE | 1 |
| GO:1901379 | regulation of potassium ion transmembrane transport | 1/36 | 91/18866 | 0.159894593 | 0.195976648 | 0.095036805 | KCNH2 | 1 |
| GO:0006402 | mRNA catabolic process | 2/36 | 376/18866 | 0.160784695 | 0.196969274 | 0.095518169 | PRKCD/PRKCA | 2 |
| GO:0006959 | humoral immune response | 2/36 | 377/18866 | 0.161456846 | 0.197261628 | 0.095659943 | TNF/IL1B | 2 |
| GO:0045582 | positive regulation of T cell differentiation | 1/36 | 92/18866 | 0.161505447 | 0.197261628 | 0.095659943 | IL1B | 1 |
| GO:0060606 | tube closure | 1/36 | 92/18866 | 0.161505447 | 0.197261628 | 0.095659943 | CASP3 | 1 |
| GO:1900407 | regulation of cellular response to oxidative stress | 1/36 | 92/18866 | 0.161505447 | 0.197261628 | 0.095659943 | TNF | 1 |
| GO:1903321 | negative regulation of protein modification by small protein conjugation or removal | 1/36 | 92/18866 | 0.161505447 | 0.197261628 | 0.095659943 | PRKCE | 1 |
| GO:1905897 | regulation of response to endoplasmic reticulum stress | 1/36 | 92/18866 | 0.161505447 | 0.197261628 | 0.095659943 | BAX | 1 |
| GO:0002532 | production of molecular mediator involved in inflammatory response | 1/36 | 93/18866 | 0.163113299 | 0.198731091 | 0.096372543 | TNF | 1 |
| GO:0051702 | interaction with symbiont | 1/36 | 93/18866 | 0.163113299 | 0.198731091 | 0.096372543 | JUN | 1 |
| GO:0072080 | nephron tubule development | 1/36 | 93/18866 | 0.163113299 | 0.198731091 | 0.096372543 | MYC | 1 |
| GO:1904894 | positive regulation of receptor signaling pathway via STAT | 1/36 | 93/18866 | 0.163113299 | 0.198731091 | 0.096372543 | TNF | 1 |
| GO:2000117 | negative regulation of cysteine-type endopeptidase activity | 1/36 | 93/18866 | 0.163113299 | 0.198731091 | 0.096372543 | TNF | 1 |
| GO:0010660 | regulation of muscle cell apoptotic process | 1/36 | 94/18866 | 0.164718153 | 0.200189636 | 0.097079849 | TP53 | 1 |
| GO:0051304 | chromosome separation | 1/36 | 94/18866 | 0.164718153 | 0.200189636 | 0.097079849 | CCNB1 | 1 |
| GO:0060993 | kidney morphogenesis | 1/36 | 94/18866 | 0.164718153 | 0.200189636 | 0.097079849 | MYC | 1 |
| GO:1901264 | carbohydrate derivative transport | 1/36 | 94/18866 | 0.164718153 | 0.200189636 | 0.097079849 | PRKCE | 1 |
| GO:1901992 | positive regulation of mitotic cell cycle phase transition | 1/36 | 94/18866 | 0.164718153 | 0.200189636 | 0.097079849 | CCNB1 | 1 |
| GO:0001654 | eye development | 2/36 | 384/18866 | 0.166177015 | 0.201637351 | 0.097781903 | JUN/BAX | 2 |
| GO:0003073 | regulation of systemic arterial blood pressure | 1/36 | 95/18866 | 0.166320014 | 0.201637351 | 0.097781903 | AR | 1 |
| GO:0045638 | negative regulation of myeloid cell differentiation | 1/36 | 95/18866 | 0.166320014 | 0.201637351 | 0.097781903 | MYC | 1 |
| GO:0061326 | renal tubule development | 1/36 | 95/18866 | 0.166320014 | 0.201637351 | 0.097781903 | MYC | 1 |
| GO:1903725 | regulation of phospholipid metabolic process | 1/36 | 95/18866 | 0.166320014 | 0.201637351 | 0.097781903 | PRKCD | 1 |
| GO:0002690 | positive regulation of leukocyte chemotaxis | 1/36 | 96/18866 | 0.167918889 | 0.203274592 | 0.098575866 | F7 | 1 |
| GO:0010972 | negative regulation of G2/M transition of mitotic cell cycle | 1/36 | 96/18866 | 0.167918889 | 0.203274592 | 0.098575866 | CDKN1A | 1 |
| GO:0035249 | synaptic transmission, glutamatergic | 1/36 | 96/18866 | 0.167918889 | 0.203274592 | 0.098575866 | TNF | 1 |
| GO:0150063 | visual system development | 2/36 | 388/18866 | 0.168885747 | 0.204199603 | 0.09902444 | JUN/BAX | 2 |
| GO:0006476 | protein deacetylation | 1/36 | 97/18866 | 0.169514783 | 0.204199603 | 0.09902444 | TP53 | 1 |
| GO:0030516 | regulation of axon extension | 1/36 | 97/18866 | 0.169514783 | 0.204199603 | 0.09902444 | MAP2 | 1 |
| GO:0032651 | regulation of interleukin-1 beta production | 1/36 | 97/18866 | 0.169514783 | 0.204199603 | 0.09902444 | TNF | 1 |
| GO:0036473 | cell death in response to oxidative stress | 1/36 | 97/18866 | 0.169514783 | 0.204199603 | 0.09902444 | PRKCD | 1 |
| GO:0042775 | mitochondrial ATP synthesis coupled electron transport | 1/36 | 97/18866 | 0.169514783 | 0.204199603 | 0.09902444 | CCNB1 | 1 |
| GO:0071674 | mononuclear cell migration | 1/36 | 97/18866 | 0.169514783 | 0.204199603 | 0.09902444 | TNF | 1 |
| GO:0098659 | inorganic cation import across plasma membrane | 1/36 | 97/18866 | 0.169514783 | 0.204199603 | 0.09902444 | KCNH2 | 1 |
| GO:0099587 | inorganic ion import across plasma membrane | 1/36 | 97/18866 | 0.169514783 | 0.204199603 | 0.09902444 | KCNH2 | 1 |
| GO:1902476 | chloride transmembrane transport | 1/36 | 97/18866 | 0.169514783 | 0.204199603 | 0.09902444 | GABRA1 | 1 |
| GO:0042692 | muscle cell differentiation | 2/36 | 390/18866 | 0.170243137 | 0.204911904 | 0.099369863 | CCNB1/CASP3 | 2 |
| GO:0001657 | ureteric bud development | 1/36 | 98/18866 | 0.1711077 | 0.204911904 | 0.099369863 | MYC | 1 |
| GO:0006942 | regulation of striated muscle contraction | 1/36 | 98/18866 | 0.1711077 | 0.204911904 | 0.099369863 | SCN5A | 1 |
| GO:0010657 | muscle cell apoptotic process | 1/36 | 98/18866 | 0.1711077 | 0.204911904 | 0.099369863 | TP53 | 1 |
| GO:0010771 | negative regulation of cell morphogenesis involved in differentiation | 1/36 | 98/18866 | 0.1711077 | 0.204911904 | 0.099369863 | MAP2 | 1 |
| GO:0014020 | primary neural tube formation | 1/36 | 98/18866 | 0.1711077 | 0.204911904 | 0.099369863 | CASP3 | 1 |
| GO:0030316 | osteoclast differentiation | 1/36 | 98/18866 | 0.1711077 | 0.204911904 | 0.099369863 | TNF | 1 |
| GO:0035278 | miRNA mediated inhibition of translation | 1/36 | 98/18866 | 0.1711077 | 0.204911904 | 0.099369863 | EIF6 | 1 |
| GO:0040033 | negative regulation of translation, ncRNA-mediated | 1/36 | 98/18866 | 0.1711077 | 0.204911904 | 0.099369863 | EIF6 | 1 |
| GO:0042773 | ATP synthesis coupled electron transport | 1/36 | 98/18866 | 0.1711077 | 0.204911904 | 0.099369863 | CCNB1 | 1 |
| GO:0044728 | DNA methylation or demethylation | 1/36 | 98/18866 | 0.1711077 | 0.204911904 | 0.099369863 | MYC | 1 |
| GO:0045974 | regulation of translation, ncRNA-mediated | 1/36 | 98/18866 | 0.1711077 | 0.204911904 | 0.099369863 | EIF6 | 1 |
| GO:0007044 | cell-substrate junction assembly | 1/36 | 99/18866 | 0.172697647 | 0.205911959 | 0.099854829 | KDR | 1 |
| GO:0010717 | regulation of epithelial to mesenchymal transition | 1/36 | 99/18866 | 0.172697647 | 0.205911959 | 0.099854829 | IL1B | 1 |
| GO:0043473 | pigmentation | 1/36 | 99/18866 | 0.172697647 | 0.205911959 | 0.099854829 | BAX | 1 |
| GO:0050830 | defense response to Gram-positive bacterium | 1/36 | 99/18866 | 0.172697647 | 0.205911959 | 0.099854829 | TNF | 1 |
| GO:0051817 | modulation of process of other organism involved in symbiotic interaction | 1/36 | 99/18866 | 0.172697647 | 0.205911959 | 0.099854829 | JUN | 1 |
| GO:0062207 | regulation of pattern recognition receptor signaling pathway | 1/36 | 99/18866 | 0.172697647 | 0.205911959 | 0.099854829 | ESR1 | 1 |
| GO:0072163 | mesonephric epithelium development | 1/36 | 99/18866 | 0.172697647 | 0.205911959 | 0.099854829 | MYC | 1 |
| GO:0072164 | mesonephric tubule development | 1/36 | 99/18866 | 0.172697647 | 0.205911959 | 0.099854829 | MYC | 1 |
| GO:0090277 | positive regulation of peptide hormone secretion | 1/36 | 99/18866 | 0.172697647 | 0.205911959 | 0.099854829 | PRKCE | 1 |
| GO:0048880 | sensory system development | 2/36 | 394/18866 | 0.172963797 | 0.206129185 | 0.099960171 | JUN/BAX | 2 |
| GO:0002702 | positive regulation of production of molecular mediator of immune response | 1/36 | 100/18866 | 0.174284629 | 0.207200369 | 0.10047963 | IL1B | 1 |
| GO:0030219 | megakaryocyte differentiation | 1/36 | 100/18866 | 0.174284629 | 0.207200369 | 0.10047963 | EIF6 | 1 |
| GO:0035282 | segmentation | 1/36 | 100/18866 | 0.174284629 | 0.207200369 | 0.10047963 | TP53 | 1 |
| GO:0045069 | regulation of viral genome replication | 1/36 | 100/18866 | 0.174284629 | 0.207200369 | 0.10047963 | TNF | 1 |
| GO:0050848 | regulation of calcium-mediated signaling | 1/36 | 100/18866 | 0.174284629 | 0.207200369 | 0.10047963 | TNF | 1 |
| GO:0042176 | regulation of protein catabolic process | 2/36 | 397/18866 | 0.175009305 | 0.207961202 | 0.100848588 | TNF/IL1B | 2 |
| GO:0001578 | microtubule bundle formation | 1/36 | 101/18866 | 0.17586865 | 0.208075921 | 0.10090422 | MAP2 | 1 |
| GO:0001708 | cell fate specification | 1/36 | 101/18866 | 0.17586865 | 0.208075921 | 0.10090422 | AR | 1 |
| GO:0007215 | glutamate receptor signaling pathway | 1/36 | 101/18866 | 0.17586865 | 0.208075921 | 0.10090422 | OPRM1 | 1 |
| GO:0015908 | fatty acid transport | 1/36 | 101/18866 | 0.17586865 | 0.208075921 | 0.10090422 | IL1B | 1 |
| GO:0030838 | positive regulation of actin filament polymerization | 1/36 | 101/18866 | 0.17586865 | 0.208075921 | 0.10090422 | PRKCE | 1 |
| GO:0031124 | mRNA 3'-end processing | 1/36 | 101/18866 | 0.17586865 | 0.208075921 | 0.10090422 | CCNB1 | 1 |
| GO:0042102 | positive regulation of T cell proliferation | 1/36 | 101/18866 | 0.17586865 | 0.208075921 | 0.10090422 | IL1B | 1 |
| GO:0060191 | regulation of lipase activity | 1/36 | 101/18866 | 0.17586865 | 0.208075921 | 0.10090422 | ESR1 | 1 |
| GO:1902882 | regulation of response to oxidative stress | 1/36 | 101/18866 | 0.17586865 | 0.208075921 | 0.10090422 | TNF | 1 |
| GO:0022600 | digestive system process | 1/36 | 102/18866 | 0.177449718 | 0.209643434 | 0.101664369 | CHRM3 | 1 |
| GO:0055024 | regulation of cardiac muscle tissue development | 1/36 | 102/18866 | 0.177449718 | 0.209643434 | 0.101664369 | CCNB1 | 1 |
| GO:0150115 | cell-substrate junction organization | 1/36 | 102/18866 | 0.177449718 | 0.209643434 | 0.101664369 | KDR | 1 |
| GO:0009150 | purine ribonucleotide metabolic process | 2/36 | 401/18866 | 0.177743123 | 0.209889065 | 0.101783485 | FASN/EIF6 | 2 |
| GO:0001823 | mesonephros development | 1/36 | 103/18866 | 0.179027836 | 0.210797764 | 0.102224149 | MYC | 1 |
| GO:0002042 | cell migration involved in sprouting angiogenesis | 1/36 | 103/18866 | 0.179027836 | 0.210797764 | 0.102224149 | KDR | 1 |
| GO:0030593 | neutrophil chemotaxis | 1/36 | 103/18866 | 0.179027836 | 0.210797764 | 0.102224149 | IL1B | 1 |
| GO:0070498 | interleukin-1-mediated signaling pathway | 1/36 | 103/18866 | 0.179027836 | 0.210797764 | 0.102224149 | IL1B | 1 |
| GO:0098869 | cellular oxidant detoxification | 1/36 | 103/18866 | 0.179027836 | 0.210797764 | 0.102224149 | TNF | 1 |
| GO:1901890 | positive regulation of cell junction assembly | 1/36 | 103/18866 | 0.179027836 | 0.210797764 | 0.102224149 | KDR | 1 |
| GO:0018205 | peptidyl-lysine modification | 2/36 | 405/18866 | 0.1804841 | 0.212042272 | 0.10282766 | CHEK1/IL1B | 2 |
| GO:0002456 | T cell mediated immunity | 1/36 | 104/18866 | 0.18060301 | 0.212042272 | 0.10282766 | IL1B | 1 |
| GO:0003300 | cardiac muscle hypertrophy | 1/36 | 104/18866 | 0.18060301 | 0.212042272 | 0.10282766 | PRKCA | 1 |
| GO:0006637 | acyl-CoA metabolic process | 1/36 | 104/18866 | 0.18060301 | 0.212042272 | 0.10282766 | FASN | 1 |
| GO:0010923 | negative regulation of phosphatase activity | 1/36 | 104/18866 | 0.18060301 | 0.212042272 | 0.10282766 | TNF | 1 |
| GO:0035383 | thioester metabolic process | 1/36 | 104/18866 | 0.18060301 | 0.212042272 | 0.10282766 | FASN | 1 |
| GO:0002696 | positive regulation of leukocyte activation | 2/36 | 406/18866 | 0.181170433 | 0.212606794 | 0.103101419 | IL1B/CDKN1A | 2 |
| GO:0007631 | feeding behavior | 1/36 | 105/18866 | 0.182175246 | 0.213276218 | 0.103426049 | OPRM1 | 1 |
| GO:0030301 | cholesterol transport | 1/36 | 105/18866 | 0.182175246 | 0.213276218 | 0.103426049 | PON1 | 1 |
| GO:0045621 | positive regulation of lymphocyte differentiation | 1/36 | 105/18866 | 0.182175246 | 0.213276218 | 0.103426049 | IL1B | 1 |
| GO:0062014 | negative regulation of small molecule metabolic process | 1/36 | 105/18866 | 0.182175246 | 0.213276218 | 0.103426049 | TP53 | 1 |
| GO:1902106 | negative regulation of leukocyte differentiation | 1/36 | 105/18866 | 0.182175246 | 0.213276218 | 0.103426049 | MYC | 1 |
| GO:0001841 | neural tube formation | 1/36 | 106/18866 | 0.183744549 | 0.214806128 | 0.104167962 | CASP3 | 1 |
| GO:0007218 | neuropeptide signaling pathway | 1/36 | 106/18866 | 0.183744549 | 0.214806128 | 0.104167962 | OPRM1 | 1 |
| GO:0019233 | sensory perception of pain | 1/36 | 106/18866 | 0.183744549 | 0.214806128 | 0.104167962 | OPRM1 | 1 |
| GO:0001503 | ossification | 2/36 | 412/18866 | 0.185297268 | 0.215712811 | 0.104607649 | TNF/FASN | 2 |
| GO:0000018 | regulation of DNA recombination | 1/36 | 107/18866 | 0.185310924 | 0.215712811 | 0.104607649 | CHEK1 | 1 |
| GO:0010596 | negative regulation of endothelial cell migration | 1/36 | 107/18866 | 0.185310924 | 0.215712811 | 0.104607649 | TNF | 1 |
| GO:0014897 | striated muscle hypertrophy | 1/36 | 107/18866 | 0.185310924 | 0.215712811 | 0.104607649 | PRKCA | 1 |
| GO:0035601 | protein deacylation | 1/36 | 107/18866 | 0.185310924 | 0.215712811 | 0.104607649 | TP53 | 1 |
| GO:0043266 | regulation of potassium ion transport | 1/36 | 107/18866 | 0.185310924 | 0.215712811 | 0.104607649 | KCNH2 | 1 |
| GO:0048524 | positive regulation of viral process | 1/36 | 107/18866 | 0.185310924 | 0.215712811 | 0.104607649 | JUN | 1 |
| GO:0051983 | regulation of chromosome segregation | 1/36 | 107/18866 | 0.185310924 | 0.215712811 | 0.104607649 | CCNB1 | 1 |
| GO:0055017 | cardiac muscle tissue growth | 1/36 | 107/18866 | 0.185310924 | 0.215712811 | 0.104607649 | CCNB1 | 1 |
| GO:0006024 | glycosaminoglycan biosynthetic process | 1/36 | 108/18866 | 0.186874377 | 0.217326667 | 0.105390272 | IL1B | 1 |
| GO:0032526 | response to retinoic acid | 1/36 | 108/18866 | 0.186874377 | 0.217326667 | 0.105390272 | SLC6A4 | 1 |
| GO:0006401 | RNA catabolic process | 2/36 | 415/18866 | 0.18736619 | 0.217795452 | 0.105617604 | PRKCD/PRKCA | 2 |
| GO:0009259 | ribonucleotide metabolic process | 2/36 | 416/18866 | 0.18805662 | 0.218417238 | 0.105919132 | FASN/EIF6 | 2 |
| GO:0002286 | T cell activation involved in immune response | 1/36 | 109/18866 | 0.188434912 | 0.218417238 | 0.105919132 | TP53 | 1 |
| GO:0006939 | smooth muscle contraction | 1/36 | 109/18866 | 0.188434912 | 0.218417238 | 0.105919132 | CHRM3 | 1 |
| GO:0014896 | muscle hypertrophy | 1/36 | 109/18866 | 0.188434912 | 0.218417238 | 0.105919132 | PRKCA | 1 |
| GO:0033138 | positive regulation of peptidyl-serine phosphorylation | 1/36 | 109/18866 | 0.188434912 | 0.218417238 | 0.105919132 | TNF | 1 |
| GO:0098781 | ncRNA transcription | 1/36 | 109/18866 | 0.188434912 | 0.218417238 | 0.105919132 | TP53 | 1 |
| GO:0072009 | nephron epithelium development | 1/36 | 110/18866 | 0.189992536 | 0.220014941 | 0.106693921 | MYC | 1 |
| GO:1901989 | positive regulation of cell cycle phase transition | 1/36 | 110/18866 | 0.189992536 | 0.220014941 | 0.106693921 | CCNB1 | 1 |
| GO:0050867 | positive regulation of cell activation | 2/36 | 421/18866 | 0.191514517 | 0.220877644 | 0.10711228 | IL1B/CDKN1A | 2 |
| GO:0006821 | chloride transport | 1/36 | 111/18866 | 0.191547252 | 0.220877644 | 0.10711228 | GABRA1 | 1 |
| GO:0032652 | regulation of interleukin-1 production | 1/36 | 111/18866 | 0.191547252 | 0.220877644 | 0.10711228 | TNF | 1 |
| GO:0035305 | negative regulation of dephosphorylation | 1/36 | 111/18866 | 0.191547252 | 0.220877644 | 0.10711228 | TNF | 1 |
| GO:0042303 | molting cycle | 1/36 | 111/18866 | 0.191547252 | 0.220877644 | 0.10711228 | TNF | 1 |
| GO:0042633 | hair cycle | 1/36 | 111/18866 | 0.191547252 | 0.220877644 | 0.10711228 | TNF | 1 |
| GO:0061387 | regulation of extent of cell growth | 1/36 | 111/18866 | 0.191547252 | 0.220877644 | 0.10711228 | MAP2 | 1 |
| GO:0098732 | macromolecule deacylation | 1/36 | 111/18866 | 0.191547252 | 0.220877644 | 0.10711228 | TP53 | 1 |
| GO:1903008 | organelle disassembly | 1/36 | 111/18866 | 0.191547252 | 0.220877644 | 0.10711228 | TP53 | 1 |
| GO:0043588 | skin development | 2/36 | 425/18866 | 0.194287504 | 0.223932311 | 0.108593608 | TNF/CASP3 | 2 |
| GO:0002223 | stimulatory C-type lectin receptor signaling pathway | 1/36 | 113/18866 | 0.194647987 | 0.224032259 | 0.108642077 | PRKCD | 1 |
| GO:0035821 | modulation of process of other organism | 1/36 | 113/18866 | 0.194647987 | 0.224032259 | 0.108642077 | JUN | 1 |
| GO:1903510 | mucopolysaccharide metabolic process | 1/36 | 113/18866 | 0.194647987 | 0.224032259 | 0.108642077 | IL1B | 1 |
| GO:0019693 | ribose phosphate metabolic process | 2/36 | 427/18866 | 0.195676137 | 0.225110082 | 0.109164756 | FASN/EIF6 | 2 |
| GO:0003279 | cardiac septum development | 1/36 | 114/18866 | 0.196194016 | 0.225494527 | 0.109351189 | TP53 | 1 |
| GO:1990748 | cellular detoxification | 1/36 | 114/18866 | 0.196194016 | 0.225494527 | 0.109351189 | TNF | 1 |
| GO:0006023 | aminoglycan biosynthetic process | 1/36 | 115/18866 | 0.197737159 | 0.226843329 | 0.110005276 | IL1B | 1 |
| GO:0006096 | glycolytic process | 1/36 | 115/18866 | 0.197737159 | 0.226843329 | 0.110005276 | EIF6 | 1 |
| GO:0030449 | regulation of complement activation | 1/36 | 115/18866 | 0.197737159 | 0.226843329 | 0.110005276 | IL1B | 1 |
| GO:0060419 | heart growth | 1/36 | 115/18866 | 0.197737159 | 0.226843329 | 0.110005276 | CCNB1 | 1 |
| GO:0002220 | innate immune response activating cell surface receptor signaling pathway | 1/36 | 116/18866 | 0.199277421 | 0.227971141 | 0.110552197 | PRKCD | 1 |
| GO:0006757 | ATP generation from ADP | 1/36 | 116/18866 | 0.199277421 | 0.227971141 | 0.110552197 | EIF6 | 1 |
| GO:0022904 | respiratory electron transport chain | 1/36 | 116/18866 | 0.199277421 | 0.227971141 | 0.110552197 | CCNB1 | 1 |
| GO:0032392 | DNA geometric change | 1/36 | 116/18866 | 0.199277421 | 0.227971141 | 0.110552197 | TP53 | 1 |
| GO:0046916 | cellular transition metal ion homeostasis | 1/36 | 116/18866 | 0.199277421 | 0.227971141 | 0.110552197 | MYC | 1 |
| GO:1903828 | negative regulation of cellular protein localization | 1/36 | 116/18866 | 0.199277421 | 0.227971141 | 0.110552197 | PKIA | 1 |
| GO:0032102 | negative regulation of response to external stimulus | 2/36 | 433/18866 | 0.199850238 | 0.228413563 | 0.110766744 | TNF/PRKCD | 2 |
| GO:0050808 | synapse organization | 2/36 | 433/18866 | 0.199850238 | 0.228413563 | 0.110766744 | TNF/GABRA1 | 2 |
| GO:0007015 | actin filament organization | 2/36 | 434/18866 | 0.200547078 | 0.229089384 | 0.111094477 | PRKCE/PRKCD | 2 |
| GO:0002758 | innate immune response-activating signal transduction | 1/36 | 117/18866 | 0.200814809 | 0.229089384 | 0.111094477 | PRKCD | 1 |
| GO:0031109 | microtubule polymerization or depolymerization | 1/36 | 117/18866 | 0.200814809 | 0.229089384 | 0.111094477 | MAP2 | 1 |
| GO:0043666 | regulation of phosphoprotein phosphatase activity | 1/36 | 117/18866 | 0.200814809 | 0.229089384 | 0.111094477 | TNF | 1 |
| GO:0007052 | mitotic spindle organization | 1/36 | 118/18866 | 0.202349326 | 0.230304866 | 0.111683912 | CCNB1 | 1 |
| GO:0015918 | sterol transport | 1/36 | 118/18866 | 0.202349326 | 0.230304866 | 0.111683912 | PON1 | 1 |
| GO:0021987 | cerebral cortex development | 1/36 | 118/18866 | 0.202349326 | 0.230304866 | 0.111683912 | BAX | 1 |
| GO:0033559 | unsaturated fatty acid metabolic process | 1/36 | 118/18866 | 0.202349326 | 0.230304866 | 0.111683912 | IL1B | 1 |
| GO:0050868 | negative regulation of T cell activation | 1/36 | 118/18866 | 0.202349326 | 0.230304866 | 0.111683912 | CASP3 | 1 |
| GO:0006304 | DNA modification | 1/36 | 120/18866 | 0.205409772 | 0.233679791 | 0.113320546 | MYC | 1 |
| GO:0006163 | purine nucleotide metabolic process | 2/36 | 442/18866 | 0.206133057 | 0.234394004 | 0.113666896 | FASN/EIF6 | 2 |
| GO:0007613 | memory | 1/36 | 121/18866 | 0.20693571 | 0.234871553 | 0.113898479 | SLC6A4 | 1 |
| GO:0030218 | erythrocyte differentiation | 1/36 | 121/18866 | 0.20693571 | 0.234871553 | 0.113898479 | CASP3 | 1 |
| GO:0098661 | inorganic anion transmembrane transport | 1/36 | 121/18866 | 0.20693571 | 0.234871553 | 0.113898479 | GABRA1 | 1 |
| GO:1900542 | regulation of purine nucleotide metabolic process | 1/36 | 121/18866 | 0.20693571 | 0.234871553 | 0.113898479 | EIF6 | 1 |
| GO:0042752 | regulation of circadian rhythm | 1/36 | 122/18866 | 0.2084588 | 0.236054591 | 0.11447218 | TP53 | 1 |
| GO:0043244 | regulation of protein-containing complex disassembly | 1/36 | 122/18866 | 0.2084588 | 0.236054591 | 0.11447218 | TNF | 1 |
| GO:0051928 | positive regulation of calcium ion transport | 1/36 | 122/18866 | 0.2084588 | 0.236054591 | 0.11447218 | BAX | 1 |
| GO:0097237 | cellular response to toxic substance | 1/36 | 122/18866 | 0.2084588 | 0.236054591 | 0.11447218 | TNF | 1 |
| GO:1990266 | neutrophil migration | 1/36 | 122/18866 | 0.2084588 | 0.236054591 | 0.11447218 | IL1B | 1 |
| GO:0001819 | positive regulation of cytokine production | 2/36 | 447/18866 | 0.20963391 | 0.237275818 | 0.115064401 | TNF/IL1B | 2 |
| GO:0006140 | regulation of nucleotide metabolic process | 1/36 | 123/18866 | 0.209979046 | 0.237338194 | 0.11509465 | EIF6 | 1 |
| GO:0048675 | axon extension | 1/36 | 123/18866 | 0.209979046 | 0.237338194 | 0.11509465 | MAP2 | 1 |
| GO:0071466 | cellular response to xenobiotic stimulus | 1/36 | 123/18866 | 0.209979046 | 0.237338194 | 0.11509465 | PCNA | 1 |
| GO:0002688 | regulation of leukocyte chemotaxis | 1/36 | 124/18866 | 0.211496452 | 0.238504268 | 0.115660125 | F7 | 1 |
| GO:0002698 | negative regulation of immune effector process | 1/36 | 124/18866 | 0.211496452 | 0.238504268 | 0.115660125 | TNF | 1 |
| GO:0010633 | negative regulation of epithelial cell migration | 1/36 | 124/18866 | 0.211496452 | 0.238504268 | 0.115660125 | TNF | 1 |
| GO:0046031 | ADP metabolic process | 1/36 | 124/18866 | 0.211496452 | 0.238504268 | 0.115660125 | EIF6 | 1 |
| GO:0051101 | regulation of DNA binding | 1/36 | 124/18866 | 0.211496452 | 0.238504268 | 0.115660125 | JUN | 1 |
| GO:0006417 | regulation of translation | 2/36 | 450/18866 | 0.211737747 | 0.238666744 | 0.115738916 | TNF/EIF6 | 2 |
| GO:0001838 | embryonic epithelial tube formation | 1/36 | 125/18866 | 0.213011025 | 0.239442339 | 0.116115033 | CASP3 | 1 |
| GO:0001952 | regulation of cell-matrix adhesion | 1/36 | 125/18866 | 0.213011025 | 0.239442339 | 0.116115033 | KDR | 1 |
| GO:0007098 | centrosome cycle | 1/36 | 125/18866 | 0.213011025 | 0.239442339 | 0.116115033 | CHEK1 | 1 |
| GO:0030968 | endoplasmic reticulum unfolded protein response | 1/36 | 125/18866 | 0.213011025 | 0.239442339 | 0.116115033 | BAX | 1 |
| GO:0034605 | cellular response to heat | 1/36 | 125/18866 | 0.213011025 | 0.239442339 | 0.116115033 | CDKN1A | 1 |
| GO:0048593 | camera-type eye morphogenesis | 1/36 | 125/18866 | 0.213011025 | 0.239442339 | 0.116115033 | BAX | 1 |
| GO:0044282 | small molecule catabolic process | 2/36 | 452/18866 | 0.213141632 | 0.2394795 | 0.116133054 | TP53/PON1 | 2 |
| GO:0055007 | cardiac muscle cell differentiation | 1/36 | 126/18866 | 0.21452277 | 0.240921043 | 0.116832115 | CCNB1 | 1 |
| GO:0006644 | phospholipid metabolic process | 2/36 | 455/18866 | 0.215249383 | 0.241516104 | 0.117120683 | PRKCD/PON1 | 2 |
| GO:0007389 | pattern specification process | 2/36 | 455/18866 | 0.215249383 | 0.241516104 | 0.117120683 | AR/TP53 | 2 |
| GO:0017015 | regulation of transforming growth factor beta receptor signaling pathway | 1/36 | 127/18866 | 0.216031691 | 0.241841223 | 0.117278346 | TP53 | 1 |
| GO:0019079 | viral genome replication | 1/36 | 127/18866 | 0.216031691 | 0.241841223 | 0.117278346 | TNF | 1 |
| GO:0030326 | embryonic limb morphogenesis | 1/36 | 127/18866 | 0.216031691 | 0.241841223 | 0.117278346 | BAX | 1 |
| GO:0035113 | embryonic appendage morphogenesis | 1/36 | 127/18866 | 0.216031691 | 0.241841223 | 0.117278346 | BAX | 1 |
| GO:0071621 | granulocyte chemotaxis | 1/36 | 127/18866 | 0.216031691 | 0.241841223 | 0.117278346 | IL1B | 1 |
| GO:0072089 | stem cell proliferation | 1/36 | 128/18866 | 0.217537794 | 0.243416264 | 0.118042146 | TP53 | 1 |
| GO:0034101 | erythrocyte homeostasis | 1/36 | 129/18866 | 0.219041083 | 0.244875164 | 0.118749624 | CASP3 | 1 |
| GO:1903844 | regulation of cellular response to transforming growth factor beta stimulus | 1/36 | 129/18866 | 0.219041083 | 0.244875164 | 0.118749624 | TP53 | 1 |
| GO:0009410 | response to xenobiotic stimulus | 1/36 | 130/18866 | 0.220541565 | 0.246328272 | 0.119454293 | PCNA | 1 |
| GO:0071426 | ribonucleoprotein complex export from nucleus | 1/36 | 130/18866 | 0.220541565 | 0.246328272 | 0.119454293 | EIF6 | 1 |
| GO:0003206 | cardiac chamber morphogenesis | 1/36 | 131/18866 | 0.222039243 | 0.247325926 | 0.119938095 | TP53 | 1 |
| GO:0003231 | cardiac ventricle development | 1/36 | 131/18866 | 0.222039243 | 0.247325926 | 0.119938095 | SCN5A | 1 |
| GO:0010508 | positive regulation of autophagy | 1/36 | 131/18866 | 0.222039243 | 0.247325926 | 0.119938095 | KDR | 1 |
| GO:0045667 | regulation of osteoblast differentiation | 1/36 | 131/18866 | 0.222039243 | 0.247325926 | 0.119938095 | TNF | 1 |
| GO:0046887 | positive regulation of hormone secretion | 1/36 | 131/18866 | 0.222039243 | 0.247325926 | 0.119938095 | PRKCE | 1 |
| GO:0071166 | ribonucleoprotein complex localization | 1/36 | 131/18866 | 0.222039243 | 0.247325926 | 0.119938095 | EIF6 | 1 |
| GO:0006165 | nucleoside diphosphate phosphorylation | 1/36 | 133/18866 | 0.225026211 | 0.250312347 | 0.121386328 | EIF6 | 1 |
| GO:0072175 | epithelial tube formation | 1/36 | 133/18866 | 0.225026211 | 0.250312347 | 0.121386328 | CASP3 | 1 |
| GO:0090101 | negative regulation of transmembrane receptor protein serine/threonine kinase signaling pathway | 1/36 | 133/18866 | 0.225026211 | 0.250312347 | 0.121386328 | TP53 | 1 |
| GO:0002697 | regulation of immune effector process | 2/36 | 470/18866 | 0.225819717 | 0.251081253 | 0.121759201 | TNF/IL1B | 2 |
| GO:0034754 | cellular hormone metabolic process | 1/36 | 134/18866 | 0.226515511 | 0.251513153 | 0.121968646 | ESR1 | 1 |
| GO:0045995 | regulation of embryonic development | 1/36 | 134/18866 | 0.226515511 | 0.251513153 | 0.121968646 | AR | 1 |
| GO:0071333 | cellular response to glucose stimulus | 1/36 | 134/18866 | 0.226515511 | 0.251513153 | 0.121968646 | PRKCE | 1 |
| GO:0072521 | purine-containing compound metabolic process | 2/36 | 472/18866 | 0.227232697 | 0.252195421 | 0.122299505 | FASN/EIF6 | 2 |
| GO:0015748 | organophosphate ester transport | 1/36 | 135/18866 | 0.228002029 | 0.252706537 | 0.122547366 | PRKCD | 1 |
| GO:0046939 | nucleotide phosphorylation | 1/36 | 135/18866 | 0.228002029 | 0.252706537 | 0.122547366 | EIF6 | 1 |
| GO:0050853 | B cell receptor signaling pathway | 1/36 | 135/18866 | 0.228002029 | 0.252706537 | 0.122547366 | BAX | 1 |
| GO:0009135 | purine nucleoside diphosphate metabolic process | 1/36 | 136/18866 | 0.229485768 | 0.253892547 | 0.123122508 | EIF6 | 1 |
| GO:0009179 | purine ribonucleoside diphosphate metabolic process | 1/36 | 136/18866 | 0.229485768 | 0.253892547 | 0.123122508 | EIF6 | 1 |
| GO:0031023 | microtubule organizing center organization | 1/36 | 136/18866 | 0.229485768 | 0.253892547 | 0.123122508 | CHEK1 | 1 |
| GO:0071331 | cellular response to hexose stimulus | 1/36 | 136/18866 | 0.229485768 | 0.253892547 | 0.123122508 | PRKCE | 1 |
| GO:0008544 | epidermis development | 2/36 | 477/18866 | 0.23076844 | 0.254956536 | 0.123638479 | TNF/CASP3 | 2 |
| GO:0030534 | adult behavior | 1/36 | 137/18866 | 0.230966735 | 0.254956536 | 0.123638479 | OPRM1 | 1 |
| GO:0032273 | positive regulation of protein polymerization | 1/36 | 137/18866 | 0.230966735 | 0.254956536 | 0.123638479 | PRKCE | 1 |
| GO:0071326 | cellular response to monosaccharide stimulus | 1/36 | 137/18866 | 0.230966735 | 0.254956536 | 0.123638479 | PRKCE | 1 |
| GO:1903038 | negative regulation of leukocyte cell-cell adhesion | 1/36 | 137/18866 | 0.230966735 | 0.254956536 | 0.123638479 | CASP3 | 1 |
| GO:0000724 | double-strand break repair via homologous recombination | 1/36 | 138/18866 | 0.232444935 | 0.255897899 | 0.124094983 | CHEK1 | 1 |
| GO:0033865 | nucleoside bisphosphate metabolic process | 1/36 | 138/18866 | 0.232444935 | 0.255897899 | 0.124094983 | FASN | 1 |
| GO:0033875 | ribonucleoside bisphosphate metabolic process | 1/36 | 138/18866 | 0.232444935 | 0.255897899 | 0.124094983 | FASN | 1 |
| GO:0034032 | purine nucleoside bisphosphate metabolic process | 1/36 | 138/18866 | 0.232444935 | 0.255897899 | 0.124094983 | FASN | 1 |
| GO:0055076 | transition metal ion homeostasis | 1/36 | 138/18866 | 0.232444935 | 0.255897899 | 0.124094983 | MYC | 1 |
| GO:0098754 | detoxification | 1/36 | 138/18866 | 0.232444935 | 0.255897899 | 0.124094983 | TNF | 1 |
| GO:0009185 | ribonucleoside diphosphate metabolic process | 1/36 | 139/18866 | 0.233920372 | 0.257406774 | 0.124826696 | EIF6 | 1 |
| GO:0007409 | axonogenesis | 2/36 | 482/18866 | 0.23430857 | 0.257718432 | 0.124977831 | PRKCA/MAP2 | 2 |
| GO:0000725 | recombinational repair | 1/36 | 140/18866 | 0.235393051 | 0.258679472 | 0.125443877 | CHEK1 | 1 |
| GO:0048284 | organelle fusion | 1/36 | 140/18866 | 0.235393051 | 0.258679472 | 0.125443877 | BAX | 1 |
| GO:0030010 | establishment of cell polarity | 1/36 | 141/18866 | 0.236862978 | 0.260178349 | 0.126170742 | MAP2 | 1 |
| GO:0046425 | regulation of receptor signaling pathway via JAK-STAT | 1/36 | 142/18866 | 0.238330157 | 0.261555894 | 0.126838768 | TNF | 1 |
| GO:0072073 | kidney epithelium development | 1/36 | 142/18866 | 0.238330157 | 0.261555894 | 0.126838768 | MYC | 1 |
| GO:0045598 | regulation of fat cell differentiation | 1/36 | 143/18866 | 0.239794594 | 0.262927972 | 0.127504142 | TNF | 1 |
| GO:0072006 | nephron development | 1/36 | 143/18866 | 0.239794594 | 0.262927972 | 0.127504142 | MYC | 1 |
| GO:0002218 | activation of innate immune response | 1/36 | 144/18866 | 0.241256293 | 0.264176717 | 0.128109708 | PRKCD | 1 |
| GO:0006405 | RNA export from nucleus | 1/36 | 144/18866 | 0.241256293 | 0.264176717 | 0.128109708 | EIF6 | 1 |
| GO:1902850 | microtubule cytoskeleton organization involved in mitosis | 1/36 | 144/18866 | 0.241256293 | 0.264176717 | 0.128109708 | CCNB1 | 1 |
| GO:0007612 | learning | 1/36 | 145/18866 | 0.24271526 | 0.265419137 | 0.128712207 | JUN | 1 |
| GO:0038127 | ERBB signaling pathway | 1/36 | 145/18866 | 0.24271526 | 0.265419137 | 0.128712207 | PRKCA | 1 |
| GO:0071322 | cellular response to carbohydrate stimulus | 1/36 | 145/18866 | 0.24271526 | 0.265419137 | 0.128712207 | PRKCE | 1 |
| GO:0034620 | cellular response to unfolded protein | 1/36 | 146/18866 | 0.244171499 | 0.266773934 | 0.129369201 | BAX | 1 |
| GO:0048813 | dendrite morphogenesis | 1/36 | 146/18866 | 0.244171499 | 0.266773934 | 0.129369201 | MAP2 | 1 |
| GO:0022604 | regulation of cell morphogenesis | 2/36 | 499/18866 | 0.246372638 | 0.269059087 | 0.130477362 | KDR/MAP2 | 2 |
| GO:0031644 | regulation of nervous system process | 1/36 | 148/18866 | 0.247075814 | 0.269467402 | 0.13067537 | OPRM1 | 1 |
| GO:1903670 | regulation of sprouting angiogenesis | 1/36 | 148/18866 | 0.247075814 | 0.269467402 | 0.13067537 | KDR | 1 |
| GO:2001251 | negative regulation of chromosome organization | 1/36 | 148/18866 | 0.247075814 | 0.269467402 | 0.13067537 | CCNB1 | 1 |
| GO:0006119 | oxidative phosphorylation | 1/36 | 149/18866 | 0.2485239 | 0.270926365 | 0.131382879 | CCNB1 | 1 |
| GO:0035107 | appendage morphogenesis | 1/36 | 150/18866 | 0.249969278 | 0.271777935 | 0.131795838 | BAX | 1 |
| GO:0035108 | limb morphogenesis | 1/36 | 150/18866 | 0.249969278 | 0.271777935 | 0.131795838 | BAX | 1 |
| GO:0035148 | tube formation | 1/36 | 150/18866 | 0.249969278 | 0.271777935 | 0.131795838 | CASP3 | 1 |
| GO:0045580 | regulation of T cell differentiation | 1/36 | 150/18866 | 0.249969278 | 0.271777935 | 0.131795838 | IL1B | 1 |
| GO:0060041 | retina development in camera-type eye | 1/36 | 150/18866 | 0.249969278 | 0.271777935 | 0.131795838 | BAX | 1 |
| GO:0097530 | granulocyte migration | 1/36 | 150/18866 | 0.249969278 | 0.271777935 | 0.131795838 | IL1B | 1 |
| GO:0006261 | DNA-dependent DNA replication | 1/36 | 151/18866 | 0.251411953 | 0.272983788 | 0.132380604 | PCNA | 1 |
| GO:0050871 | positive regulation of B cell activation | 1/36 | 151/18866 | 0.251411953 | 0.272983788 | 0.132380604 | CDKN1A | 1 |
| GO:1904892 | regulation of receptor signaling pathway via STAT | 1/36 | 151/18866 | 0.251411953 | 0.272983788 | 0.132380604 | TNF | 1 |
| GO:0001837 | epithelial to mesenchymal transition | 1/36 | 152/18866 | 0.25285193 | 0.274062467 | 0.132903698 | IL1B | 1 |
| GO:0006090 | pyruvate metabolic process | 1/36 | 152/18866 | 0.25285193 | 0.274062467 | 0.132903698 | EIF6 | 1 |
| GO:0007605 | sensory perception of sound | 1/36 | 152/18866 | 0.25285193 | 0.274062467 | 0.132903698 | CASP3 | 1 |
| GO:0050684 | regulation of mRNA processing | 1/36 | 152/18866 | 0.25285193 | 0.274062467 | 0.132903698 | CCNB1 | 1 |
| GO:0001678 | cellular glucose homeostasis | 1/36 | 153/18866 | 0.254289214 | 0.27525574 | 0.133482364 | PRKCE | 1 |
| GO:0016202 | regulation of striated muscle tissue development | 1/36 | 153/18866 | 0.254289214 | 0.27525574 | 0.133482364 | CCNB1 | 1 |
| GO:0031123 | RNA 3'-end processing | 1/36 | 153/18866 | 0.254289214 | 0.27525574 | 0.133482364 | CCNB1 | 1 |
| GO:0010977 | negative regulation of neuron projection development | 1/36 | 154/18866 | 0.255723809 | 0.276564736 | 0.134117147 | MAP2 | 1 |
| GO:0051250 | negative regulation of lymphocyte activation | 1/36 | 154/18866 | 0.255723809 | 0.276564736 | 0.134117147 | CASP3 | 1 |
| GO:0002455 | humoral immune response mediated by circulating immunoglobulin | 1/36 | 155/18866 | 0.257155722 | 0.277868528 | 0.134749407 | TNF | 1 |
| GO:0009132 | nucleoside diphosphate metabolic process | 1/36 | 155/18866 | 0.257155722 | 0.277868528 | 0.134749407 | EIF6 | 1 |
| GO:0008360 | regulation of cell shape | 1/36 | 156/18866 | 0.258584955 | 0.278921821 | 0.135260191 | KDR | 1 |
| GO:0034401 | chromatin organization involved in regulation of transcription | 1/36 | 156/18866 | 0.258584955 | 0.278921821 | 0.135260191 | CHEK1 | 1 |
| GO:0048592 | eye morphogenesis | 1/36 | 156/18866 | 0.258584955 | 0.278921821 | 0.135260191 | BAX | 1 |
| GO:1901861 | regulation of muscle tissue development | 1/36 | 156/18866 | 0.258584955 | 0.278921821 | 0.135260191 | CCNB1 | 1 |
| GO:0048634 | regulation of muscle organ development | 1/36 | 157/18866 | 0.260011516 | 0.280091387 | 0.135827359 | CCNB1 | 1 |
| GO:0098739 | import across plasma membrane | 1/36 | 157/18866 | 0.260011516 | 0.280091387 | 0.135827359 | KCNH2 | 1 |
| GO:1903900 | regulation of viral life cycle | 1/36 | 157/18866 | 0.260011516 | 0.280091387 | 0.135827359 | TNF | 1 |
| GO:0030203 | glycosaminoglycan metabolic process | 1/36 | 158/18866 | 0.261435407 | 0.281255006 | 0.136391644 | IL1B | 1 |
| GO:0035051 | cardiocyte differentiation | 1/36 | 158/18866 | 0.261435407 | 0.281255006 | 0.136391644 | CCNB1 | 1 |
| GO:1903707 | negative regulation of hemopoiesis | 1/36 | 158/18866 | 0.261435407 | 0.281255006 | 0.136391644 | MYC | 1 |
| GO:0050777 | negative regulation of immune response | 1/36 | 159/18866 | 0.262856635 | 0.282536356 | 0.137013021 | TNF | 1 |
| GO:0055067 | monovalent inorganic cation homeostasis | 1/36 | 159/18866 | 0.262856635 | 0.282536356 | 0.137013021 | KCNH2 | 1 |
| GO:0000070 | mitotic sister chromatid segregation | 1/36 | 161/18866 | 0.265691117 | 0.285333199 | 0.138369321 | CCNB1 | 1 |
| GO:0010970 | transport along microtubule | 1/36 | 161/18866 | 0.265691117 | 0.285333199 | 0.138369321 | MAP2 | 1 |
| GO:0007338 | single fertilization | 1/36 | 162/18866 | 0.267104382 | 0.2866002 | 0.13898374 | AR | 1 |
| GO:0019827 | stem cell population maintenance | 1/36 | 162/18866 | 0.267104382 | 0.2866002 | 0.13898374 | MYC | 1 |
| GO:0021915 | neural tube development | 1/36 | 163/18866 | 0.268515003 | 0.287987912 | 0.139656696 | CASP3 | 1 |
| GO:0001764 | neuron migration | 1/36 | 164/18866 | 0.269922983 | 0.289119077 | 0.140205242 | BAX | 1 |
| GO:0007259 | receptor signaling pathway via JAK-STAT | 1/36 | 164/18866 | 0.269922983 | 0.289119077 | 0.140205242 | TNF | 1 |
| GO:0098727 | maintenance of cell number | 1/36 | 164/18866 | 0.269922983 | 0.289119077 | 0.140205242 | MYC | 1 |
| GO:0016482 | cytosolic transport | 1/36 | 165/18866 | 0.271328329 | 0.290497622 | 0.140873753 | MAP2 | 1 |
| GO:0009408 | response to heat | 1/36 | 166/18866 | 0.272731044 | 0.291617906 | 0.141417023 | CDKN1A | 1 |
| GO:0035967 | cellular response to topologically incorrect protein | 1/36 | 166/18866 | 0.272731044 | 0.291617906 | 0.141417023 | BAX | 1 |
| GO:0048515 | spermatid differentiation | 1/36 | 166/18866 | 0.272731044 | 0.291617906 | 0.141417023 | BAX | 1 |
| GO:0038095 | Fc-epsilon receptor signaling pathway | 1/36 | 169/18866 | 0.276923457 | 0.295842945 | 0.143465911 | JUN | 1 |
| GO:0055002 | striated muscle cell development | 1/36 | 169/18866 | 0.276923457 | 0.295842945 | 0.143465911 | CCNB1 | 1 |
| GO:0000910 | cytokinesis | 1/36 | 172/18866 | 0.28109237 | 0.299774877 | 0.14537266 | PRKCE | 1 |
| GO:0006022 | aminoglycan metabolic process | 1/36 | 172/18866 | 0.28109237 | 0.299774877 | 0.14537266 | IL1B | 1 |
| GO:0015698 | inorganic anion transport | 1/36 | 172/18866 | 0.28109237 | 0.299774877 | 0.14537266 | GABRA1 | 1 |
| GO:1990138 | neuron projection extension | 1/36 | 172/18866 | 0.28109237 | 0.299774877 | 0.14537266 | MAP2 | 1 |
| GO:0009152 | purine ribonucleotide biosynthetic process | 1/36 | 173/18866 | 0.282476807 | 0.300989827 | 0.145961837 | FASN | 1 |
| GO:0050954 | sensory perception of mechanical stimulus | 1/36 | 173/18866 | 0.282476807 | 0.300989827 | 0.145961837 | CASP3 | 1 |
| GO:0051099 | positive regulation of binding | 1/36 | 174/18866 | 0.283858652 | 0.30219991 | 0.146548654 | PON1 | 1 |
| GO:0097696 | receptor signaling pathway via STAT | 1/36 | 174/18866 | 0.283858652 | 0.30219991 | 0.146548654 | TNF | 1 |
| GO:0010921 | regulation of phosphatase activity | 1/36 | 175/18866 | 0.28523791 | 0.303536658 | 0.147196896 | TNF | 1 |
| GO:0140013 | meiotic nuclear division | 1/36 | 177/18866 | 0.287988683 | 0.306331116 | 0.148552039 | PDE3A | 1 |
| GO:0006956 | complement activation | 1/36 | 178/18866 | 0.289360206 | 0.307523509 | 0.149130277 | IL1B | 1 |
| GO:0019083 | viral transcription | 1/36 | 178/18866 | 0.289360206 | 0.307523509 | 0.149130277 | JUN | 1 |
| GO:0008361 | regulation of cell size | 1/36 | 181/18866 | 0.293459384 | 0.311340877 | 0.150981469 | MAP2 | 1 |
| GO:0045619 | regulation of lymphocyte differentiation | 1/36 | 181/18866 | 0.293459384 | 0.311340877 | 0.150981469 | IL1B | 1 |
| GO:0048736 | appendage development | 1/36 | 181/18866 | 0.293459384 | 0.311340877 | 0.150981469 | BAX | 1 |
| GO:0060173 | limb development | 1/36 | 181/18866 | 0.293459384 | 0.311340877 | 0.150981469 | BAX | 1 |
| GO:0090288 | negative regulation of cellular response to growth factor stimulus | 1/36 | 182/18866 | 0.294820661 | 0.312649989 | 0.151616309 | TP53 | 1 |
| GO:0001959 | regulation of cytokine-mediated signaling pathway | 1/36 | 183/18866 | 0.296179388 | 0.313144013 | 0.151855881 | TNF | 1 |
| GO:0006986 | response to unfolded protein | 1/36 | 183/18866 | 0.296179388 | 0.313144013 | 0.151855881 | BAX | 1 |
| GO:0007051 | spindle organization | 1/36 | 183/18866 | 0.296179388 | 0.313144013 | 0.151855881 | CCNB1 | 1 |
| GO:0043433 | negative regulation of DNA-binding transcription factor activity | 1/36 | 183/18866 | 0.296179388 | 0.313144013 | 0.151855881 | ESR1 | 1 |
| GO:0055001 | muscle cell development | 1/36 | 183/18866 | 0.296179388 | 0.313144013 | 0.151855881 | CCNB1 | 1 |
| GO:0071347 | cellular response to interleukin-1 | 1/36 | 183/18866 | 0.296179388 | 0.313144013 | 0.151855881 | IL1B | 1 |
| GO:0120032 | regulation of plasma membrane bounded cell projection assembly | 1/36 | 183/18866 | 0.296179388 | 0.313144013 | 0.151855881 | PRKCD | 1 |
| GO:0002695 | negative regulation of leukocyte activation | 1/36 | 184/18866 | 0.29753557 | 0.314171968 | 0.152354377 | CASP3 | 1 |
| GO:0007416 | synapse assembly | 1/36 | 184/18866 | 0.29753557 | 0.314171968 | 0.152354377 | GABRA1 | 1 |
| GO:0022900 | electron transport chain | 1/36 | 184/18866 | 0.29753557 | 0.314171968 | 0.152354377 | CCNB1 | 1 |
| GO:0060491 | regulation of cell projection assembly | 1/36 | 185/18866 | 0.298889211 | 0.315330044 | 0.152915974 | PRKCD | 1 |
| GO:2001257 | regulation of cation channel activity | 1/36 | 185/18866 | 0.298889211 | 0.315330044 | 0.152915974 | OPRM1 | 1 |
| GO:0009260 | ribonucleotide biosynthetic process | 1/36 | 186/18866 | 0.300240316 | 0.316483459 | 0.15347531 | FASN | 1 |
| GO:0050770 | regulation of axonogenesis | 1/36 | 186/18866 | 0.300240316 | 0.316483459 | 0.15347531 | MAP2 | 1 |
| GO:0002285 | lymphocyte activation involved in immune response | 1/36 | 187/18866 | 0.301588889 | 0.317359933 | 0.153900347 | TP53 | 1 |
| GO:0008217 | regulation of blood pressure | 1/36 | 187/18866 | 0.301588889 | 0.317359933 | 0.153900347 | AR | 1 |
| GO:0045333 | cellular respiration | 1/36 | 187/18866 | 0.301588889 | 0.317359933 | 0.153900347 | CCNB1 | 1 |
| GO:0048639 | positive regulation of developmental growth | 1/36 | 187/18866 | 0.301588889 | 0.317359933 | 0.153900347 | CCNB1 | 1 |
| GO:0007369 | gastrulation | 1/36 | 189/18866 | 0.304278459 | 0.320052964 | 0.155206304 | TP53 | 1 |
| GO:0099111 | microtubule-based transport | 1/36 | 190/18866 | 0.305619466 | 0.321325819 | 0.155823562 | MAP2 | 1 |
| GO:0016525 | negative regulation of angiogenesis | 1/36 | 192/18866 | 0.308293944 | 0.323721827 | 0.15698548 | TNF | 1 |
| GO:0021953 | central nervous system neuron differentiation | 1/36 | 192/18866 | 0.308293944 | 0.323721827 | 0.15698548 | MAP2 | 1 |
| GO:0044272 | sulfur compound biosynthetic process | 1/36 | 192/18866 | 0.308293944 | 0.323721827 | 0.15698548 | FASN | 1 |
| GO:1903046 | meiotic cell cycle process | 1/36 | 193/18866 | 0.309627424 | 0.324983038 | 0.157597091 | PDE3A | 1 |
| GO:0046390 | ribose phosphate biosynthetic process | 1/36 | 194/18866 | 0.310958406 | 0.325961949 | 0.158071804 | FASN | 1 |
| GO:1902115 | regulation of organelle assembly | 1/36 | 194/18866 | 0.310958406 | 0.325961949 | 0.158071804 | TNF | 1 |
| GO:2000181 | negative regulation of blood vessel morphogenesis | 1/36 | 194/18866 | 0.310958406 | 0.325961949 | 0.158071804 | TNF | 1 |
| GO:0007219 | Notch signaling pathway | 1/36 | 195/18866 | 0.312286892 | 0.326796385 | 0.158476455 | MYC | 1 |
| GO:0019080 | viral gene expression | 1/36 | 195/18866 | 0.312286892 | 0.326796385 | 0.158476455 | JUN | 1 |
| GO:0031497 | chromatin assembly | 1/36 | 195/18866 | 0.312286892 | 0.326796385 | 0.158476455 | TP53 | 1 |
| GO:1901136 | carbohydrate derivative catabolic process | 1/36 | 195/18866 | 0.312286892 | 0.326796385 | 0.158476455 | PRKCD | 1 |
| GO:0000819 | sister chromatid segregation | 1/36 | 196/18866 | 0.313612888 | 0.327625379 | 0.158878466 | CCNB1 | 1 |
| GO:0002040 | sprouting angiogenesis | 1/36 | 196/18866 | 0.313612888 | 0.327625379 | 0.158878466 | KDR | 1 |
| GO:0050728 | negative regulation of inflammatory response | 1/36 | 196/18866 | 0.313612888 | 0.327625379 | 0.158878466 | PRKCD | 1 |
| GO:0060759 | regulation of response to cytokine stimulus | 1/36 | 196/18866 | 0.313612888 | 0.327625379 | 0.158878466 | TNF | 1 |
| GO:0006164 | purine nucleotide biosynthetic process | 1/36 | 197/18866 | 0.314936398 | 0.328868081 | 0.159481102 | FASN | 1 |
| GO:0043112 | receptor metabolic process | 1/36 | 198/18866 | 0.316257427 | 0.330107136 | 0.160081968 | TNF | 1 |
| GO:0030705 | cytoskeleton-dependent intracellular transport | 1/36 | 200/18866 | 0.318892059 | 0.332433123 | 0.161209931 | MAP2 | 1 |
| GO:0050657 | nucleic acid transport | 1/36 | 200/18866 | 0.318892059 | 0.332433123 | 0.161209931 | EIF6 | 1 |
| GO:0050658 | RNA transport | 1/36 | 200/18866 | 0.318892059 | 0.332433123 | 0.161209931 | EIF6 | 1 |
| GO:0051236 | establishment of RNA localization | 1/36 | 203/18866 | 0.322825511 | 0.336390759 | 0.163129145 | EIF6 | 1 |
| GO:0060348 | bone development | 1/36 | 204/18866 | 0.324131747 | 0.337608586 | 0.163719718 | TP53 | 1 |
| GO:0035966 | response to topologically incorrect protein | 1/36 | 205/18866 | 0.325435533 | 0.33882283 | 0.164308552 | BAX | 1 |
| GO:0006888 | endoplasmic reticulum to Golgi vesicle-mediated transport | 1/36 | 206/18866 | 0.326736874 | 0.340033499 | 0.164895653 | F7 | 1 |
| GO:1901343 | negative regulation of vasculature development | 1/36 | 207/18866 | 0.328035774 | 0.341240603 | 0.165481026 | TNF | 1 |
| GO:0072522 | purine-containing compound biosynthetic process | 1/36 | 208/18866 | 0.329332237 | 0.342299171 | 0.165994367 | FASN | 1 |
| GO:1902905 | positive regulation of supramolecular fiber organization | 1/36 | 208/18866 | 0.329332237 | 0.342299171 | 0.165994367 | PRKCE | 1 |
| GO:0040029 | regulation of gene expression, epigenetic | 1/36 | 209/18866 | 0.330626269 | 0.343498726 | 0.166576078 | CHEK1 | 1 |
| GO:0002377 | immunoglobulin production | 1/36 | 210/18866 | 0.331917872 | 0.344694745 | 0.167156075 | TNF | 1 |
| GO:0031396 | regulation of protein ubiquitination | 1/36 | 211/18866 | 0.333207053 | 0.345887237 | 0.167734362 | PRKCE | 1 |
| GO:0030278 | regulation of ossification | 1/36 | 212/18866 | 0.334493815 | 0.34692958 | 0.168239835 | TNF | 1 |
| GO:0051650 | establishment of vesicle localization | 1/36 | 212/18866 | 0.334493815 | 0.34692958 | 0.168239835 | MAP2 | 1 |
| GO:0045089 | positive regulation of innate immune response | 1/36 | 213/18866 | 0.335778163 | 0.348114607 | 0.168814501 | PRKCD | 1 |
| GO:0050870 | positive regulation of T cell activation | 1/36 | 214/18866 | 0.337060101 | 0.349296137 | 0.169387472 | IL1B | 1 |
| GO:0071805 | potassium ion transmembrane transport | 1/36 | 218/18866 | 0.342163841 | 0.35443554 | 0.171879771 | KCNH2 | 1 |
| GO:0009952 | anterior/posterior pattern specification | 1/36 | 219/18866 | 0.343433795 | 0.355600998 | 0.172444947 | TP53 | 1 |
| GO:0006333 | chromatin assembly or disassembly | 1/36 | 220/18866 | 0.344701365 | 0.356462448 | 0.172862698 | TP53 | 1 |
| GO:0007163 | establishment or maintenance of cell polarity | 1/36 | 220/18866 | 0.344701365 | 0.356462448 | 0.172862698 | MAP2 | 1 |
| GO:0017148 | negative regulation of translation | 1/36 | 220/18866 | 0.344701365 | 0.356462448 | 0.172862698 | EIF6 | 1 |
| GO:0042445 | hormone metabolic process | 1/36 | 221/18866 | 0.345966556 | 0.357620166 | 0.173424122 | ESR1 | 1 |
| GO:0097529 | myeloid leukocyte migration | 1/36 | 222/18866 | 0.347229372 | 0.358774456 | 0.173983883 | IL1B | 1 |
| GO:0008037 | cell recognition | 1/36 | 226/18866 | 0.352256972 | 0.363816099 | 0.176428774 | CASP3 | 1 |
| GO:0006364 | rRNA processing | 1/36 | 227/18866 | 0.353507978 | 0.36495462 | 0.176980888 | EIF6 | 1 |
| GO:0006836 | neurotransmitter transport | 1/36 | 229/18866 | 0.356002947 | 0.366913197 | 0.177930679 | SLC6A4 | 1 |
| GO:0048762 | mesenchymal cell differentiation | 1/36 | 229/18866 | 0.356002947 | 0.366913197 | 0.177930679 | IL1B | 1 |
| GO:0050807 | regulation of synapse organization | 1/36 | 229/18866 | 0.356002947 | 0.366913197 | 0.177930679 | TNF | 1 |
| GO:0051648 | vesicle localization | 1/36 | 229/18866 | 0.356002947 | 0.366913197 | 0.177930679 | MAP2 | 1 |
| GO:0007160 | cell-matrix adhesion | 1/36 | 230/18866 | 0.357246918 | 0.367886403 | 0.178402625 | KDR | 1 |
| GO:0051495 | positive regulation of cytoskeleton organization | 1/36 | 230/18866 | 0.357246918 | 0.367886403 | 0.178402625 | PRKCE | 1 |
| GO:0048738 | cardiac muscle tissue development | 1/36 | 231/18866 | 0.358488553 | 0.368855574 | 0.178872614 | CCNB1 | 1 |
| GO:0051259 | protein complex oligomerization | 1/36 | 231/18866 | 0.358488553 | 0.368855574 | 0.178872614 | TP53 | 1 |
| GO:0009266 | response to temperature stimulus | 1/36 | 233/18866 | 0.360964832 | 0.371092404 | 0.179957341 | CDKN1A | 1 |
| GO:0022618 | ribonucleoprotein complex assembly | 1/36 | 233/18866 | 0.360964832 | 0.371092404 | 0.179957341 | EIF6 | 1 |
| GO:0001505 | regulation of neurotransmitter levels | 1/36 | 235/18866 | 0.363431816 | 0.373315945 | 0.181035624 | SLC6A4 | 1 |
| GO:0045444 | fat cell differentiation | 1/36 | 235/18866 | 0.363431816 | 0.373315945 | 0.181035624 | TNF | 1 |
| GO:0006403 | RNA localization | 1/36 | 236/18866 | 0.364661833 | 0.374266221 | 0.18149645 | EIF6 | 1 |
| GO:0044242 | cellular lipid catabolic process | 1/36 | 236/18866 | 0.364661833 | 0.374266221 | 0.18149645 | PRKCD | 1 |
| GO:0048588 | developmental cell growth | 1/36 | 237/18866 | 0.36588954 | 0.375369336 | 0.182031394 | MAP2 | 1 |
| GO:0016072 | rRNA metabolic process | 1/36 | 238/18866 | 0.36711494 | 0.376311974 | 0.182488516 | EIF6 | 1 |
| GO:0034249 | negative regulation of cellular amide metabolic process | 1/36 | 238/18866 | 0.36711494 | 0.376311974 | 0.182488516 | EIF6 | 1 |
| GO:0050803 | regulation of synapse structure or activity | 1/36 | 240/18866 | 0.369558837 | 0.37850102 | 0.183550071 | TNF | 1 |
| GO:0071826 | ribonucleoprotein complex subunit organization | 1/36 | 240/18866 | 0.369558837 | 0.37850102 | 0.183550071 | EIF6 | 1 |
| GO:0006813 | potassium ion transport | 1/36 | 242/18866 | 0.371993558 | 0.380677026 | 0.184605302 | KCNH2 | 1 |
| GO:1903320 | regulation of protein modification by small protein conjugation or removal | 1/36 | 242/18866 | 0.371993558 | 0.380677026 | 0.184605302 | PRKCE | 1 |
| GO:0042593 | glucose homeostasis | 1/36 | 245/18866 | 0.375628508 | 0.384236661 | 0.18633151 | PRKCE | 1 |
| GO:0033500 | carbohydrate homeostasis | 1/36 | 246/18866 | 0.376835606 | 0.385310876 | 0.186852439 | PRKCE | 1 |
| GO:0016358 | dendrite development | 1/36 | 247/18866 | 0.378040435 | 0.386381877 | 0.187371809 | MAP2 | 1 |
| GO:0051321 | meiotic cell cycle | 1/36 | 254/18866 | 0.386411054 | 0.394608627 | 0.191361285 | PDE3A | 1 |
| GO:0090092 | regulation of transmembrane receptor protein serine/threonine kinase signaling pathway | 1/36 | 254/18866 | 0.386411054 | 0.394608627 | 0.191361285 | TP53 | 1 |
| GO:0010951 | negative regulation of endopeptidase activity | 1/36 | 258/18866 | 0.391144982 | 0.399110944 | 0.193544636 | TNF | 1 |
| GO:0051607 | defense response to virus | 1/36 | 258/18866 | 0.391144982 | 0.399110944 | 0.193544636 | IL1B | 1 |
| GO:0015931 | nucleobase-containing compound transport | 1/36 | 261/18866 | 0.394672106 | 0.402542593 | 0.195208778 | EIF6 | 1 |
| GO:0031330 | negative regulation of cellular catabolic process | 1/36 | 265/18866 | 0.399344051 | 0.407138557 | 0.197437542 | TP53 | 1 |
| GO:0009165 | nucleotide biosynthetic process | 1/36 | 266/18866 | 0.400506549 | 0.408154245 | 0.19793009 | FASN | 1 |
| GO:0031348 | negative regulation of defense response | 1/36 | 269/18866 | 0.403980932 | 0.411182914 | 0.19939881 | PRKCD | 1 |
| GO:0090596 | sensory organ morphogenesis | 1/36 | 269/18866 | 0.403980932 | 0.411182914 | 0.19939881 | BAX | 1 |
| GO:1901293 | nucleoside phosphate biosynthetic process | 1/36 | 269/18866 | 0.403980932 | 0.411182914 | 0.19939881 | FASN | 1 |
| GO:0010466 | negative regulation of peptidase activity | 1/36 | 272/18866 | 0.407435735 | 0.414355729 | 0.200937434 | TNF | 1 |
| GO:0098813 | nuclear chromosome segregation | 1/36 | 272/18866 | 0.407435735 | 0.414355729 | 0.200937434 | CCNB1 | 1 |
| GO:0045927 | positive regulation of growth | 1/36 | 274/18866 | 0.409728113 | 0.4165145 | 0.201984307 | CCNB1 | 1 |
| GO:0016054 | organic acid catabolic process | 1/36 | 282/18866 | 0.41881171 | 0.425396255 | 0.20629142 | PON1 | 1 |
| GO:0046395 | carboxylic acid catabolic process | 1/36 | 282/18866 | 0.41881171 | 0.425396255 | 0.20629142 | PON1 | 1 |
| GO:0007411 | axon guidance | 1/36 | 284/18866 | 0.421061288 | 0.427504326 | 0.207313707 | PRKCA | 1 |
| GO:0097485 | neuron projection guidance | 1/36 | 285/18866 | 0.4221829 | 0.428465903 | 0.207780013 | PRKCA | 1 |
| GO:0031503 | protein-containing complex localization | 1/36 | 291/18866 | 0.42886834 | 0.435070981 | 0.210983076 | EIF6 | 1 |
| GO:0006310 | DNA recombination | 1/36 | 299/18866 | 0.437665382 | 0.44381186 | 0.215221873 | CHEK1 | 1 |
| GO:0030216 | keratinocyte differentiation | 1/36 | 306/18866 | 0.445254617 | 0.451321257 | 0.218863476 | CASP3 | 1 |
| GO:0045088 | regulation of innate immune response | 1/36 | 307/18866 | 0.446330632 | 0.452225217 | 0.219301842 | PRKCD | 1 |
| GO:0010769 | regulation of cell morphogenesis involved in differentiation | 1/36 | 310/18866 | 0.449546516 | 0.455107916 | 0.220699776 | MAP2 | 1 |
| GO:0042254 | ribosome biogenesis | 1/36 | 310/18866 | 0.449546516 | 0.455107916 | 0.220699776 | EIF6 | 1 |
| GO:0098656 | anion transmembrane transport | 1/36 | 315/18866 | 0.454866009 | 0.460303401 | 0.223219272 | GABRA1 | 1 |
| GO:0043414 | macromolecule methylation | 1/36 | 318/18866 | 0.458033679 | 0.463317958 | 0.224681149 | MYC | 1 |
| GO:0050851 | antigen receptor-mediated signaling pathway | 1/36 | 325/18866 | 0.465355476 | 0.470530351 | 0.228178723 | BAX | 1 |
| GO:0007059 | chromosome segregation | 1/36 | 334/18866 | 0.474628026 | 0.479708441 | 0.232629541 | CCNB1 | 1 |
| GO:0019058 | viral life cycle | 1/36 | 341/18866 | 0.48173173 | 0.486687818 | 0.236014116 | TNF | 1 |
| GO:0006650 | glycerophospholipid metabolic process | 1/36 | 343/18866 | 0.48374415 | 0.488519904 | 0.236902567 | PON1 | 1 |
| GO:0030336 | negative regulation of cell migration | 1/36 | 350/18866 | 0.490727965 | 0.495368896 | 0.240223913 | TNF | 1 |
| GO:0001818 | negative regulation of cytokine production | 1/36 | 360/18866 | 0.500545742 | 0.505071844 | 0.244929255 | TNF | 1 |
| GO:0009913 | epidermal cell differentiation | 1/36 | 365/18866 | 0.505385379 | 0.509536388 | 0.247094289 | CASP3 | 1 |
| GO:2000146 | negative regulation of cell motility | 1/36 | 365/18866 | 0.505385379 | 0.509536388 | 0.247094289 | TNF | 1 |
| GO:0007018 | microtubule-based movement | 1/36 | 368/18866 | 0.508267244 | 0.512231561 | 0.248401284 | MAP2 | 1 |
| GO:0006790 | sulfur compound metabolic process | 1/36 | 371/18866 | 0.511132781 | 0.514696873 | 0.249596812 | FASN | 1 |
| GO:0032259 | methylation | 1/36 | 371/18866 | 0.511132781 | 0.514696873 | 0.249596812 | MYC | 1 |
| GO:0048193 | Golgi vesicle transport | 1/36 | 374/18866 | 0.513982081 | 0.517353837 | 0.250885278 | F7 | 1 |
| GO:0030111 | regulation of Wnt signaling pathway | 1/36 | 375/18866 | 0.514928255 | 0.518093797 | 0.251244114 | ESR1 | 1 |
| GO:0014706 | striated muscle tissue development | 1/36 | 389/18866 | 0.52798813 | 0.531016329 | 0.257510759 | CCNB1 | 1 |
| GO:0040013 | negative regulation of locomotion | 1/36 | 397/18866 | 0.535296753 | 0.538146408 | 0.260968416 | TNF | 1 |
| GO:0034470 | ncRNA processing | 1/36 | 400/18866 | 0.538009021 | 0.540430502 | 0.262076063 | EIF6 | 1 |
| GO:0051271 | negative regulation of cellular component movement | 1/36 | 400/18866 | 0.538009021 | 0.540430502 | 0.262076063 | TNF | 1 |
| GO:0043547 | positive regulation of GTPase activity | 1/36 | 406/18866 | 0.543387465 | 0.545609909 | 0.264587761 | JUN | 1 |
| GO:0007517 | muscle organ development | 1/36 | 407/18866 | 0.544277934 | 0.546280592 | 0.264913002 | CCNB1 | 1 |
| GO:0060537 | muscle tissue development | 1/36 | 409/18866 | 0.546053809 | 0.547839028 | 0.265668749 | CCNB1 | 1 |
| GO:0043161 | proteasome-mediated ubiquitin-dependent protein catabolic process | 1/36 | 424/18866 | 0.559160094 | 0.560759 | 0.271934153 | CCNB1 | 1 |
| GO:0046486 | glycerolipid metabolic process | 1/36 | 434/18866 | 0.567692443 | 0.569083278 | 0.275970924 | PON1 | 1 |
| GO:0043087 | regulation of GTPase activity | 1/36 | 481/18866 | 0.605690683 | 0.606926786 | 0.294322733 | JUN | 1 |
| GO:0022613 | ribonucleoprotein complex biogenesis | 1/36 | 482/18866 | 0.606462787 | 0.607452526 | 0.294577686 | EIF6 | 1 |
| GO:0010498 | proteasomal protein catabolic process | 1/36 | 483/18866 | 0.607233421 | 0.607976366 | 0.294831716 | CCNB1 | 1 |
| GO:0043312 | neutrophil degranulation | 1/36 | 487/18866 | 0.610301313 | 0.610798909 | 0.296200478 | PRKCD | 1 |
| GO:0002283 | neutrophil activation involved in immune response | 1/36 | 490/18866 | 0.61258693 | 0.612836558 | 0.297188615 | PRKCD | 1 |
| GO:0034660 | ncRNA metabolic process | 1/36 | 493/18866 | 0.614859512 | 0.614859512 | 0.298169625 | EIF6 | 1 |

**Table S4.** Results of CC GO terms enrichment of XBCQD in monkeypox virus.

| **ID** | **Description** | **GeneRatio** | **BgRatio** | **pvalue** | **p.adjust** | **qvalue** | **geneID** | **Count** |
| --- | --- | --- | --- | --- | --- | --- | --- | --- |
| GO:0034399 | nuclear periphery | 5/36 | 131/19559 | 3.98358E-06 | 0.000617454 | 0.000419324 | TP53/PRKCD/PCNA/EIF6/MAP2 | 5 |
| GO:0045211 | postsynaptic membrane | 6/36 | 280/19559 | 1.10758E-05 | 0.000858377 | 0.000582938 | SLC6A4/OPRM1/GABRA1/CHRNA2/CHRM4/CHRM3 | 6 |
| GO:0045121 | membrane raft | 6/36 | 329/19559 | 2.75407E-05 | 0.001078201 | 0.000732225 | SCN5A/KDR/TNF/CASP3/SLC6A4/OPRM1 | 6 |
| GO:0098857 | membrane microdomain | 6/36 | 330/19559 | 2.80136E-05 | 0.001078201 | 0.000732225 | SCN5A/KDR/TNF/CASP3/SLC6A4/OPRM1 | 6 |
| GO:0098589 | membrane region | 6/36 | 343/19559 | 3.47807E-05 | 0.001078201 | 0.000732225 | SCN5A/KDR/TNF/CASP3/SLC6A4/OPRM1 | 6 |
| GO:0099055 | integral component of postsynaptic membrane | 4/36 | 117/19559 | 6.1788E-05 | 0.001252506 | 0.000850598 | SLC6A4/OPRM1/GABRA1/CHRM3 | 4 |
| GO:0000307 | cyclin-dependent protein kinase holoenzyme complex | 3/36 | 43/19559 | 6.71809E-05 | 0.001252506 | 0.000850598 | PCNA/CDKN1A/CCNB1 | 3 |
| GO:0097060 | synaptic membrane | 6/36 | 387/19559 | 6.80352E-05 | 0.001252506 | 0.000850598 | SLC6A4/OPRM1/GABRA1/CHRNA2/CHRM4/CHRM3 | 6 |
| GO:0098936 | intrinsic component of postsynaptic membrane | 4/36 | 122/19559 | 7.27261E-05 | 0.001252506 | 0.000850598 | SLC6A4/OPRM1/GABRA1/CHRM3 | 4 |
| GO:0099699 | integral component of synaptic membrane | 4/36 | 154/19559 | 0.000178928 | 0.002526215 | 0.001715596 | SLC6A4/OPRM1/GABRA1/CHRM3 | 4 |
| GO:0005652 | nuclear lamina | 2/36 | 11/19559 | 0.00017928 | 0.002526215 | 0.001715596 | PCNA/EIF6 | 2 |
| GO:0099240 | intrinsic component of synaptic membrane | 4/36 | 166/19559 | 0.000238482 | 0.003080395 | 0.002091949 | SLC6A4/OPRM1/GABRA1/CHRM3 | 4 |
| GO:0099056 | integral component of presynaptic membrane | 3/36 | 74/19559 | 0.000339331 | 0.004045869 | 0.002747619 | SLC6A4/OPRM1/CHRM3 | 3 |
| GO:0098889 | intrinsic component of presynaptic membrane | 3/36 | 83/19559 | 0.000475532 | 0.005264824 | 0.003575432 | SLC6A4/OPRM1/CHRM3 | 3 |
| GO:1902554 | serine/threonine protein kinase complex | 3/36 | 89/19559 | 0.000583321 | 0.006027649 | 0.00409348 | PCNA/CDKN1A/CCNB1 | 3 |
| GO:1902911 | protein kinase complex | 3/36 | 104/19559 | 0.000917849 | 0.008891663 | 0.006038481 | PCNA/CDKN1A/CCNB1 | 3 |
| GO:0061695 | transferase complex, transferring phosphorus-containing groups | 4/36 | 253/19559 | 0.001163105 | 0.01060478 | 0.007201888 | TP53/PCNA/CDKN1A/CCNB1 | 4 |
| GO:0032839 | dendrite cytoplasm | 2/36 | 34/19559 | 0.001780626 | 0.015333165 | 0.010413015 | OPRM1/MAP2 | 2 |
| GO:0034702 | ion channel complex | 4/36 | 299/19559 | 0.00214466 | 0.017495912 | 0.011881774 | SCN5A/KCNH2/GABRA1/CHRNA2 | 4 |
| GO:1902495 | transmembrane transporter complex | 4/36 | 322/19559 | 0.002803513 | 0.02063518 | 0.014013705 | SCN5A/KCNH2/GABRA1/CHRNA2 | 4 |
| GO:0032590 | dendrite membrane | 2/36 | 43/19559 | 0.002836497 | 0.02063518 | 0.014013705 | OPRM1/GABRA1 | 2 |
| GO:0042734 | presynaptic membrane | 3/36 | 156/19559 | 0.002929845 | 0.02063518 | 0.014013705 | SLC6A4/OPRM1/CHRM3 | 3 |
| GO:1990351 | transporter complex | 4/36 | 330/19559 | 0.003061994 | 0.02063518 | 0.014013705 | SCN5A/KCNH2/GABRA1/CHRNA2 | 4 |
| GO:0032589 | neuron projection membrane | 2/36 | 61/19559 | 0.005630263 | 0.036362112 | 0.024694134 | OPRM1/GABRA1 | 2 |
| GO:0005657 | replication fork | 2/36 | 71/19559 | 0.007557919 | 0.0468591 | 0.031822818 | TP53/PCNA | 2 |
| GO:0099060 | integral component of postsynaptic specialization membrane | 2/36 | 74/19559 | 0.008186552 | 0.048804444 | 0.033143935 | GABRA1/CHRM3 | 2 |
| GO:0098948 | intrinsic component of postsynaptic specialization membrane | 2/36 | 77/19559 | 0.008837956 | 0.050736415 | 0.034455969 | GABRA1/CHRM3 | 2 |
| GO:0120111 | neuron projection cytoplasm | 2/36 | 87/19559 | 0.011170496 | 0.061836675 | 0.041994346 | OPRM1/MAP2 | 2 |
| GO:0000794 | condensed nuclear chromosome | 2/36 | 103/19559 | 0.015399813 | 0.082309343 | 0.055897686 | CHEK1/CCNB1 | 2 |
| GO:0016363 | nuclear matrix | 2/36 | 109/19559 | 0.017137235 | 0.088542381 | 0.060130649 | TP53/PRKCD | 2 |
| GO:0099634 | postsynaptic specialization membrane | 2/36 | 118/19559 | 0.019892112 | 0.099460561 | 0.067545372 | GABRA1/CHRM3 | 2 |
| GO:0044292 | dendrite terminus | 1/36 | 13/19559 | 0.023672317 | 0.114662784 | 0.077869463 | MAP2 | 1 |
| GO:0000940 | condensed chromosome outer kinetochore | 1/36 | 14/19559 | 0.025470526 | 0.116198703 | 0.078912532 | CCNB1 | 1 |
| GO:0042383 | sarcolemma | 2/36 | 135/19559 | 0.025562704 | 0.116198703 | 0.078912532 | SCN5A/OPRM1 | 2 |
| GO:0098687 | chromosomal region | 3/36 | 350/19559 | 0.026238417 | 0.116198703 | 0.078912532 | CHEK1/PCNA/CCNB1 | 3 |
| GO:0099572 | postsynaptic specialization | 3/36 | 361/19559 | 0.02840708 | 0.116612379 | 0.079193466 | MAP2/GABRA1/CHRM3 | 3 |
| GO:0005892 | acetylcholine-gated channel complex | 1/36 | 16/19559 | 0.02905729 | 0.116612379 | 0.079193466 | CHRNA2 | 1 |
| GO:0072562 | blood microparticle | 2/36 | 148/19559 | 0.030290099 | 0.116612379 | 0.079193466 | PRSS1/PON1 | 2 |
| GO:0000778 | condensed nuclear chromosome kinetochore | 1/36 | 17/19559 | 0.030845855 | 0.116612379 | 0.079193466 | CCNB1 | 1 |
| GO:0001518 | voltage-gated sodium channel complex | 1/36 | 17/19559 | 0.030845855 | 0.116612379 | 0.079193466 | SCN5A | 1 |
| GO:0043194 | axon initial segment | 1/36 | 17/19559 | 0.030845855 | 0.116612379 | 0.079193466 | MAP2 | 1 |
| GO:0097440 | apical dendrite | 1/36 | 18/19559 | 0.032631217 | 0.120424731 | 0.0817825 | MAP2 | 1 |
| GO:1902711 | GABA-A receptor complex | 1/36 | 19/19559 | 0.034413382 | 0.123141987 | 0.083627835 | GABRA1 | 1 |
| GO:0090575 | RNA polymerase II transcription regulator complex | 2/36 | 161/19559 | 0.035337084 | 0.123141987 | 0.083627835 | JUN/TP53 | 2 |
| GO:1902710 | GABA receptor complex | 1/36 | 20/19559 | 0.036192354 | 0.123141987 | 0.083627835 | GABRA1 | 1 |
| GO:0000781 | chromosome, telomeric region | 2/36 | 164/19559 | 0.036545364 | 0.123141987 | 0.083627835 | CHEK1/PCNA | 2 |
| GO:0031256 | leading edge membrane | 2/36 | 175/19559 | 0.041110357 | 0.131307691 | 0.089173304 | OPRM1/GABRA1 | 2 |
| GO:0030687 | preribosome, large subunit precursor | 1/36 | 23/19559 | 0.041510173 | 0.131307691 | 0.089173304 | EIF6 | 1 |
| GO:0046930 | pore complex | 1/36 | 23/19559 | 0.041510173 | 0.131307691 | 0.089173304 | BAX | 1 |
| GO:0035327 | transcriptionally active chromatin | 1/36 | 24/19559 | 0.043276432 | 0.13415694 | 0.091108278 | ESR1 | 1 |
| GO:0030894 | replisome | 1/36 | 26/19559 | 0.046799462 | 0.1368608 | 0.092944516 | PCNA | 1 |
| GO:0034706 | sodium channel complex | 1/36 | 26/19559 | 0.046799462 | 0.1368608 | 0.092944516 | SCN5A | 1 |
| GO:0001891 | phagocytic cup | 1/36 | 27/19559 | 0.048556244 | 0.1368608 | 0.092944516 | TNF | 1 |
| GO:0034364 | high-density lipoprotein particle | 1/36 | 27/19559 | 0.048556244 | 0.1368608 | 0.092944516 | PON1 | 1 |
| GO:0005741 | mitochondrial outer membrane | 2/36 | 192/19559 | 0.04856351 | 0.1368608 | 0.092944516 | BAX/PGR | 2 |
| GO:0032993 | protein-DNA complex | 2/36 | 205/19559 | 0.054570296 | 0.150834432 | 0.102434249 | ESR1/PCNA | 2 |
| GO:0000780 | condensed nuclear chromosome, centromeric region | 1/36 | 32/19559 | 0.057293042 | 0.150834432 | 0.102434249 | CCNB1 | 1 |
| GO:0032838 | plasma membrane bounded cell projection cytoplasm | 2/36 | 211/19559 | 0.057427791 | 0.150834432 | 0.102434249 | OPRM1/MAP2 | 2 |
| GO:0005669 | transcription factor TFIID complex | 1/36 | 34/19559 | 0.060765878 | 0.150834432 | 0.102434249 | TP53 | 1 |
| GO:0005719 | nuclear euchromatin | 1/36 | 34/19559 | 0.060765878 | 0.150834432 | 0.102434249 | JUN | 1 |
| GO:0031968 | organelle outer membrane | 2/36 | 218/19559 | 0.060826859 | 0.150834432 | 0.102434249 | BAX/PGR | 2 |
| GO:0019867 | outer membrane | 2/36 | 220/19559 | 0.061810648 | 0.150834432 | 0.102434249 | BAX/PGR | 2 |
| GO:0034703 | cation channel complex | 2/36 | 221/19559 | 0.062304615 | 0.150834432 | 0.102434249 | SCN5A/KCNH2 | 2 |
| GO:0000793 | condensed chromosome | 2/36 | 222/19559 | 0.062799955 | 0.150834432 | 0.102434249 | CHEK1/CCNB1 | 2 |
| GO:0034358 | plasma lipoprotein particle | 1/36 | 36/19559 | 0.064226274 | 0.150834432 | 0.102434249 | PON1 | 1 |
| GO:1990777 | lipoprotein particle | 1/36 | 36/19559 | 0.064226274 | 0.150834432 | 0.102434249 | PON1 | 1 |
| GO:0043198 | dendritic shaft | 1/36 | 38/19559 | 0.067674275 | 0.156559889 | 0.106322505 | MAP2 | 1 |
| GO:0032994 | protein-lipid complex | 1/36 | 39/19559 | 0.06939364 | 0.158176679 | 0.107420495 | PON1 | 1 |
| GO:0043596 | nuclear replication fork | 1/36 | 41/19559 | 0.072823127 | 0.163588183 | 0.11109554 | PCNA | 1 |
| GO:0000791 | euchromatin | 1/36 | 42/19559 | 0.074533259 | 0.165037931 | 0.112080089 | JUN | 1 |
| GO:0099568 | cytoplasmic region | 2/36 | 254/19559 | 0.079338212 | 0.173203139 | 0.117625222 | OPRM1/MAP2 | 2 |
| GO:0034707 | chloride channel complex | 1/36 | 50/19559 | 0.088104363 | 0.185559977 | 0.126016963 | GABRA1 | 1 |
| GO:0009925 | basal plasma membrane | 1/36 | 51/19559 | 0.089787086 | 0.185559977 | 0.126016963 | CHRM3 | 1 |
| GO:0014704 | intercalated disc | 1/36 | 51/19559 | 0.089787086 | 0.185559977 | 0.126016963 | SCN5A | 1 |
| GO:0099061 | integral component of postsynaptic density membrane | 1/36 | 51/19559 | 0.089787086 | 0.185559977 | 0.126016963 | CHRM3 | 1 |
| GO:0030315 | T-tubule | 1/36 | 54/19559 | 0.094817162 | 0.190865715 | 0.12962018 | SCN5A | 1 |
| GO:0099146 | intrinsic component of postsynaptic density membrane | 1/36 | 54/19559 | 0.094817162 | 0.190865715 | 0.12962018 | CHRM3 | 1 |
| GO:0035861 | site of double-strand break | 1/36 | 61/19559 | 0.106449134 | 0.211533536 | 0.143656052 | TP53 | 1 |
| GO:0150034 | distal axon | 2/36 | 309/19559 | 0.110464694 | 0.216734526 | 0.147188133 | MAP2/CHRM3 | 2 |
| GO:0016328 | lateral plasma membrane | 1/36 | 65/19559 | 0.113030591 | 0.218996771 | 0.148724462 | SCN5A | 1 |
| GO:0044304 | main axon | 1/36 | 68/19559 | 0.117935731 | 0.225679486 | 0.153262809 | MAP2 | 1 |
| GO:0045178 | basal part of cell | 1/36 | 69/19559 | 0.11956491 | 0.226006841 | 0.153485122 | CHRM3 | 1 |
| GO:0014069 | postsynaptic density | 2/36 | 337/19559 | 0.12738112 | 0.231980025 | 0.157541613 | MAP2/CHRM3 | 2 |
| GO:0044291 | cell-cell contact zone | 1/36 | 74/19559 | 0.127667018 | 0.231980025 | 0.157541613 | SCN5A | 1 |
| GO:0098982 | GABA-ergic synapse | 1/36 | 74/19559 | 0.127667018 | 0.231980025 | 0.157541613 | GABRA1 | 1 |
| GO:0017053 | transcription repressor complex | 1/36 | 76/19559 | 0.130887524 | 0.231980025 | 0.157541613 | JUN | 1 |
| GO:0032279 | asymmetric synapse | 2/36 | 343/19559 | 0.131084964 | 0.231980025 | 0.157541613 | MAP2/CHRM3 | 2 |
| GO:0031253 | cell projection membrane | 2/36 | 344/19559 | 0.131704788 | 0.231980025 | 0.157541613 | OPRM1/GABRA1 | 2 |
| GO:0030684 | preribosome | 1/36 | 78/19559 | 0.13409647 | 0.233538796 | 0.158600201 | EIF6 | 1 |
| GO:0016591 | RNA polymerase II, holoenzyme | 1/36 | 79/19559 | 0.13569662 | 0.233699735 | 0.158709497 | TP53 | 1 |
| GO:0005901 | caveola | 1/36 | 82/19559 | 0.140479841 | 0.236677993 | 0.160732083 | SCN5A | 1 |
| GO:0090734 | site of DNA damage | 1/36 | 82/19559 | 0.140479841 | 0.236677993 | 0.160732083 | TP53 | 1 |
| GO:0098984 | neuron to neuron synapse | 2/36 | 368/19559 | 0.146782531 | 0.244637551 | 0.166137556 | MAP2/CHRM3 | 2 |
| GO:0008076 | voltage-gated potassium channel complex | 1/36 | 88/19559 | 0.149969197 | 0.247289634 | 0.167938631 | KCNH2 | 1 |
| GO:0035578 | azurophil granule lumen | 1/36 | 91/19559 | 0.154675601 | 0.249659883 | 0.169548307 | PRKCD | 1 |
| GO:0098839 | postsynaptic density membrane | 1/36 | 92/19559 | 0.156238765 | 0.249659883 | 0.169548307 | CHRM3 | 1 |
| GO:1905368 | peptidase complex | 1/36 | 92/19559 | 0.156238765 | 0.249659883 | 0.169548307 | F7 | 1 |
| GO:0034705 | potassium channel complex | 1/36 | 96/19559 | 0.162463366 | 0.254920537 | 0.173120908 | KCNH2 | 1 |
| GO:0016605 | PML body | 1/36 | 102/19559 | 0.171716639 | 0.254920537 | 0.173120908 | TP53 | 1 |
| GO:0005796 | Golgi lumen | 1/36 | 103/19559 | 0.173249157 | 0.254920537 | 0.173120908 | F7 | 1 |
| GO:0055029 | nuclear DNA-directed RNA polymerase complex | 1/36 | 103/19559 | 0.173249157 | 0.254920537 | 0.173120908 | TP53 | 1 |
| GO:0000428 | DNA-directed RNA polymerase complex | 1/36 | 104/19559 | 0.174778918 | 0.254920537 | 0.173120908 | TP53 | 1 |
| GO:0005667 | transcription regulator complex | 2/36 | 413/19559 | 0.175945864 | 0.254920537 | 0.173120908 | JUN/TP53 | 2 |
| GO:0042470 | melanosome | 1/36 | 105/19559 | 0.176305927 | 0.254920537 | 0.173120908 | FASN | 1 |
| GO:0048770 | pigment granule | 1/36 | 105/19559 | 0.176305927 | 0.254920537 | 0.173120908 | FASN | 1 |
| GO:0005925 | focal adhesion | 2/36 | 415/19559 | 0.177264597 | 0.254920537 | 0.173120908 | SLC6A4/OPRM1 | 2 |
| GO:0000777 | condensed chromosome kinetochore | 1/36 | 106/19559 | 0.177830189 | 0.254920537 | 0.173120908 | CCNB1 | 1 |
| GO:0030175 | filopodium | 1/36 | 106/19559 | 0.177830189 | 0.254920537 | 0.173120908 | MAP2 | 1 |
| GO:0030880 | RNA polymerase complex | 1/36 | 108/19559 | 0.18087049 | 0.254920537 | 0.173120908 | TP53 | 1 |
| GO:0031252 | cell leading edge | 2/36 | 421/19559 | 0.181230735 | 0.254920537 | 0.173120908 | OPRM1/GABRA1 | 2 |
| GO:0030055 | cell-substrate junction | 2/36 | 423/19559 | 0.182555998 | 0.254920537 | 0.173120908 | SLC6A4/OPRM1 | 2 |
| GO:0062023 | collagen-containing extracellular matrix | 2/36 | 427/19559 | 0.185211178 | 0.25631904 | 0.174070656 | PRSS1/F7 | 2 |
| GO:0044853 | plasma membrane raft | 1/36 | 113/19559 | 0.1884235 | 0.258457013 | 0.175522589 | SCN5A | 1 |
| GO:0000779 | condensed chromosome, centromeric region | 1/36 | 122/19559 | 0.201848641 | 0.274443328 | 0.18637917 | CCNB1 | 1 |
| GO:0000784 | nuclear chromosome, telomeric region | 1/36 | 124/19559 | 0.204802551 | 0.276038221 | 0.187462289 | PCNA | 1 |
| GO:0030018 | Z disc | 1/36 | 128/19559 | 0.21067852 | 0.281505895 | 0.191175481 | SCN5A | 1 |
| GO:0043679 | axon terminus | 1/36 | 130/19559 | 0.213600653 | 0.281505895 | 0.191175481 | CHRM3 | 1 |
| GO:0005635 | nuclear envelope | 2/36 | 473/19559 | 0.216123881 | 0.281505895 | 0.191175481 | TP53/BAX | 2 |
| GO:0005759 | mitochondrial matrix | 2/36 | 473/19559 | 0.216123881 | 0.281505895 | 0.191175481 | TP53/CCNB1 | 2 |
| GO:0000776 | kinetochore | 1/36 | 137/19559 | 0.223745525 | 0.289004636 | 0.196268004 | CCNB1 | 1 |
| GO:0031674 | I band | 1/36 | 140/19559 | 0.228054272 | 0.29187216 | 0.198215389 | SCN5A | 1 |
| GO:0005911 | cell-cell junction | 2/36 | 493/19559 | 0.229731636 | 0.29187216 | 0.198215389 | SCN5A/PRKCD | 2 |
| GO:0044306 | neuron projection terminus | 1/36 | 149/19559 | 0.240841466 | 0.303499408 | 0.206111652 | CHRM3 | 1 |
| GO:0005766 | primary lysosome | 1/36 | 155/19559 | 0.2492516 | 0.308332464 | 0.209393864 | PRKCD | 1 |
| GO:0042582 | azurophil granule | 1/36 | 155/19559 | 0.2492516 | 0.308332464 | 0.209393864 | PRKCD | 1 |
| GO:0043204 | perikaryon | 1/36 | 156/19559 | 0.250644455 | 0.308332464 | 0.209393864 | OPRM1 | 1 |
| GO:0005875 | microtubule associated complex | 1/36 | 159/19559 | 0.25480796 | 0.310986093 | 0.211195988 | MAP2 | 1 |
| GO:0000922 | spindle pole | 1/36 | 165/19559 | 0.263067607 | 0.31855843 | 0.216338492 | CCNB1 | 1 |
| GO:0005775 | vacuolar lumen | 1/36 | 173/19559 | 0.273942134 | 0.329155277 | 0.223534993 | PRKCD | 1 |
| GO:0043197 | dendritic spine | 1/36 | 175/19559 | 0.276636294 | 0.329835581 | 0.223996999 | OPRM1 | 1 |
| GO:0044309 | neuron spine | 1/36 | 177/19559 | 0.279320732 | 0.330493996 | 0.22444414 | OPRM1 | 1 |
| GO:0030426 | growth cone | 1/36 | 184/19559 | 0.288640211 | 0.338933581 | 0.230175607 | MAP2 | 1 |
| GO:0055037 | recycling endosome | 1/36 | 190/19559 | 0.296534992 | 0.344519287 | 0.233968956 | TNF | 1 |
| GO:0030427 | site of polarized growth | 1/36 | 191/19559 | 0.29784248 | 0.344519287 | 0.233968956 | MAP2 | 1 |
| GO:0000775 | chromosome, centromeric region | 1/36 | 196/19559 | 0.304344563 | 0.349432646 | 0.237305702 | CCNB1 | 1 |
| GO:0030017 | sarcomere | 1/36 | 207/19559 | 0.31844376 | 0.362932227 | 0.246473499 | SCN5A | 1 |
| GO:0005882 | intermediate filament | 1/36 | 215/19559 | 0.32852282 | 0.371686402 | 0.252418609 | EIF6 | 1 |
| GO:0098858 | actin-based cell projection | 1/36 | 220/19559 | 0.334748485 | 0.375985617 | 0.25533828 | MAP2 | 1 |
| GO:0030016 | myofibril | 1/36 | 227/19559 | 0.343370223 | 0.382894853 | 0.26003046 | SCN5A | 1 |
| GO:0043292 | contractile fiber | 1/36 | 238/19559 | 0.356699615 | 0.394917431 | 0.268195199 | SCN5A | 1 |
| GO:0016323 | basolateral plasma membrane | 1/36 | 246/19559 | 0.366228103 | 0.402591177 | 0.273406572 | CHRM3 | 1 |
| GO:0045111 | intermediate filament cytoskeleton | 1/36 | 251/19559 | 0.372113577 | 0.406180313 | 0.275844016 | EIF6 | 1 |
| GO:0098798 | mitochondrial protein complex | 1/36 | 265/19559 | 0.388311631 | 0.420897222 | 0.285838521 | BAX | 1 |
| GO:0031965 | nuclear membrane | 1/36 | 301/19559 | 0.428120702 | 0.460824367 | 0.312953729 | TP53 | 1 |
| GO:0098802 | plasma membrane signaling receptor complex | 1/36 | 307/19559 | 0.434505891 | 0.462413123 | 0.314032681 | CHRNA2 | 1 |
| GO:0005788 | endoplasmic reticulum lumen | 1/36 | 308/19559 | 0.435563328 | 0.462413123 | 0.314032681 | F7 | 1 |
| GO:0034774 | secretory granule lumen | 1/36 | 322/19559 | 0.450167127 | 0.474666019 | 0.322353833 | PRKCD | 1 |
| GO:0060205 | cytoplasmic vesicle lumen | 1/36 | 326/19559 | 0.454271723 | 0.474687847 | 0.322368656 | PRKCD | 1 |
| GO:0031983 | vesicle lumen | 1/36 | 328/19559 | 0.456312833 | 0.474687847 | 0.322368656 | PRKCD | 1 |
| GO:0098978 | glutamatergic synapse | 1/36 | 361/19559 | 0.488938816 | 0.505236776 | 0.343114958 | CHRM3 | 1 |
| GO:0005819 | spindle | 1/36 | 367/19559 | 0.494662706 | 0.507766354 | 0.344832838 | CCNB1 | 1 |
| GO:0005769 | early endosome | 1/36 | 377/19559 | 0.504064302 | 0.51401294 | 0.349075002 | KDR | 1 |
| GO:0016607 | nuclear speck | 1/36 | 401/19559 | 0.525939641 | 0.532814669 | 0.361843578 | AR | 1 |
| GO:0009897 | external side of plasma membrane | 1/36 | 417/19559 | 0.539998936 | 0.543505423 | 0.369103853 | TNF | 1 |
| GO:0005874 | microtubule | 1/36 | 431/19559 | 0.551967617 | 0.551967617 | 0.374850674 | MAP2 | 1 |

**Table S5.** Results of MF GO terms enrichment of XBCQD in monkeypox virus.

| **ID** | **Description** | **GeneRatio** | **BgRatio** | **pvalue** | **p.adjust** | **qvalue** | **geneID** | **Count** |
| --- | --- | --- | --- | --- | --- | --- | --- | --- |
| GO:0008144 | drug binding | 5/36 | 104/18352 | 1.73918E-06 | 0.000198355 | 9.15485E-05 | FASN/SLC6A4/GABRA1/CHRNA2/CHRM3 | 5 |
| GO:0004879 | nuclear receptor activity | 4/36 | 52/18352 | 3.15648E-06 | 0.000198355 | 9.15485E-05 | ESR2/ESR1/AR/PGR | 4 |
| GO:0098531 | ligand-activated transcription factor activity | 4/36 | 52/18352 | 3.15648E-06 | 0.000198355 | 9.15485E-05 | ESR2/ESR1/AR/PGR | 4 |
| GO:0004697 | protein kinase C activity | 3/36 | 16/18352 | 3.81452E-06 | 0.000198355 | 9.15485E-05 | PRKCE/PRKCD/PRKCA | 3 |
| GO:0004698 | calcium-dependent protein kinase C activity | 3/36 | 16/18352 | 3.81452E-06 | 0.000198355 | 9.15485E-05 | PRKCE/PRKCD/PRKCA | 3 |
| GO:0035173 | histone kinase activity | 3/36 | 17/18352 | 4.62567E-06 | 0.000200446 | 9.25135E-05 | CHEK1/PRKCA/CCNB1 | 3 |
| GO:0001091 | RNA polymerase II general transcription initiation factor binding | 3/36 | 19/18352 | 6.57383E-06 | 0.000244171 | 0.000112694 | ESR1/AR/TP53 | 3 |
| GO:0098960 | postsynaptic neurotransmitter receptor activity | 4/36 | 65/18352 | 7.75197E-06 | 0.000251939 | 0.00011628 | GABRA1/CHRNA2/CHRM4/CHRM3 | 4 |
| GO:0015464 | acetylcholine receptor activity | 3/36 | 22/18352 | 1.04054E-05 | 0.0003006 | 0.000138738 | CHRNA2/CHRM4/CHRM3 | 3 |
| GO:0009931 | calcium-dependent protein serine/threonine kinase activity | 3/36 | 23/18352 | 1.19501E-05 | 0.000310702 | 0.000143401 | PRKCE/PRKCD/PRKCA | 3 |
| GO:0010857 | calcium-dependent protein kinase activity | 3/36 | 24/18352 | 1.36388E-05 | 0.000322372 | 0.000148787 | PRKCE/PRKCD/PRKCA | 3 |
| GO:0003707 | steroid hormone receptor activity | 3/36 | 26/18352 | 1.7473E-05 | 0.000378582 | 0.00017473 | ESR2/ESR1/PGR | 3 |
| GO:0001223 | transcription coactivator binding | 3/36 | 29/18352 | 2.44572E-05 | 0.000489144 | 0.000225759 | ESR1/AR/PGR | 3 |
| GO:0030291 | protein serine/threonine kinase inhibitor activity | 3/36 | 31/18352 | 3.00052E-05 | 0.000542906 | 0.000250572 | PKIA/CDKN1A/CASP3 | 3 |
| GO:0044389 | ubiquitin-like protein ligase binding | 6/36 | 316/18352 | 3.13215E-05 | 0.000542906 | 0.000250572 | JUN/SCN5A/TP53/CDKN1A/CCNB1/KCNH2 | 6 |
| GO:0005496 | steroid binding | 4/36 | 106/18352 | 5.37179E-05 | 0.000872916 | 0.000402884 | ESR2/ESR1/AR/PGR | 4 |
| GO:0030594 | neurotransmitter receptor activity | 4/36 | 111/18352 | 6.4312E-05 | 0.000983595 | 0.000453967 | GABRA1/CHRNA2/CHRM4/CHRM3 | 4 |
| GO:0140296 | general transcription initiation factor binding | 3/36 | 44/18352 | 8.68717E-05 | 0.001254813 | 0.000579144 | ESR1/AR/TP53 | 3 |
| GO:0016538 | cyclin-dependent protein serine/threonine kinase regulator activity | 3/36 | 50/18352 | 0.000127528 | 0.001745118 | 0.000805439 | CDKN1A/CCNB1/CASP3 | 3 |
| GO:0001221 | transcription cofactor binding | 3/36 | 51/18352 | 0.000135316 | 0.001759108 | 0.000811896 | ESR1/AR/PGR | 3 |
| GO:0099528 | G protein-coupled neurotransmitter receptor activity | 2/36 | 10/18352 | 0.000166704 | 0.00206396 | 0.000952597 | CHRM4/CHRM3 | 2 |
| GO:0004861 | cyclin-dependent protein serine/threonine kinase inhibitor activity | 2/36 | 12/18352 | 0.000243897 | 0.002882417 | 0.001330346 | CDKN1A/CASP3 | 2 |
| GO:0031625 | ubiquitin protein ligase binding | 5/36 | 297/18352 | 0.000268181 | 0.002987825 | 0.001378996 | JUN/SCN5A/TP53/CDKN1A/KCNH2 | 5 |
| GO:0004860 | protein kinase inhibitor activity | 3/36 | 65/18352 | 0.000278523 | 0.002987825 | 0.001378996 | PKIA/CDKN1A/CASP3 | 3 |
| GO:0042166 | acetylcholine binding | 2/36 | 13/18352 | 0.000287886 | 0.002987825 | 0.001378996 | CHRNA2/CHRM3 | 2 |
| GO:0015267 | channel activity | 6/36 | 479/18352 | 0.000306829 | 0.002987825 | 0.001378996 | SCN5A/BAX/OPRM1/KCNH2/GABRA1/CHRNA2 | 6 |
| GO:0022803 | passive transmembrane transporter activity | 6/36 | 480/18352 | 0.000310274 | 0.002987825 | 0.001378996 | SCN5A/BAX/OPRM1/KCNH2/GABRA1/CHRNA2 | 6 |
| GO:0019210 | kinase inhibitor activity | 3/36 | 69/18352 | 0.000332294 | 0.003007507 | 0.00138808 | PKIA/CDKN1A/CASP3 | 3 |
| GO:0050998 | nitric-oxide synthase binding | 2/36 | 14/18352 | 0.000335453 | 0.003007507 | 0.00138808 | SCN5A/SLC6A4 | 2 |
| GO:0001098 | basal transcription machinery binding | 3/36 | 72/18352 | 0.000376726 | 0.003159639 | 0.001458295 | ESR1/AR/TP53 | 3 |
| GO:0001099 | basal RNA polymerase II transcription machinery binding | 3/36 | 72/18352 | 0.000376726 | 0.003159639 | 0.001458295 | ESR1/AR/TP53 | 3 |
| GO:0019887 | protein kinase regulator activity | 4/36 | 185/18352 | 0.000457575 | 0.003717796 | 0.001715906 | PKIA/CDKN1A/CCNB1/CASP3 | 4 |
| GO:0022836 | gated channel activity | 5/36 | 337/18352 | 0.000478677 | 0.003771398 | 0.001740645 | SCN5A/OPRM1/KCNH2/GABRA1/CHRNA2 | 5 |
| GO:0140297 | DNA-binding transcription factor binding | 5/36 | 347/18352 | 0.00054675 | 0.004181032 | 0.001929707 | ESR1/NCOA2/JUN/PCNA/MYC | 5 |
| GO:0051117 | ATPase binding | 3/36 | 88/18352 | 0.000678411 | 0.005039624 | 0.00232598 | ESR1/AR/PGR | 3 |
| GO:0042165 | neurotransmitter binding | 2/36 | 21/18352 | 0.000767464 | 0.005542798 | 0.002558214 | CHRNA2/CHRM3 | 2 |
| GO:0019207 | kinase regulator activity | 4/36 | 216/18352 | 0.000818268 | 0.005749989 | 0.002653841 | PKIA/CDKN1A/CCNB1/CASP3 | 4 |
| GO:0016922 | nuclear receptor binding | 3/36 | 101/18352 | 0.001012423 | 0.006927106 | 0.003197126 | ESR1/NCOA2/PCNA | 3 |
| GO:0035035 | histone acetyltransferase binding | 2/36 | 28/18352 | 0.001369565 | 0.009130433 | 0.004214046 | TP53/PCNA | 2 |
| GO:0005216 | ion channel activity | 5/36 | 431/18352 | 0.001443862 | 0.0093851 | 0.004331585 | SCN5A/OPRM1/KCNH2/GABRA1/CHRNA2 | 5 |
| GO:0061629 | RNA polymerase II-specific DNA-binding transcription factor binding | 4/36 | 267/18352 | 0.001789145 | 0.011345801 | 0.005236523 | ESR1/NCOA2/JUN/PCNA | 4 |
| GO:0004993 | G protein-coupled serotonin receptor activity | 2/36 | 34/18352 | 0.002017639 | 0.012199678 | 0.005630621 | CHRM4/CHRM3 | 2 |
| GO:0099589 | serotonin receptor activity | 2/36 | 34/18352 | 0.002017639 | 0.012199678 | 0.005630621 | CHRM4/CHRM3 | 2 |
| GO:0002020 | protease binding | 3/36 | 137/18352 | 0.002426695 | 0.014291815 | 0.006596222 | TP53/TNF/CASP3 | 3 |
| GO:0015276 | ligand-gated ion channel activity | 3/36 | 139/18352 | 0.002528552 | 0.014291815 | 0.006596222 | KCNH2/GABRA1/CHRNA2 | 3 |
| GO:0022834 | ligand-gated channel activity | 3/36 | 139/18352 | 0.002528552 | 0.014291815 | 0.006596222 | KCNH2/GABRA1/CHRNA2 | 3 |
| GO:0005178 | integrin binding | 3/36 | 144/18352 | 0.002794688 | 0.015137894 | 0.00698672 | KDR/PRKCA/IL1B | 3 |
| GO:0035257 | nuclear hormone receptor binding | 3/36 | 144/18352 | 0.002794688 | 0.015137894 | 0.00698672 | ESR1/NCOA2/PCNA | 3 |
| GO:0030331 | estrogen receptor binding | 2/36 | 42/18352 | 0.003066246 | 0.016269876 | 0.007509173 | ESR1/PCNA | 2 |
| GO:0001046 | core promoter sequence-specific DNA binding | 2/36 | 46/18352 | 0.003667814 | 0.019072631 | 0.008802753 | TP53/MYC | 2 |
| GO:1904315 | transmitter-gated ion channel activity involved in regulation of postsynaptic membrane potential | 2/36 | 47/18352 | 0.00382612 | 0.019505708 | 0.009002634 | GABRA1/CHRNA2 | 2 |
| GO:0032813 | tumor necrosis factor receptor superfamily binding | 2/36 | 48/18352 | 0.003987567 | 0.019671366 | 0.009079092 | TNF/CASP3 | 2 |
| GO:0005261 | cation channel activity | 4/36 | 334/18352 | 0.004009932 | 0.019671366 | 0.009079092 | SCN5A/OPRM1/KCNH2/CHRNA2 | 4 |
| GO:0099529 | neurotransmitter receptor activity involved in regulation of postsynaptic membrane potential | 2/36 | 50/18352 | 0.004319834 | 0.020799203 | 0.009599632 | GABRA1/CHRNA2 | 2 |
| GO:0008227 | G protein-coupled amine receptor activity | 2/36 | 51/18352 | 0.004490631 | 0.021228438 | 0.009797741 | CHRM4/CHRM3 | 2 |
| GO:0051427 | hormone receptor binding | 3/36 | 177/18352 | 0.004985143 | 0.023145307 | 0.010682449 | ESR1/NCOA2/PCNA | 3 |
| GO:0001085 | RNA polymerase II transcription factor binding | 2/36 | 56/18352 | 0.005390757 | 0.024589419 | 0.011348963 | AR/TP53 | 2 |
| GO:0022824 | transmitter-gated ion channel activity | 2/36 | 60/18352 | 0.006165509 | 0.026717206 | 0.012331018 | GABRA1/CHRNA2 | 2 |
| GO:0022835 | transmitter-gated channel activity | 2/36 | 60/18352 | 0.006165509 | 0.026717206 | 0.012331018 | GABRA1/CHRNA2 | 2 |
| GO:0097110 | scaffold protein binding | 2/36 | 60/18352 | 0.006165509 | 0.026717206 | 0.012331018 | SCN5A/KCNH2 | 2 |
| GO:0005244 | voltage-gated ion channel activity | 3/36 | 197/18352 | 0.006703402 | 0.028111042 | 0.012974327 | SCN5A/OPRM1/KCNH2 | 3 |
| GO:0022832 | voltage-gated channel activity | 3/36 | 197/18352 | 0.006703402 | 0.028111042 | 0.012974327 | SCN5A/OPRM1/KCNH2 | 3 |
| GO:0030971 | receptor tyrosine kinase binding | 2/36 | 72/18352 | 0.008773289 | 0.036207224 | 0.016711026 | TP53/PCNA | 2 |
| GO:0005230 | extracellular ligand-gated ion channel activity | 2/36 | 74/18352 | 0.009248288 | 0.037571172 | 0.017340541 | GABRA1/CHRNA2 | 2 |
| GO:0046873 | metal ion transmembrane transporter activity | 4/36 | 428/18352 | 0.009528879 | 0.038115514 | 0.017591776 | SCN5A/SLC6A4/OPRM1/KCNH2 | 4 |
| GO:0004674 | protein serine/threonine kinase activity | 4/36 | 435/18352 | 0.010071912 | 0.039677231 | 0.018312568 | CHEK1/PRKCE/PRKCD/PRKCA | 4 |
| GO:0033613 | activating transcription factor binding | 2/36 | 80/18352 | 0.010740661 | 0.041680177 | 0.019237005 | JUN/MYC | 2 |
| GO:0035258 | steroid hormone receptor binding | 2/36 | 81/18352 | 0.010999097 | 0.042055369 | 0.019410171 | ESR1/PCNA | 2 |
| GO:0030295 | protein kinase activator activity | 2/36 | 82/18352 | 0.011260276 | 0.042430025 | 0.019583088 | CDKN1A/CCNB1 | 2 |
| GO:0008013 | beta-catenin binding | 2/36 | 85/18352 | 0.012060163 | 0.044794892 | 0.020674565 | ESR1/AR | 2 |
| GO:0008081 | phosphoric diester hydrolase activity | 2/36 | 89/18352 | 0.013164417 | 0.047538173 | 0.021940695 | PDE3A/CHRM3 | 2 |
| GO:0019209 | kinase activator activity | 2/36 | 89/18352 | 0.013164417 | 0.047538173 | 0.021940695 | CDKN1A/CCNB1 | 2 |
| GO:1990782 | protein tyrosine kinase binding | 2/36 | 98/18352 | 0.015803271 | 0.055783099 | 0.025746046 | TP53/PCNA | 2 |
| GO:0005126 | cytokine receptor binding | 3/36 | 271/18352 | 0.015876728 | 0.055783099 | 0.025746046 | TNF/IL1B/CASP3 | 3 |
| GO:0015108 | chloride transmembrane transporter activity | 2/36 | 102/18352 | 0.017043078 | 0.059082671 | 0.027268925 | SLC6A4/GABRA1 | 2 |
| GO:0051087 | chaperone binding | 2/36 | 104/18352 | 0.017678121 | 0.060477783 | 0.027912823 | TP53/BAX | 2 |
| GO:0099094 | ligand-gated cation channel activity | 2/36 | 108/18352 | 0.018978066 | 0.061667115 | 0.028461746 | KCNH2/CHRNA2 | 2 |
| GO:0001094 | TFIID-class transcription factor complex binding | 1/36 | 10/18352 | 0.019448859 | 0.061667115 | 0.028461746 | TP53 | 1 |
| GO:0002162 | dystroglycan binding | 1/36 | 10/18352 | 0.019448859 | 0.061667115 | 0.028461746 | MAP2 | 1 |
| GO:0008603 | cAMP-dependent protein kinase regulator activity | 1/36 | 10/18352 | 0.019448859 | 0.061667115 | 0.028461746 | PKIA | 1 |
| GO:0043560 | insulin receptor substrate binding | 1/36 | 10/18352 | 0.019448859 | 0.061667115 | 0.028461746 | PRKCD | 1 |
| GO:0097199 | cysteine-type endopeptidase activity involved in apoptotic signaling pathway | 1/36 | 10/18352 | 0.019448859 | 0.061667115 | 0.028461746 | CASP3 | 1 |
| GO:0004312 | fatty acid synthase activity | 1/36 | 11/18352 | 0.021373396 | 0.063148669 | 0.029145539 | FASN | 1 |
| GO:0008503 | benzodiazepine receptor activity | 1/36 | 11/18352 | 0.021373396 | 0.063148669 | 0.029145539 | GABRA1 | 1 |
| GO:0015378 | sodium:chloride symporter activity | 1/36 | 11/18352 | 0.021373396 | 0.063148669 | 0.029145539 | SLC6A4 | 1 |
| GO:0032404 | mismatch repair complex binding | 1/36 | 11/18352 | 0.021373396 | 0.063148669 | 0.029145539 | PCNA | 1 |
| GO:0051400 | BH domain binding | 1/36 | 11/18352 | 0.021373396 | 0.063148669 | 0.029145539 | BAX | 1 |
| GO:0070513 | death domain binding | 1/36 | 11/18352 | 0.021373396 | 0.063148669 | 0.029145539 | BAX | 1 |
| GO:0004115 | 3',5'-cyclic-AMP phosphodiesterase activity | 1/36 | 12/18352 | 0.023294259 | 0.064430929 | 0.029737352 | PDE3A | 1 |
| GO:0016004 | phospholipase activator activity | 1/36 | 12/18352 | 0.023294259 | 0.064430929 | 0.029737352 | CASP3 | 1 |
| GO:0043023 | ribosomal large subunit binding | 1/36 | 12/18352 | 0.023294259 | 0.064430929 | 0.029737352 | EIF6 | 1 |
| GO:0043176 | amine binding | 1/36 | 12/18352 | 0.023294259 | 0.064430929 | 0.029737352 | SLC6A4 | 1 |
| GO:0051378 | serotonin binding | 1/36 | 12/18352 | 0.023294259 | 0.064430929 | 0.029737352 | SLC6A4 | 1 |
| GO:1902282 | voltage-gated potassium channel activity involved in ventricular cardiac muscle cell action potential repolarization | 1/36 | 12/18352 | 0.023294259 | 0.064430929 | 0.029737352 | KCNH2 | 1 |
| GO:0001162 | RNA polymerase II intronic transcription regulatory region sequence-specific DNA binding | 1/36 | 13/18352 | 0.025211457 | 0.066211907 | 0.030559342 | NCOA2 | 1 |
| GO:0019104 | DNA N-glycosylase activity | 1/36 | 13/18352 | 0.025211457 | 0.066211907 | 0.030559342 | PCNA | 1 |
| GO:0022851 | GABA-gated chloride ion channel activity | 1/36 | 13/18352 | 0.025211457 | 0.066211907 | 0.030559342 | GABRA1 | 1 |
| GO:0031005 | filamin binding | 1/36 | 13/18352 | 0.025211457 | 0.066211907 | 0.030559342 | OPRM1 | 1 |
| GO:0034236 | protein kinase A catalytic subunit binding | 1/36 | 13/18352 | 0.025211457 | 0.066211907 | 0.030559342 | PKIA | 1 |
| GO:0031072 | heat shock protein binding | 2/36 | 127/18352 | 0.025677782 | 0.066762234 | 0.030813339 | KDR/BAX | 2 |
| GO:0015373 | anion:sodium symporter activity | 1/36 | 14/18352 | 0.027124996 | 0.067812489 | 0.031298072 | SLC6A4 | 1 |
| GO:0030983 | mismatched DNA binding | 1/36 | 14/18352 | 0.027124996 | 0.067812489 | 0.031298072 | PCNA | 1 |
| GO:0035497 | cAMP response element binding | 1/36 | 14/18352 | 0.027124996 | 0.067812489 | 0.031298072 | JUN | 1 |
| GO:0060229 | lipase activator activity | 1/36 | 14/18352 | 0.027124996 | 0.067812489 | 0.031298072 | CASP3 | 1 |
| GO:0004713 | protein tyrosine kinase activity | 2/36 | 135/18352 | 0.028747004 | 0.069509663 | 0.032081383 | KDR/PRKCD | 2 |
| GO:0005237 | inhibitory extracellular ligand-gated ion channel activity | 1/36 | 15/18352 | 0.029034882 | 0.069509663 | 0.032081383 | GABRA1 | 1 |
| GO:0086008 | voltage-gated potassium channel activity involved in cardiac muscle cell action potential repolarization | 1/36 | 15/18352 | 0.029034882 | 0.069509663 | 0.032081383 | KCNH2 | 1 |
| GO:0097153 | cysteine-type endopeptidase activity involved in apoptotic process | 1/36 | 15/18352 | 0.029034882 | 0.069509663 | 0.032081383 | CASP3 | 1 |
| GO:0019838 | growth factor binding | 2/36 | 136/18352 | 0.02914059 | 0.069509663 | 0.032081383 | SCN5A/KDR | 2 |
| GO:0022843 | voltage-gated cation channel activity | 2/36 | 140/18352 | 0.030736592 | 0.069953844 | 0.03228639 | OPRM1/KCNH2 | 2 |
| GO:0001161 | intronic transcription regulatory region sequence-specific DNA binding | 1/36 | 16/18352 | 0.030941123 | 0.069953844 | 0.03228639 | NCOA2 | 1 |
| GO:0005149 | interleukin-1 receptor binding | 1/36 | 16/18352 | 0.030941123 | 0.069953844 | 0.03228639 | IL1B | 1 |
| GO:0022848 | acetylcholine-gated cation-selective channel activity | 1/36 | 16/18352 | 0.030941123 | 0.069953844 | 0.03228639 | CHRNA2 | 1 |
| GO:0070402 | NADPH binding | 1/36 | 16/18352 | 0.030941123 | 0.069953844 | 0.03228639 | FASN | 1 |
| GO:0071837 | HMG box domain binding | 1/36 | 16/18352 | 0.030941123 | 0.069953844 | 0.03228639 | JUN | 1 |
| GO:0015103 | inorganic anion transmembrane transporter activity | 2/36 | 145/18352 | 0.032779618 | 0.072986057 | 0.033685873 | SLC6A4/GABRA1 | 2 |
| GO:0015377 | cation:chloride symporter activity | 1/36 | 17/18352 | 0.032843726 | 0.072986057 | 0.033685873 | SLC6A4 | 1 |
| GO:0042578 | phosphoric ester hydrolase activity | 3/36 | 369/18352 | 0.035297344 | 0.077773809 | 0.035895604 | PON1/PDE3A/CHRM3 | 3 |
| GO:0015081 | sodium ion transmembrane transporter activity | 2/36 | 152/18352 | 0.0357274 | 0.078059866 | 0.036027631 | SCN5A/SLC6A4 | 2 |
| GO:0004890 | GABA-A receptor activity | 1/36 | 19/18352 | 0.036638042 | 0.078081073 | 0.036037418 | GABRA1 | 1 |
| GO:0005326 | neurotransmitter transmembrane transporter activity | 1/36 | 19/18352 | 0.036638042 | 0.078081073 | 0.036037418 | SLC6A4 | 1 |
| GO:0099095 | ligand-gated anion channel activity | 1/36 | 19/18352 | 0.036638042 | 0.078081073 | 0.036037418 | GABRA1 | 1 |
| GO:0015077 | monovalent inorganic cation transmembrane transporter activity | 3/36 | 381/18352 | 0.038252359 | 0.07872504 | 0.036334634 | SCN5A/SLC6A4/KCNH2 | 3 |
| GO:0005123 | death receptor binding | 1/36 | 20/18352 | 0.038529769 | 0.07872504 | 0.036334634 | CASP3 | 1 |
| GO:0030506 | ankyrin binding | 1/36 | 20/18352 | 0.038529769 | 0.07872504 | 0.036334634 | SCN5A | 1 |
| GO:0031681 | G-protein beta-subunit binding | 1/36 | 20/18352 | 0.038529769 | 0.07872504 | 0.036334634 | OPRM1 | 1 |
| GO:0070182 | DNA polymerase binding | 1/36 | 20/18352 | 0.038529769 | 0.07872504 | 0.036334634 | PCNA | 1 |
| GO:0004857 | enzyme inhibitor activity | 3/36 | 383/18352 | 0.038756943 | 0.07872504 | 0.036334634 | PKIA/CDKN1A/CASP3 | 3 |
| GO:0005242 | inward rectifier potassium channel activity | 1/36 | 22/18352 | 0.042302394 | 0.08395895 | 0.038750285 | KCNH2 | 1 |
| GO:0015296 | anion:cation symporter activity | 1/36 | 22/18352 | 0.042302394 | 0.08395895 | 0.038750285 | SLC6A4 | 1 |
| GO:0016917 | GABA receptor activity | 1/36 | 22/18352 | 0.042302394 | 0.08395895 | 0.038750285 | GABRA1 | 1 |
| GO:0004252 | serine-type endopeptidase activity | 2/36 | 169/18352 | 0.043293518 | 0.084468085 | 0.03898527 | PRSS1/F7 | 2 |
| GO:0004114 | 3',5'-cyclic-nucleotide phosphodiesterase activity | 1/36 | 23/18352 | 0.044183306 | 0.084468085 | 0.03898527 | PDE3A | 1 |
| GO:0005248 | voltage-gated sodium channel activity | 1/36 | 23/18352 | 0.044183306 | 0.084468085 | 0.03898527 | SCN5A | 1 |
| GO:0017134 | fibroblast growth factor binding | 1/36 | 23/18352 | 0.044183306 | 0.084468085 | 0.03898527 | SCN5A | 1 |
| GO:0070412 | R-SMAD binding | 1/36 | 23/18352 | 0.044183306 | 0.084468085 | 0.03898527 | JUN | 1 |
| GO:0015101 | organic cation transmembrane transporter activity | 1/36 | 24/18352 | 0.046060626 | 0.087414327 | 0.040345074 | SLC6A4 | 1 |
| GO:0004112 | cyclic-nucleotide phosphodiesterase activity | 1/36 | 25/18352 | 0.047934361 | 0.088692977 | 0.04093522 | PDE3A | 1 |
| GO:0004190 | aspartic-type endopeptidase activity | 1/36 | 25/18352 | 0.047934361 | 0.088692977 | 0.04093522 | CASP3 | 1 |
| GO:0001965 | G-protein alpha-subunit binding | 1/36 | 26/18352 | 0.049804518 | 0.088692977 | 0.04093522 | OPRM1 | 1 |
| GO:0004435 | phosphatidylinositol phospholipase C activity | 1/36 | 26/18352 | 0.049804518 | 0.088692977 | 0.04093522 | CHRM3 | 1 |
| GO:0016628 | oxidoreductase activity, acting on the CH-CH group of donors, NAD or NADP as acceptor | 1/36 | 26/18352 | 0.049804518 | 0.088692977 | 0.04093522 | FASN | 1 |
| GO:0017025 | TBP-class protein binding | 1/36 | 26/18352 | 0.049804518 | 0.088692977 | 0.04093522 | ESR1 | 1 |
| GO:0017075 | syntaxin-1 binding | 1/36 | 26/18352 | 0.049804518 | 0.088692977 | 0.04093522 | SLC6A4 | 1 |
| GO:0042923 | neuropeptide binding | 1/36 | 26/18352 | 0.049804518 | 0.088692977 | 0.04093522 | OPRM1 | 1 |
| GO:0070001 | aspartic-type peptidase activity | 1/36 | 26/18352 | 0.049804518 | 0.088692977 | 0.04093522 | CASP3 | 1 |
| GO:0008236 | serine-type peptidase activity | 2/36 | 187/18352 | 0.051894558 | 0.091786293 | 0.042362905 | PRSS1/F7 | 2 |
| GO:0008022 | protein C-terminus binding | 2/36 | 189/18352 | 0.052885571 | 0.092779378 | 0.042821252 | PCNA/OPRM1 | 2 |
| GO:0003785 | actin monomer binding | 1/36 | 28/18352 | 0.053534123 | 0.092779378 | 0.042821252 | PRKCE | 1 |
| GO:0004629 | phospholipase C activity | 1/36 | 28/18352 | 0.053534123 | 0.092779378 | 0.042821252 | CHRM3 | 1 |
| GO:0017171 | serine hydrolase activity | 2/36 | 191/18352 | 0.053883408 | 0.092779378 | 0.042821252 | PRSS1/F7 | 2 |
| GO:0004175 | endopeptidase activity | 3/36 | 440/18352 | 0.054562996 | 0.09333144 | 0.043076049 | PRSS1/F7/CASP3 | 3 |
| GO:0005231 | excitatory extracellular ligand-gated ion channel activity | 1/36 | 30/18352 | 0.057249494 | 0.09665499 | 0.044609995 | CHRNA2 | 1 |
| GO:0030332 | cyclin binding | 1/36 | 30/18352 | 0.057249494 | 0.09665499 | 0.044609995 | CDKN1A | 1 |
| GO:0005516 | calmodulin binding | 2/36 | 200/18352 | 0.058456387 | 0.098055875 | 0.045256558 | SCN5A/MAP2 | 2 |
| GO:0005164 | tumor necrosis factor receptor binding | 1/36 | 31/18352 | 0.059101858 | 0.098503097 | 0.045462968 | TNF | 1 |
| GO:0016417 | S-acyltransferase activity | 1/36 | 32/18352 | 0.060950684 | 0.099044861 | 0.045713013 | FASN | 1 |
| GO:0048019 | receptor antagonist activity | 1/36 | 32/18352 | 0.060950684 | 0.099044861 | 0.045713013 | ESR2 | 1 |
| GO:0051721 | protein phosphatase 2A binding | 1/36 | 32/18352 | 0.060950684 | 0.099044861 | 0.045713013 | TP53 | 1 |
| GO:0071889 | 14-3-3 protein binding | 1/36 | 32/18352 | 0.060950684 | 0.099044861 | 0.045713013 | PRKCE | 1 |
| GO:0005251 | delayed rectifier potassium channel activity | 1/36 | 33/18352 | 0.062795977 | 0.101409653 | 0.046804455 | KCNH2 | 1 |
| GO:0016799 | hydrolase activity, hydrolyzing N-glycosyl compounds | 1/36 | 34/18352 | 0.064637745 | 0.103739591 | 0.047879811 | PCNA | 1 |
| GO:0097718 | disordered domain specific binding | 1/36 | 36/18352 | 0.06831073 | 0.108961901 | 0.050290108 | TP53 | 1 |
| GO:0030546 | signaling receptor activator activity | 3/36 | 492/18352 | 0.071296568 | 0.113031144 | 0.05216822 | TNF/PRKCE/IL1B | 3 |
| GO:0043539 | protein serine/threonine kinase activator activity | 1/36 | 38/18352 | 0.071969691 | 0.113406786 | 0.052341594 | CCNB1 | 1 |
| GO:0016790 | thiolester hydrolase activity | 1/36 | 40/18352 | 0.07561468 | 0.118432632 | 0.054661215 | FASN | 1 |
| GO:0030547 | receptor inhibitor activity | 1/36 | 41/18352 | 0.077431952 | 0.119838616 | 0.05531013 | ESR2 | 1 |
| GO:0005125 | cytokine activity | 2/36 | 235/18352 | 0.077434183 | 0.119838616 | 0.05531013 | TNF/IL1B | 2 |
| GO:0030544 | Hsp70 protein binding | 1/36 | 42/18352 | 0.079245749 | 0.121199381 | 0.055938176 | BAX | 1 |
| GO:0051879 | Hsp90 protein binding | 1/36 | 42/18352 | 0.079245749 | 0.121199381 | 0.055938176 | KDR | 1 |
| GO:0005245 | voltage-gated calcium channel activity | 1/36 | 44/18352 | 0.08286295 | 0.125257947 | 0.05781136 | OPRM1 | 1 |
| GO:0005272 | sodium channel activity | 1/36 | 44/18352 | 0.08286295 | 0.125257947 | 0.05781136 | SCN5A | 1 |
| GO:0008188 | neuropeptide receptor activity | 1/36 | 45/18352 | 0.084666365 | 0.12651296 | 0.058390597 | OPRM1 | 1 |
| GO:0048156 | tau protein binding | 1/36 | 45/18352 | 0.084666365 | 0.12651296 | 0.058390597 | MAP2 | 1 |
| GO:0004715 | non-membrane spanning protein tyrosine kinase activity | 1/36 | 46/18352 | 0.086466333 | 0.128464267 | 0.0592912 | PRKCD | 1 |
| GO:0001102 | RNA polymerase II activating transcription factor binding | 1/36 | 47/18352 | 0.08826286 | 0.130388315 | 0.060179223 | JUN | 1 |
| GO:1901618 | organic hydroxy compound transmembrane transporter activity | 1/36 | 48/18352 | 0.090055951 | 0.132285578 | 0.061054882 | SLC6A4 | 1 |
| GO:0070888 | E-box binding | 1/36 | 50/18352 | 0.093631854 | 0.13676563 | 0.063122598 | MYC | 1 |
| GO:0003743 | translation initiation factor activity | 1/36 | 51/18352 | 0.095414679 | 0.137821202 | 0.063609786 | EIF6 | 1 |
| GO:0051018 | protein kinase A binding | 1/36 | 51/18352 | 0.095414679 | 0.137821202 | 0.063609786 | PKIA | 1 |
| GO:0030374 | nuclear receptor transcription coactivator activity | 1/36 | 54/18352 | 0.10074272 | 0.143918171 | 0.066423771 | NCOA2 | 1 |
| GO:0050661 | NADP binding | 1/36 | 54/18352 | 0.10074272 | 0.143918171 | 0.066423771 | FASN | 1 |
| GO:0043022 | ribosome binding | 1/36 | 57/18352 | 0.106040245 | 0.149839477 | 0.069156682 | EIF6 | 1 |
| GO:0043621 | protein self-association | 1/36 | 57/18352 | 0.106040245 | 0.149839477 | 0.069156682 | TP53 | 1 |
| GO:1990841 | promoter-specific chromatin binding | 1/36 | 58/18352 | 0.107799335 | 0.151501769 | 0.069923893 | TP53 | 1 |
| GO:0016627 | oxidoreductase activity, acting on the CH-CH group of donors | 1/36 | 59/18352 | 0.10955506 | 0.152322543 | 0.070302712 | FASN | 1 |
| GO:0016836 | hydro-lyase activity | 1/36 | 59/18352 | 0.10955506 | 0.152322543 | 0.070302712 | FASN | 1 |
| GO:0005507 | copper ion binding | 1/36 | 60/18352 | 0.111307425 | 0.153935801 | 0.071047293 | TP53 | 1 |
| GO:0004714 | transmembrane receptor protein tyrosine kinase activity | 1/36 | 61/18352 | 0.113056437 | 0.155527374 | 0.071781865 | KDR | 1 |
| GO:0003684 | damaged DNA binding | 1/36 | 65/18352 | 0.120019081 | 0.164236637 | 0.075801525 | PCNA | 1 |
| GO:0002039 | p53 binding | 1/36 | 66/18352 | 0.121751422 | 0.16573492 | 0.07649304 | TP53 | 1 |
| GO:0046982 | protein heterodimerization activity | 2/36 | 321/18352 | 0.130527213 | 0.176755602 | 0.081579508 | TP53/BAX | 2 |
| GO:0015370 | solute:sodium symporter activity | 1/36 | 72/18352 | 0.13207605 | 0.177926285 | 0.082119824 | SLC6A4 | 1 |
| GO:0016835 | carbon-oxygen lyase activity | 1/36 | 74/18352 | 0.135491296 | 0.180655061 | 0.083379259 | FASN | 1 |
| GO:0070491 | repressing transcription factor binding | 1/36 | 74/18352 | 0.135491296 | 0.180655061 | 0.083379259 | MYC | 1 |
| GO:0005254 | chloride channel activity | 1/36 | 75/18352 | 0.137194016 | 0.181908163 | 0.083957614 | GABRA1 | 1 |
| GO:0045296 | cadherin binding | 2/36 | 332/18352 | 0.137830416 | 0.181908163 | 0.083957614 | KDR/FASN | 2 |
| GO:0001227 | DNA-binding transcription repressor activity, RNA polymerase II-specific | 2/36 | 335/18352 | 0.139838326 | 0.183579744 | 0.084729113 | JUN/MYC | 2 |
| GO:0001217 | DNA-binding transcription repressor activity | 2/36 | 336/18352 | 0.140509112 | 0.183579744 | 0.084729113 | JUN/MYC | 2 |
| GO:0019905 | syntaxin binding | 1/36 | 78/18352 | 0.142282636 | 0.184967426 | 0.085369581 | SLC6A4 | 1 |
| GO:0046332 | SMAD binding | 1/36 | 79/18352 | 0.143972349 | 0.18623289 | 0.085953641 | JUN | 1 |
| GO:0019199 | transmembrane receptor protein kinase activity | 1/36 | 80/18352 | 0.145658826 | 0.187481658 | 0.086529996 | KDR | 1 |
| GO:0008135 | translation factor activity, RNA binding | 1/36 | 84/18352 | 0.15237249 | 0.194200233 | 0.089630877 | EIF6 | 1 |
| GO:0042562 | hormone binding | 1/36 | 84/18352 | 0.15237249 | 0.194200233 | 0.089630877 | AR | 1 |
| GO:0008509 | anion transmembrane transporter activity | 2/36 | 357/18352 | 0.154756939 | 0.196277093 | 0.090589428 | SLC6A4/GABRA1 | 2 |
| GO:0043178 | alcohol binding | 1/36 | 86/18352 | 0.155710059 | 0.196527259 | 0.090704889 | PRKCE | 1 |
| GO:0005253 | anion channel activity | 1/36 | 87/18352 | 0.157374049 | 0.197667888 | 0.091231333 | GABRA1 | 1 |
| GO:0005249 | voltage-gated potassium channel activity | 1/36 | 88/18352 | 0.15903485 | 0.198793563 | 0.091750875 | KCNH2 | 1 |
| GO:0072341 | modified amino acid binding | 1/36 | 90/18352 | 0.162346911 | 0.201962664 | 0.093213537 | FASN | 1 |
| GO:0033218 | amide binding | 2/36 | 381/18352 | 0.171376126 | 0.212179965 | 0.097929215 | FASN/OPRM1 | 2 |
| GO:0015294 | solute:cation symporter activity | 1/36 | 98/18352 | 0.175468791 | 0.216217467 | 0.099792677 | SLC6A4 | 1 |
| GO:0004620 | phospholipase activity | 1/36 | 105/18352 | 0.186786327 | 0.228002089 | 0.105231733 | CHRM3 | 1 |
| GO:0016407 | acetyltransferase activity | 1/36 | 105/18352 | 0.186786327 | 0.228002089 | 0.105231733 | FASN | 1 |
| GO:0047485 | protein N-terminus binding | 1/36 | 107/18352 | 0.189992072 | 0.230831489 | 0.10653761 | TP53 | 1 |
| GO:0090079 | translation regulator activity, nucleic acid binding | 1/36 | 109/18352 | 0.193185529 | 0.23361971 | 0.107824481 | EIF6 | 1 |
| GO:0004197 | cysteine-type endopeptidase activity | 1/36 | 114/18352 | 0.201115714 | 0.241743506 | 0.111573926 | CASP3 | 1 |
| GO:0000149 | SNARE binding | 1/36 | 115/18352 | 0.202692632 | 0.241743506 | 0.111573926 | SLC6A4 | 1 |
| GO:0042826 | histone deacetylase binding | 1/36 | 115/18352 | 0.202692632 | 0.241743506 | 0.111573926 | TP53 | 1 |
| GO:0003730 | mRNA 3'-UTR binding | 1/36 | 117/18352 | 0.205837395 | 0.244373163 | 0.112787614 | TP53 | 1 |
| GO:0005262 | calcium channel activity | 1/36 | 118/18352 | 0.207405251 | 0.245115297 | 0.113130137 | OPRM1 | 1 |
| GO:0005267 | potassium channel activity | 1/36 | 120/18352 | 0.21053194 | 0.246245066 | 0.113651569 | KCNH2 | 1 |
| GO:0016616 | oxidoreductase activity, acting on the CH-OH group of donors, NAD or NADP as acceptor | 1/36 | 120/18352 | 0.21053194 | 0.246245066 | 0.113651569 | FASN | 1 |
| GO:0003779 | actin binding | 2/36 | 437/18352 | 0.211202499 | 0.246245066 | 0.113651569 | PRKCE/SLC6A4 | 2 |
| GO:0016298 | lipase activity | 1/36 | 130/18352 | 0.225986381 | 0.261139818 | 0.12052607 | CHRM3 | 1 |
| GO:0016614 | oxidoreductase activity, acting on CH-OH group of donors | 1/36 | 130/18352 | 0.225986381 | 0.261139818 | 0.12052607 | FASN | 1 |
| GO:0044325 | ion channel binding | 1/36 | 132/18352 | 0.229041779 | 0.262925088 | 0.12135004 | SCN5A | 1 |
[truncated: 28,311 more chars]
